# Textual overlap rather than domain alignment: A comparative study of fine-tuning strategies for specialised machine translation with large language models

**DOI:** 10.1371/journal.pone.0352256

**Published:** 2026-07-20

**Authors:** Lixue Yang, Jiaxin Zhu, Zengxin Zhang

**Affiliations:** School of Foreign Languages, Tianjin University of Technology and Education, Tianjin, China; Commonwealth Scientific and Industrial Research Organisation, AUSTRALIA

## Abstract

General-purpose large language models (LLMs) may struggle in specialised machine translation, but the conditions under which fine-tuning improves translation performance remain unclear. This study compares full-parameter fine-tuning (FPFT) and parameter-efficient fine-tuning (PEFT) for Chinese-English political discourse translation using a purpose-built corpus and the Qwen3-14B model. Translation performance was assessed on three 50-item test sets using BLEU, ROUGE-L F1, METEOR, and BERTScore F1, together with BLEU pass-rate likelihood-ratio G^2^ tests, paired t-tests, and paired Cohen’s $d_{z}$ for item-level score differences. The results reveal a clear contrast between unseen in-domain evaluation, maximum-overlap benchmarking, and semantically related but non-fine-tuned evaluation. On Test Set A and Test Set C, neither fine-tuning strategy produced a statistically significant BLEU pass-rate advantage over the base model, and paired tests across the continuous metrics did not show consistent fine-tuning gains. On Test Set B, which was sampled from the fine-tuning corpus, both fine-tuned models substantially outperformed the base model across all four metrics, with FPFT achieving the highest scores and PEFT providing a more computationally efficient alternative. These findings indicate that textual overlap between training and deployment data, rather than broad domain similarity alone, strongly conditions the observed benefit of fine-tuning. The study offers an empirically grounded framework for selecting fine-tuning strategies in specialised machine translation.

## 1. Introduction

The application of machine translation (MT) into specialised domains remains a persistent challenge. This issue is especially pronounced for low-resource languages, which are affected by broader structural disadvantages: despite representing a large share of global linguistic diversity, they often have limited accessible text data, scarce bilingual resources, and insufficient computational infrastructure, all of which have slowed progress in machine translation for these languages [1]. Within specialised domains, this challenge manifests as a severe scarcity of high-quality, domain-aligned bilingual corpora, creating a fundamental bottleneck that directly limits translation performance [2]. To address this capability gap, fine-tuning pre-trained models on relevant, high-quality data has become a standard approach.

Previous studies have shown that fine-tuning can substantially improve the overall translation quality of large language models (LLMs) [3], particularly in handling the lexical, syntactic, and structural nuances of specialised texts [4]. In resource-constrained scenarios, the quality of fine-tuning data has been identified as more important than sheer volume [5]. Beyond general improvements, tailored fine-tuning strategies, such as two-stage algorithms that incorporate instruction-conflicting samples, have been shown to address persistent issues such as off-target translations and to improve accuracy in challenging zero-shot scenarios [6]. Empirical evidence suggests that filtering parallel sentence pairs with rule-based and scoring mechanisms before fine-tuning yields greater translation performance gains than using randomly sampled data [2].

Nevertheless, much of the empirical foundation for this work relies on publicly available parallel corpora, including OPUS-derived data used in prior fine-tuning work [3], and shared-task datasets from the Conference on Machine Translation (WMT) [6]. In domain-specific settings, OPUS-derived parallel data have also been filtered and curated for technical fields such as finance, medicine, and law [2]. These resources are primarily oriented towards news, general-domain text, or narrowly defined technical domains. This leaves a notable gap in systematic investigations based on corpora derived from formal political discourse. Consequently, our understanding of how fine-tuning strategies perform in this important real-world context remains limited.

To address this gap, this study systematically investigates the fine-tuning of large language models (LLMs) for Chinese-English political discourse translation, a domain characterized by high-stakes communicative requirements and distinctive linguistic conventions. Using a purpose-built corpus derived from national political discourse, we compare full-parameter fine-tuning (FPFT) and parameter-efficient fine-tuning (PEFT) under three evaluation conditions: unseen in-domain data, maximum-overlap data sampled from the fine-tuning corpus, and semantically related but non-fine-tuned data. Rather than treating fine-tuning performance as a single aggregate outcome, the study combines BLEU, ROUGE-L F1, METEOR, BERTScore F1, BLEU pass-rate significance testing, and paired significance and effect-size analyses across the four continuous metrics to examine when, and under what data-overlap conditions, fine-tuning is beneficial. The goal is to provide empirically grounded guidance for selecting adaptation strategies for specialised, high-impact translation tasks.

## 2. Literature review

### 2.1. Technical approaches to FPFT and PEFT

Fine-tuning approaches can broadly be classified into FPFT and PEFT. FPFT updates the entire pre-trained model, rather than adding or training only lightweight adaptation modules. It has been examined in multilingual news-analysis tasks, including genre identification, framing analysis, persuasion-technique recognition, and cross-lingual prediction settings [7]. However, this approach requires substantial computational resources and GPU memory [8]. For large language models such as Qwen3-14B, which contains approximately 14 billion parameters, full-parameter updating entails considerable computational cost and memory consumption.

This resource demand stems from the large parameter scale of modern LLMs. However, prior work suggests that, despite their large parameter counts, over-parameterized language models often exhibit low-dimensional structure during adaptation, making effective fine-tuning possible in reduced parameter subspaces or through low-rank weight updates [9,10]. This observation indicates that task-specific adaptation may not require updating every model parameter. PEFT methods build on this principle by updating only a small subset of parameters or injecting lightweight trainable modules into an otherwise frozen pre-trained model.

This approach reduces the number of trainable parameters and lowers the computational overhead of adaptation [8]. Empirical comparisons summarized in [4] show that PEFT can substantially reduce the proportion of trainable parameters while retaining competitive performance in selected benchmark settings. For example, BitFit updates only about 0.08% to 0.09% of model parameters, while Adapter and LoRA variants can operate with similarly small trainable parameter budgets, although their performance varies across tasks such as sentiment classification (SST-2) and natural language inference (MNLI). In the MNLI results reported for ${\text{RoBERTa}}_{\text{base}}$, Adapter and LoRA achieved higher accuracy than full fine-tuning despite updating only 0.5% of the parameters (94.2% and 94.2% vs. 86.4%) [4], suggesting that comparable or superior performance can sometimes be achieved with far fewer trainable parameters.

Nevertheless, performance differences between FPFT and PEFT remain context-dependent. Some studies indicate that FPFT can still outperform PEFT in specific experimental settings, although the relative advantage depends on the task, training scenario, model architecture, and language pair [7,11,12]. The primary drawback of FPFT remains its high computational and operational cost. Its substantial resource demand makes deployment difficult on hardware with constrained capacity [8]. Moreover, when model adaptation is extended to multiple language pairs or specialised domains, the associated storage and training-time costs may become prohibitively high [12]. Because each fully fine-tuned variant retains the full parameter set of the original model, scaling this approach to billion-parameter models creates major deployment hurdles [10]. This combination of model redundancy and associated cost helps explain the development and adoption of parameter-efficient alternatives.

Consequently, PEFT has emerged as an important alternative. Among various PEFT methods, low-rank adaptation (LoRA) has gained prominence because it balances efficiency and effectiveness. LoRA keeps the pre-trained parameters frozen while introducing trainable low-rank matrices into selected Transformer weight matrices. This design substantially reduces both the number of trainable parameters and the memory footprint required for fine-tuning [10]. This combination of minimal architectural modification and strong empirical performance has made LoRA an influential technique for efficient adaptation of large language models.

A limitation of standard LoRA is that it tends to distribute the update budget uniformly across selected weight matrices, without explicitly accounting for differences in their importance. To address this limitation, AdaLoRA adaptively allocates the parameter budget across weight matrices according to importance scores and dynamically adjusts the rank of incremental matrices to improve parameter efficiency and model performance [13]. This shift from static to dynamic budget allocation illustrates the ongoing refinement of PEFT methods toward greater parameter efficiency and task performance.

### 2.2. Fine-tuning of large language models for translation tasks

Recent surveys on LLM-based interactive machine translation indicate growing interest in using LLMs to support higher-quality and more efficient translation workflows, including human-in-the-loop interaction, low-resource language translation, and domain adaptation [14]. To address the computational and storage bottlenecks associated with FPFT, PEFT has emerged as an important strategy for machine translation because it aims to achieve competitive performance while updating only a small subset of parameters. Among various PEFT methods, LoRA and its variants have been reported to be effective in machine translation tasks. For example, a comparative study of fine-tuning mT0, a multilingual text-to-text language model, for Chinese-to-English translation found that LoRA, Adapter, and Prefix-Tuning enabled the model to produce English translations, with LoRA showing the strongest performance among the mT0-based PEFT methods [15]. This finding supports the relevance of LoRA as a practical PEFT strategy for translation.

Building on the LoRA framework, Language-specific fine-tuning with LoRA (LSFTL) has been proposed for low-resource machine translation [1]. Rather than updating the full model, LSFTL applies low-rank adaptation to selected Transformer components, including attention and feed-forward layers, allowing language-relevant parameters to be adjusted with limited computational overhead. In evaluations on Asian low-resource languages including Hindi, Malay, Thai, and Vietnamese, LSFTL improved COMET-based translation quality, with the clearest reported gains for the Hindi-Malay language pair, and helped smaller models achieve performance closer to that of larger systems [1]. These findings suggest that LSFTL is particularly useful when translation systems must be adapted under resource constraints.

Other PEFT methods, such as Adapters and Prefix-Tuning, have also shown value in machine translation settings. A recent systematic comparison of PEFT architectures for low-resource neural machine translation found that several PEFT methods improved both in-domain and out-of-domain translation performance, while their effectiveness varied across target languages, domains, dataset sizes, and architectural choices [16]. This variability is consistent with evidence that PEFT performance is sensitive to the proportion of parameters updated: as the number of trainable parameters decreases, performance may decline, with larger drops observed for linguistically distant language pairs [12]. Overall, these findings suggest that no single PEFT method is universally optimal; rather, LoRA, Adapters, Prefix-Tuning, and their variants may need to be selected according to the target language pair, domain, data scale, and available computational resources [12,16].

### 2.3. Challenges and specialised strategies in fine-tuning for translation

The fine-tuning process itself introduces specific challenges. A prominent issue known as the “fine-tuning paradox” arises when improvements in overall translation quality from parallel-data fine-tuning are accompanied by degradation in other desirable LLM translation abilities, such as formality control, few-shot technical-domain translation, and document-level contextualisation [3]. To address this issue, researchers have proposed mixing monolingual text with parallel fine-tuning data, a strategy shown to better preserve these abilities while further improving overall translation quality [3]. Incorporating monolingual data therefore represents an important strategy for mitigating the fine-tuning paradox and maintaining a more balanced capability profile.

For low-resource languages, the quality of training data is especially important. In domain-specific low-resource machine translation, carefully curated bilingual corpora have been shown to improve fine-tuning outcomes for Qwen and Llama models compared with randomly sampled corpora. In this approach, bilingual sentence pairs were filtered using LASER 3, a multilingual sentence-embedding model, together with rule-based scoring and domain-specific lexicons [2]. This finding further suggests that, in data-scarce environments, data quality may be as important as data volume for successful fine-tuning.

Fine-tuning strategies can also extend beyond isolated translation tasks to support integrated multilingual pipelines. For example, Translate-and-Test Transfer Learning (T3L) revisits the traditional translate-and-test approach for cross-lingual text classification by connecting a neural machine translation model with a high-resource-language classifier and enabling end-to-end fine-tuning through soft, differentiable translation representations [17]. Evidence from low-resource settings further suggests that, for some languages, improving the translator with a relatively small parallel corpus can yield larger downstream classification gains than using the same data to further train a baseline multilingual language model [17]. This suggests that fine-tuning design should be aligned with the intended deployment function rather than evaluated only as a generic adaptation procedure.

Despite this growing body of work, the comparative performance of FPFT and LoRA-based PEFT remains insufficiently examined in specialised political translation, particularly under evaluation settings that distinguish unseen in-domain performance from performance under high-overlap conditions. As a result, it remains unclear whether the observed benefits of fine-tuning reflect broader in-domain generalisation or are driven mainly by textual-overlap sensitivity. This gap motivates the present study.

## 3. Research methods

### 3.1. Corpus construction

The study employed a purpose-built Chinese-English parallel corpus compiled from officially published political materials issued between 2014 and 2024, including *Xi Jinping: The Governance of China* (Vols. 1–3), *100 Years of the Communist Party of China* (Vols. 1–2), and national institutional reports such as the annual work reports of the NPC Standing Committee and the Standing Committee of the CPPCC National Committee. These materials represent formal political and policy discourse and were selected because they contain recurrent China-specific political concepts, institutional terminology, and formulaic bilingual renderings.

After sentence-level or segment-level alignment, the corpus contained 10,453 parallel pairs, comprising 495,418 Chinese characters and 1,914,454 English words. Quality control excluded duplicate or near-duplicate entries, incomplete segments, obvious mistranslations, noisy OCR outputs, and non-parallel matches. Alibaba Cloud Bailian was used for supervised fine-tuning (SFT) and token accounting, but not for corpus construction.

For supervised fine-tuning, each pair was converted into a ChatML record comprising a system prompt, a user instruction containing the source text, and an assistant reference translation. To enable bidirectional learning, a second record was created for each pair by reversing the translation direction in the system prompt and swapping the source and target texts. This yielded 20,906 ChatML entries, which were split into a training set of 19,906 items and a validation set of 1,000 items. Token statistics are reported in Table 1.

**Table 1:** Summary statistics for the fine-tuning datasets.

Statistic	Training Dataset	Validation Dataset	Bailian Final Dataset
Total tokens	2,165,483	107,175	2,165,483
Mean	108.79	107.18	108.79
SD	48.78	45.28	48.78
Min	37	40	37
Max	454	341	454
Size	19,906	1,000	19,906

Note: The Bailian final dataset was identical to the training dataset and was used only for platform-specific export and inference configuration.

For the training dataset, the mean sequence length was 108.79 tokens (SD = 48.78), with a range of 37–454 tokens, indicating a diverse text-length distribution suitable for learning varied input structures. A sharding strategy was adopted in which each entry constituted a single training slice, mitigating the risk of excessive memory consumption per batch.

The validation set exhibited sequence-length characteristics similar to those of the training set, with a mean of 107.18 tokens (SD = 45.28) and a range of 40–341 tokens, indicating a slightly more concentrated but still representative distribution. This similarity supported reliable monitoring of model performance during fine-tuning without substantial distributional imbalance.

### 3.2. Fine-tuning procedure

Fine-tuning was conducted on the Alibaba Cloud Bailian platform using Qwen3-14B as the base model. Two supervised fine-tuning conditions were implemented: FPFT and PEFT. In the FPFT condition, all model parameters were updated, whereas in the PEFT condition, a LoRA-based adapter updated only a small subset of parameters.

Both conditions used the same training data and a three-epoch training schedule. Shared hyperparameters were kept constant when they applied to both conditions, whereas method-specific settings were configured separately. Training logs, including loss, learning rate, training speed, training-step progression, and consumed tokens, were extracted using Python scripts and visualised for comparative analysis. The hyperparameter settings for each condition are summarised in Table 2.

**Table 2:** Hyperparameter settings for the supervised fine-tuning conditions.

Method	Epochs	Learning rate	Batch size	LR scheduler type	Eval steps	Max length	Warm-up ratio	Weight decay	LoRA rank	LoRA alpha	LoRA dropout
SFT-FPFT	3	1e-5	16	linear	50	8192	0.05	0.01	NA	NA	NA
SFT-PEFT	3	3e-4	16	linear	50	8192	0.05	0.01	8	16	0.1

Note: LoRA parameters were applicable only to the PEFT condition.

### 3.3. Translation quality evaluation

Three test sets, A, B, and C, were constructed in spreadsheet format, each containing 50 items. Each item included a Prompt column specifying the translation instruction and source text, such as “Please translate into English: [Chinese source text]”, and a Completion column containing the human reference translation.

Set A contained 50 unseen in-domain items whose source texts and reference translations were absent from both the training and validation data. It was designed to assess generalisation to new material from the same broad political domain.

Set B contained 50 maximum-overlap items sampled directly from the fine-tuning corpus. This set was therefore interpreted as a diagnostic benchmark for overlap-sensitive performance rather than as a test of generalisation to unseen in-domain material.

Set C contained 50 semantically related items that were not included in fine-tuning. It belonged to the same broad political-discourse domain as Set B and was designed to test whether the gains observed under maximum textual overlap would transfer to closely related but non-overlapping cases.

The base, FPFT, and PEFT models were evaluated on all three 50-item test sets. For each test item, all three models were used to translate the source text provided in the Prompt column, and each output was compared with the corresponding reference translation. Translation quality was measured using four complementary automatic metrics: BLEU, ROUGE-L F1, METEOR, and BERTScore F1. BLEU evaluates a candidate translation by measuring clipped n-gram overlap with one or more human reference translations and applying a brevity penalty so that overly short outputs are not rewarded simply for matching a small number of words [18]. ROUGE-L F1 uses the longest common subsequence (LCS) between a candidate output and its reference to reward words that appear in the same order, and combines LCS-based recall and precision into an F1 score so that both content coverage and sentence-level ordering are reflected without requiring exact consecutive n-gram matches [19]. METEOR assesses translation quality by first mapping words in the candidate output to words in the reference through exact, morphological, and synonym-based matches, and then scoring the alignment according to content coverage, lexical precision, and the degree to which matched words remain in a coherent order [20]. BERTScore F1 evaluates translation quality by matching candidate and reference tokens through contextual embeddings rather than exact surface overlap, thereby capturing meaning-preserving paraphrases and semantic similarities that lexical metrics like BLEU often miss [21].

A threshold of BLEU ≥ 0.4 was used as an operational criterion for reporting pass rates, whereas ROUGE-L F1, METEOR, and BERTScore F1 were analysed as continuous complementary metrics to examine whether the BLEU-based pattern was supported by broader lexical, structural, and semantic evidence.

Statistical comparisons of BLEU pass-rate distributions were performed using likelihood-ratio G² tests based on 2 × 2 contingency tables. Within each test set, a separate table was constructed for each model pair: rows represented the two models, columns indicated whether translations reached the operational passing threshold (BLEU ≥ 0.4), and each model contributed 50 items. Following Agresti’s notation [22], the likelihood-ratio statistic was defined formally as shown in Eq 1.


$$\begin{array}{c} G^{\text{2}}=\text{2}\;\sum_{i=\text{1}}^{\text{2}}\sum_{j=\text{1}}^{\text{2}}n_{ij}\text{log}\left(\frac{n_{ij}}{{\hat{\mu }}_{ij}}\right) \end{array}$$ (1)

where $n_{ij}$ denotes the observed cell count and ${\hat{\mu }}_{ij}$ denotes the expected cell count under the null hypothesis of equal pass/fail distributions. Larger $G^{\text{2}}$ values indicate greater divergence between model pass/fail distributions, and p-values were evaluated using the chi-square distribution with one degree of freedom.

For the four continuous item-level metrics, model pairs were additionally compared using two-sided paired t-tests and paired Cohen’s $d_{z}$. For each model pair, the paired t-test was calculated from the item-level score differences across the same 50 test items and tested whether the mean difference differed significantly from zero. Consistent with Cohen’s treatment of paired observations as a one-sample analysis of difference scores [23], paired Cohen’s $d_{z}$ was calculated from the item-level difference scores as shown in Eq 2.


$$\begin{array}{c} d_{z}=\frac{\overset{―}{Z}}{{\text{S}}_{Z}} \end{array}$$ (2)

where Z denotes the paired score difference, $\overset{―}{Z}$ is the mean paired difference, and ${\text{S}}_{Z}$ is the sample standard deviation of the paired differences. Because each paired t-test compared item-level metric scores across the same 50 test items, the degrees of freedom were 49 for each test set. Because Set B intentionally contained exact overlap with the fine-tuning corpus, claims about generalisation are based primarily on Sets A and C, whereas Set B is used to estimate the upper-bound effect of textual overlap.

A step-by-step protocol documenting corpus preparation, ChatML conversion, FPFT/PEFT configuration, training-log extraction, figure generation, three-test-set construction, and multi-metric translation-quality evaluation is available at protocols.io: https://dx.doi.org/10.17504/protocols.io.kqdg3re7pg25/v1.

## 4. Results

This study compared FPFT and PEFT by fine-tuning the Qwen3-14B model for Chinese-English political discourse translation. The results revealed distinct patterns across five key aspects: loss convergence, computational efficiency, learning-rate schedules, token consumption, and translation performance.

### 4.1. Loss convergence patterns of FPFT and PEFT

**Fig 1:**
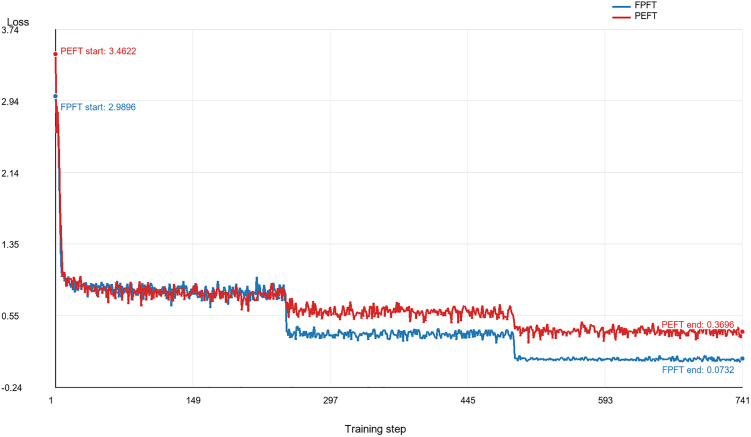
Training loss trajectories of FPFT and PEFT across training steps.

Fig 1 shows training loss against training steps. The first logged loss was 2.9896 for FPFT and 3.4622 for PEFT. During the early phase, both methods reduced loss rapidly, with FPFT showing a slightly sharper initial descent: FPFT dropped below 1.0 at logged step 8, whereas PEFT first dropped below 1.0 at logged step 9. Thereafter, both trajectories entered a lower-loss regime within the first few dozen logged steps. The figure labels the initial and final logged loss values for each method, allowing direct comparison of the early-stage decline and final convergence levels.

During the early-to-middle phase of training, after the initial rapid decline, FPFT exhibited a more volatile trajectory than PEFT. Within the first 40 logged steps, FPFT decreased from 2.9896 to a minimum of approximately 0.7568, but this descent was not strictly monotonic: after first falling below 1.0 at step 8, it briefly rebounded above 1.0 at steps 9–10 before continuing downward. This pattern suggests that full-parameter updates may have made the optimization path more sensitive to local variation in the training data. Because FPFT updates the entire parameter space, the model can adapt rapidly but is also more vulnerable to unstable parameter movement and local overreaction to transient noise. By contrast, PEFT followed a more gradual and constrained downward trajectory. Because LoRA updates only a limited set of low-rank matrices rather than the full model, the optimization process is more constrained and regularized, which may help suppress large oscillations and produce a more controlled descent.

In the later phase of training, the FPFT oscillation amplitude diminished substantially. From logged step 415 onward, the FPFT loss remained below 0.4, and from step 600 to the end of training it fluctuated within a low range of approximately 0.0314–0.1084. It reached a final logged loss of 0.0732 at the last recorded step. PEFT, meanwhile, maintained a smoother and more gradual downward trajectory throughout the middle-to-late stages. From logged step 235 onward, its loss remained below 0.9; from step 375 to the end of training, it ranged from approximately 0.2406 to 0.6764. However, under the present training configuration, this smoother descent did not translate into the lowest final loss, and PEFT reached 0.3696 at the final logged step.

These final values, taken directly from the last entries of the two loss logs, quantify a clear divergence in optimization behavior: FPFT converged more rapidly and ultimately reached a substantially lower final loss, whereas PEFT converged more gradually and more smoothly but plateaued at a higher loss level. This contrast suggests two distinct optimization profiles under the same training schedule. Because FPFT updates the entire parameter space, it can adapt more aggressively and reduce loss faster, but it may also be more susceptible to unstable parameter movement and temporary overreaction to local noise in the training data. PEFT, implemented through low-rank adaptation, constrains updates to a smaller parameter subspace and therefore follows a more regularized and controlled trajectory. In the present experiment, the more constrained PEFT path was associated with greater optimization stability, whereas FPFT achieved the stronger end-of-training fit.

### 4.2. Training throughput and computational efficiency

**Fig 2:**
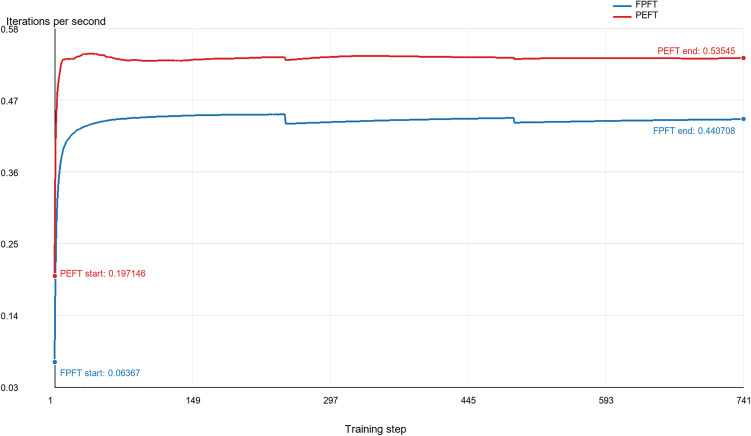
Training throughput of FPFT and PEFT across training steps.

Fig 2 shows a clear and persistent throughput advantage for PEFT when speed is measured in iterations per second (iter/s) across training steps. Throughout the training process, PEFT consistently ran faster than FPFT, indicating a substantial computational-efficiency advantage under the same training data and three-epoch schedule. This separation remained visible across the full training run and is quantified in the step-level comparisons below.

At the first logged step, FPFT began at 0.06367 iter/s, whereas PEFT started substantially higher at 0.197146 iter/s. The separation became visible almost immediately during the warm-up stage. By step 3, PEFT had already reached 0.447376 iter/s, while FPFT was still at 0.272274 iter/s. By step 10, PEFT had risen to 0.532992 iter/s, compared with 0.389233 iter/s for FPFT. By step 20, the two methods had largely entered their operational range, but PEFT still maintained a clear lead (0.534339 vs. 0.417053 iter/s).

As training continued, the gap remained stable rather than transient. FPFT generally plateaued in the range of approximately 0.42–0.45 iter/s, whereas PEFT remained near 0.53–0.54 iter/s across the middle and late stages of training. At the final logged step, FPFT reached 0.440708 iter/s and PEFT reached 0.53545 iter/s. Using the late-stage values, PEFT maintained a throughput advantage of approximately 21%−22% over FPFT. This difference is operationally meaningful because, under the same training schedule, PEFT could either complete the same training run in less time or execute more optimization steps within the same wall-clock budget.

### 4.3. Learning rate schedules under FPFT and PEFT

**Fig 3:**
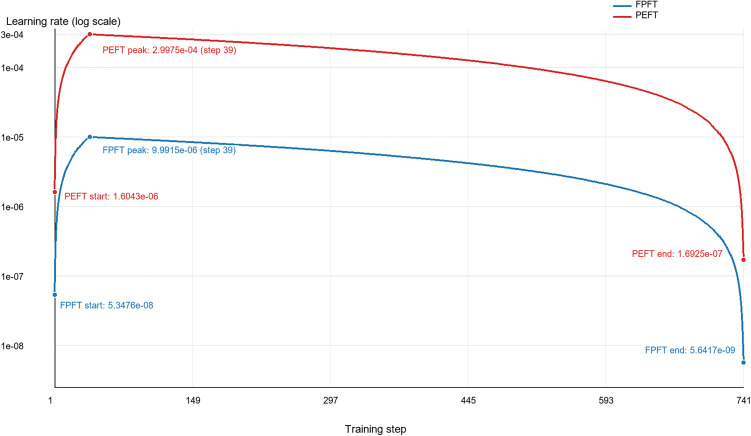
Learning-rate schedules of FPFT and PEFT across training steps on a logarithmic scale.

Fig 3 shows the learning-rate schedules of FPFT and PEFT across training steps on a logarithmic scale. The logarithmic y-axis allows the lower-magnitude FPFT schedule and the higher-magnitude PEFT schedule to be displayed within the same figure. The values reported below are the original learning-rate values extracted from the training logs.

The two methods followed markedly different learning-rate schedules despite being trained on the same data. FPFT began at 5.3476×${\text{1}\text{0}}^{-\text{8}}$, increased gradually during warm-up, reached a peak of approximately 9.9915×${\text{1}\text{0}}^{-\text{6}}$ at step 39, and then decayed steadily throughout the remainder of training, ending at 5.6417×${\text{1}\text{0}}^{-\text{9}}$. PEFT, by contrast, started at a much higher value of 1.6043×${\text{1}\text{0}}^{-\text{6}}$, rose rapidly to a peak of approximately 2.9975×${\text{1}\text{0}}^{-\text{4}}$ also at step 39, and then followed a higher-magnitude decay trajectory before reaching 1.6925×${\text{1}\text{0}}^{-\text{7}}$ at the final logged step.

The early-stage contrast was substantial. By step 10, FPFT had reached 2.4064×${\text{1}\text{0}}^{-\text{6}}$, whereas PEFT had already risen to 7.2193×${\text{1}\text{0}}^{-\text{5}}$. By step 20, the values were 5.0802×${\text{1}\text{0}}^{-\text{6}}$ for FPFT and 1.5241×${\text{1}\text{0}}^{-\text{4}}$ for PEFT; by step 30, they had increased further to 7.7540×${\text{1}\text{0}}^{-\text{6}}$ and 2.3262×${\text{1}\text{0}}^{-\text{4}}$, respectively. Thus, although both methods warmed up over a similar number of steps, PEFT operated at a substantially higher absolute learning-rate scale from the beginning.

This difference remained pronounced through the middle phase of training. At step 200, FPFT used a learning rate of 7.7066×${\text{1}\text{0}}^{-\text{6}}$, whereas PEFT used 2.3120×${\text{1}\text{0}}^{-\text{4}}$; at step 300, the corresponding values were 6.2821×${\text{1}\text{0}}^{-\text{6}}$ and 1.8846×${\text{1}\text{0}}^{-\text{4}}$. In other words, PEFT operated at approximately 30 times the FPFT learning rate across the stable middle portion of training. This divergence reflects the different learning-rate settings used for the two methods: because FPFT updated the full parameter space, it was trained under a more conservative learning-rate schedule, whereas PEFT, operating in a smaller low-rank adaptation space, used a substantially larger step size throughout training.

### 4.4. Token consumption under equivalent training schedules

**Fig 4:**
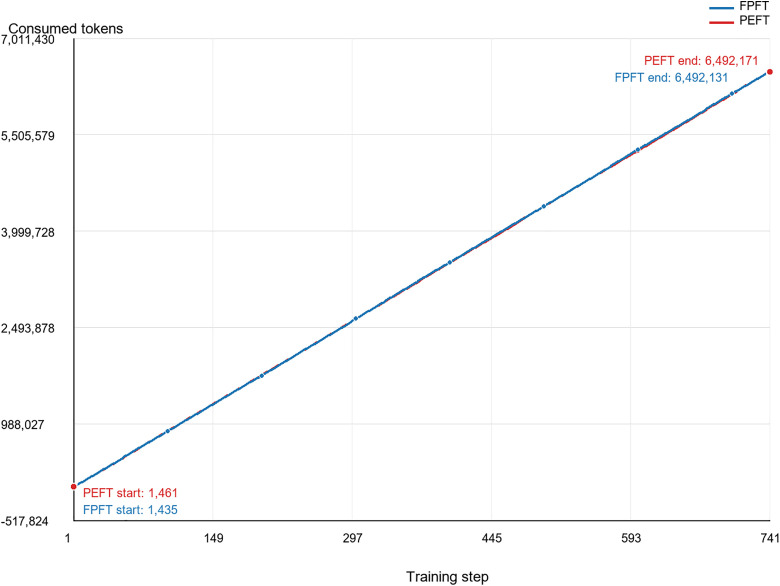
Cumulative consumed tokens of FPFT and PEFT across training steps.

Fig 4 shows cumulative consumed tokens across training steps. The two trajectories increase almost linearly and remain very close throughout the full logged training run, indicating that FPFT and PEFT processed essentially the same token budget under the same training data and three-epoch schedule. The near-overlapping trajectories are consistent with the experimental design, in which both methods used the same training data and three-epoch schedule. Accordingly, PEFT’s efficiency advantage is best interpreted as computational rather than token-based.

The token logs confirm this visual impression. FPFT increased from 1,435 tokens at the first logged step to 6,492,131 tokens at the final logged step, whereas PEFT increased from 1,461 tokens to 6,492,171 tokens. The final difference was therefore 40 tokens, corresponding to approximately 0.0006% of the total training budget. Across the 741 logged steps, the stepwise difference in cumulative consumed tokens (PEFT minus FPFT) ranged from −19,223 to +7,104 tokens, indicating that the cumulative token count was slightly higher for PEFT at some checkpoints and slightly higher for FPFT at others. These fluctuations did not accumulate into a meaningful final difference and instead reflect minor implementation-level variation rather than a systematic difference in data exposure.

Both final totals also remained very close to the theoretical expectation of 6,496,449 tokens, based on 2,165,483 tokens per epoch over three epochs. The observed totals were lower by 4,318 tokens for FPFT and 4,278 tokens for PEFT, again suggesting negligible boundary effects during batching or token accounting. Fig 4 therefore shows that token consumption was governed almost entirely by dataset size and the prescribed number of epochs, rather than by the choice of fine-tuning method. Together with the throughput results in Section 4.2, this indicates that PEFT’s practical efficiency advantage was computational rather than token-based.

### 4.5. Performance across unseen, maximum-overlap, and semantically related test sets

**Fig 5:**
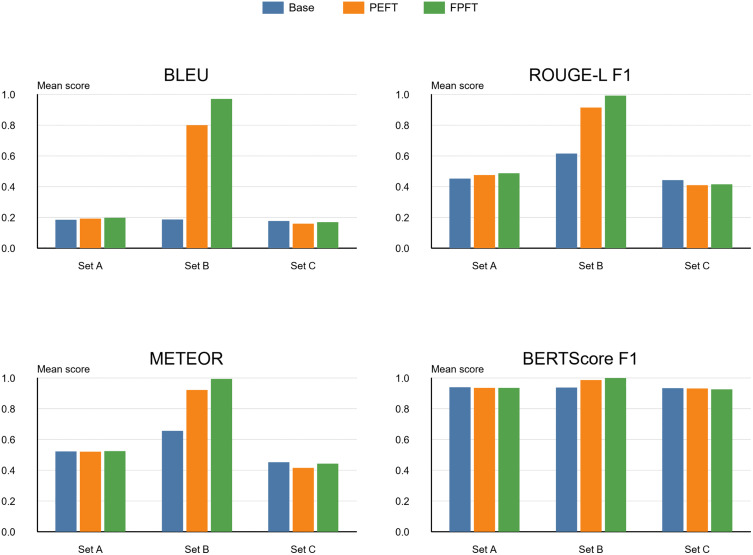
Mean translation-quality scores across models and test sets.

Translation quality was compared across the three test sets using BLEU, ROUGE-L F1, METEOR, and BERTScore F1 (Table 3; Fig 5). Test Set A contained unseen in-domain items, Test Set B was a maximum-overlap benchmark sampled from the fine-tuning corpus, and Test Set C was semantically related to Set B but was not used for fine-tuning. Fig 5 compares the four evaluation metrics for the base, PEFT, and FPFT models across Test Sets A, B, and C. Together, Table 3 and Fig 5 show that the largest fine-tuning gains occurred on the maximum-overlap Set B, whereas semantically related but non-fine-tuned Set C did not show comparable gains.

**Table 3:** Multi-metric translation-quality summary across test sets.

Test set	Model	Mean BLEU	BLEU pass count/rate	Mean ROUGE-L F1	Mean METEOR	Mean BERTScore F1
Set A	Base	0.1817	6/50 (12%)	0.4505	0.5193	0.9371
Set A	PEFT	0.1889	2/50 (4%)	0.4736	0.5169	0.9330
Set A	FPFT	0.1944	6/50 (12%)	0.4854	0.5219	0.9324
Set B	Base	0.1834	6/50 (12%)	0.6124	0.6543	0.9355
Set B	PEFT	0.7977	42/50 (84%)	0.9124	0.9191	0.9829
Set B	FPFT	0.9687	49/50 (98%)	0.9894	0.9920	0.9980
Set C	Base	0.1734	6/50 (12%)	0.4397	0.4504	0.9302
Set C	PEFT	0.1555	4/50 (8%)	0.4069	0.4117	0.9283
Set C	FPFT	0.1661	4/50 (8%)	0.4122	0.4389	0.9239

Note: BLEU pass count refers to the number of translations with BLEU ≥ 0.4 out of 50 items. ROUGE-L F1, METEOR, and BERTScore F1 are reported as complementary reference-based automatic metrics. Full item-level scores and standard deviations are provided in S2 Dataset.

On Test Set A, all three models produced low BLEU scores and none of the fine-tuned models achieved a statistically significant BLEU pass-rate advantage over the base model. Mean BLEU scores were 0.1817 for the base model, 0.1889 for PEFT, and 0.1944 for FPFT; BLEU pass rates were 12%, 4%, and 12%, respectively. The same pattern was visible in the complementary metrics: ROUGE-L F1 was 0.4505, 0.4736, and 0.4854; METEOR was 0.5193, 0.5169, and 0.5219; and BERTScore F1 was 0.9371, 0.9330, and 0.9324 for the base, PEFT, and FPFT models, respectively. BLEU pass-rate likelihood-ratio G^2^ tests confirmed no significant differences between any model pair on Set A (all p > 0.05; Table 4).

**Table 4:** BLEU pass-rate likelihood-ratio G^2^ test comparisons.

Test set	Comparison	BLEU pass counts	G^2^	p	Sig.
Set A	Base vs. PEFT	6 vs. 2	2.27	0.132	n.s.
Set A	Base vs. FPFT	6 vs. 6	0.00	1.000	n.s.
Set A	PEFT vs. FPFT	2 vs. 6	2.27	0.132	n.s.
Set B	Base vs. PEFT	6 vs. 42	57.81	<0.001	***
Set B	Base vs. FPFT	6 vs. 49	91.13	<0.001	***
Set B	PEFT vs. FPFT	42 vs. 49	6.74	0.009	**
Set C	Base vs. PEFT	6 vs. 4	0.45	0.504	n.s.
Set C	Base vs. FPFT	6 vs. 4	0.45	0.504	n.s.
Set C	PEFT vs. FPFT	4 vs. 4	0.00	1.000	n.s.

Note: G^2^ values compare BLEU pass/fail distributions between model pairs; n.s. = not significant; ** = p < 0.01; *** = p < 0.001.

For Set A, paired t-tests for the four continuous metrics were non-significant across all model pairs (Table 5). For example, Base vs. PEFT comparisons yielded BLEU t(49) = −0.34, p = 0.738, $d_{z}$ = −0.05; ROUGE-L F1 t(49) = −1.09, p = 0.281, $d_{z}$ = −0.15; METEOR t(49) = 0.10, p = 0.918, $d_{z}$ = 0.01; and BERTScore F1 t(49) = 1.32, p = 0.193, $d_{z}$ = 0.19. The corresponding Base vs. FPFT tests were also non-significant: BLEU t(49) = −0.42, p = 0.675, $d_{z}$ = −0.06; ROUGE-L F1 t(49) = −1.20, p = 0.235, $d_{z}$ = −0.17; METEOR t(49) = −0.09, p = 0.928, $d_{z}$ = −0.01; and BERTScore F1 t(49) = 1.25, p = 0.219, $d_{z}$ = 0.18. These results support the conclusion that the observed Set A differences were small and non-systematic rather than reliable fine-tuning gains.

**Table 5:** Paired t-test results for item-level translation-quality metrics.

Set	Metric	Comparison	Mean difference	t(49)	p	Cohen’s $d_{z}$
Set A	BLEU	Base vs. PEFT	−0.0072	−0.34	0.738	−0.05
Set A	BLEU	Base vs. FPFT	−0.0127	−0.42	0.675	−0.06
Set A	BLEU	PEFT vs. FPFT	−0.0055	−0.29	0.769	−0.04
Set A	ROUGE-L F1	Base vs. PEFT	−0.0232	−1.09	0.281	−0.15
Set A	ROUGE-L F1	Base vs. FPFT	−0.0349	−1.20	0.235	−0.17
Set A	ROUGE-L F1	PEFT vs. FPFT	−0.0118	−0.53	0.598	−0.08
Set A	METEOR	Base vs. PEFT	0.0025	0.10	0.918	0.01
Set A	METEOR	Base vs. FPFT	−0.0026	−0.09	0.928	−0.01
Set A	METEOR	PEFT vs. FPFT	−0.0050	−0.19	0.852	−0.03
Set A	BERTScore F1	Base vs. PEFT	0.0041	1.32	0.193	0.19
Set A	BERTScore F1	Base vs. FPFT	0.0047	1.25	0.219	0.18
Set A	BERTScore F1	PEFT vs. FPFT	0.0006	0.23	0.821	0.03
Set B	BLEU	Base vs. PEFT	−0.6143	−12.28	<0.001	−1.74
Set B	BLEU	Base vs. FPFT	−0.7853	−25.52	<0.001	−3.61
Set B	BLEU	PEFT vs. FPFT	−0.1710	−4.13	<0.001	−0.58
Set B	ROUGE-L F1	Base vs. PEFT	−0.3000	−9.22	<0.001	−1.30
Set B	ROUGE-L F1	Base vs. FPFT	−0.3770	−12.90	<0.001	−1.82
Set B	ROUGE-L F1	PEFT vs. FPFT	−0.0770	−3.38	0.001	−0.48
Set B	METEOR	Base vs. PEFT	−0.2647	−8.03	<0.001	−1.14
Set B	METEOR	Base vs. FPFT	−0.3376	−11.24	<0.001	−1.59
Set B	METEOR	PEFT vs. FPFT	−0.0729	−3.10	0.003	−0.44
Set B	BERTScore F1	Base vs. PEFT	−0.0474	−8.69	<0.001	−1.23
Set B	BERTScore F1	Base vs. FPFT	−0.0625	−13.03	<0.001	−1.84
Set B	BERTScore F1	PEFT vs. FPFT	−0.0151	−3.33	0.002	−0.47
Set C	BLEU	Base vs. PEFT	0.0179	0.71	0.482	0.10
Set C	BLEU	Base vs. FPFT	0.0073	0.35	0.729	0.05
Set C	BLEU	PEFT vs. FPFT	−0.0106	−0.64	0.527	−0.09
Set C	ROUGE-L F1	Base vs. PEFT	0.0328	1.29	0.203	0.18
Set C	ROUGE-L F1	Base vs. FPFT	0.0275	1.12	0.266	0.16
Set C	ROUGE-L F1	PEFT vs. FPFT	−0.0054	−0.33	0.746	−0.05
Set C	METEOR	Base vs. PEFT	0.0387	1.44	0.158	0.20
Set C	METEOR	Base vs. FPFT	0.0115	0.46	0.646	0.07
Set C	METEOR	PEFT vs. FPFT	−0.0272	−1.66	0.103	−0.24
Set C	BERTScore F1	Base vs. PEFT	0.0019	0.56	0.579	0.08
Set C	BERTScore F1	Base vs. FPFT	0.0063	1.74	0.088	0.25
Set C	BERTScore F1	PEFT vs. FPFT	0.0044	1.65	0.106	0.23

Note: Mean difference is calculated as the first model minus the second model in each comparison. Negative values indicate that the second model scored higher. Paired tests used the same 50 items within each test set. Full precision values are provided in S2 Dataset.

On Test Set B, both fine-tuned models showed large gains over the base model across all four metrics. The base model achieved a mean BLEU of 0.1834 and a BLEU pass rate of 12%, whereas PEFT achieved a mean BLEU of 0.7977 with an 84% pass rate and FPFT achieved a mean BLEU of 0.9687 with a 98% pass rate. The additional metrics supported the same conclusion: mean ROUGE-L F1 increased from 0.6124 for the base model to 0.9124 for PEFT and 0.9894 for FPFT; METEOR increased from 0.6543 to 0.9191 and 0.9920; and BERTScore F1 increased from 0.9355 to 0.9829 and 0.9980. Likelihood-ratio G^2^ tests showed significant BLEU pass-rate differences for Base vs. PEFT (G^2^ = 57.81, p < 0.001), Base vs. FPFT (G^2^ = 91.13, p < 0.001), and PEFT vs. FPFT (G^2^ = 6.74, p = 0.009). The paired BLEU-score effect sizes also favoured the fine-tuned models, especially FPFT ($d_{z}$ = −1.74 for Base vs. PEFT, $d_{z}$ = −3.61 for Base vs. FPFT, and $d_{z}$ = −0.58 for PEFT vs. FPFT).

For Set B, paired t-tests confirmed significant fine-tuning advantages across all four continuous metrics. Base vs. PEFT comparisons were significant for BLEU, t(49) = −12.28, p < 0.001, $d_{z}$ = −1.74; ROUGE-L F1, t(49) = −9.22, p < 0.001, $d_{z}$ = −1.30; METEOR, t(49) = −8.03, p < 0.001, $d_{z}$ = −1.14; and BERTScore F1, t(49) = −8.69, p < 0.001, $d_{z}$ = −1.23. Base vs. FPFT comparisons showed even larger effects: BLEU t(49) = −25.52, p < 0.001, $d_{z}$ = −3.61; ROUGE-L F1 t(49) = −12.90, p < 0.001, $d_{z}$ = −1.82; METEOR t(49) = −11.24, p < 0.001, $d_{z}$ = −1.59; and BERTScore F1 t(49) = −13.03, p < 0.001, $d_{z}$ = −1.84. FPFT also significantly outperformed PEFT on BLEU, t(49) = −4.13, p < 0.001, $d_{z}$ = −0.58; ROUGE-L F1, t(49) = −3.38, p = 0.001, $d_{z}$ = −0.48; METEOR, t(49) = −3.10, p = 0.003, $d_{z}$ = −0.44; and BERTScore F1, t(49) = −3.33, p = 0.002, $d_{z}$ = −0.47.

On Test Set C, which was semantically related to Set B but excluded from fine-tuning, the Set B gains did not transfer. Mean BLEU scores were 0.1734 for the base model, 0.1555 for PEFT, and 0.1661 for FPFT; BLEU pass rates were 12%, 8%, and 8%, respectively. ROUGE-L F1 was 0.4397, 0.4069, and 0.4122, while METEOR was 0.4504, 0.4117, and 0.4389. BERTScore F1 was 0.9302 for the base model, 0.9283 for PEFT, and 0.9239 for FPFT. Likelihood-ratio G² tests showed no significant BLEU pass-rate differences between any model pair on Set C (all p > 0.05). These results indicate that semantic relatedness to the fine-tuning domain was not sufficient to reproduce the high-overlap gains observed on Set B.

For Set C, paired t-tests were non-significant across all four continuous metrics. Base vs. PEFT results were BLEU t(49) = 0.71, p = 0.482, $d_{z}$ = 0.10; ROUGE-L F1 t(49) = 1.29, p = 0.203, $d_{z}$ = 0.18; METEOR t(49) = 1.44, p = 0.158, $d_{z}$ = 0.20; and BERTScore F1 t(49) = 0.56, p = 0.579, $d_{z}$ = 0.08. Base vs. FPFT comparisons were also non-significant for BLEU, t(49) = 0.35, p = 0.729, $d_{z}$ = 0.05; ROUGE-L F1, t(49) = 1.12, p = 0.266, $d_{z}$ = 0.16; METEOR, t(49) = 0.46, p = 0.646, $d_{z}$ = 0.07; and BERTScore F1, t(49) = 1.74, p = 0.088, $d_{z}$ = 0.25. PEFT vs. FPFT was likewise non-significant across all four metrics, including BERTScore F1, t(49) = 1.65, p = 0.106, $d_{z}$ = 0.23. This reinforces the interpretation that FPFT’s high matched-data performance did not generalise reliably to semantically related but non-overlapping inputs.

## 5. Discussion

This section interprets the empirical findings by focusing on the relationship between textual overlap, semantic relatedness, fine-tuning behaviour, and deployment-oriented model selection. Rather than treating FPFT or PEFT as universally superior, the discussion considers why their effectiveness varied across evaluation conditions and what this variation implies for specialised Chinese-English political discourse translation.

### 5.1. Textual overlap and the boundaries of generalisation

The contrast among Test Sets A, B, and C is best understood as a contrast among unseen in-domain evaluation, maximum-overlap evaluation, and semantically related but non-fine-tuned evaluation. When applied to unseen in-domain texts in Test Set A, neither FPFT nor PEFT produced a statistically significant BLEU pass-rate advantage over the base model, and paired t-tests on the continuous metrics were also non-significant. Test Set C further sharpened this interpretation: although it was semantically related to Set B and belonged to the same broad political domain, neither fine-tuned model showed a BLEU pass-rate advantage over the base model, and paired tests across BLEU, ROUGE-L F1, METEOR, and BERTScore F1 were non-significant. This suggests that the specialised knowledge acquired from a finite political discourse corpus remained narrow and instance-sensitive rather than broadly transferable to new but semantically related texts.

This interpretation is consistent with prior machine translation studies showing that the benefits of fine-tuning are conditional rather than uniform: fine-tuning can improve translation quality but may also affect broader LLM capabilities [3], and the effectiveness of parameter-efficient transfer depends on factors such as the adaptation method, backbone model, language pair, parameter budget, and training-data size [12]. Related evidence from limited-data LLM fine-tuning further suggests that specialised adaptation can be effective with small training subsets, although the magnitude and scope of these gains vary across tasks and datasets [24].

By contrast, Test Set B was intentionally sampled from the fine-tuning corpus in order to estimate the upper-bound effect of textual overlap. Under this maximum-overlap condition, both fine-tuned models showed substantial descriptive gains, statistically significant BLEU pass-rate advantages over the base model, and significant paired-test differences across BLEU, ROUGE-L F1, METEOR, and BERTScore F1. The paired-test effects were especially large for Base vs. FPFT on BLEU, t(49) = −25.52, p < 0.001, $d_{z}$ = −3.61, and remained large for ROUGE-L F1, t(49) = −12.90, p < 0.001, $d_{z}$ = −1.82; METEOR, t(49) = −11.24, p < 0.001, $d_{z}$ = −1.59; and BERTScore F1, t(49) = −13.03, p < 0.001, $d_{z}$ = −1.84. PEFT also significantly exceeded the base model across all four metrics, with Base vs. PEFT $d_{z}$ values ranging from −1.14 to −1.74. These findings show that fine-tuning can be exceptionally powerful when the target text distribution is stable and closely matched with the adaptation corpus, such as recurrent administrative formulae, standardised reports, or templated diplomatic communication. However, the Set C results show that semantic proximity to the fine-tuning domain alone was insufficient to reproduce the Set B gains when direct textual overlap was removed.

### 5.2. Strategic implications for fine-tuning method selection

The training dynamics reported in Sections 4.1 through 4.4 help explain the downstream evaluation outcomes. Because FPFT updates the full parameter space of the model, it reduced loss more aggressively during training and achieved the lowest final logged training loss. At the same time, this broader update space was associated with a more volatile middle-phase optimization trajectory. In practical terms, full-parameter adaptation appears to perturb the pre-trained weight space more strongly, which can improve corpus-specific fitting when the training and application materials are closely aligned, but may reduce the extent to which broader linguistic priors are preserved. This interpretation is consistent with the multi-metric results: FPFT performed strongest on maximum-overlap Set B, including a very large paired BLEU difference relative to the base model, t(49) = −25.52, p < 0.001, $d_{z}$ = −3.61, but it did not produce a statistically significant BLEU pass-rate advantage on unseen Set A or semantically related unseen Set C. In addition, paired comparisons on Set C were non-significant across the continuous metrics, including ROUGE-L F1, METEOR, and BERTScore F1. The non-significant BERTScore F1 results are especially relevant here because they indicate that stronger corpus-specific fitting did not translate into a reliable semantic-similarity advantage on non-overlapping but related material.

PEFT, by contrast, updates only a low-rank subset of parameters and therefore follows a more constrained optimization path. In the present study, this mechanism was associated with smoother convergence, approximately 21%−22% higher training throughput, and essentially identical token consumption relative to FPFT under the same three-epoch schedule. On maximum-overlap Test Set B, PEFT produced substantial gains over the base model across BLEU, ROUGE-L F1, METEOR, and BERTScore F1, with paired t-tests confirming significant Base vs. PEFT differences for all four metrics (all p < 0.001; $d_{z}$ = −1.74, −1.30, −1.14, and −1.23, respectively). However, PEFT did not yield a significant BLEU pass-rate advantage on Sets A or C, and the paired t-tests on Set C were non-significant across all four metrics for Base vs. PEFT. This indicates that PEFT’s computational efficiency and matched-data gains did not automatically translate into stronger generalisation when textual overlap was absent.

In this respect, our findings also speak to the broader PEFT literature, which characterises PEFT as a way to adapt pre-trained models to downstream tasks while substantially reducing trainable parameters, memory use, and computational costs [8,10]. PEFT’s constrained update mechanism was associated with a smoother and more stable optimization process, but under the present conditions it did not match FPFT’s mean BLEU score or pass rate on maximum-overlap data. This trade-off is consistent with prior comparisons showing that parameter-efficient methods can reduce trainable parameters and training time in broader NLP settings [7,11] and can improve machine translation performance under low-resource conditions [1,12,16], but they do not uniformly dominate full-parameter fine-tuning across tasks, data sizes, and model settings [11,12].

Taken together, these findings indicate that fine-tuning method choice should be understood as a deployment-sensitive decision rather than as a search for a universally superior method. In other words, the relative value of FPFT, PEFT, and the base model depends on the interaction among textual overlap, formulaic regularity in the target materials, adaptation-specific training dynamics such as loss stability and convergence behaviour, and practical resource constraints.

### 5.3. Strategic framework for deployment: Balancing quality, efficiency, and robustness

Building on the preceding discussion of evaluation outcomes and training dynamics, this section translates the findings into a practical deployment framework for translation applications. This deployment-oriented framing is consistent with recent work on sustainable machine translation, which argues that model adaptation should weigh the expected benefits of fine-tuning against the associated time, cost, and energy requirements [25].

Under high-overlap conditions in which sufficient computational resources are available, FPFT may be the preferred strategy because it delivers the highest upper-bound performance on matched materials. In the present study, this was most clearly reflected in Test Set B, where FPFT achieved the highest mean scores for BLEU (0.9687), ROUGE-L F1 (0.9894), METEOR (0.9920), and BERTScore F1 (0.9980), with a 98% BLEU pass rate.

Under high-overlap but resource-constrained conditions, PEFT may be the more pragmatic option. Although it did not match FPFT’s peak performance on Test Set B, it still produced substantial gains over the base model across all four metrics, including a statistically significant BLEU pass-rate improvement (mean BLEU 0.7977; pass rate 84%), while maintaining a clear computational advantage. In particular, PEFT achieved higher training throughput than FPFT while processing essentially the same token budget, making it a practical option for rapid prototyping, cost-sensitive deployment, and scenarios requiring multiple specialised models.

Under low-overlap, semantically related but non-overlapping, or otherwise unpredictable conditions, the base model may remain the safest operational baseline. On Test Sets A and C, neither FPFT nor PEFT yielded a statistically significant BLEU pass-rate advantage over the base model. In such scenarios, deploying a highly specialised fine-tuned model may provide little additional value relative to the broader robustness retained by the base model.

### 5.4. Limitations and future research

This study has several limitations that define the scope of the present findings and point to future work. The modelling evidence is based on a single base model (Qwen3-14B), which limits the extent to which the observed pattern can be assumed to hold across other large language model families. The PEFT comparison is also restricted to LoRA; alternative parameter-efficient strategies, including Adapters and Prefix-Tuning, were not evaluated. The data design further involved one training scale and three 50-item evaluation sets, leaving the quantitative relationships among overlap ratio, semantic relatedness, data scale, and fine-tuning benefit to be examined more systematically. In addition, although the evaluation combines BLEU, ROUGE-L F1, METEOR, and BERTScore F1, these metrics are all automatic, reference-based measures and cannot fully replace expert human assessment of adequacy, style, and political-discourse nuance.

Future research should therefore test whether the present framework generalises across additional base models, compare multiple PEFT methods within the same political translation setting, vary training-data scale and overlap ratio explicitly, and combine automatic metrics with human assessment. This mixed-evaluation direction is supported by recent LLM-based translation research that combines automatic metrics, error counts, qualitative examples, and expert ratings to provide a more comprehensive assessment of translation system performance [26]. Future evaluation could further incorporate fine-grained error analysis, including error-span detection and severity assessment, as well as LLM-supported quality estimation procedures. Recent work on machine translation quality estimation suggests that such fine-grained approaches can provide more precise and diagnostic evidence about translation errors than aggregate metrics alone [27]. These directions would help clarify the scope and the practical transferability of the overlap-sensitive effects identified here.

## 6. Conclusion

This study provides an empirically grounded comparison of FPFT and PEFT for adapting a large language model to specialised Chinese-English political discourse translation. Using the Qwen3-14B model, the results show that fine-tuning benefits became evident primarily under conditions of direct textual overlap between the fine-tuning corpus and the evaluation data, whereas domain alignment or semantic similarity alone did not yield statistically significant improvements in translation quality.

Under unseen in-domain and semantically related but non-overlapping conditions, neither FPFT nor PEFT produced a statistically significant BLEU pass-rate advantage over the base model. This suggests that fine-tuning on a finite specialised corpus did not automatically yield broader transferable gains across new texts from the same domain or from semantically related contexts. By contrast, on the maximum-overlap benchmark, both fine-tuned models improved markedly over the base model across BLEU, ROUGE-L F1, METEOR, and BERTScore F1; FPFT achieved the highest scores, while PEFT also delivered substantial gains. These results indicate that the strongest effects of fine-tuning in the present study should be interpreted as overlap-sensitive specialisation performance rather than as evidence of robust unseen in-domain generalisation.

The practical implication is therefore conditional rather than absolute. When the target text distribution is highly stable and closely matched with the adaptation corpus, FPFT may be preferable for achieving peak specialised performance. When overlap remains high but computational efficiency is a stronger priority, PEFT offers a practical alternative. When deployment inputs are diverse, weakly matched, or difficult to anticipate, retaining the base model may be the more reliable choice. In this sense, model selection is best treated as a deployment decision guided by expected training-application overlap, resource constraints, and the need for robustness under non-overlapping or unpredictable inputs.

By combining BLEU pass-rate likelihood-ratio G^2^ testing with paired t-tests and paired effect-size interpretation across continuous metric scores, the study shows that fine-tuning strategies should be assessed not only by whether they improve aggregate metric scores, but also by how reliably those gains transfer under different overlap levels and operational constraints. More broadly, the findings suggest that effective specialisation depends on carefully balancing the learning of domain-specific patterns against the preservation of the base model’s broader linguistic priors. This perspective may inform future work on efficient and reliable adaptation strategies for specialised translation and related language tasks.

## Supporting information

S1 File Description of the shared data materials, including corpus sources, test-set definitions, and field definitions. (DOCX)

S2 DatasetEvaluation files for Test Sets A, B, and C; item-level BLEU, ROUGE-L F1, METEOR, and BERTScore F1 scores; aggregated multi-metric comparison tables; BLEU pass-rate likelihood-ratio G^2^ tests; paired t-tests; and paired Cohen’s $d_{z}$ calculations.(XLSX)

S3 File Python scripts, FPFT/PEFT training logs, and item-level metric data used to generate Figs 1–5 and the reported tables and source data. (ZIP)

## Abbreviations

BERTScore F1: F1 score of the BERT-based semantic similarity metric
BLEU: Bilingual Evaluation Understudy
ChatML: Chat Markup Language
COMET: Crosslingual Optimized Metric for Evaluation of Translation
FPFT: Full-parameter fine-tuning
iter/s: iterations per second
LLM: Large language model
LoRA: Low-rank adaptation
LR: Learning rate
LSFTL: Language-specific fine-tuning with LoRA
METEOR: Metric for Evaluation of Translation with Explicit ORdering
MT: Machine translation
PEFT: Parameter-efficient fine-tuning
ROUGE-L F1: F1 score of the Recall-Oriented Understudy for Gisting Evaluation based on the longest common subsequence
SFT: Supervised fine-tuning
T3L: Translate-and-Test Transfer Learning
