# Supplementary material for: Textual overlap rather than domain alignment: A comparative study of fine-tuning strategies for specialised machine translation with large language models
Source: PLoS One. 2026 Jul 20;21(7):e0352256. doi: 10.1371/journal.pone.0352256 (PMC13384323; doi:10.1371/journal.pone.0352256)
Supplement: S1 File — (DOCX) [file pone.0352256.s001.docx]

**S1 File. Description of the shared data materials**

This supporting file provides a description of the data materials and metadata used in the paper entitled “Textual Overlap Rather Than Domain Alignment: A Comparative Study of Fine-Tuning Strategies for Specialised Machine Translation with Large Language Models.” It is intended to help readers, editors, and reviewers inspect the structure, provenance, field definitions, and evaluation use of the shared materials.

**Corpus-source metadata**

The bilingual corpus was compiled from officially published Chinese-English political materials issued from 2014 to 2024. The sources included Xi Jinping: The Governance of China (Vols. 1-3), 100 Years of the Communist Party of China (Vols. 1-2), and annual institutional reports, including the work reports of the Standing Committee of the National People’s Congress and the Standing Committee of the National Committee of the Chinese People’s Political Consultative Conference.

**Corpus construction and screening**

The corpus contained 10,453 aligned Chinese-English translation pairs before the Chinese-to-English and English-to-Chinese bidirectional augmentation step. Entries were retained only when sentence- or segment-level alignment was sufficiently clear for supervised fine-tuning. Duplicate or near-duplicate entries, incomplete records, evidently mistranslated pairs, severely noisy OCR lines, and non-parallel pairs were excluded during quality control.

**ChatML conversion**

For supervised fine-tuning, each bilingual pair was converted into a ChatML record consisting of a system prompt, a user instruction, and an assistant response containing the reference translation. Bidirectional training entries were created by reversing the translation direction in the system prompt and swapping the source and target texts. This process yielded 20,906 ChatML entries in total.

**Dataset splits**

After bidirectional augmentation, the dataset was split into a training set (19,906 items; 2,165,483 tokens) and a validation set (1,000 items; 107,175 tokens). The Bailian final dataset recorded for the deployed fine-tuned models was identical to the training set (19,906 items; 2,165,483 tokens).

**Test-set descriptions**

Test Set A contains 50 unseen in-domain items whose source texts and reference translations were excluded from both the training and validation data. It is used to assess generalisation to new material from the same broad political-discourse domain.

Test Set B contains 50 maximum-overlap items sampled directly from the fine-tuning corpus. It is interpreted as a diagnostic benchmark for overlap-sensitive performance rather than as a conventional unseen in-domain test set.

Test Set C contains 50 political-discourse items that are semantically related to Set B but were not included in the fine-tuning corpus. It was added to examine whether gains observed on the maximum-overlap Set B transfer to related but non-overlapping material from the same broad domain.

**Evaluation workbook fields**

In the English-language evaluation files, Item_ID denotes the paired item identifier used to match the same underlying source item across Base, PEFT, and FPFT outputs within each test set. Reference denotes the human English reference translation, and Translation denotes the model-generated English translation. Model identifies whether the output was produced by the base model, PEFT model, or FPFT model, and Set identifies whether the item belongs to Test Set A, B, or C.

The accompanying S2 Dataset contains test-set metadata, Item_ID-based item-level BLEU, ROUGE-L F1, METEOR, and BERTScore F1 scores, aggregated multi-metric summaries, BLEU pass-rate likelihood-ratio G^2^ tests, and paired t-tests with paired Cohen's $d_{z}$ effect-size calculations corresponding to the results reported in the manuscript.

**Evaluation statistics**

BLEU was used both as an item-level translation-quality metric and as the basis for the operational pass threshold, with BLEU ≥ 0.4 used for reporting pass rates. ROUGE-L F1, METEOR, and BERTScore F1 were used as complementary automatic reference-based metrics to evaluate lexical and sequence overlap, unigram alignment with fragmentation, and contextual semantic similarity.

BLEU pass-rate distributions were compared using likelihood-ratio G^2^ tests for 2 × 2 pass/fail contingency tables. For each model pair within each test set, the two rows represented the two models and the two columns represented translations that did or did not reach BLEU ≥ 0.4. The resulting p values were evaluated using the chi-square distribution with one degree of freedom.

For the four continuous item-level metrics, model pairs were compared using two-sided paired t-tests across the same 50 test items within each test set; therefore, the degrees of freedom were 49 for each test set. Paired Cohen’s $d_{z}$ was calculated from the item-level difference scores as the mean paired difference divided by the sample standard deviation of the paired differences.

**Metric software configuration**

The item-level metrics were computed using Python 3.11 with sacrebleu 2.6.0 for sentence-level BLEU scaled to 0-1, a word-level longest-common-subsequence implementation for ROUGE-L F1, NLTK 3.9.1 meteor_score for METEOR, and the official bert-score 0.3.12 package for BERTScore F1. BERTScore F1 was computed using roberta-large, the recommended English layer 17, and the PyTorch/Transformers backend. Because the roberta-large model files are not included in S3 due to file-size constraints, users can reproduce the calculation by using the Hugging Face model identifier roberta-large or by specifying a local roberta-large directory with the --bertscore-model command-line option.

**S3 code package**

The S3 File contains Python scripts, FPFT/PEFT training logs, Item_ID-based item-level metric data, generated TIFF figure files, and CSV table-data files used to reproduce Figs 1-5 and the reported Table 3-5 source data. The intermediate source translation workbooks used during local metric calculation are not redistributed.

**Reproducibility note**

Because some underlying source texts and published translations may be subject to copyright restrictions, the full bilingual corpus is not redistributed with this paper. Instead, this supporting file summarizes corpus provenance, dataset structure, field definitions, and evaluation use to support transparency and reproducibility.
