# Supplementary material for: Textual overlap rather than domain alignment: A comparative study of fine-tuning strategies for specialised machine translation with large language models
Source: PLoS One. 2026 Jul 20;21(7):e0352256. doi: 10.1371/journal.pone.0352256 (PMC13384323; doi:10.1371/journal.pone.0352256)
Supplement: S3 File — (ZIP) [file pone.0352256.s003.zip › fpft_log.docx]

1

2025-10-03 16:12:56,096 - INFO - data process succeeded, start to fine-tune

2

2025-10-03 16:16:02,534 - INFO - data process succeeded, start to fine-tune

3

Fine-tune started

4

dataset info: {'train_dataset': {'total': 2165483, 'mean': 108.7854415754044, 'std': 48.783743636290694, 'min': 37, 'max': 454, 'size': 19906}, 'val_dataset': {'total': 107175, 'mean': 107.175, 'std': 45.28253940538229, 'min': 40, 'max': 341, 'size': 1000}, 'final_dataset': {'total': 2165483, 'mean': 108.7854415754044, 'std': 48.783743636290694, 'min': 37, 'max': 454, 'size': 19906}, 'total_token': 2165483}

5

{'loss': 2.9896466732025146, 'learning_rate': 5.3475935828877005e-08, 'train_speed(iter/s)': 0.06367, 'epoch': 0.0008038585209003215, 'consumed_train_tokens': 1435}

6

{'loss': 2.7668280601501465, 'learning_rate': 2.6737967914438503e-07, 'train_speed(iter/s)': 0.201998, 'epoch': 0.0040192926045016075, 'consumed_train_tokens': 8434}

7

{'loss': 2.6931806564331056, 'learning_rate': 5.347593582887701e-07, 'train_speed(iter/s)': 0.272274, 'epoch': 0.008038585209003215, 'consumed_train_tokens': 17258}

8

{'loss': 2.5549026489257813, 'learning_rate': 8.021390374331551e-07, 'train_speed(iter/s)': 0.312475, 'epoch': 0.012057877813504822, 'consumed_train_tokens': 25437}

9

{'loss': 2.0645523071289062, 'learning_rate': 1.0695187165775401e-06, 'train_speed(iter/s)': 0.335489, 'epoch': 0.01607717041800643, 'consumed_train_tokens': 33826}

10

{'loss': 1.5643875122070312, 'learning_rate': 1.3368983957219254e-06, 'train_speed(iter/s)': 0.351699, 'epoch': 0.02009646302250804, 'consumed_train_tokens': 41440}

11

{'loss': 1.1227645874023438, 'learning_rate': 1.6042780748663103e-06, 'train_speed(iter/s)': 0.36646, 'epoch': 0.024115755627009645, 'consumed_train_tokens': 50150}

12

{'loss': 0.9826555252075195, 'learning_rate': 1.8716577540106954e-06, 'train_speed(iter/s)': 0.375402, 'epoch': 0.028135048231511254, 'consumed_train_tokens': 59258}

13

{'loss': 1.0138092041015625, 'learning_rate': 2.1390374331550802e-06, 'train_speed(iter/s)': 0.383835, 'epoch': 0.03215434083601286, 'consumed_train_tokens': 68443}

14

{'loss': 1.0118820190429687, 'learning_rate': 2.4064171122994653e-06, 'train_speed(iter/s)': 0.389233, 'epoch': 0.03617363344051447, 'consumed_train_tokens': 77008}

15

{'loss': 0.9333718299865723, 'learning_rate': 2.673796791443851e-06, 'train_speed(iter/s)': 0.395565, 'epoch': 0.04019292604501608, 'consumed_train_tokens': 85030}

16

{'loss': 0.9656410217285156, 'learning_rate': 2.9411764705882355e-06, 'train_speed(iter/s)': 0.399059, 'epoch': 0.04421221864951769, 'consumed_train_tokens': 93522}

17

{'loss': 0.9377279281616211, 'learning_rate': 3.2085561497326205e-06, 'train_speed(iter/s)': 0.402188, 'epoch': 0.04823151125401929, 'consumed_train_tokens': 101553}

18

{'loss': 0.8797224044799805, 'learning_rate': 3.4759358288770056e-06, 'train_speed(iter/s)': 0.406432, 'epoch': 0.0522508038585209, 'consumed_train_tokens': 110705}

19

{'loss': 0.9831997871398925, 'learning_rate': 3.7433155080213907e-06, 'train_speed(iter/s)': 0.407945, 'epoch': 0.05627009646302251, 'consumed_train_tokens': 118977}

20

{'loss': 0.9176892280578614, 'learning_rate': 4.010695187165775e-06, 'train_speed(iter/s)': 0.410665, 'epoch': 0.06028938906752412, 'consumed_train_tokens': 128461}

21

{'loss': 0.8874513626098632, 'learning_rate': 4.2780748663101604e-06, 'train_speed(iter/s)': 0.411657, 'epoch': 0.06430868167202572, 'consumed_train_tokens': 136789}

22

{'loss': 0.8665586471557617, 'learning_rate': 4.5454545454545455e-06, 'train_speed(iter/s)': 0.414423, 'epoch': 0.06832797427652733, 'consumed_train_tokens': 145136}

23

{'loss': 0.9137736320495605, 'learning_rate': 4.812834224598931e-06, 'train_speed(iter/s)': 0.415586, 'epoch': 0.07234726688102894, 'consumed_train_tokens': 153383}

24

{'loss': 0.8364657402038574, 'learning_rate': 5.0802139037433165e-06, 'train_speed(iter/s)': 0.417053, 'epoch': 0.07636655948553055, 'consumed_train_tokens': 161719}

25

{'loss': 0.9720272064208985, 'learning_rate': 5.347593582887702e-06, 'train_speed(iter/s)': 0.418513, 'epoch': 0.08038585209003216, 'consumed_train_tokens': 170577}

26

{'loss': 0.8112249374389648, 'learning_rate': 5.614973262032086e-06, 'train_speed(iter/s)': 0.419403, 'epoch': 0.08440514469453377, 'consumed_train_tokens': 179005}

27

{'loss': 0.8260951995849609, 'learning_rate': 5.882352941176471e-06, 'train_speed(iter/s)': 0.421085, 'epoch': 0.08842443729903537, 'consumed_train_tokens': 187554}

28

{'loss': 0.8451935768127441, 'learning_rate': 6.149732620320856e-06, 'train_speed(iter/s)': 0.421902, 'epoch': 0.09244372990353698, 'consumed_train_tokens': 196189}

29

{'loss': 0.845604133605957, 'learning_rate': 6.417112299465241e-06, 'train_speed(iter/s)': 0.423527, 'epoch': 0.09646302250803858, 'consumed_train_tokens': 204420}

30

{'loss': 0.8326454162597656, 'learning_rate': 6.684491978609626e-06, 'train_speed(iter/s)': 0.424082, 'epoch': 0.10048231511254019, 'consumed_train_tokens': 213166}

31

{'loss': 0.8806550979614258, 'learning_rate': 6.951871657754011e-06, 'train_speed(iter/s)': 0.425027, 'epoch': 0.1045016077170418, 'consumed_train_tokens': 222020}

32

{'loss': 0.8867603302001953, 'learning_rate': 7.219251336898396e-06, 'train_speed(iter/s)': 0.425775, 'epoch': 0.1085209003215434, 'consumed_train_tokens': 230504}

33

{'loss': 0.8470218658447266, 'learning_rate': 7.486631016042781e-06, 'train_speed(iter/s)': 0.426283, 'epoch': 0.11254019292604502, 'consumed_train_tokens': 239479}

34

{'loss': 0.8979010581970215, 'learning_rate': 7.754010695187166e-06, 'train_speed(iter/s)': 0.427392, 'epoch': 0.11655948553054662, 'consumed_train_tokens': 247783}

35

{'loss': 0.8837850570678711, 'learning_rate': 8.02139037433155e-06, 'train_speed(iter/s)': 0.427799, 'epoch': 0.12057877813504823, 'consumed_train_tokens': 256826}

36

{'loss': 0.8815362930297852, 'learning_rate': 8.288770053475937e-06, 'train_speed(iter/s)': 0.428888, 'epoch': 0.12459807073954984, 'consumed_train_tokens': 266055}

37

{'loss': 0.8148338317871093, 'learning_rate': 8.556149732620321e-06, 'train_speed(iter/s)': 0.429244, 'epoch': 0.12861736334405144, 'consumed_train_tokens': 274877}

38

{'loss': 0.9205718040466309, 'learning_rate': 8.823529411764707e-06, 'train_speed(iter/s)': 0.429898, 'epoch': 0.13263665594855306, 'consumed_train_tokens': 284092}

39

{'loss': 0.8678748130798339, 'learning_rate': 9.090909090909091e-06, 'train_speed(iter/s)': 0.43029, 'epoch': 0.13665594855305466, 'consumed_train_tokens': 292378}

40

{'loss': 0.7568210601806641, 'learning_rate': 9.358288770053477e-06, 'train_speed(iter/s)': 0.430609, 'epoch': 0.14067524115755628, 'consumed_train_tokens': 300823}

41

{'loss': 0.8764461517333985, 'learning_rate': 9.625668449197861e-06, 'train_speed(iter/s)': 0.431466, 'epoch': 0.14469453376205788, 'consumed_train_tokens': 309094}

42

{'loss': 0.849027156829834, 'learning_rate': 9.893048128342247e-06, 'train_speed(iter/s)': 0.431827, 'epoch': 0.1487138263665595, 'consumed_train_tokens': 317582}

43

{'loss': 0.8337915420532227, 'learning_rate': 9.991537376586742e-06, 'train_speed(iter/s)': 0.432726, 'epoch': 0.1527331189710611, 'consumed_train_tokens': 326547}

44

{'loss': 0.9105710029602051, 'learning_rate': 9.977433004231313e-06, 'train_speed(iter/s)': 0.432605, 'epoch': 0.1567524115755627, 'consumed_train_tokens': 334883}

45

{'loss': 0.8176158905029297, 'learning_rate': 9.963328631875882e-06, 'train_speed(iter/s)': 0.433391, 'epoch': 0.1607717041800643, 'consumed_train_tokens': 343742}

46

{'loss': 0.9006740570068359, 'learning_rate': 9.949224259520453e-06, 'train_speed(iter/s)': 0.433703, 'epoch': 0.1647909967845659, 'consumed_train_tokens': 352128}

47

{'loss': 0.8758627891540527, 'learning_rate': 9.935119887165022e-06, 'train_speed(iter/s)': 0.434147, 'epoch': 0.16881028938906753, 'consumed_train_tokens': 360450}

48

{'loss': 0.8331684112548828, 'learning_rate': 9.921015514809593e-06, 'train_speed(iter/s)': 0.434386, 'epoch': 0.17282958199356913, 'consumed_train_tokens': 368674}

49

{'loss': 0.9100008010864258, 'learning_rate': 9.906911142454162e-06, 'train_speed(iter/s)': 0.434654, 'epoch': 0.17684887459807075, 'consumed_train_tokens': 376832}

50

{'loss': 0.7729828357696533, 'learning_rate': 9.892806770098731e-06, 'train_speed(iter/s)': 0.435241, 'epoch': 0.18086816720257234, 'consumed_train_tokens': 385996}

51

{'loss': 0.8875587463378907, 'learning_rate': 9.878702397743302e-06, 'train_speed(iter/s)': 0.435392, 'epoch': 0.18488745980707397, 'consumed_train_tokens': 394540}

52

{'loss': 0.8787792205810547, 'learning_rate': 9.864598025387871e-06, 'train_speed(iter/s)': 0.436045, 'epoch': 0.18890675241157556, 'consumed_train_tokens': 403548}

53

{'loss': 0.8363935470581054, 'learning_rate': 9.850493653032442e-06, 'train_speed(iter/s)': 0.436081, 'epoch': 0.19292604501607716, 'consumed_train_tokens': 411819}

54

{'loss': 0.8452218055725098, 'learning_rate': 9.836389280677011e-06, 'train_speed(iter/s)': 0.436513, 'epoch': 0.19694533762057878, 'consumed_train_tokens': 419673}

55

{'loss': 0.9227931022644043, 'learning_rate': 9.822284908321582e-06, 'train_speed(iter/s)': 0.436926, 'epoch': 0.20096463022508038, 'consumed_train_tokens': 428284}

56

{'loss': 0.8790755271911621, 'learning_rate': 9.808180535966151e-06, 'train_speed(iter/s)': 0.437092, 'epoch': 0.204983922829582, 'consumed_train_tokens': 437471}

57

{'loss': 0.9018000602722168, 'learning_rate': 9.79407616361072e-06, 'train_speed(iter/s)': 0.437583, 'epoch': 0.2090032154340836, 'consumed_train_tokens': 446614}

58

{'loss': 0.8312362670898438, 'learning_rate': 9.77997179125529e-06, 'train_speed(iter/s)': 0.437705, 'epoch': 0.21302250803858522, 'consumed_train_tokens': 455153}

59

{'loss': 0.8742752075195312, 'learning_rate': 9.751763046544429e-06, 'train_speed(iter/s)': 0.43827, 'epoch': 0.22106109324758844, 'consumed_train_tokens': 473583}

60

{'loss': 0.8363051414489746, 'learning_rate': 9.737658674188998e-06, 'train_speed(iter/s)': 0.438236, 'epoch': 0.22508038585209003, 'consumed_train_tokens': 482733}

61

{'loss': 0.9085205078125, 'learning_rate': 9.723554301833569e-06, 'train_speed(iter/s)': 0.438569, 'epoch': 0.22909967845659163, 'consumed_train_tokens': 491295}

62

{'loss': 0.8760858535766601, 'learning_rate': 9.709449929478138e-06, 'train_speed(iter/s)': 0.43878, 'epoch': 0.23311897106109325, 'consumed_train_tokens': 499738}

63

{'loss': 0.8319509506225586, 'learning_rate': 9.695345557122709e-06, 'train_speed(iter/s)': 0.439173, 'epoch': 0.23713826366559485, 'consumed_train_tokens': 508175}

64

{'loss': 0.8962970733642578, 'learning_rate': 9.681241184767278e-06, 'train_speed(iter/s)': 0.43928, 'epoch': 0.24115755627009647, 'consumed_train_tokens': 517387}

65

{'loss': 0.8459666252136231, 'learning_rate': 9.667136812411849e-06, 'train_speed(iter/s)': 0.439673, 'epoch': 0.24517684887459806, 'consumed_train_tokens': 526101}

66

{'loss': 0.9003363609313965, 'learning_rate': 9.653032440056418e-06, 'train_speed(iter/s)': 0.439718, 'epoch': 0.2491961414790997, 'consumed_train_tokens': 534433}

67

{'loss': 0.7846570491790772, 'learning_rate': 9.638928067700987e-06, 'train_speed(iter/s)': 0.439931, 'epoch': 0.2532154340836013, 'consumed_train_tokens': 543070}

68

{'loss': 0.8437472343444824, 'learning_rate': 9.624823695345558e-06, 'train_speed(iter/s)': 0.440145, 'epoch': 0.2572347266881029, 'consumed_train_tokens': 552120}

69

{'loss': 0.7980759620666504, 'learning_rate': 9.610719322990127e-06, 'train_speed(iter/s)': 0.440303, 'epoch': 0.2612540192926045, 'consumed_train_tokens': 561154}

70

{'loss': 0.8408727645874023, 'learning_rate': 9.596614950634698e-06, 'train_speed(iter/s)': 0.440556, 'epoch': 0.2652733118971061, 'consumed_train_tokens': 569924}

71

{'loss': 0.8068592071533203, 'learning_rate': 9.582510578279267e-06, 'train_speed(iter/s)': 0.44054, 'epoch': 0.2692926045016077, 'consumed_train_tokens': 578762}

72

{'loss': 0.9342202186584473, 'learning_rate': 9.568406205923838e-06, 'train_speed(iter/s)': 0.440821, 'epoch': 0.2733118971061093, 'consumed_train_tokens': 588190}

73

{'loss': 0.7570268630981445, 'learning_rate': 9.554301833568407e-06, 'train_speed(iter/s)': 0.440813, 'epoch': 0.27733118971061094, 'consumed_train_tokens': 596605}

74

{'loss': 0.7720812797546387, 'learning_rate': 9.540197461212976e-06, 'train_speed(iter/s)': 0.440947, 'epoch': 0.28135048231511256, 'consumed_train_tokens': 605245}

75

{'loss': 0.9133710861206055, 'learning_rate': 9.526093088857547e-06, 'train_speed(iter/s)': 0.441136, 'epoch': 0.2853697749196141, 'consumed_train_tokens': 614272}

76

{'loss': 0.7560438632965087, 'learning_rate': 9.511988716502116e-06, 'train_speed(iter/s)': 0.441283, 'epoch': 0.28938906752411575, 'consumed_train_tokens': 621998}

77

{'loss': 0.8795669555664063, 'learning_rate': 9.497884344146687e-06, 'train_speed(iter/s)': 0.441384, 'epoch': 0.2934083601286174, 'consumed_train_tokens': 630554}

78

{'loss': 0.8330381393432618, 'learning_rate': 9.483779971791256e-06, 'train_speed(iter/s)': 0.44133, 'epoch': 0.297427652733119, 'consumed_train_tokens': 638817}

79

{'loss': 0.803952980041504, 'learning_rate': 9.469675599435827e-06, 'train_speed(iter/s)': 0.441533, 'epoch': 0.30144694533762056, 'consumed_train_tokens': 647003}

80

{'loss': 0.8080390930175781, 'learning_rate': 9.455571227080396e-06, 'train_speed(iter/s)': 0.441579, 'epoch': 0.3054662379421222, 'consumed_train_tokens': 655266}

81

{'loss': 0.8482237815856933, 'learning_rate': 9.441466854724965e-06, 'train_speed(iter/s)': 0.441885, 'epoch': 0.3094855305466238, 'consumed_train_tokens': 663865}

82

{'loss': 0.8421257019042969, 'learning_rate': 9.427362482369536e-06, 'train_speed(iter/s)': 0.441838, 'epoch': 0.3135048231511254, 'consumed_train_tokens': 672598}

83

{'loss': 0.9029973983764649, 'learning_rate': 9.413258110014105e-06, 'train_speed(iter/s)': 0.441902, 'epoch': 0.317524115755627, 'consumed_train_tokens': 681185}

84

{'loss': 0.7975649356842041, 'learning_rate': 9.399153737658676e-06, 'train_speed(iter/s)': 0.442116, 'epoch': 0.3215434083601286, 'consumed_train_tokens': 689603}

85

{'loss': 0.8651233673095703, 'learning_rate': 9.385049365303245e-06, 'train_speed(iter/s)': 0.442078, 'epoch': 0.32556270096463025, 'consumed_train_tokens': 698635}

86

{'loss': 0.8037633895874023, 'learning_rate': 9.370944992947814e-06, 'train_speed(iter/s)': 0.44237, 'epoch': 0.3295819935691318, 'consumed_train_tokens': 707139}

87

{'loss': 0.905362892150879, 'learning_rate': 9.356840620592385e-06, 'train_speed(iter/s)': 0.442436, 'epoch': 0.33360128617363344, 'consumed_train_tokens': 715438}

88

{'loss': 0.7205351829528809, 'learning_rate': 9.342736248236954e-06, 'train_speed(iter/s)': 0.442736, 'epoch': 0.33762057877813506, 'consumed_train_tokens': 723931}

89

{'loss': 0.8497194290161133, 'learning_rate': 9.328631875881525e-06, 'train_speed(iter/s)': 0.442694, 'epoch': 0.34163987138263663, 'consumed_train_tokens': 732458}

90

{'loss': 0.8039112091064453, 'learning_rate': 9.314527503526094e-06, 'train_speed(iter/s)': 0.442749, 'epoch': 0.34565916398713825, 'consumed_train_tokens': 740936}

91

{'loss': 0.8286870956420899, 'learning_rate': 9.300423131170665e-06, 'train_speed(iter/s)': 0.44284, 'epoch': 0.3496784565916399, 'consumed_train_tokens': 749931}

92

{'loss': 0.7761863708496094, 'learning_rate': 9.286318758815234e-06, 'train_speed(iter/s)': 0.442898, 'epoch': 0.3536977491961415, 'consumed_train_tokens': 758393}

93

{'loss': 0.8031447410583497, 'learning_rate': 9.272214386459803e-06, 'train_speed(iter/s)': 0.443029, 'epoch': 0.35771704180064307, 'consumed_train_tokens': 766342}

94

{'loss': 0.7733681678771973, 'learning_rate': 9.258110014104374e-06, 'train_speed(iter/s)': 0.442955, 'epoch': 0.3617363344051447, 'consumed_train_tokens': 774790}

95

{'loss': 0.8082757949829101, 'learning_rate': 9.244005641748943e-06, 'train_speed(iter/s)': 0.443214, 'epoch': 0.3657556270096463, 'consumed_train_tokens': 783874}

96

{'loss': 0.8139203071594239, 'learning_rate': 9.229901269393513e-06, 'train_speed(iter/s)': 0.44319, 'epoch': 0.36977491961414793, 'consumed_train_tokens': 792556}

97

{'loss': 0.7993620872497559, 'learning_rate': 9.215796897038083e-06, 'train_speed(iter/s)': 0.443284, 'epoch': 0.3737942122186495, 'consumed_train_tokens': 801400}

98

{'loss': 0.8485307693481445, 'learning_rate': 9.201692524682653e-06, 'train_speed(iter/s)': 0.443406, 'epoch': 0.3778135048231511, 'consumed_train_tokens': 809290}

99

{'loss': 0.9119300842285156, 'learning_rate': 9.187588152327223e-06, 'train_speed(iter/s)': 0.443514, 'epoch': 0.38183279742765275, 'consumed_train_tokens': 818090}

100

{'loss': 0.7293354511260987, 'learning_rate': 9.173483779971792e-06, 'train_speed(iter/s)': 0.44366, 'epoch': 0.3858520900321543, 'consumed_train_tokens': 826469}

101

{'loss': 0.8964365005493165, 'learning_rate': 9.159379407616362e-06, 'train_speed(iter/s)': 0.443658, 'epoch': 0.38987138263665594, 'consumed_train_tokens': 834916}

102

{'loss': 0.8617910385131836, 'learning_rate': 9.145275035260932e-06, 'train_speed(iter/s)': 0.443918, 'epoch': 0.39389067524115756, 'consumed_train_tokens': 844746}

103

{'loss': 0.7918103218078614, 'learning_rate': 9.131170662905502e-06, 'train_speed(iter/s)': 0.443867, 'epoch': 0.3979099678456592, 'consumed_train_tokens': 853027}

104

{'loss': 0.8552597045898438, 'learning_rate': 9.117066290550071e-06, 'train_speed(iter/s)': 0.44397, 'epoch': 0.40192926045016075, 'consumed_train_tokens': 861284}

105

{'loss': 0.8084210395812989, 'learning_rate': 9.102961918194642e-06, 'train_speed(iter/s)': 0.444058, 'epoch': 0.4059485530546624, 'consumed_train_tokens': 869826}

106

{'loss': 0.8032593727111816, 'learning_rate': 9.088857545839211e-06, 'train_speed(iter/s)': 0.444126, 'epoch': 0.409967845659164, 'consumed_train_tokens': 878195}

107

{'loss': 0.8455934524536133, 'learning_rate': 9.07475317348378e-06, 'train_speed(iter/s)': 0.444285, 'epoch': 0.4139871382636656, 'consumed_train_tokens': 887610}

108

{'loss': 0.9147495269775391, 'learning_rate': 9.06064880112835e-06, 'train_speed(iter/s)': 0.444237, 'epoch': 0.4180064308681672, 'consumed_train_tokens': 896240}

109

{'loss': 0.887578010559082, 'learning_rate': 9.04654442877292e-06, 'train_speed(iter/s)': 0.444457, 'epoch': 0.4220257234726688, 'consumed_train_tokens': 905201}

110

{'loss': 0.8475646018981934, 'learning_rate': 9.03244005641749e-06, 'train_speed(iter/s)': 0.44439, 'epoch': 0.42604501607717044, 'consumed_train_tokens': 913778}

111

{'loss': 0.7385244369506836, 'learning_rate': 9.018335684062059e-06, 'train_speed(iter/s)': 0.444493, 'epoch': 0.430064308681672, 'consumed_train_tokens': 921704}

112

{'loss': 0.8634418487548828, 'learning_rate': 9.00423131170663e-06, 'train_speed(iter/s)': 0.444615, 'epoch': 0.4340836012861736, 'consumed_train_tokens': 930417}

113

{'loss': 0.8512213706970215, 'learning_rate': 8.990126939351199e-06, 'train_speed(iter/s)': 0.444657, 'epoch': 0.43810289389067525, 'consumed_train_tokens': 939643}

114

{'loss': 0.7973270416259766, 'learning_rate': 8.97602256699577e-06, 'train_speed(iter/s)': 0.444707, 'epoch': 0.44212218649517687, 'consumed_train_tokens': 948897}

115

{'loss': 0.7980223655700683, 'learning_rate': 8.961918194640339e-06, 'train_speed(iter/s)': 0.444796, 'epoch': 0.44614147909967844, 'consumed_train_tokens': 957648}

116

{'loss': 0.8225831031799317, 'learning_rate': 8.94781382228491e-06, 'train_speed(iter/s)': 0.444854, 'epoch': 0.45016077170418006, 'consumed_train_tokens': 966121}

117

{'loss': 0.7755729675292968, 'learning_rate': 8.933709449929478e-06, 'train_speed(iter/s)': 0.444817, 'epoch': 0.4541800643086817, 'consumed_train_tokens': 974478}

118

{'loss': 0.8246108055114746, 'learning_rate': 8.919605077574048e-06, 'train_speed(iter/s)': 0.444916, 'epoch': 0.45819935691318325, 'consumed_train_tokens': 983471}

119

{'loss': 0.7780660629272461, 'learning_rate': 8.905500705218618e-06, 'train_speed(iter/s)': 0.444883, 'epoch': 0.4622186495176849, 'consumed_train_tokens': 993019}

120

{'loss': 0.816097640991211, 'learning_rate': 8.891396332863187e-06, 'train_speed(iter/s)': 0.444967, 'epoch': 0.4662379421221865, 'consumed_train_tokens': 1002046}

121

{'loss': 0.8772773742675781, 'learning_rate': 8.877291960507758e-06, 'train_speed(iter/s)': 0.445047, 'epoch': 0.4702572347266881, 'consumed_train_tokens': 1010965}

122

{'loss': 0.788902473449707, 'learning_rate': 8.863187588152327e-06, 'train_speed(iter/s)': 0.445143, 'epoch': 0.4742765273311897, 'consumed_train_tokens': 1019470}

123

{'loss': 0.860240364074707, 'learning_rate': 8.849083215796898e-06, 'train_speed(iter/s)': 0.445208, 'epoch': 0.4782958199356913, 'consumed_train_tokens': 1028105}

124

{'loss': 0.8470748901367188, 'learning_rate': 8.834978843441467e-06, 'train_speed(iter/s)': 0.445138, 'epoch': 0.48231511254019294, 'consumed_train_tokens': 1038170}

125

{'loss': 0.7347418308258057, 'learning_rate': 8.820874471086036e-06, 'train_speed(iter/s)': 0.445321, 'epoch': 0.48633440514469456, 'consumed_train_tokens': 1046552}

126

{'loss': 0.7767784118652343, 'learning_rate': 8.806770098730607e-06, 'train_speed(iter/s)': 0.445272, 'epoch': 0.4903536977491961, 'consumed_train_tokens': 1055010}

127

{'loss': 0.7497474670410156, 'learning_rate': 8.792665726375176e-06, 'train_speed(iter/s)': 0.445374, 'epoch': 0.49437299035369775, 'consumed_train_tokens': 1063550}

128

{'loss': 0.9039283752441406, 'learning_rate': 8.778561354019747e-06, 'train_speed(iter/s)': 0.445456, 'epoch': 0.4983922829581994, 'consumed_train_tokens': 1073713}

129

{'loss': 0.8420183181762695, 'learning_rate': 8.764456981664316e-06, 'train_speed(iter/s)': 0.445487, 'epoch': 0.502411575562701, 'consumed_train_tokens': 1081181}

130

{'loss': 0.6535048484802246, 'learning_rate': 8.750352609308887e-06, 'train_speed(iter/s)': 0.445507, 'epoch': 0.5064308681672026, 'consumed_train_tokens': 1089753}

131

{'loss': 0.8454002380371094, 'learning_rate': 8.736248236953456e-06, 'train_speed(iter/s)': 0.445516, 'epoch': 0.5104501607717041, 'consumed_train_tokens': 1098242}

132

{'loss': 0.7496801853179932, 'learning_rate': 8.722143864598025e-06, 'train_speed(iter/s)': 0.445672, 'epoch': 0.5144694533762058, 'consumed_train_tokens': 1106644}

133

{'loss': 0.8276382446289062, 'learning_rate': 8.708039492242596e-06, 'train_speed(iter/s)': 0.445692, 'epoch': 0.5184887459807074, 'consumed_train_tokens': 1115731}

134

{'loss': 0.7413704872131348, 'learning_rate': 8.693935119887165e-06, 'train_speed(iter/s)': 0.445762, 'epoch': 0.522508038585209, 'consumed_train_tokens': 1124065}

135

{'loss': 0.8089105606079101, 'learning_rate': 8.679830747531736e-06, 'train_speed(iter/s)': 0.445859, 'epoch': 0.5265273311897106, 'consumed_train_tokens': 1132465}

136

{'loss': 0.8569328308105468, 'learning_rate': 8.665726375176305e-06, 'train_speed(iter/s)': 0.445923, 'epoch': 0.5305466237942122, 'consumed_train_tokens': 1141328}

137

{'loss': 0.8635215759277344, 'learning_rate': 8.651622002820876e-06, 'train_speed(iter/s)': 0.445982, 'epoch': 0.5345659163987139, 'consumed_train_tokens': 1149535}

138

{'loss': 0.8810981750488281, 'learning_rate': 8.637517630465445e-06, 'train_speed(iter/s)': 0.446034, 'epoch': 0.5385852090032154, 'consumed_train_tokens': 1157977}

139

{'loss': 0.8806976318359375, 'learning_rate': 8.623413258110014e-06, 'train_speed(iter/s)': 0.446078, 'epoch': 0.542604501607717, 'consumed_train_tokens': 1166892}

140

{'loss': 0.8513910293579101, 'learning_rate': 8.609308885754585e-06, 'train_speed(iter/s)': 0.446059, 'epoch': 0.5466237942122186, 'consumed_train_tokens': 1175642}

141

{'loss': 0.8873628616333008, 'learning_rate': 8.595204513399154e-06, 'train_speed(iter/s)': 0.44613, 'epoch': 0.5506430868167203, 'consumed_train_tokens': 1184499}

142

{'loss': 0.7933486461639404, 'learning_rate': 8.581100141043725e-06, 'train_speed(iter/s)': 0.446187, 'epoch': 0.5546623794212219, 'consumed_train_tokens': 1193253}

143

{'loss': 0.9358002662658691, 'learning_rate': 8.566995768688294e-06, 'train_speed(iter/s)': 0.446218, 'epoch': 0.5586816720257235, 'consumed_train_tokens': 1201514}

144

{'loss': 0.7827283859252929, 'learning_rate': 8.552891396332865e-06, 'train_speed(iter/s)': 0.446289, 'epoch': 0.5627009646302251, 'consumed_train_tokens': 1210455}

145

{'loss': 0.8588062286376953, 'learning_rate': 8.538787023977434e-06, 'train_speed(iter/s)': 0.446347, 'epoch': 0.5667202572347267, 'consumed_train_tokens': 1219742}

146

{'loss': 0.88583402633667, 'learning_rate': 8.524682651622003e-06, 'train_speed(iter/s)': 0.446433, 'epoch': 0.5707395498392283, 'consumed_train_tokens': 1228527}

147

{'loss': 0.8114978790283203, 'learning_rate': 8.510578279266574e-06, 'train_speed(iter/s)': 0.446423, 'epoch': 0.5747588424437299, 'consumed_train_tokens': 1237145}

148

{'loss': 0.7785935878753663, 'learning_rate': 8.496473906911143e-06, 'train_speed(iter/s)': 0.446426, 'epoch': 0.5787781350482315, 'consumed_train_tokens': 1246165}

149

{'loss': 0.7470799922943115, 'learning_rate': 8.482369534555714e-06, 'train_speed(iter/s)': 0.446491, 'epoch': 0.5827974276527331, 'consumed_train_tokens': 1255058}

150

{'loss': 0.7341714859008789, 'learning_rate': 8.468265162200283e-06, 'train_speed(iter/s)': 0.446513, 'epoch': 0.5868167202572347, 'consumed_train_tokens': 1264641}

151

{'loss': 0.814209270477295, 'learning_rate': 8.454160789844854e-06, 'train_speed(iter/s)': 0.446604, 'epoch': 0.5908360128617364, 'consumed_train_tokens': 1273656}

152

{'loss': 0.848335075378418, 'learning_rate': 8.440056417489423e-06, 'train_speed(iter/s)': 0.446621, 'epoch': 0.594855305466238, 'consumed_train_tokens': 1282231}

153

{'loss': 0.7860597133636474, 'learning_rate': 8.425952045133992e-06, 'train_speed(iter/s)': 0.446675, 'epoch': 0.5988745980707395, 'consumed_train_tokens': 1290766}

154

{'loss': 0.8485092163085938, 'learning_rate': 8.411847672778563e-06, 'train_speed(iter/s)': 0.446644, 'epoch': 0.6028938906752411, 'consumed_train_tokens': 1299299}

155

{'loss': 0.7872490882873535, 'learning_rate': 8.397743300423132e-06, 'train_speed(iter/s)': 0.446818, 'epoch': 0.6069131832797428, 'consumed_train_tokens': 1307628}

156

{'loss': 0.9092720985412598, 'learning_rate': 8.383638928067703e-06, 'train_speed(iter/s)': 0.446817, 'epoch': 0.6109324758842444, 'consumed_train_tokens': 1316158}

157

{'loss': 0.7977198600769043, 'learning_rate': 8.369534555712272e-06, 'train_speed(iter/s)': 0.44686, 'epoch': 0.614951768488746, 'consumed_train_tokens': 1324459}

158

{'loss': 0.7538899421691895, 'learning_rate': 8.355430183356841e-06, 'train_speed(iter/s)': 0.446938, 'epoch': 0.6189710610932476, 'consumed_train_tokens': 1333168}

159

{'loss': 0.7629237651824952, 'learning_rate': 8.34132581100141e-06, 'train_speed(iter/s)': 0.446986, 'epoch': 0.6229903536977492, 'consumed_train_tokens': 1341647}

160

{'loss': 0.7519967079162597, 'learning_rate': 8.327221438645981e-06, 'train_speed(iter/s)': 0.447075, 'epoch': 0.6270096463022508, 'consumed_train_tokens': 1351202}

161

{'loss': 0.8206949234008789, 'learning_rate': 8.31311706629055e-06, 'train_speed(iter/s)': 0.447104, 'epoch': 0.6310289389067524, 'consumed_train_tokens': 1359863}

162

{'loss': 0.8194667816162109, 'learning_rate': 8.29901269393512e-06, 'train_speed(iter/s)': 0.447131, 'epoch': 0.635048231511254, 'consumed_train_tokens': 1368982}

163

{'loss': 0.7097901344299317, 'learning_rate': 8.28490832157969e-06, 'train_speed(iter/s)': 0.44711, 'epoch': 0.6390675241157556, 'consumed_train_tokens': 1377714}

164

{'loss': 0.7913718223571777, 'learning_rate': 8.270803949224259e-06, 'train_speed(iter/s)': 0.447161, 'epoch': 0.6430868167202572, 'consumed_train_tokens': 1386698}

165

{'loss': 0.8007086753845215, 'learning_rate': 8.25669957686883e-06, 'train_speed(iter/s)': 0.447243, 'epoch': 0.6471061093247589, 'consumed_train_tokens': 1395387}

166

{'loss': 0.7965718269348144, 'learning_rate': 8.242595204513399e-06, 'train_speed(iter/s)': 0.447236, 'epoch': 0.6511254019292605, 'consumed_train_tokens': 1405342}

167

{'loss': 0.8811780929565429, 'learning_rate': 8.22849083215797e-06, 'train_speed(iter/s)': 0.447312, 'epoch': 0.655144694533762, 'consumed_train_tokens': 1413756}

168

{'loss': 0.7270000457763672, 'learning_rate': 8.214386459802539e-06, 'train_speed(iter/s)': 0.447321, 'epoch': 0.6591639871382636, 'consumed_train_tokens': 1422354}

169

{'loss': 0.725405216217041, 'learning_rate': 8.20028208744711e-06, 'train_speed(iter/s)': 0.4474, 'epoch': 0.6631832797427653, 'consumed_train_tokens': 1431285}

170

{'loss': 0.7713145256042481, 'learning_rate': 8.186177715091679e-06, 'train_speed(iter/s)': 0.447362, 'epoch': 0.6672025723472669, 'consumed_train_tokens': 1439309}

171

{'loss': 0.7872252941131592, 'learning_rate': 8.172073342736248e-06, 'train_speed(iter/s)': 0.447336, 'epoch': 0.6712218649517685, 'consumed_train_tokens': 1447904}

172

{'loss': 0.6342533588409424, 'learning_rate': 8.157968970380819e-06, 'train_speed(iter/s)': 0.44733, 'epoch': 0.6752411575562701, 'consumed_train_tokens': 1456810}

173

{'loss': 0.722012996673584, 'learning_rate': 8.143864598025388e-06, 'train_speed(iter/s)': 0.447323, 'epoch': 0.6792604501607717, 'consumed_train_tokens': 1465625}

174

{'loss': 0.7455225944519043, 'learning_rate': 8.129760225669959e-06, 'train_speed(iter/s)': 0.447372, 'epoch': 0.6832797427652733, 'consumed_train_tokens': 1474119}

175

{'loss': 0.8651277542114257, 'learning_rate': 8.115655853314528e-06, 'train_speed(iter/s)': 0.447408, 'epoch': 0.6872990353697749, 'consumed_train_tokens': 1483013}

176

{'loss': 0.8163820266723633, 'learning_rate': 8.101551480959099e-06, 'train_speed(iter/s)': 0.447449, 'epoch': 0.6913183279742765, 'consumed_train_tokens': 1492122}

177

{'loss': 0.808280086517334, 'learning_rate': 8.087447108603668e-06, 'train_speed(iter/s)': 0.447385, 'epoch': 0.6953376205787781, 'consumed_train_tokens': 1500362}

178

{'loss': 0.7753993511199951, 'learning_rate': 8.073342736248237e-06, 'train_speed(iter/s)': 0.447427, 'epoch': 0.6993569131832797, 'consumed_train_tokens': 1509360}

179

{'loss': 0.7675658226013183, 'learning_rate': 8.059238363892808e-06, 'train_speed(iter/s)': 0.447422, 'epoch': 0.7033762057877814, 'consumed_train_tokens': 1517826}

180

{'loss': 0.9341964721679688, 'learning_rate': 8.045133991537377e-06, 'train_speed(iter/s)': 0.447408, 'epoch': 0.707395498392283, 'consumed_train_tokens': 1527075}

181

{'loss': 0.8163600921630859, 'learning_rate': 8.031029619181948e-06, 'train_speed(iter/s)': 0.447414, 'epoch': 0.7114147909967846, 'consumed_train_tokens': 1536051}

182

{'loss': 0.8144311904907227, 'learning_rate': 8.016925246826517e-06, 'train_speed(iter/s)': 0.447461, 'epoch': 0.7154340836012861, 'consumed_train_tokens': 1544135}

183

{'loss': 0.8243328094482422, 'learning_rate': 8.002820874471088e-06, 'train_speed(iter/s)': 0.447481, 'epoch': 0.7194533762057878, 'consumed_train_tokens': 1553765}

184

{'loss': 0.7580021858215332, 'learning_rate': 7.988716502115657e-06, 'train_speed(iter/s)': 0.447529, 'epoch': 0.7234726688102894, 'consumed_train_tokens': 1562001}

185

{'loss': 0.7643326759338379, 'learning_rate': 7.974612129760226e-06, 'train_speed(iter/s)': 0.447536, 'epoch': 0.727491961414791, 'consumed_train_tokens': 1570612}

186

{'loss': 0.7521597862243652, 'learning_rate': 7.960507757404797e-06, 'train_speed(iter/s)': 0.447586, 'epoch': 0.7315112540192926, 'consumed_train_tokens': 1579369}

187

{'loss': 0.8676981925964355, 'learning_rate': 7.946403385049366e-06, 'train_speed(iter/s)': 0.447614, 'epoch': 0.7355305466237942, 'consumed_train_tokens': 1587709}

188

{'loss': 0.8355885505676269, 'learning_rate': 7.932299012693936e-06, 'train_speed(iter/s)': 0.447666, 'epoch': 0.7395498392282959, 'consumed_train_tokens': 1596900}

189

{'loss': 0.797625732421875, 'learning_rate': 7.918194640338506e-06, 'train_speed(iter/s)': 0.447684, 'epoch': 0.7435691318327974, 'consumed_train_tokens': 1605279}

190

{'loss': 0.7058095932006836, 'learning_rate': 7.904090267983075e-06, 'train_speed(iter/s)': 0.447729, 'epoch': 0.747588424437299, 'consumed_train_tokens': 1614021}

191

{'loss': 0.6820828914642334, 'learning_rate': 7.889985895627646e-06, 'train_speed(iter/s)': 0.447773, 'epoch': 0.7516077170418006, 'consumed_train_tokens': 1623269}

192

{'loss': 0.6799546241760254, 'learning_rate': 7.875881523272215e-06, 'train_speed(iter/s)': 0.447791, 'epoch': 0.7556270096463023, 'consumed_train_tokens': 1631422}

193

{'loss': 0.8855274200439454, 'learning_rate': 7.861777150916785e-06, 'train_speed(iter/s)': 0.447798, 'epoch': 0.7596463022508039, 'consumed_train_tokens': 1640254}

194

{'loss': 0.815947437286377, 'learning_rate': 7.847672778561355e-06, 'train_speed(iter/s)': 0.447857, 'epoch': 0.7636655948553055, 'consumed_train_tokens': 1648989}

195

{'loss': 0.7471772193908691, 'learning_rate': 7.833568406205925e-06, 'train_speed(iter/s)': 0.447923, 'epoch': 0.7676848874598071, 'consumed_train_tokens': 1657122}

196

{'loss': 0.8707752227783203, 'learning_rate': 7.819464033850494e-06, 'train_speed(iter/s)': 0.447929, 'epoch': 0.7717041800643086, 'consumed_train_tokens': 1665628}

197

{'loss': 0.8002625465393066, 'learning_rate': 7.805359661495064e-06, 'train_speed(iter/s)': 0.447938, 'epoch': 0.7757234726688103, 'consumed_train_tokens': 1674030}

198

{'loss': 0.7737696647644043, 'learning_rate': 7.791255289139634e-06, 'train_speed(iter/s)': 0.447947, 'epoch': 0.7797427652733119, 'consumed_train_tokens': 1682491}

199

{'loss': 0.8090566635131836, 'learning_rate': 7.777150916784204e-06, 'train_speed(iter/s)': 0.447932, 'epoch': 0.7837620578778135, 'consumed_train_tokens': 1691209}

200

{'loss': 0.9053695678710938, 'learning_rate': 7.763046544428774e-06, 'train_speed(iter/s)': 0.447906, 'epoch': 0.7877813504823151, 'consumed_train_tokens': 1700037}

201

{'loss': 0.7616188049316406, 'learning_rate': 7.748942172073343e-06, 'train_speed(iter/s)': 0.447919, 'epoch': 0.7918006430868167, 'consumed_train_tokens': 1707794}

202

{'loss': 0.7419300079345703, 'learning_rate': 7.734837799717914e-06, 'train_speed(iter/s)': 0.447943, 'epoch': 0.7958199356913184, 'consumed_train_tokens': 1715452}

203

{'loss': 0.8130973815917969, 'learning_rate': 7.720733427362483e-06, 'train_speed(iter/s)': 0.448001, 'epoch': 0.7998392282958199, 'consumed_train_tokens': 1723982}

204

{'loss': 0.8605534553527832, 'learning_rate': 7.706629055007052e-06, 'train_speed(iter/s)': 0.448064, 'epoch': 0.8038585209003215, 'consumed_train_tokens': 1732852}

205

{'loss': 0.8327526092529297, 'learning_rate': 7.692524682651623e-06, 'train_speed(iter/s)': 0.448101, 'epoch': 0.8078778135048231, 'consumed_train_tokens': 1741919}

206

{'loss': 0.8319933891296387, 'learning_rate': 7.678420310296192e-06, 'train_speed(iter/s)': 0.448141, 'epoch': 0.8118971061093248, 'consumed_train_tokens': 1751021}

207

{'loss': 0.8060617446899414, 'learning_rate': 7.664315937940763e-06, 'train_speed(iter/s)': 0.448159, 'epoch': 0.8159163987138264, 'consumed_train_tokens': 1759577}

208

{'loss': 0.7634103775024415, 'learning_rate': 7.650211565585332e-06, 'train_speed(iter/s)': 0.448131, 'epoch': 0.819935691318328, 'consumed_train_tokens': 1767609}

209

{'loss': 0.7473262786865235, 'learning_rate': 7.636107193229901e-06, 'train_speed(iter/s)': 0.44814, 'epoch': 0.8239549839228296, 'consumed_train_tokens': 1777004}

210

{'loss': 0.8310269355773926, 'learning_rate': 7.622002820874471e-06, 'train_speed(iter/s)': 0.448111, 'epoch': 0.8279742765273312, 'consumed_train_tokens': 1785831}

211

{'loss': 0.7715398788452148, 'learning_rate': 7.607898448519041e-06, 'train_speed(iter/s)': 0.448122, 'epoch': 0.8319935691318328, 'consumed_train_tokens': 1794351}

212

{'loss': 0.8811748504638672, 'learning_rate': 7.5937940761636105e-06, 'train_speed(iter/s)': 0.448119, 'epoch': 0.8360128617363344, 'consumed_train_tokens': 1803155}

213

{'loss': 0.7232779026031494, 'learning_rate': 7.5796897038081805e-06, 'train_speed(iter/s)': 0.448117, 'epoch': 0.840032154340836, 'consumed_train_tokens': 1811831}

214

{'loss': 0.8314292907714844, 'learning_rate': 7.56558533145275e-06, 'train_speed(iter/s)': 0.448131, 'epoch': 0.8440514469453376, 'consumed_train_tokens': 1820769}

215

{'loss': 0.7099541664123535, 'learning_rate': 7.55148095909732e-06, 'train_speed(iter/s)': 0.448049, 'epoch': 0.8480707395498392, 'consumed_train_tokens': 1829294}

216

{'loss': 0.801812744140625, 'learning_rate': 7.53737658674189e-06, 'train_speed(iter/s)': 0.448024, 'epoch': 0.8520900321543409, 'consumed_train_tokens': 1837769}

217

{'loss': 0.7899589538574219, 'learning_rate': 7.52327221438646e-06, 'train_speed(iter/s)': 0.44801, 'epoch': 0.8561093247588425, 'consumed_train_tokens': 1846370}

218

{'loss': 0.7278778076171875, 'learning_rate': 7.50916784203103e-06, 'train_speed(iter/s)': 0.448003, 'epoch': 0.860128617363344, 'consumed_train_tokens': 1854995}

219

{'loss': 0.7869907855987549, 'learning_rate': 7.495063469675599e-06, 'train_speed(iter/s)': 0.448016, 'epoch': 0.8641479099678456, 'consumed_train_tokens': 1863282}

220

{'loss': 0.8214468002319336, 'learning_rate': 7.480959097320169e-06, 'train_speed(iter/s)': 0.448044, 'epoch': 0.8681672025723473, 'consumed_train_tokens': 1872103}

221

{'loss': 0.7864680767059327, 'learning_rate': 7.466854724964739e-06, 'train_speed(iter/s)': 0.448009, 'epoch': 0.8721864951768489, 'consumed_train_tokens': 1881203}

222

{'loss': 0.9791780471801758, 'learning_rate': 7.452750352609309e-06, 'train_speed(iter/s)': 0.44795, 'epoch': 0.8762057877813505, 'consumed_train_tokens': 1890461}

223

{'loss': 0.7152877807617187, 'learning_rate': 7.438645980253879e-06, 'train_speed(iter/s)': 0.447973, 'epoch': 0.8802250803858521, 'consumed_train_tokens': 1898810}

224

{'loss': 0.8107693672180176, 'learning_rate': 7.424541607898449e-06, 'train_speed(iter/s)': 0.447986, 'epoch': 0.8842443729903537, 'consumed_train_tokens': 1908026}

225

{'loss': 0.895068359375, 'learning_rate': 7.410437235543019e-06, 'train_speed(iter/s)': 0.448007, 'epoch': 0.8882636655948553, 'consumed_train_tokens': 1917258}

226

{'loss': 0.797370433807373, 'learning_rate': 7.396332863187588e-06, 'train_speed(iter/s)': 0.448014, 'epoch': 0.8922829581993569, 'consumed_train_tokens': 1926142}

227

{'loss': 0.7675235271453857, 'learning_rate': 7.382228490832158e-06, 'train_speed(iter/s)': 0.448037, 'epoch': 0.8963022508038585, 'consumed_train_tokens': 1935037}

228

{'loss': 0.7654478549957275, 'learning_rate': 7.368124118476728e-06, 'train_speed(iter/s)': 0.448079, 'epoch': 0.9003215434083601, 'consumed_train_tokens': 1943495}

229

{'loss': 0.7483449935913086, 'learning_rate': 7.354019746121298e-06, 'train_speed(iter/s)': 0.448096, 'epoch': 0.9043408360128617, 'consumed_train_tokens': 1952757}

230

{'loss': 0.8892021179199219, 'learning_rate': 7.339915373765868e-06, 'train_speed(iter/s)': 0.448122, 'epoch': 0.9083601286173634, 'consumed_train_tokens': 1961859}

231

{'loss': 0.8275882720947265, 'learning_rate': 7.325811001410438e-06, 'train_speed(iter/s)': 0.44811, 'epoch': 0.912379421221865, 'consumed_train_tokens': 1969998}

232

{'loss': 0.75252685546875, 'learning_rate': 7.311706629055007e-06, 'train_speed(iter/s)': 0.448143, 'epoch': 0.9163987138263665, 'consumed_train_tokens': 1978689}

233

{'loss': 0.7235503673553467, 'learning_rate': 7.297602256699577e-06, 'train_speed(iter/s)': 0.448152, 'epoch': 0.9204180064308681, 'consumed_train_tokens': 1987418}

234

{'loss': 0.8311444282531738, 'learning_rate': 7.283497884344147e-06, 'train_speed(iter/s)': 0.448191, 'epoch': 0.9244372990353698, 'consumed_train_tokens': 1996440}

235

{'loss': 0.772868537902832, 'learning_rate': 7.269393511988717e-06, 'train_speed(iter/s)': 0.448202, 'epoch': 0.9284565916398714, 'consumed_train_tokens': 2005205}

236

{'loss': 0.8467222213745117, 'learning_rate': 7.255289139633287e-06, 'train_speed(iter/s)': 0.448225, 'epoch': 0.932475884244373, 'consumed_train_tokens': 2014763}

237

{'loss': 0.7524055480957031, 'learning_rate': 7.241184767277857e-06, 'train_speed(iter/s)': 0.448226, 'epoch': 0.9364951768488746, 'consumed_train_tokens': 2024054}

238

{'loss': 0.802772331237793, 'learning_rate': 7.227080394922427e-06, 'train_speed(iter/s)': 0.4482, 'epoch': 0.9405144694533762, 'consumed_train_tokens': 2033078}

239

{'loss': 0.7793844223022461, 'learning_rate': 7.212976022566996e-06, 'train_speed(iter/s)': 0.448218, 'epoch': 0.9445337620578779, 'consumed_train_tokens': 2042117}

240

{'loss': 0.915839958190918, 'learning_rate': 7.198871650211566e-06, 'train_speed(iter/s)': 0.448207, 'epoch': 0.9485530546623794, 'consumed_train_tokens': 2051406}

241

{'loss': 0.8378366470336914, 'learning_rate': 7.184767277856136e-06, 'train_speed(iter/s)': 0.448221, 'epoch': 0.952572347266881, 'consumed_train_tokens': 2060288}

242

{'loss': 0.827699089050293, 'learning_rate': 7.170662905500706e-06, 'train_speed(iter/s)': 0.448204, 'epoch': 0.9565916398713826, 'consumed_train_tokens': 2069864}

243

{'loss': 0.817927360534668, 'learning_rate': 7.156558533145276e-06, 'train_speed(iter/s)': 0.448237, 'epoch': 0.9606109324758842, 'consumed_train_tokens': 2078387}

244

{'loss': 0.7249794006347656, 'learning_rate': 7.142454160789846e-06, 'train_speed(iter/s)': 0.448242, 'epoch': 0.9646302250803859, 'consumed_train_tokens': 2086944}

245

{'loss': 0.8343404769897461, 'learning_rate': 7.128349788434416e-06, 'train_speed(iter/s)': 0.44817, 'epoch': 0.9686495176848875, 'consumed_train_tokens': 2096007}

246

{'loss': 0.7365711688995361, 'learning_rate': 7.114245416078985e-06, 'train_speed(iter/s)': 0.448217, 'epoch': 0.9726688102893891, 'consumed_train_tokens': 2104866}

247

{'loss': 0.8940666198730469, 'learning_rate': 7.100141043723555e-06, 'train_speed(iter/s)': 0.44823, 'epoch': 0.9766881028938906, 'consumed_train_tokens': 2114102}

248

{'loss': 0.8422797203063965, 'learning_rate': 7.086036671368125e-06, 'train_speed(iter/s)': 0.448266, 'epoch': 0.9807073954983923, 'consumed_train_tokens': 2123369}

249

{'loss': 0.7144449710845947, 'learning_rate': 7.071932299012695e-06, 'train_speed(iter/s)': 0.44828, 'epoch': 0.9847266881028939, 'consumed_train_tokens': 2132291}

250

{'loss': 0.7417737960815429, 'learning_rate': 7.057827926657265e-06, 'train_speed(iter/s)': 0.448335, 'epoch': 0.9887459807073955, 'consumed_train_tokens': 2141744}

251

{'loss': 0.8929542541503906, 'learning_rate': 7.043723554301835e-06, 'train_speed(iter/s)': 0.448353, 'epoch': 0.9927652733118971, 'consumed_train_tokens': 2150353}

252

{'loss': 0.717988395690918, 'learning_rate': 7.029619181946405e-06, 'train_speed(iter/s)': 0.448359, 'epoch': 0.9967845659163987, 'consumed_train_tokens': 2158566}

253

{'eval_loss': 0.7605763077735901, 'eval_runtime': 35.4656, 'eval_samples_per_second': 28.196, 'eval_steps_per_second': 3.525, 'epoch': 1.0}

254

{'loss': 0.791725492477417, 'learning_rate': 7.015514809590974e-06, 'train_speed(iter/s)': 0.433906, 'epoch': 1.0008038585209003, 'consumed_train_tokens': 2166838}

255

{'loss': 0.37198500633239745, 'learning_rate': 7.001410437235544e-06, 'train_speed(iter/s)': 0.433935, 'epoch': 1.004823151125402, 'consumed_train_tokens': 2175088}

256

{'loss': 0.3763444185256958, 'learning_rate': 6.987306064880114e-06, 'train_speed(iter/s)': 0.433879, 'epoch': 1.0088424437299035, 'consumed_train_tokens': 2183877}

257

{'loss': 0.3263286590576172, 'learning_rate': 6.973201692524684e-06, 'train_speed(iter/s)': 0.433952, 'epoch': 1.0128617363344052, 'consumed_train_tokens': 2192048}

258

{'loss': 0.4006176948547363, 'learning_rate': 6.959097320169254e-06, 'train_speed(iter/s)': 0.433959, 'epoch': 1.0168810289389068, 'consumed_train_tokens': 2200493}

259

{'loss': 0.30408492088317873, 'learning_rate': 6.944992947813824e-06, 'train_speed(iter/s)': 0.434053, 'epoch': 1.0209003215434083, 'consumed_train_tokens': 2209442}

260

{'loss': 0.3548752307891846, 'learning_rate': 6.930888575458394e-06, 'train_speed(iter/s)': 0.434079, 'epoch': 1.02491961414791, 'consumed_train_tokens': 2217380}

261

{'loss': 0.30938494205474854, 'learning_rate': 6.916784203102962e-06, 'train_speed(iter/s)': 0.434105, 'epoch': 1.0289389067524115, 'consumed_train_tokens': 2225841}

262

{'loss': 0.33631718158721924, 'learning_rate': 6.902679830747532e-06, 'train_speed(iter/s)': 0.434175, 'epoch': 1.0329581993569132, 'consumed_train_tokens': 2234880}

263

{'loss': 0.35761685371398927, 'learning_rate': 6.888575458392102e-06, 'train_speed(iter/s)': 0.434204, 'epoch': 1.0369774919614148, 'consumed_train_tokens': 2243008}

264

{'loss': 0.3714000701904297, 'learning_rate': 6.874471086036672e-06, 'train_speed(iter/s)': 0.434297, 'epoch': 1.0409967845659165, 'consumed_train_tokens': 2252085}

265

{'loss': 0.28180546760559083, 'learning_rate': 6.860366713681241e-06, 'train_speed(iter/s)': 0.434297, 'epoch': 1.045016077170418, 'consumed_train_tokens': 2260034}

266

{'loss': 0.4225193977355957, 'learning_rate': 6.846262341325811e-06, 'train_speed(iter/s)': 0.434369, 'epoch': 1.0490353697749195, 'consumed_train_tokens': 2268493}

267

{'loss': 0.43266935348510743, 'learning_rate': 6.832157968970381e-06, 'train_speed(iter/s)': 0.434421, 'epoch': 1.0530546623794212, 'consumed_train_tokens': 2277978}

268

{'loss': 0.38342995643615724, 'learning_rate': 6.818053596614951e-06, 'train_speed(iter/s)': 0.43446, 'epoch': 1.0570739549839228, 'consumed_train_tokens': 2286935}

269

{'loss': 0.41286520957946776, 'learning_rate': 6.803949224259521e-06, 'train_speed(iter/s)': 0.434553, 'epoch': 1.0610932475884245, 'consumed_train_tokens': 2295862}

270

{'loss': 0.3235588788986206, 'learning_rate': 6.789844851904091e-06, 'train_speed(iter/s)': 0.434565, 'epoch': 1.065112540192926, 'consumed_train_tokens': 2304822}

271

{'loss': 0.34587440490722654, 'learning_rate': 6.775740479548661e-06, 'train_speed(iter/s)': 0.434676, 'epoch': 1.0691318327974277, 'consumed_train_tokens': 2313885}

272

{'loss': 0.3458560466766357, 'learning_rate': 6.76163610719323e-06, 'train_speed(iter/s)': 0.434693, 'epoch': 1.0731511254019293, 'consumed_train_tokens': 2322588}

273

{'loss': 0.32102859020233154, 'learning_rate': 6.7475317348378e-06, 'train_speed(iter/s)': 0.434773, 'epoch': 1.077170418006431, 'consumed_train_tokens': 2331488}

274

{'loss': 0.37778096199035643, 'learning_rate': 6.73342736248237e-06, 'train_speed(iter/s)': 0.434805, 'epoch': 1.0811897106109325, 'consumed_train_tokens': 2339668}

275

{'loss': 0.32762956619262695, 'learning_rate': 6.71932299012694e-06, 'train_speed(iter/s)': 0.434833, 'epoch': 1.085209003215434, 'consumed_train_tokens': 2349552}

276

{'loss': 0.3793424129486084, 'learning_rate': 6.70521861777151e-06, 'train_speed(iter/s)': 0.434907, 'epoch': 1.0892282958199357, 'consumed_train_tokens': 2358439}

277

{'loss': 0.3124091148376465, 'learning_rate': 6.69111424541608e-06, 'train_speed(iter/s)': 0.434939, 'epoch': 1.0932475884244373, 'consumed_train_tokens': 2366281}

278

{'loss': 0.3009192943572998, 'learning_rate': 6.67700987306065e-06, 'train_speed(iter/s)': 0.43503, 'epoch': 1.097266881028939, 'consumed_train_tokens': 2375430}

279

{'loss': 0.3438709735870361, 'learning_rate': 6.662905500705219e-06, 'train_speed(iter/s)': 0.435042, 'epoch': 1.1012861736334405, 'consumed_train_tokens': 2384471}

280

{'loss': 0.3634363651275635, 'learning_rate': 6.648801128349789e-06, 'train_speed(iter/s)': 0.435085, 'epoch': 1.105305466237942, 'consumed_train_tokens': 2393102}

281

{'loss': 0.29756894111633303, 'learning_rate': 6.634696755994359e-06, 'train_speed(iter/s)': 0.435127, 'epoch': 1.1093247588424437, 'consumed_train_tokens': 2401721}

282

{'loss': 0.3688725471496582, 'learning_rate': 6.620592383638929e-06, 'train_speed(iter/s)': 0.435167, 'epoch': 1.1133440514469453, 'consumed_train_tokens': 2409630}

283

{'loss': 0.315659236907959, 'learning_rate': 6.6064880112834985e-06, 'train_speed(iter/s)': 0.435243, 'epoch': 1.117363344051447, 'consumed_train_tokens': 2418248}

284

{'loss': 0.31591851711273194, 'learning_rate': 6.5923836389280685e-06, 'train_speed(iter/s)': 0.435281, 'epoch': 1.1213826366559485, 'consumed_train_tokens': 2426205}

285

{'loss': 0.4254110336303711, 'learning_rate': 6.5782792665726385e-06, 'train_speed(iter/s)': 0.435344, 'epoch': 1.1254019292604502, 'consumed_train_tokens': 2435298}

286

{'loss': 0.3146360874176025, 'learning_rate': 6.564174894217208e-06, 'train_speed(iter/s)': 0.435374, 'epoch': 1.1294212218649518, 'consumed_train_tokens': 2444761}

287

{'loss': 0.3056559801101685, 'learning_rate': 6.5500705218617775e-06, 'train_speed(iter/s)': 0.435479, 'epoch': 1.1334405144694535, 'consumed_train_tokens': 2453351}

288

{'loss': 0.33408145904541015, 'learning_rate': 6.5359661495063475e-06, 'train_speed(iter/s)': 0.435516, 'epoch': 1.137459807073955, 'consumed_train_tokens': 2462336}

289

{'loss': 0.3553158760070801, 'learning_rate': 6.5218617771509175e-06, 'train_speed(iter/s)': 0.435547, 'epoch': 1.1414790996784565, 'consumed_train_tokens': 2471432}

290

{'loss': 0.34101595878601076, 'learning_rate': 6.5077574047954874e-06, 'train_speed(iter/s)': 0.435647, 'epoch': 1.1454983922829582, 'consumed_train_tokens': 2479752}

291

{'loss': 0.2914024829864502, 'learning_rate': 6.493653032440057e-06, 'train_speed(iter/s)': 0.435664, 'epoch': 1.1495176848874598, 'consumed_train_tokens': 2489111}

292

{'loss': 0.33856873512268065, 'learning_rate': 6.4795486600846265e-06, 'train_speed(iter/s)': 0.435738, 'epoch': 1.1535369774919615, 'consumed_train_tokens': 2497627}

293

{'loss': 0.29731574058532717, 'learning_rate': 6.4654442877291965e-06, 'train_speed(iter/s)': 0.435769, 'epoch': 1.157556270096463, 'consumed_train_tokens': 2506411}

294

{'loss': 0.362610912322998, 'learning_rate': 6.4513399153737664e-06, 'train_speed(iter/s)': 0.435855, 'epoch': 1.1615755627009645, 'consumed_train_tokens': 2515913}

295

{'loss': 0.3535648584365845, 'learning_rate': 6.437235543018336e-06, 'train_speed(iter/s)': 0.435878, 'epoch': 1.1655948553054662, 'consumed_train_tokens': 2524994}

296

{'loss': 0.30220880508422854, 'learning_rate': 6.423131170662906e-06, 'train_speed(iter/s)': 0.435921, 'epoch': 1.1696141479099678, 'consumed_train_tokens': 2533866}

297

{'loss': 0.3323086977005005, 'learning_rate': 6.409026798307476e-06, 'train_speed(iter/s)': 0.435968, 'epoch': 1.1736334405144695, 'consumed_train_tokens': 2542840}

298

{'loss': 0.33263111114501953, 'learning_rate': 6.394922425952046e-06, 'train_speed(iter/s)': 0.436003, 'epoch': 1.177652733118971, 'consumed_train_tokens': 2552112}

299

{'loss': 0.33649208545684817, 'learning_rate': 6.380818053596615e-06, 'train_speed(iter/s)': 0.436099, 'epoch': 1.1816720257234727, 'consumed_train_tokens': 2560696}

300

{'loss': 0.3943511962890625, 'learning_rate': 6.366713681241185e-06, 'train_speed(iter/s)': 0.436147, 'epoch': 1.1856913183279743, 'consumed_train_tokens': 2569434}

301

{'loss': 0.33062598705291746, 'learning_rate': 6.352609308885755e-06, 'train_speed(iter/s)': 0.436246, 'epoch': 1.189710610932476, 'consumed_train_tokens': 2578395}

302

{'loss': 0.3296172618865967, 'learning_rate': 6.324400564174895e-06, 'train_speed(iter/s)': 0.436323, 'epoch': 1.197749196141479, 'consumed_train_tokens': 2596226}

303

{'loss': 0.33703203201293946, 'learning_rate': 6.310296191819465e-06, 'train_speed(iter/s)': 0.436389, 'epoch': 1.2017684887459807, 'consumed_train_tokens': 2605181}

304

{'loss': 0.32495412826538084, 'learning_rate': 6.296191819464035e-06, 'train_speed(iter/s)': 0.436432, 'epoch': 1.2057877813504823, 'consumed_train_tokens': 2613262}

305

{'loss': 0.30184767246246336, 'learning_rate': 6.282087447108604e-06, 'train_speed(iter/s)': 0.436519, 'epoch': 1.209807073954984, 'consumed_train_tokens': 2621446}

306

{'loss': 0.3444897890090942, 'learning_rate': 6.267983074753174e-06, 'train_speed(iter/s)': 0.436552, 'epoch': 1.2138263665594855, 'consumed_train_tokens': 2630006}

307

{'loss': 0.2997079849243164, 'learning_rate': 6.253878702397744e-06, 'train_speed(iter/s)': 0.436613, 'epoch': 1.217845659163987, 'consumed_train_tokens': 2638731}

308

{'loss': 0.3489809513092041, 'learning_rate': 6.239774330042314e-06, 'train_speed(iter/s)': 0.436632, 'epoch': 1.2218649517684887, 'consumed_train_tokens': 2648155}

309

{'loss': 0.32632343769073485, 'learning_rate': 6.225669957686884e-06, 'train_speed(iter/s)': 0.436719, 'epoch': 1.2258842443729903, 'consumed_train_tokens': 2657687}

310

{'loss': 0.3357006311416626, 'learning_rate': 6.211565585331452e-06, 'train_speed(iter/s)': 0.43675, 'epoch': 1.229903536977492, 'consumed_train_tokens': 2666589}

311

{'loss': 0.32148141860961915, 'learning_rate': 6.197461212976022e-06, 'train_speed(iter/s)': 0.436771, 'epoch': 1.2339228295819935, 'consumed_train_tokens': 2674531}

312

{'loss': 0.3981813907623291, 'learning_rate': 6.183356840620592e-06, 'train_speed(iter/s)': 0.436844, 'epoch': 1.2379421221864952, 'consumed_train_tokens': 2683220}

313

{'loss': 0.36016454696655276, 'learning_rate': 6.169252468265162e-06, 'train_speed(iter/s)': 0.436873, 'epoch': 1.2419614147909968, 'consumed_train_tokens': 2691952}

314

{'loss': 0.3753058433532715, 'learning_rate': 6.155148095909732e-06, 'train_speed(iter/s)': 0.436941, 'epoch': 1.2459807073954985, 'consumed_train_tokens': 2699564}

315

{'loss': 0.34635348320007325, 'learning_rate': 6.141043723554302e-06, 'train_speed(iter/s)': 0.436964, 'epoch': 1.25, 'consumed_train_tokens': 2708154}

316

{'loss': 0.3527932405471802, 'learning_rate': 6.126939351198871e-06, 'train_speed(iter/s)': 0.437003, 'epoch': 1.2540192926045015, 'consumed_train_tokens': 2716668}

317

{'loss': 0.2911869287490845, 'learning_rate': 6.112834978843441e-06, 'train_speed(iter/s)': 0.437045, 'epoch': 1.2580385852090032, 'consumed_train_tokens': 2725275}

318

{'loss': 0.3689796686172485, 'learning_rate': 6.098730606488011e-06, 'train_speed(iter/s)': 0.437073, 'epoch': 1.2620578778135048, 'consumed_train_tokens': 2734103}

319

{'loss': 0.33817601203918457, 'learning_rate': 6.084626234132581e-06, 'train_speed(iter/s)': 0.43715, 'epoch': 1.2660771704180065, 'consumed_train_tokens': 2742967}

320

{'loss': 0.3313025712966919, 'learning_rate': 6.070521861777151e-06, 'train_speed(iter/s)': 0.437153, 'epoch': 1.270096463022508, 'consumed_train_tokens': 2752166}

321

{'loss': 0.36984782218933104, 'learning_rate': 6.056417489421721e-06, 'train_speed(iter/s)': 0.437244, 'epoch': 1.2741157556270095, 'consumed_train_tokens': 2760700}

322

{'loss': 0.3067387819290161, 'learning_rate': 6.042313117066291e-06, 'train_speed(iter/s)': 0.437248, 'epoch': 1.2781350482315113, 'consumed_train_tokens': 2769570}

323

{'loss': 0.3667274475097656, 'learning_rate': 6.02820874471086e-06, 'train_speed(iter/s)': 0.43734, 'epoch': 1.282154340836013, 'consumed_train_tokens': 2778883}

324

{'loss': 0.39458575248718264, 'learning_rate': 6.01410437235543e-06, 'train_speed(iter/s)': 0.437365, 'epoch': 1.2861736334405145, 'consumed_train_tokens': 2787218}

325

{'loss': 0.2834217071533203, 'learning_rate': 6e-06, 'train_speed(iter/s)': 0.437352, 'epoch': 1.290192926045016, 'consumed_train_tokens': 2795776}

326

{'loss': 0.3637289524078369, 'learning_rate': 5.98589562764457e-06, 'train_speed(iter/s)': 0.437432, 'epoch': 1.2942122186495177, 'consumed_train_tokens': 2804886}

327

{'loss': 0.37062458992004393, 'learning_rate': 5.97179125528914e-06, 'train_speed(iter/s)': 0.437463, 'epoch': 1.2982315112540193, 'consumed_train_tokens': 2813139}

328

{'loss': 0.3605234146118164, 'learning_rate': 5.95768688293371e-06, 'train_speed(iter/s)': 0.437545, 'epoch': 1.302250803858521, 'consumed_train_tokens': 2821403}

329

{'loss': 0.3432703256607056, 'learning_rate': 5.94358251057828e-06, 'train_speed(iter/s)': 0.437578, 'epoch': 1.3062700964630225, 'consumed_train_tokens': 2830446}

330

{'loss': 0.31415705680847167, 'learning_rate': 5.929478138222849e-06, 'train_speed(iter/s)': 0.437663, 'epoch': 1.310289389067524, 'consumed_train_tokens': 2839616}

331

{'loss': 0.3136310338973999, 'learning_rate': 5.915373765867419e-06, 'train_speed(iter/s)': 0.437675, 'epoch': 1.3143086816720257, 'consumed_train_tokens': 2848749}

332

{'loss': 0.266917085647583, 'learning_rate': 5.901269393511989e-06, 'train_speed(iter/s)': 0.437729, 'epoch': 1.3183279742765273, 'consumed_train_tokens': 2857613}

333

{'loss': 0.3219770431518555, 'learning_rate': 5.887165021156559e-06, 'train_speed(iter/s)': 0.437762, 'epoch': 1.322347266881029, 'consumed_train_tokens': 2866438}

334

{'loss': 0.33306136131286623, 'learning_rate': 5.873060648801129e-06, 'train_speed(iter/s)': 0.437784, 'epoch': 1.3263665594855305, 'consumed_train_tokens': 2874691}

335

{'loss': 0.3571188926696777, 'learning_rate': 5.858956276445699e-06, 'train_speed(iter/s)': 0.437873, 'epoch': 1.330385852090032, 'consumed_train_tokens': 2883348}

336

{'loss': 0.3263860702514648, 'learning_rate': 5.844851904090269e-06, 'train_speed(iter/s)': 0.437894, 'epoch': 1.3344051446945338, 'consumed_train_tokens': 2891817}

337

{'loss': 0.3573432445526123, 'learning_rate': 5.830747531734838e-06, 'train_speed(iter/s)': 0.43797, 'epoch': 1.3384244372990355, 'consumed_train_tokens': 2900239}

338

{'loss': 0.35084075927734376, 'learning_rate': 5.802538787023978e-06, 'train_speed(iter/s)': 0.438067, 'epoch': 1.3464630225080385, 'consumed_train_tokens': 2916936}

339

{'loss': 0.3660449028015137, 'learning_rate': 5.788434414668548e-06, 'train_speed(iter/s)': 0.438079, 'epoch': 1.3504823151125402, 'consumed_train_tokens': 2925508}

340

{'loss': 0.34335358142852784, 'learning_rate': 5.774330042313118e-06, 'train_speed(iter/s)': 0.438109, 'epoch': 1.3545016077170418, 'consumed_train_tokens': 2934264}

341

{'loss': 0.36290774345397947, 'learning_rate': 5.760225669957688e-06, 'train_speed(iter/s)': 0.43818, 'epoch': 1.3585209003215435, 'consumed_train_tokens': 2943236}

342

{'loss': 0.3482844352722168, 'learning_rate': 5.746121297602257e-06, 'train_speed(iter/s)': 0.438183, 'epoch': 1.362540192926045, 'consumed_train_tokens': 2951781}

343

{'loss': 0.2836845874786377, 'learning_rate': 5.732016925246827e-06, 'train_speed(iter/s)': 0.438262, 'epoch': 1.3665594855305465, 'consumed_train_tokens': 2959829}

344

{'loss': 0.35254745483398436, 'learning_rate': 5.717912552891397e-06, 'train_speed(iter/s)': 0.438276, 'epoch': 1.3705787781350482, 'consumed_train_tokens': 2968524}

345

{'loss': 0.3241276264190674, 'learning_rate': 5.703808180535967e-06, 'train_speed(iter/s)': 0.438362, 'epoch': 1.3745980707395498, 'consumed_train_tokens': 2977088}

346

{'loss': 0.32745277881622314, 'learning_rate': 5.689703808180537e-06, 'train_speed(iter/s)': 0.438368, 'epoch': 1.3786173633440515, 'consumed_train_tokens': 2985708}

347

{'loss': 0.36051030158996583, 'learning_rate': 5.675599435825107e-06, 'train_speed(iter/s)': 0.438389, 'epoch': 1.382636655948553, 'consumed_train_tokens': 2994400}

348

{'loss': 0.3407313346862793, 'learning_rate': 5.661495063469677e-06, 'train_speed(iter/s)': 0.438438, 'epoch': 1.3866559485530547, 'consumed_train_tokens': 3002752}

349

{'loss': 0.399715518951416, 'learning_rate': 5.647390691114246e-06, 'train_speed(iter/s)': 0.438461, 'epoch': 1.3906752411575563, 'consumed_train_tokens': 3011457}

350

{'loss': 0.3879087448120117, 'learning_rate': 5.633286318758816e-06, 'train_speed(iter/s)': 0.438542, 'epoch': 1.394694533762058, 'consumed_train_tokens': 3020663}

351

{'loss': 0.29447736740112307, 'learning_rate': 5.619181946403386e-06, 'train_speed(iter/s)': 0.438561, 'epoch': 1.3987138263665595, 'consumed_train_tokens': 3029490}

352

{'loss': 0.3241616725921631, 'learning_rate': 5.605077574047956e-06, 'train_speed(iter/s)': 0.438632, 'epoch': 1.402733118971061, 'consumed_train_tokens': 3038128}

353

{'loss': 0.34447331428527833, 'learning_rate': 5.590973201692526e-06, 'train_speed(iter/s)': 0.438641, 'epoch': 1.4067524115755627, 'consumed_train_tokens': 3047631}

354

{'loss': 0.31197123527526854, 'learning_rate': 5.576868829337096e-06, 'train_speed(iter/s)': 0.438667, 'epoch': 1.4107717041800643, 'consumed_train_tokens': 3056511}

355

{'loss': 0.30965251922607423, 'learning_rate': 5.562764456981666e-06, 'train_speed(iter/s)': 0.438728, 'epoch': 1.414790996784566, 'consumed_train_tokens': 3064764}

356

{'loss': 0.33586893081665037, 'learning_rate': 5.548660084626235e-06, 'train_speed(iter/s)': 0.43875, 'epoch': 1.4188102893890675, 'consumed_train_tokens': 3072969}

357

{'loss': 0.37369213104248045, 'learning_rate': 5.534555712270805e-06, 'train_speed(iter/s)': 0.438822, 'epoch': 1.422829581993569, 'consumed_train_tokens': 3082139}

358

{'loss': 0.3290096759796143, 'learning_rate': 5.520451339915375e-06, 'train_speed(iter/s)': 0.438851, 'epoch': 1.4268488745980707, 'consumed_train_tokens': 3091200}

359

{'loss': 0.38088788986206057, 'learning_rate': 5.506346967559945e-06, 'train_speed(iter/s)': 0.438916, 'epoch': 1.4308681672025725, 'consumed_train_tokens': 3100063}

360

{'loss': 0.39420456886291505, 'learning_rate': 5.492242595204514e-06, 'train_speed(iter/s)': 0.438929, 'epoch': 1.434887459807074, 'consumed_train_tokens': 3109103}

361

{'loss': 0.3881077289581299, 'learning_rate': 5.478138222849083e-06, 'train_speed(iter/s)': 0.438958, 'epoch': 1.4389067524115755, 'consumed_train_tokens': 3118232}

362

{'loss': 0.3411705493927002, 'learning_rate': 5.464033850493653e-06, 'train_speed(iter/s)': 0.439016, 'epoch': 1.4429260450160772, 'consumed_train_tokens': 3126347}

363

{'loss': 0.3566492319107056, 'learning_rate': 5.449929478138223e-06, 'train_speed(iter/s)': 0.439034, 'epoch': 1.4469453376205788, 'consumed_train_tokens': 3135231}

364

{'loss': 0.2862566947937012, 'learning_rate': 5.435825105782793e-06, 'train_speed(iter/s)': 0.439094, 'epoch': 1.4509646302250805, 'consumed_train_tokens': 3143635}

365

{'loss': 0.30328917503356934, 'learning_rate': 5.421720733427363e-06, 'train_speed(iter/s)': 0.439111, 'epoch': 1.454983922829582, 'consumed_train_tokens': 3152001}

366

{'loss': 0.2823933601379395, 'learning_rate': 5.407616361071933e-06, 'train_speed(iter/s)': 0.439167, 'epoch': 1.4590032154340835, 'consumed_train_tokens': 3160083}

367

{'loss': 0.2995652198791504, 'learning_rate': 5.393511988716502e-06, 'train_speed(iter/s)': 0.439189, 'epoch': 1.4630225080385852, 'consumed_train_tokens': 3168645}

368

{'loss': 0.3536720037460327, 'learning_rate': 5.379407616361072e-06, 'train_speed(iter/s)': 0.439243, 'epoch': 1.4670418006430868, 'consumed_train_tokens': 3177517}

369

{'loss': 0.39251155853271485, 'learning_rate': 5.365303244005642e-06, 'train_speed(iter/s)': 0.439248, 'epoch': 1.4710610932475885, 'consumed_train_tokens': 3186492}

370

{'loss': 0.3447323560714722, 'learning_rate': 5.351198871650212e-06, 'train_speed(iter/s)': 0.439247, 'epoch': 1.47508038585209, 'consumed_train_tokens': 3195151}

371

{'loss': 0.3396031618118286, 'learning_rate': 5.337094499294782e-06, 'train_speed(iter/s)': 0.439303, 'epoch': 1.4790996784565915, 'consumed_train_tokens': 3204272}

372

{'loss': 0.3353287220001221, 'learning_rate': 5.3229901269393516e-06, 'train_speed(iter/s)': 0.439316, 'epoch': 1.4831189710610932, 'consumed_train_tokens': 3212974}

373

{'loss': 0.33901548385620117, 'learning_rate': 5.294781382228491e-06, 'train_speed(iter/s)': 0.439394, 'epoch': 1.4911575562700965, 'consumed_train_tokens': 3230125}

374

{'loss': 0.32702207565307617, 'learning_rate': 5.280677009873061e-06, 'train_speed(iter/s)': 0.439455, 'epoch': 1.495176848874598, 'consumed_train_tokens': 3238669}

375

{'loss': 0.29780054092407227, 'learning_rate': 5.2665726375176306e-06, 'train_speed(iter/s)': 0.439442, 'epoch': 1.4991961414790997, 'consumed_train_tokens': 3246994}

376

{'loss': 0.3942999839782715, 'learning_rate': 5.2524682651622005e-06, 'train_speed(iter/s)': 0.439457, 'epoch': 1.5032154340836013, 'consumed_train_tokens': 3255810}

377

{'loss': 0.369649600982666, 'learning_rate': 5.2383638928067705e-06, 'train_speed(iter/s)': 0.439516, 'epoch': 1.507234726688103, 'consumed_train_tokens': 3263891}

378

{'loss': 0.3070779085159302, 'learning_rate': 5.2242595204513405e-06, 'train_speed(iter/s)': 0.439502, 'epoch': 1.5112540192926045, 'consumed_train_tokens': 3271985}

379

{'loss': 0.37681989669799804, 'learning_rate': 5.2101551480959104e-06, 'train_speed(iter/s)': 0.43957, 'epoch': 1.515273311897106, 'consumed_train_tokens': 3280107}

380

{'loss': 0.3610947370529175, 'learning_rate': 5.1960507757404795e-06, 'train_speed(iter/s)': 0.439594, 'epoch': 1.5192926045016077, 'consumed_train_tokens': 3288094}

381

{'loss': 0.26323461532592773, 'learning_rate': 5.1819464033850495e-06, 'train_speed(iter/s)': 0.439656, 'epoch': 1.5233118971061095, 'consumed_train_tokens': 3296489}

382

{'loss': 0.3855780601501465, 'learning_rate': 5.1678420310296195e-06, 'train_speed(iter/s)': 0.439677, 'epoch': 1.527331189710611, 'consumed_train_tokens': 3305432}

383

{'loss': 0.3363001823425293, 'learning_rate': 5.1537376586741894e-06, 'train_speed(iter/s)': 0.439708, 'epoch': 1.5313504823151125, 'consumed_train_tokens': 3314276}

384

{'loss': 0.34399867057800293, 'learning_rate': 5.139633286318759e-06, 'train_speed(iter/s)': 0.439765, 'epoch': 1.535369774919614, 'consumed_train_tokens': 3323469}

385

{'loss': 0.2675591230392456, 'learning_rate': 5.125528913963329e-06, 'train_speed(iter/s)': 0.43978, 'epoch': 1.5393890675241158, 'consumed_train_tokens': 3332781}

386

{'loss': 0.3214686155319214, 'learning_rate': 5.111424541607899e-06, 'train_speed(iter/s)': 0.439851, 'epoch': 1.5434083601286175, 'consumed_train_tokens': 3341093}

387

{'loss': 0.3759006977081299, 'learning_rate': 5.0973201692524684e-06, 'train_speed(iter/s)': 0.439857, 'epoch': 1.547427652733119, 'consumed_train_tokens': 3349534}

388

{'loss': 0.35971932411193847, 'learning_rate': 5.083215796897038e-06, 'train_speed(iter/s)': 0.439901, 'epoch': 1.5514469453376205, 'consumed_train_tokens': 3358633}

389

{'loss': 0.3814763307571411, 'learning_rate': 5.069111424541608e-06, 'train_speed(iter/s)': 0.439917, 'epoch': 1.555466237942122, 'consumed_train_tokens': 3367804}

390

{'loss': 0.3414622783660889, 'learning_rate': 5.055007052186178e-06, 'train_speed(iter/s)': 0.439957, 'epoch': 1.5594855305466238, 'consumed_train_tokens': 3375949}

391

{'loss': 0.3163294315338135, 'learning_rate': 5.040902679830748e-06, 'train_speed(iter/s)': 0.440008, 'epoch': 1.5635048231511255, 'consumed_train_tokens': 3384501}

392

{'loss': 0.36906886100769043, 'learning_rate': 5.026798307475318e-06, 'train_speed(iter/s)': 0.440018, 'epoch': 1.567524115755627, 'consumed_train_tokens': 3393053}

393

{'loss': 0.4019340515136719, 'learning_rate': 5.012693935119887e-06, 'train_speed(iter/s)': 0.440085, 'epoch': 1.5715434083601285, 'consumed_train_tokens': 3401694}

394

{'loss': 0.34444541931152345, 'learning_rate': 4.998589562764457e-06, 'train_speed(iter/s)': 0.440104, 'epoch': 1.5755627009646302, 'consumed_train_tokens': 3409625}

395

{'loss': 0.3973322868347168, 'learning_rate': 4.984485190409027e-06, 'train_speed(iter/s)': 0.44018, 'epoch': 1.579581993569132, 'consumed_train_tokens': 3418144}

396

{'loss': 0.3354644775390625, 'learning_rate': 4.970380818053597e-06, 'train_speed(iter/s)': 0.44017, 'epoch': 1.5836012861736335, 'consumed_train_tokens': 3426780}

397

{'loss': 0.39513344764709474, 'learning_rate': 4.956276445698166e-06, 'train_speed(iter/s)': 0.440209, 'epoch': 1.587620578778135, 'consumed_train_tokens': 3435175}

398

{'loss': 0.37238202095031736, 'learning_rate': 4.942172073342736e-06, 'train_speed(iter/s)': 0.440229, 'epoch': 1.5916398713826365, 'consumed_train_tokens': 3443443}

399

{'loss': 0.297206974029541, 'learning_rate': 4.928067700987306e-06, 'train_speed(iter/s)': 0.440242, 'epoch': 1.5956591639871383, 'consumed_train_tokens': 3452378}

400

{'loss': 0.31514577865600585, 'learning_rate': 4.913963328631876e-06, 'train_speed(iter/s)': 0.440305, 'epoch': 1.59967845659164, 'consumed_train_tokens': 3460502}

401

{'loss': 0.29359347820281984, 'learning_rate': 4.899858956276446e-06, 'train_speed(iter/s)': 0.440324, 'epoch': 1.6036977491961415, 'consumed_train_tokens': 3468847}

402

{'loss': 0.3141382455825806, 'learning_rate': 4.885754583921016e-06, 'train_speed(iter/s)': 0.440386, 'epoch': 1.607717041800643, 'consumed_train_tokens': 3476967}

403

{'loss': 0.3364486932754517, 'learning_rate': 4.871650211565585e-06, 'train_speed(iter/s)': 0.440396, 'epoch': 1.6117363344051447, 'consumed_train_tokens': 3485307}

404

{'loss': 0.2899660587310791, 'learning_rate': 4.857545839210155e-06, 'train_speed(iter/s)': 0.440467, 'epoch': 1.6157556270096463, 'consumed_train_tokens': 3493457}

405

{'loss': 0.3379205703735352, 'learning_rate': 4.843441466854725e-06, 'train_speed(iter/s)': 0.440483, 'epoch': 1.619774919614148, 'consumed_train_tokens': 3502072}

406

{'loss': 0.31379060745239257, 'learning_rate': 4.829337094499295e-06, 'train_speed(iter/s)': 0.44052, 'epoch': 1.6237942122186495, 'consumed_train_tokens': 3511108}

407

{'loss': 0.37242960929870605, 'learning_rate': 4.815232722143865e-06, 'train_speed(iter/s)': 0.44054, 'epoch': 1.627813504823151, 'consumed_train_tokens': 3519500}

408

{'loss': 0.3664816379547119, 'learning_rate': 4.801128349788435e-06, 'train_speed(iter/s)': 0.440554, 'epoch': 1.6318327974276527, 'consumed_train_tokens': 3528191}

409

{'loss': 0.32759971618652345, 'learning_rate': 4.787023977433004e-06, 'train_speed(iter/s)': 0.440608, 'epoch': 1.6358520900321545, 'consumed_train_tokens': 3536491}

410

{'loss': 0.3432008743286133, 'learning_rate': 4.772919605077574e-06, 'train_speed(iter/s)': 0.440611, 'epoch': 1.639871382636656, 'consumed_train_tokens': 3545342}

411

{'loss': 0.34554233551025393, 'learning_rate': 4.758815232722144e-06, 'train_speed(iter/s)': 0.440659, 'epoch': 1.6438906752411575, 'consumed_train_tokens': 3554666}

412

{'loss': 0.3751444101333618, 'learning_rate': 4.744710860366714e-06, 'train_speed(iter/s)': 0.440663, 'epoch': 1.647909967845659, 'consumed_train_tokens': 3563839}

413

{'loss': 0.3593360662460327, 'learning_rate': 4.730606488011284e-06, 'train_speed(iter/s)': 0.440689, 'epoch': 1.6519292604501608, 'consumed_train_tokens': 3572673}

414

{'loss': 0.33102548122406006, 'learning_rate': 4.716502115655854e-06, 'train_speed(iter/s)': 0.440726, 'epoch': 1.6559485530546625, 'consumed_train_tokens': 3581451}

415

{'loss': 0.3117652416229248, 'learning_rate': 4.702397743300424e-06, 'train_speed(iter/s)': 0.44073, 'epoch': 1.659967845659164, 'consumed_train_tokens': 3590343}

416

{'loss': 0.3097792625427246, 'learning_rate': 4.688293370944993e-06, 'train_speed(iter/s)': 0.440798, 'epoch': 1.6639871382636655, 'consumed_train_tokens': 3598539}

417

{'loss': 0.2929570198059082, 'learning_rate': 4.674188998589563e-06, 'train_speed(iter/s)': 0.440807, 'epoch': 1.6680064308681672, 'consumed_train_tokens': 3607480}

418

{'loss': 0.3934943199157715, 'learning_rate': 4.660084626234133e-06, 'train_speed(iter/s)': 0.440862, 'epoch': 1.6720257234726688, 'consumed_train_tokens': 3615600}

419

{'loss': 0.40728254318237306, 'learning_rate': 4.645980253878703e-06, 'train_speed(iter/s)': 0.440855, 'epoch': 1.6760450160771705, 'consumed_train_tokens': 3624527}

420

{'loss': 0.3436574935913086, 'learning_rate': 4.631875881523273e-06, 'train_speed(iter/s)': 0.440888, 'epoch': 1.680064308681672, 'consumed_train_tokens': 3633878}

421

{'loss': 0.3181351900100708, 'learning_rate': 4.617771509167843e-06, 'train_speed(iter/s)': 0.440926, 'epoch': 1.6840836012861735, 'consumed_train_tokens': 3643063}

422

{'loss': 0.29608664512634275, 'learning_rate': 4.603667136812412e-06, 'train_speed(iter/s)': 0.440939, 'epoch': 1.6881028938906752, 'consumed_train_tokens': 3652166}

423

{'loss': 0.34759092330932617, 'learning_rate': 4.589562764456982e-06, 'train_speed(iter/s)': 0.44099, 'epoch': 1.692122186495177, 'consumed_train_tokens': 3661521}

424

{'loss': 0.3503521203994751, 'learning_rate': 4.575458392101552e-06, 'train_speed(iter/s)': 0.440996, 'epoch': 1.6961414790996785, 'consumed_train_tokens': 3670326}

425

{'loss': 0.34438467025756836, 'learning_rate': 4.561354019746121e-06, 'train_speed(iter/s)': 0.441047, 'epoch': 1.70016077170418, 'consumed_train_tokens': 3679662}

426

{'loss': 0.3257571220397949, 'learning_rate': 4.547249647390691e-06, 'train_speed(iter/s)': 0.441066, 'epoch': 1.7041800643086815, 'consumed_train_tokens': 3687381}

427

{'loss': 0.3663474559783936, 'learning_rate': 4.533145275035261e-06, 'train_speed(iter/s)': 0.4411, 'epoch': 1.7081993569131833, 'consumed_train_tokens': 3696056}

428

{'loss': 0.32528252601623536, 'learning_rate': 4.519040902679831e-06, 'train_speed(iter/s)': 0.441134, 'epoch': 1.712218649517685, 'consumed_train_tokens': 3705722}

429

{'loss': 0.3107001781463623, 'learning_rate': 4.504936530324401e-06, 'train_speed(iter/s)': 0.441163, 'epoch': 1.7162379421221865, 'consumed_train_tokens': 3714321}

430

{'loss': 0.34585368633270264, 'learning_rate': 4.490832157968971e-06, 'train_speed(iter/s)': 0.441192, 'epoch': 1.720257234726688, 'consumed_train_tokens': 3723032}

431

{'loss': 0.34760055541992185, 'learning_rate': 4.476727785613541e-06, 'train_speed(iter/s)': 0.441201, 'epoch': 1.7242765273311897, 'consumed_train_tokens': 3732232}

432

{'loss': 0.29735689163208007, 'learning_rate': 4.46262341325811e-06, 'train_speed(iter/s)': 0.441254, 'epoch': 1.7282958199356915, 'consumed_train_tokens': 3740858}

433

{'loss': 0.30090861320495604, 'learning_rate': 4.44851904090268e-06, 'train_speed(iter/s)': 0.44127, 'epoch': 1.732315112540193, 'consumed_train_tokens': 3748797}

434

{'loss': 0.33608551025390626, 'learning_rate': 4.43441466854725e-06, 'train_speed(iter/s)': 0.441274, 'epoch': 1.7363344051446945, 'consumed_train_tokens': 3757209}

435

{'loss': 0.3375882387161255, 'learning_rate': 4.42031029619182e-06, 'train_speed(iter/s)': 0.441306, 'epoch': 1.740353697749196, 'consumed_train_tokens': 3766282}

436

{'loss': 0.31686484813690186, 'learning_rate': 4.40620592383639e-06, 'train_speed(iter/s)': 0.441321, 'epoch': 1.7443729903536977, 'consumed_train_tokens': 3775093}

437

{'loss': 0.3857429027557373, 'learning_rate': 4.39210155148096e-06, 'train_speed(iter/s)': 0.44135, 'epoch': 1.7483922829581995, 'consumed_train_tokens': 3784182}

438

{'loss': 0.28017287254333495, 'learning_rate': 4.37799717912553e-06, 'train_speed(iter/s)': 0.441354, 'epoch': 1.752411575562701, 'consumed_train_tokens': 3793277}

439

{'loss': 0.3496924161911011, 'learning_rate': 4.363892806770099e-06, 'train_speed(iter/s)': 0.441411, 'epoch': 1.7564308681672025, 'consumed_train_tokens': 3802116}

440

{'loss': 0.35175585746765137, 'learning_rate': 4.349788434414669e-06, 'train_speed(iter/s)': 0.441415, 'epoch': 1.760450160771704, 'consumed_train_tokens': 3811175}

441

{'loss': 0.3992527961730957, 'learning_rate': 4.335684062059239e-06, 'train_speed(iter/s)': 0.441471, 'epoch': 1.7644694533762058, 'consumed_train_tokens': 3820096}

442

{'loss': 0.3458489656448364, 'learning_rate': 4.321579689703809e-06, 'train_speed(iter/s)': 0.441488, 'epoch': 1.7684887459807075, 'consumed_train_tokens': 3829223}

443

{'loss': 0.3815749645233154, 'learning_rate': 4.307475317348379e-06, 'train_speed(iter/s)': 0.441514, 'epoch': 1.772508038585209, 'consumed_train_tokens': 3838701}

444

{'loss': 0.29511475563049316, 'learning_rate': 4.293370944992949e-06, 'train_speed(iter/s)': 0.441549, 'epoch': 1.7765273311897105, 'consumed_train_tokens': 3846890}

445

{'loss': 0.308378267288208, 'learning_rate': 4.279266572637519e-06, 'train_speed(iter/s)': 0.441561, 'epoch': 1.7805466237942122, 'consumed_train_tokens': 3855651}

446

{'loss': 0.3573030710220337, 'learning_rate': 4.265162200282088e-06, 'train_speed(iter/s)': 0.441612, 'epoch': 1.784565916398714, 'consumed_train_tokens': 3864449}

447

{'loss': 0.2966639518737793, 'learning_rate': 4.251057827926658e-06, 'train_speed(iter/s)': 0.441631, 'epoch': 1.7885852090032155, 'consumed_train_tokens': 3873131}

448

{'loss': 0.35056257247924805, 'learning_rate': 4.236953455571227e-06, 'train_speed(iter/s)': 0.441695, 'epoch': 1.792604501607717, 'consumed_train_tokens': 3882066}

449

{'loss': 0.2972948789596558, 'learning_rate': 4.222849083215797e-06, 'train_speed(iter/s)': 0.441713, 'epoch': 1.7966237942122185, 'consumed_train_tokens': 3890687}

450

{'loss': 0.3844153881072998, 'learning_rate': 4.208744710860367e-06, 'train_speed(iter/s)': 0.441743, 'epoch': 1.8006430868167203, 'consumed_train_tokens': 3899172}

451

{'loss': 0.3405346393585205, 'learning_rate': 4.194640338504937e-06, 'train_speed(iter/s)': 0.441779, 'epoch': 1.804662379421222, 'consumed_train_tokens': 3907956}

452

{'loss': 0.3017918825149536, 'learning_rate': 4.180535966149507e-06, 'train_speed(iter/s)': 0.441787, 'epoch': 1.8086816720257235, 'consumed_train_tokens': 3915808}

453

{'loss': 0.3809141874313354, 'learning_rate': 4.166431593794077e-06, 'train_speed(iter/s)': 0.441805, 'epoch': 1.812700964630225, 'consumed_train_tokens': 3924549}

454

{'loss': 0.3929145812988281, 'learning_rate': 4.152327221438647e-06, 'train_speed(iter/s)': 0.441794, 'epoch': 1.8167202572347267, 'consumed_train_tokens': 3933435}

455

{'loss': 0.34644250869750975, 'learning_rate': 4.138222849083216e-06, 'train_speed(iter/s)': 0.441849, 'epoch': 1.8207395498392283, 'consumed_train_tokens': 3942378}

456

{'loss': 0.3093562126159668, 'learning_rate': 4.124118476727786e-06, 'train_speed(iter/s)': 0.441855, 'epoch': 1.82475884244373, 'consumed_train_tokens': 3951214}

457

{'loss': 0.3992335796356201, 'learning_rate': 4.110014104372356e-06, 'train_speed(iter/s)': 0.441883, 'epoch': 1.8287781350482315, 'consumed_train_tokens': 3960019}

458

{'loss': 0.3145911693572998, 'learning_rate': 4.095909732016926e-06, 'train_speed(iter/s)': 0.441902, 'epoch': 1.832797427652733, 'consumed_train_tokens': 3968943}

459

{'loss': 0.3576366424560547, 'learning_rate': 4.0818053596614956e-06, 'train_speed(iter/s)': 0.44191, 'epoch': 1.8368167202572347, 'consumed_train_tokens': 3978057}

460

{'loss': 0.36985933780670166, 'learning_rate': 4.0677009873060655e-06, 'train_speed(iter/s)': 0.441937, 'epoch': 1.8408360128617365, 'consumed_train_tokens': 3986555}

461

{'loss': 0.3282939910888672, 'learning_rate': 4.053596614950635e-06, 'train_speed(iter/s)': 0.441921, 'epoch': 1.844855305466238, 'consumed_train_tokens': 3996179}

462

{'loss': 0.35359184741973876, 'learning_rate': 4.039492242595205e-06, 'train_speed(iter/s)': 0.441973, 'epoch': 1.8488745980707395, 'consumed_train_tokens': 4005055}

463

{'loss': 0.39034504890441896, 'learning_rate': 4.0253878702397746e-06, 'train_speed(iter/s)': 0.441961, 'epoch': 1.852893890675241, 'consumed_train_tokens': 4014510}

464

{'loss': 0.3285416603088379, 'learning_rate': 4.0112834978843445e-06, 'train_speed(iter/s)': 0.441993, 'epoch': 1.8569131832797428, 'consumed_train_tokens': 4023166}

465

{'loss': 0.3536065101623535, 'learning_rate': 3.9971791255289145e-06, 'train_speed(iter/s)': 0.442026, 'epoch': 1.8609324758842445, 'consumed_train_tokens': 4031443}

466

{'loss': 0.2760436773300171, 'learning_rate': 3.9830747531734845e-06, 'train_speed(iter/s)': 0.442054, 'epoch': 1.864951768488746, 'consumed_train_tokens': 4039811}

467

{'loss': 0.3044134616851807, 'learning_rate': 3.968970380818054e-06, 'train_speed(iter/s)': 0.442098, 'epoch': 1.8689710610932475, 'consumed_train_tokens': 4048282}

468

{'loss': 0.35029337406158445, 'learning_rate': 3.9548660084626235e-06, 'train_speed(iter/s)': 0.442105, 'epoch': 1.8729903536977492, 'consumed_train_tokens': 4057277}

469

{'loss': 0.34403815269470217, 'learning_rate': 3.9407616361071935e-06, 'train_speed(iter/s)': 0.442154, 'epoch': 1.8770096463022508, 'consumed_train_tokens': 4065838}

470

{'loss': 0.39057583808898927, 'learning_rate': 3.9266572637517635e-06, 'train_speed(iter/s)': 0.44215, 'epoch': 1.8810289389067525, 'consumed_train_tokens': 4074466}

471

{'loss': 0.3372459411621094, 'learning_rate': 3.912552891396333e-06, 'train_speed(iter/s)': 0.442189, 'epoch': 1.885048231511254, 'consumed_train_tokens': 4083695}

472

{'loss': 0.3222489356994629, 'learning_rate': 3.898448519040903e-06, 'train_speed(iter/s)': 0.44222, 'epoch': 1.8890675241157555, 'consumed_train_tokens': 4092224}

473

{'loss': 0.3937295198440552, 'learning_rate': 3.8843441466854725e-06, 'train_speed(iter/s)': 0.442245, 'epoch': 1.8930868167202572, 'consumed_train_tokens': 4100971}

474

{'loss': 0.33322694301605227, 'learning_rate': 3.8702397743300425e-06, 'train_speed(iter/s)': 0.442273, 'epoch': 1.897106109324759, 'consumed_train_tokens': 4110122}

475

{'loss': 0.33219141960144044, 'learning_rate': 3.856135401974612e-06, 'train_speed(iter/s)': 0.442295, 'epoch': 1.9011254019292605, 'consumed_train_tokens': 4118626}

476

{'loss': 0.39002141952514646, 'learning_rate': 3.842031029619182e-06, 'train_speed(iter/s)': 0.442328, 'epoch': 1.905144694533762, 'consumed_train_tokens': 4127295}

477

{'loss': 0.35986464023590087, 'learning_rate': 3.8279266572637515e-06, 'train_speed(iter/s)': 0.442345, 'epoch': 1.9091639871382635, 'consumed_train_tokens': 4135740}

478

{'loss': 0.33466877937316897, 'learning_rate': 3.813822284908322e-06, 'train_speed(iter/s)': 0.442402, 'epoch': 1.9131832797427653, 'consumed_train_tokens': 4144167}

479

{'loss': 0.36184158325195315, 'learning_rate': 3.7997179125528914e-06, 'train_speed(iter/s)': 0.442397, 'epoch': 1.917202572347267, 'consumed_train_tokens': 4152850}

480

{'loss': 0.3154908180236816, 'learning_rate': 3.7856135401974614e-06, 'train_speed(iter/s)': 0.442428, 'epoch': 1.9212218649517685, 'consumed_train_tokens': 4161488}

481

{'loss': 0.24207019805908203, 'learning_rate': 3.7715091678420313e-06, 'train_speed(iter/s)': 0.442456, 'epoch': 1.92524115755627, 'consumed_train_tokens': 4169809}

482

{'loss': 0.2944824695587158, 'learning_rate': 3.757404795486601e-06, 'train_speed(iter/s)': 0.442485, 'epoch': 1.9292604501607717, 'consumed_train_tokens': 4177766}

483

{'loss': 0.3440701484680176, 'learning_rate': 3.743300423131171e-06, 'train_speed(iter/s)': 0.442505, 'epoch': 1.9332797427652733, 'consumed_train_tokens': 4186483}

484

{'loss': 0.3220889806747437, 'learning_rate': 3.729196050775741e-06, 'train_speed(iter/s)': 0.442509, 'epoch': 1.937299035369775, 'consumed_train_tokens': 4194406}

485

{'loss': 0.3129761219024658, 'learning_rate': 3.7150916784203108e-06, 'train_speed(iter/s)': 0.442556, 'epoch': 1.9413183279742765, 'consumed_train_tokens': 4202678}

486

{'loss': 0.3229027032852173, 'learning_rate': 3.7009873060648803e-06, 'train_speed(iter/s)': 0.442563, 'epoch': 1.945337620578778, 'consumed_train_tokens': 4211317}

487

{'loss': 0.3656746864318848, 'learning_rate': 3.6868829337094503e-06, 'train_speed(iter/s)': 0.442588, 'epoch': 1.9493569131832797, 'consumed_train_tokens': 4220092}

488

{'loss': 0.3667583465576172, 'learning_rate': 3.6727785613540202e-06, 'train_speed(iter/s)': 0.442624, 'epoch': 1.9533762057877815, 'consumed_train_tokens': 4227988}

489

{'loss': 0.30112106800079347, 'learning_rate': 3.6586741889985898e-06, 'train_speed(iter/s)': 0.442626, 'epoch': 1.957395498392283, 'consumed_train_tokens': 4237594}

490

{'loss': 0.33269314765930175, 'learning_rate': 3.6445698166431597e-06, 'train_speed(iter/s)': 0.442644, 'epoch': 1.9614147909967845, 'consumed_train_tokens': 4246320}

491

{'loss': 0.34230217933654783, 'learning_rate': 3.6304654442877297e-06, 'train_speed(iter/s)': 0.442658, 'epoch': 1.965434083601286, 'consumed_train_tokens': 4254935}

492

{'loss': 0.3924901485443115, 'learning_rate': 3.6163610719322997e-06, 'train_speed(iter/s)': 0.442699, 'epoch': 1.9694533762057878, 'consumed_train_tokens': 4265055}

493

{'loss': 0.3943302631378174, 'learning_rate': 3.602256699576869e-06, 'train_speed(iter/s)': 0.442704, 'epoch': 1.9734726688102895, 'consumed_train_tokens': 4273948}

494

{'loss': 0.337265682220459, 'learning_rate': 3.588152327221439e-06, 'train_speed(iter/s)': 0.442723, 'epoch': 1.977491961414791, 'consumed_train_tokens': 4282408}

495

{'loss': 0.33838815689086915, 'learning_rate': 3.574047954866009e-06, 'train_speed(iter/s)': 0.442745, 'epoch': 1.9815112540192925, 'consumed_train_tokens': 4291555}

496

{'loss': 0.29926974773406984, 'learning_rate': 3.5599435825105787e-06, 'train_speed(iter/s)': 0.442748, 'epoch': 1.9855305466237942, 'consumed_train_tokens': 4299886}

497

{'loss': 0.2888603210449219, 'learning_rate': 3.5458392101551486e-06, 'train_speed(iter/s)': 0.44278, 'epoch': 1.989549839228296, 'consumed_train_tokens': 4308591}

498

{'loss': 0.3709650754928589, 'learning_rate': 3.5317348377997177e-06, 'train_speed(iter/s)': 0.44279, 'epoch': 1.9935691318327975, 'consumed_train_tokens': 4317023}

499

{'loss': 0.3504521131515503, 'learning_rate': 3.5176304654442877e-06, 'train_speed(iter/s)': 0.442809, 'epoch': 1.997588424437299, 'consumed_train_tokens': 4325745}

500

{'eval_loss': 0.838269829750061, 'eval_runtime': 35.4964, 'eval_samples_per_second': 28.172, 'eval_steps_per_second': 3.521, 'epoch': 2.0}

501

{'loss': 0.26158292293548585, 'learning_rate': 3.5035260930888577e-06, 'train_speed(iter/s)': 0.435607, 'epoch': 2.0016077170418005, 'consumed_train_tokens': 4333901}

502

{'loss': 0.0804736077785492, 'learning_rate': 3.4894217207334276e-06, 'train_speed(iter/s)': 0.435622, 'epoch': 2.0056270096463025, 'consumed_train_tokens': 4342578}

503

{'loss': 0.07877426147460938, 'learning_rate': 3.475317348377997e-06, 'train_speed(iter/s)': 0.435591, 'epoch': 2.009646302250804, 'consumed_train_tokens': 4351237}

504

{'loss': 0.08058228492736816, 'learning_rate': 3.461212976022567e-06, 'train_speed(iter/s)': 0.435618, 'epoch': 2.0136655948553055, 'consumed_train_tokens': 4360849}

505

{'loss': 0.048887109756469725, 'learning_rate': 3.447108603667137e-06, 'train_speed(iter/s)': 0.435626, 'epoch': 2.017684887459807, 'consumed_train_tokens': 4370386}

506

{'loss': 0.07579739093780517, 'learning_rate': 3.4330042313117066e-06, 'train_speed(iter/s)': 0.435637, 'epoch': 2.0217041800643085, 'consumed_train_tokens': 4379121}

507

{'loss': 0.10913642644882202, 'learning_rate': 3.4188998589562766e-06, 'train_speed(iter/s)': 0.435692, 'epoch': 2.0257234726688105, 'consumed_train_tokens': 4387994}

508

{'loss': 0.05811023712158203, 'learning_rate': 3.4047954866008466e-06, 'train_speed(iter/s)': 0.435709, 'epoch': 2.029742765273312, 'consumed_train_tokens': 4396316}

509

{'loss': 0.08209856152534485, 'learning_rate': 3.390691114245416e-06, 'train_speed(iter/s)': 0.435742, 'epoch': 2.0337620578778135, 'consumed_train_tokens': 4405282}

510

{'loss': 0.07104701399803162, 'learning_rate': 3.376586741889986e-06, 'train_speed(iter/s)': 0.435763, 'epoch': 2.037781350482315, 'consumed_train_tokens': 4414117}

511

{'loss': 0.07782026529312133, 'learning_rate': 3.362482369534556e-06, 'train_speed(iter/s)': 0.435823, 'epoch': 2.0418006430868165, 'consumed_train_tokens': 4423593}

512

{'loss': 0.07862737774848938, 'learning_rate': 3.348377997179126e-06, 'train_speed(iter/s)': 0.435847, 'epoch': 2.0458199356913185, 'consumed_train_tokens': 4431831}

513

{'loss': 0.08319194912910462, 'learning_rate': 3.3342736248236955e-06, 'train_speed(iter/s)': 0.435856, 'epoch': 2.04983922829582, 'consumed_train_tokens': 4440848}

514

{'loss': 0.05780504941940308, 'learning_rate': 3.3201692524682655e-06, 'train_speed(iter/s)': 0.435909, 'epoch': 2.0538585209003215, 'consumed_train_tokens': 4448356}

515

{'loss': 0.04927523732185364, 'learning_rate': 3.3060648801128354e-06, 'train_speed(iter/s)': 0.435925, 'epoch': 2.057877813504823, 'consumed_train_tokens': 4456666}

516

{'loss': 0.05040984153747559, 'learning_rate': 3.291960507757405e-06, 'train_speed(iter/s)': 0.435981, 'epoch': 2.061897106109325, 'consumed_train_tokens': 4465525}

517

{'loss': 0.05969190001487732, 'learning_rate': 3.277856135401975e-06, 'train_speed(iter/s)': 0.435996, 'epoch': 2.0659163987138265, 'consumed_train_tokens': 4473989}

518

{'loss': 0.0642296850681305, 'learning_rate': 3.263751763046545e-06, 'train_speed(iter/s)': 0.436054, 'epoch': 2.069935691318328, 'consumed_train_tokens': 4483360}

519

{'loss': 0.06926605701446534, 'learning_rate': 3.249647390691115e-06, 'train_speed(iter/s)': 0.436068, 'epoch': 2.0739549839228295, 'consumed_train_tokens': 4491882}

520

{'loss': 0.08104807138442993, 'learning_rate': 3.2355430183356844e-06, 'train_speed(iter/s)': 0.436071, 'epoch': 2.077974276527331, 'consumed_train_tokens': 4500790}

521

{'loss': 0.08176295757293701, 'learning_rate': 3.2214386459802544e-06, 'train_speed(iter/s)': 0.43612, 'epoch': 2.081993569131833, 'consumed_train_tokens': 4510121}

522

{'loss': 0.05891358256340027, 'learning_rate': 3.2073342736248243e-06, 'train_speed(iter/s)': 0.436137, 'epoch': 2.0860128617363345, 'consumed_train_tokens': 4518025}

523

{'loss': 0.06949127912521362, 'learning_rate': 3.193229901269394e-06, 'train_speed(iter/s)': 0.43619, 'epoch': 2.090032154340836, 'consumed_train_tokens': 4527718}

524

{'loss': 0.06979156732559204, 'learning_rate': 3.179125528913964e-06, 'train_speed(iter/s)': 0.436199, 'epoch': 2.0940514469453375, 'consumed_train_tokens': 4536334}

525

{'loss': 0.07283205986022949, 'learning_rate': 3.165021156558533e-06, 'train_speed(iter/s)': 0.436239, 'epoch': 2.098070739549839, 'consumed_train_tokens': 4545070}

526

{'loss': 0.08264817595481873, 'learning_rate': 3.150916784203103e-06, 'train_speed(iter/s)': 0.436264, 'epoch': 2.102090032154341, 'consumed_train_tokens': 4554365}

527

{'loss': 0.05106252431869507, 'learning_rate': 3.136812411847673e-06, 'train_speed(iter/s)': 0.436303, 'epoch': 2.1061093247588425, 'consumed_train_tokens': 4562068}

528

{'loss': 0.07025182247161865, 'learning_rate': 3.122708039492243e-06, 'train_speed(iter/s)': 0.436317, 'epoch': 2.110128617363344, 'consumed_train_tokens': 4570782}

529

{'loss': 0.07543216347694397, 'learning_rate': 3.1086036671368124e-06, 'train_speed(iter/s)': 0.436324, 'epoch': 2.1141479099678455, 'consumed_train_tokens': 4579761}

530

{'loss': 0.060170704126358034, 'learning_rate': 3.0944992947813823e-06, 'train_speed(iter/s)': 0.436343, 'epoch': 2.1181672025723475, 'consumed_train_tokens': 4587940}

531

{'loss': 0.069877028465271, 'learning_rate': 3.0803949224259523e-06, 'train_speed(iter/s)': 0.436337, 'epoch': 2.122186495176849, 'consumed_train_tokens': 4597776}

532

{'loss': 0.07221965789794922, 'learning_rate': 3.066290550070522e-06, 'train_speed(iter/s)': 0.436367, 'epoch': 2.1262057877813505, 'consumed_train_tokens': 4606457}

533

{'loss': 0.058580464124679564, 'learning_rate': 3.052186177715092e-06, 'train_speed(iter/s)': 0.436378, 'epoch': 2.130225080385852, 'consumed_train_tokens': 4615850}

534

{'loss': 0.06267050504684449, 'learning_rate': 3.0380818053596618e-06, 'train_speed(iter/s)': 0.436424, 'epoch': 2.1342443729903535, 'consumed_train_tokens': 4624519}

535

{'loss': 0.0585421085357666, 'learning_rate': 3.0239774330042317e-06, 'train_speed(iter/s)': 0.436441, 'epoch': 2.1382636655948555, 'consumed_train_tokens': 4633246}

536

{'loss': 0.05702952146530151, 'learning_rate': 3.0098730606488013e-06, 'train_speed(iter/s)': 0.436448, 'epoch': 2.142282958199357, 'consumed_train_tokens': 4642499}

537

{'loss': 0.06751812100410462, 'learning_rate': 2.9957686882933712e-06, 'train_speed(iter/s)': 0.436498, 'epoch': 2.1463022508038585, 'consumed_train_tokens': 4651387}

538

{'loss': 0.06194249391555786, 'learning_rate': 2.981664315937941e-06, 'train_speed(iter/s)': 0.436509, 'epoch': 2.15032154340836, 'consumed_train_tokens': 4660153}

539

{'loss': 0.0642240285873413, 'learning_rate': 2.9675599435825107e-06, 'train_speed(iter/s)': 0.436536, 'epoch': 2.154340836012862, 'consumed_train_tokens': 4668725}

540

{'loss': 0.08144440650939941, 'learning_rate': 2.9534555712270807e-06, 'train_speed(iter/s)': 0.436565, 'epoch': 2.1583601286173635, 'consumed_train_tokens': 4677415}

541

{'loss': 0.05841068625450134, 'learning_rate': 2.9393511988716507e-06, 'train_speed(iter/s)': 0.436601, 'epoch': 2.162379421221865, 'consumed_train_tokens': 4685886}

542

{'loss': 0.06620699763298035, 'learning_rate': 2.92524682651622e-06, 'train_speed(iter/s)': 0.436617, 'epoch': 2.1663987138263665, 'consumed_train_tokens': 4694342}

543

{'loss': 0.06480457782745361, 'learning_rate': 2.91114245416079e-06, 'train_speed(iter/s)': 0.436659, 'epoch': 2.170418006430868, 'consumed_train_tokens': 4703051}

544

{'loss': 0.050562113523483276, 'learning_rate': 2.89703808180536e-06, 'train_speed(iter/s)': 0.43668, 'epoch': 2.17443729903537, 'consumed_train_tokens': 4711658}

545

{'loss': 0.06641823053359985, 'learning_rate': 2.88293370944993e-06, 'train_speed(iter/s)': 0.4367, 'epoch': 2.1784565916398715, 'consumed_train_tokens': 4721097}

546

{'loss': 0.049515488743782046, 'learning_rate': 2.8688293370944996e-06, 'train_speed(iter/s)': 0.436736, 'epoch': 2.182475884244373, 'consumed_train_tokens': 4729440}

547

{'loss': 0.06853473782539368, 'learning_rate': 2.8547249647390696e-06, 'train_speed(iter/s)': 0.436754, 'epoch': 2.1864951768488745, 'consumed_train_tokens': 4738273}

548

{'loss': 0.06836003065109253, 'learning_rate': 2.8406205923836396e-06, 'train_speed(iter/s)': 0.436788, 'epoch': 2.190514469453376, 'consumed_train_tokens': 4747050}

549

{'loss': 0.06548181176185608, 'learning_rate': 2.826516220028209e-06, 'train_speed(iter/s)': 0.436791, 'epoch': 2.194533762057878, 'consumed_train_tokens': 4755384}

550

{'loss': 0.05120590925216675, 'learning_rate': 2.8124118476727786e-06, 'train_speed(iter/s)': 0.436826, 'epoch': 2.1985530546623795, 'consumed_train_tokens': 4764333}

551

{'loss': 0.07258702516555786, 'learning_rate': 2.798307475317348e-06, 'train_speed(iter/s)': 0.436835, 'epoch': 2.202572347266881, 'consumed_train_tokens': 4773315}

552

{'loss': 0.08206385970115662, 'learning_rate': 2.784203102961918e-06, 'train_speed(iter/s)': 0.436865, 'epoch': 2.2065916398713825, 'consumed_train_tokens': 4781791}

553

{'loss': 0.0858729600906372, 'learning_rate': 2.770098730606488e-06, 'train_speed(iter/s)': 0.436896, 'epoch': 2.210610932475884, 'consumed_train_tokens': 4790510}

554

{'loss': 0.06899688243865967, 'learning_rate': 2.755994358251058e-06, 'train_speed(iter/s)': 0.436927, 'epoch': 2.214630225080386, 'consumed_train_tokens': 4799863}

555

{'loss': 0.059636902809143064, 'learning_rate': 2.7418899858956276e-06, 'train_speed(iter/s)': 0.436933, 'epoch': 2.2186495176848875, 'consumed_train_tokens': 4808290}

556

{'loss': 0.06018332242965698, 'learning_rate': 2.7277856135401976e-06, 'train_speed(iter/s)': 0.436949, 'epoch': 2.222668810289389, 'consumed_train_tokens': 4816439}

557

{'loss': 0.07517130374908447, 'learning_rate': 2.7136812411847675e-06, 'train_speed(iter/s)': 0.43699, 'epoch': 2.2266881028938905, 'consumed_train_tokens': 4825018}

558

{'loss': 0.05229204297065735, 'learning_rate': 2.699576868829337e-06, 'train_speed(iter/s)': 0.436985, 'epoch': 2.2307073954983925, 'consumed_train_tokens': 4832821}

559

{'loss': 0.08674585223197936, 'learning_rate': 2.685472496473907e-06, 'train_speed(iter/s)': 0.437012, 'epoch': 2.234726688102894, 'consumed_train_tokens': 4842197}

560

{'loss': 0.0784593939781189, 'learning_rate': 2.671368124118477e-06, 'train_speed(iter/s)': 0.437048, 'epoch': 2.2387459807073955, 'consumed_train_tokens': 4850406}

561

{'loss': 0.05940561890602112, 'learning_rate': 2.657263751763047e-06, 'train_speed(iter/s)': 0.437074, 'epoch': 2.242765273311897, 'consumed_train_tokens': 4860069}

562

{'loss': 0.05572831630706787, 'learning_rate': 2.6431593794076165e-06, 'train_speed(iter/s)': 0.437099, 'epoch': 2.2467845659163985, 'consumed_train_tokens': 4869372}

563

{'loss': 0.06498958468437195, 'learning_rate': 2.6290550070521864e-06, 'train_speed(iter/s)': 0.437128, 'epoch': 2.2508038585209005, 'consumed_train_tokens': 4878590}

564

{'loss': 0.05210442543029785, 'learning_rate': 2.6149506346967564e-06, 'train_speed(iter/s)': 0.437156, 'epoch': 2.254823151125402, 'consumed_train_tokens': 4887276}

565

{'loss': 0.09384630918502808, 'learning_rate': 2.600846262341326e-06, 'train_speed(iter/s)': 0.437165, 'epoch': 2.2588424437299035, 'consumed_train_tokens': 4896308}

566

{'loss': 0.07336938977241517, 'learning_rate': 2.586741889985896e-06, 'train_speed(iter/s)': 0.437202, 'epoch': 2.262861736334405, 'consumed_train_tokens': 4904986}

567

{'loss': 0.05656155347824097, 'learning_rate': 2.572637517630466e-06, 'train_speed(iter/s)': 0.437209, 'epoch': 2.266881028938907, 'consumed_train_tokens': 4914151}

568

{'loss': 0.07836430072784424, 'learning_rate': 2.5585331452750354e-06, 'train_speed(iter/s)': 0.437238, 'epoch': 2.2709003215434085, 'consumed_train_tokens': 4922525}

569

{'loss': 0.06591943502426148, 'learning_rate': 2.5444287729196054e-06, 'train_speed(iter/s)': 0.437262, 'epoch': 2.27491961414791, 'consumed_train_tokens': 4931216}

570

{'loss': 0.06307260990142823, 'learning_rate': 2.5303244005641753e-06, 'train_speed(iter/s)': 0.437289, 'epoch': 2.2789389067524115, 'consumed_train_tokens': 4940877}

571

{'loss': 0.06706664562225342, 'learning_rate': 2.5162200282087453e-06, 'train_speed(iter/s)': 0.437307, 'epoch': 2.282958199356913, 'consumed_train_tokens': 4950047}

572

{'loss': 0.06985098123550415, 'learning_rate': 2.502115655853315e-06, 'train_speed(iter/s)': 0.437318, 'epoch': 2.286977491961415, 'consumed_train_tokens': 4958024}

573

{'loss': 0.06064713001251221, 'learning_rate': 2.4880112834978844e-06, 'train_speed(iter/s)': 0.437349, 'epoch': 2.2909967845659165, 'consumed_train_tokens': 4965990}

574

{'loss': 0.09043799638748169, 'learning_rate': 2.4739069111424543e-06, 'train_speed(iter/s)': 0.437373, 'epoch': 2.295016077170418, 'consumed_train_tokens': 4975004}

575

{'loss': 0.07067066431045532, 'learning_rate': 2.4598025387870243e-06, 'train_speed(iter/s)': 0.437403, 'epoch': 2.2990353697749195, 'consumed_train_tokens': 4983614}

576

{'loss': 0.059562861919403076, 'learning_rate': 2.445698166431594e-06, 'train_speed(iter/s)': 0.437432, 'epoch': 2.303054662379421, 'consumed_train_tokens': 4992505}

577

{'loss': 0.061577439308166504, 'learning_rate': 2.431593794076164e-06, 'train_speed(iter/s)': 0.437447, 'epoch': 2.307073954983923, 'consumed_train_tokens': 5002059}

578

{'loss': 0.09321972131729125, 'learning_rate': 2.4174894217207338e-06, 'train_speed(iter/s)': 0.437495, 'epoch': 2.3110932475884245, 'consumed_train_tokens': 5011246}

579

{'loss': 0.08092663884162903, 'learning_rate': 2.3892806770098733e-06, 'train_speed(iter/s)': 0.43756, 'epoch': 2.3191318327974275, 'consumed_train_tokens': 5028419}

580

{'loss': 0.07313514947891235, 'learning_rate': 2.3751763046544432e-06, 'train_speed(iter/s)': 0.43758, 'epoch': 2.323151125401929, 'consumed_train_tokens': 5036520}

581

{'loss': 0.046095409989356996, 'learning_rate': 2.3610719322990128e-06, 'train_speed(iter/s)': 0.437607, 'epoch': 2.327170418006431, 'consumed_train_tokens': 5045339}

582

{'loss': 0.06511886119842529, 'learning_rate': 2.3469675599435827e-06, 'train_speed(iter/s)': 0.437642, 'epoch': 2.3311897106109325, 'consumed_train_tokens': 5054033}

583

{'loss': 0.06839130520820617, 'learning_rate': 2.3328631875881523e-06, 'train_speed(iter/s)': 0.437648, 'epoch': 2.335209003215434, 'consumed_train_tokens': 5062257}

584

{'loss': 0.056369245052337646, 'learning_rate': 2.3187588152327222e-06, 'train_speed(iter/s)': 0.43769, 'epoch': 2.3392282958199355, 'consumed_train_tokens': 5071177}

585

{'loss': 0.0652003526687622, 'learning_rate': 2.304654442877292e-06, 'train_speed(iter/s)': 0.437704, 'epoch': 2.3432475884244375, 'consumed_train_tokens': 5080151}

586

{'loss': 0.07287459373474121, 'learning_rate': 2.290550070521862e-06, 'train_speed(iter/s)': 0.437748, 'epoch': 2.347266881028939, 'consumed_train_tokens': 5089141}

587

{'loss': 0.05998371839523316, 'learning_rate': 2.2764456981664317e-06, 'train_speed(iter/s)': 0.437763, 'epoch': 2.3512861736334405, 'consumed_train_tokens': 5098094}

588

{'loss': 0.059172630310058594, 'learning_rate': 2.2623413258110017e-06, 'train_speed(iter/s)': 0.43779, 'epoch': 2.355305466237942, 'consumed_train_tokens': 5107164}

589

{'loss': 0.06303284168243409, 'learning_rate': 2.2482369534555716e-06, 'train_speed(iter/s)': 0.437816, 'epoch': 2.359324758842444, 'consumed_train_tokens': 5115955}

590

{'loss': 0.05158138871192932, 'learning_rate': 2.234132581100141e-06, 'train_speed(iter/s)': 0.437831, 'epoch': 2.3633440514469455, 'consumed_train_tokens': 5124517}

591

{'loss': 0.04870752096176147, 'learning_rate': 2.220028208744711e-06, 'train_speed(iter/s)': 0.437873, 'epoch': 2.367363344051447, 'consumed_train_tokens': 5133444}

592

{'loss': 0.06328004598617554, 'learning_rate': 2.205923836389281e-06, 'train_speed(iter/s)': 0.437886, 'epoch': 2.3713826366559485, 'consumed_train_tokens': 5143387}

593

{'loss': 0.08563151359558105, 'learning_rate': 2.1918194640338506e-06, 'train_speed(iter/s)': 0.437928, 'epoch': 2.37540192926045, 'consumed_train_tokens': 5152913}

594

{'loss': 0.07174869179725647, 'learning_rate': 2.1777150916784206e-06, 'train_speed(iter/s)': 0.437938, 'epoch': 2.379421221864952, 'consumed_train_tokens': 5161912}

595

{'loss': 0.05723352432250976, 'learning_rate': 2.16361071932299e-06, 'train_speed(iter/s)': 0.43797, 'epoch': 2.3834405144694535, 'consumed_train_tokens': 5170519}

596

{'loss': 0.05061270594596863, 'learning_rate': 2.14950634696756e-06, 'train_speed(iter/s)': 0.437999, 'epoch': 2.387459807073955, 'consumed_train_tokens': 5179619}

597

{'loss': 0.07239852547645569, 'learning_rate': 2.13540197461213e-06, 'train_speed(iter/s)': 0.438, 'epoch': 2.3914790996784565, 'consumed_train_tokens': 5188513}

598

{'loss': 0.05219123363494873, 'learning_rate': 2.1212976022566996e-06, 'train_speed(iter/s)': 0.438046, 'epoch': 2.395498392282958, 'consumed_train_tokens': 5197566}

599

{'loss': 0.06905498504638671, 'learning_rate': 2.1071932299012696e-06, 'train_speed(iter/s)': 0.438058, 'epoch': 2.39951768488746, 'consumed_train_tokens': 5206834}

600

{'loss': 0.061874997615814206, 'learning_rate': 2.0930888575458395e-06, 'train_speed(iter/s)': 0.438102, 'epoch': 2.4035369774919615, 'consumed_train_tokens': 5215050}

601

{'loss': 0.07490845918655395, 'learning_rate': 2.078984485190409e-06, 'train_speed(iter/s)': 0.438118, 'epoch': 2.407556270096463, 'consumed_train_tokens': 5223255}

602

{'loss': 0.07439334988594055, 'learning_rate': 2.064880112834979e-06, 'train_speed(iter/s)': 0.438157, 'epoch': 2.4115755627009645, 'consumed_train_tokens': 5231882}

603

{'loss': 0.061666679382324216, 'learning_rate': 2.050775740479549e-06, 'train_speed(iter/s)': 0.438179, 'epoch': 2.415594855305466, 'consumed_train_tokens': 5240650}

604

{'loss': 0.07559508085250854, 'learning_rate': 2.036671368124119e-06, 'train_speed(iter/s)': 0.438208, 'epoch': 2.419614147909968, 'consumed_train_tokens': 5249658}

605

{'loss': 0.06144961714744568, 'learning_rate': 2.0225669957686885e-06, 'train_speed(iter/s)': 0.438242, 'epoch': 2.4236334405144695, 'consumed_train_tokens': 5258033}

606

{'loss': 0.048069503903388974, 'learning_rate': 2.008462623413258e-06, 'train_speed(iter/s)': 0.438238, 'epoch': 2.427652733118971, 'consumed_train_tokens': 5267023}

607

{'loss': 0.0516661524772644, 'learning_rate': 1.994358251057828e-06, 'train_speed(iter/s)': 0.438278, 'epoch': 2.4316720257234725, 'consumed_train_tokens': 5275267}

608

{'loss': 0.08369715213775634, 'learning_rate': 1.980253878702398e-06, 'train_speed(iter/s)': 0.438292, 'epoch': 2.435691318327974, 'consumed_train_tokens': 5283991}

609

{'loss': 0.09051575660705566, 'learning_rate': 1.9661495063469675e-06, 'train_speed(iter/s)': 0.438323, 'epoch': 2.439710610932476, 'consumed_train_tokens': 5292819}

610

{'loss': 0.05886510610580444, 'learning_rate': 1.9520451339915374e-06, 'train_speed(iter/s)': 0.438339, 'epoch': 2.4437299035369775, 'consumed_train_tokens': 5301059}

611

{'loss': 0.06599385738372802, 'learning_rate': 1.9379407616361074e-06, 'train_speed(iter/s)': 0.438337, 'epoch': 2.447749196141479, 'consumed_train_tokens': 5308669}

612

{'loss': 0.06276222467422485, 'learning_rate': 1.9238363892806774e-06, 'train_speed(iter/s)': 0.438377, 'epoch': 2.4517684887459805, 'consumed_train_tokens': 5317389}

613

{'loss': 0.08723877072334289, 'learning_rate': 1.909732016925247e-06, 'train_speed(iter/s)': 0.438389, 'epoch': 2.4557877813504825, 'consumed_train_tokens': 5326360}

614

{'loss': 0.05338106155395508, 'learning_rate': 1.8956276445698169e-06, 'train_speed(iter/s)': 0.438439, 'epoch': 2.459807073954984, 'consumed_train_tokens': 5335532}

615

{'loss': 0.05907174348831177, 'learning_rate': 1.8815232722143866e-06, 'train_speed(iter/s)': 0.438451, 'epoch': 2.4638263665594855, 'consumed_train_tokens': 5343732}

616

{'loss': 0.053596901893615725, 'learning_rate': 1.8674188998589566e-06, 'train_speed(iter/s)': 0.438501, 'epoch': 2.467845659163987, 'consumed_train_tokens': 5352095}

617

{'loss': 0.06360788345336914, 'learning_rate': 1.8533145275035263e-06, 'train_speed(iter/s)': 0.438506, 'epoch': 2.471864951768489, 'consumed_train_tokens': 5360116}

618

{'loss': 0.06261759400367736, 'learning_rate': 1.8392101551480959e-06, 'train_speed(iter/s)': 0.438536, 'epoch': 2.4758842443729905, 'consumed_train_tokens': 5368865}

619

{'loss': 0.062107205390930176, 'learning_rate': 1.8251057827926658e-06, 'train_speed(iter/s)': 0.43856, 'epoch': 2.479903536977492, 'consumed_train_tokens': 5376932}

620

{'loss': 0.0595166802406311, 'learning_rate': 1.8110014104372356e-06, 'train_speed(iter/s)': 0.438581, 'epoch': 2.4839228295819935, 'consumed_train_tokens': 5385222}

621

{'loss': 0.05744399428367615, 'learning_rate': 1.7968970380818053e-06, 'train_speed(iter/s)': 0.43862, 'epoch': 2.487942122186495, 'consumed_train_tokens': 5393838}

622

{'loss': 0.07258445024490356, 'learning_rate': 1.7827926657263753e-06, 'train_speed(iter/s)': 0.438628, 'epoch': 2.491961414790997, 'consumed_train_tokens': 5402912}

623

{'loss': 0.05456082820892334, 'learning_rate': 1.768688293370945e-06, 'train_speed(iter/s)': 0.438666, 'epoch': 2.4959807073954985, 'consumed_train_tokens': 5411790}

624

{'loss': 0.06201680898666382, 'learning_rate': 1.754583921015515e-06, 'train_speed(iter/s)': 0.438669, 'epoch': 2.5, 'consumed_train_tokens': 5419864}

625

{'loss': 0.05513502955436707, 'learning_rate': 1.7404795486600848e-06, 'train_speed(iter/s)': 0.438704, 'epoch': 2.5040192926045015, 'consumed_train_tokens': 5428978}

626

{'loss': 0.06274003386497498, 'learning_rate': 1.7263751763046547e-06, 'train_speed(iter/s)': 0.438727, 'epoch': 2.508038585209003, 'consumed_train_tokens': 5439128}

627

{'loss': 0.05712069272994995, 'learning_rate': 1.7122708039492245e-06, 'train_speed(iter/s)': 0.438736, 'epoch': 2.512057877813505, 'consumed_train_tokens': 5447046}

628

{'loss': 0.07302256822586059, 'learning_rate': 1.6981664315937942e-06, 'train_speed(iter/s)': 0.438777, 'epoch': 2.5160771704180065, 'consumed_train_tokens': 5456005}

629

{'loss': 0.05360268354415894, 'learning_rate': 1.6840620592383642e-06, 'train_speed(iter/s)': 0.438784, 'epoch': 2.520096463022508, 'consumed_train_tokens': 5464419}

630

{'loss': 0.05975008010864258, 'learning_rate': 1.669957686882934e-06, 'train_speed(iter/s)': 0.438817, 'epoch': 2.5241157556270095, 'consumed_train_tokens': 5472566}

631

{'loss': 0.06385658979415894, 'learning_rate': 1.6558533145275035e-06, 'train_speed(iter/s)': 0.438833, 'epoch': 2.528135048231511, 'consumed_train_tokens': 5480804}

632

{'loss': 0.053632885217666626, 'learning_rate': 1.6417489421720734e-06, 'train_speed(iter/s)': 0.43885, 'epoch': 2.532154340836013, 'consumed_train_tokens': 5489674}

633

{'loss': 0.05162864923477173, 'learning_rate': 1.6276445698166432e-06, 'train_speed(iter/s)': 0.438875, 'epoch': 2.5361736334405145, 'consumed_train_tokens': 5497681}

634

{'loss': 0.08934869766235351, 'learning_rate': 1.613540197461213e-06, 'train_speed(iter/s)': 0.438893, 'epoch': 2.540192926045016, 'consumed_train_tokens': 5506221}

635

{'loss': 0.08354313373565674, 'learning_rate': 1.599435825105783e-06, 'train_speed(iter/s)': 0.438922, 'epoch': 2.5442122186495175, 'consumed_train_tokens': 5515380}

636

{'loss': 0.049110320210456845, 'learning_rate': 1.5853314527503527e-06, 'train_speed(iter/s)': 0.438924, 'epoch': 2.548231511254019, 'consumed_train_tokens': 5524147}

637

{'loss': 0.061899739503860476, 'learning_rate': 1.5712270803949226e-06, 'train_speed(iter/s)': 0.43895, 'epoch': 2.552250803858521, 'consumed_train_tokens': 5532279}

638

{'loss': 0.06500414609909058, 'learning_rate': 1.5571227080394924e-06, 'train_speed(iter/s)': 0.438949, 'epoch': 2.5562700964630225, 'consumed_train_tokens': 5540420}

639

{'loss': 0.04865860342979431, 'learning_rate': 1.5430183356840623e-06, 'train_speed(iter/s)': 0.438978, 'epoch': 2.560289389067524, 'consumed_train_tokens': 5549568}

640

{'loss': 0.05178437232971191, 'learning_rate': 1.528913963328632e-06, 'train_speed(iter/s)': 0.438994, 'epoch': 2.564308681672026, 'consumed_train_tokens': 5558417}

641

{'loss': 0.06258593201637268, 'learning_rate': 1.5148095909732018e-06, 'train_speed(iter/s)': 0.439021, 'epoch': 2.5683279742765275, 'consumed_train_tokens': 5567383}

642

{'loss': 0.043620461225509645, 'learning_rate': 1.5007052186177718e-06, 'train_speed(iter/s)': 0.439047, 'epoch': 2.572347266881029, 'consumed_train_tokens': 5575731}

643

{'loss': 0.08434635400772095, 'learning_rate': 1.4866008462623415e-06, 'train_speed(iter/s)': 0.439052, 'epoch': 2.5763665594855305, 'consumed_train_tokens': 5585170}

644

{'loss': 0.06129905581474304, 'learning_rate': 1.472496473906911e-06, 'train_speed(iter/s)': 0.439088, 'epoch': 2.580385852090032, 'consumed_train_tokens': 5593323}

645

{'loss': 0.054739254713058474, 'learning_rate': 1.458392101551481e-06, 'train_speed(iter/s)': 0.439095, 'epoch': 2.584405144694534, 'consumed_train_tokens': 5601255}

646

{'loss': 0.0691527009010315, 'learning_rate': 1.4442877291960508e-06, 'train_speed(iter/s)': 0.439126, 'epoch': 2.5884244372990355, 'consumed_train_tokens': 5610371}

647

{'loss': 0.09670791625976563, 'learning_rate': 1.4301833568406205e-06, 'train_speed(iter/s)': 0.439137, 'epoch': 2.592443729903537, 'consumed_train_tokens': 5618763}

648

{'loss': 0.05838689208030701, 'learning_rate': 1.4160789844851905e-06, 'train_speed(iter/s)': 0.43916, 'epoch': 2.5964630225080385, 'consumed_train_tokens': 5627420}

649

{'loss': 0.05179818868637085, 'learning_rate': 1.4019746121297603e-06, 'train_speed(iter/s)': 0.439174, 'epoch': 2.60048231511254, 'consumed_train_tokens': 5635727}

650

{'loss': 0.046194452047348025, 'learning_rate': 1.3878702397743302e-06, 'train_speed(iter/s)': 0.439172, 'epoch': 2.604501607717042, 'consumed_train_tokens': 5644641}

651

{'loss': 0.06654124259948731, 'learning_rate': 1.3737658674189e-06, 'train_speed(iter/s)': 0.439212, 'epoch': 2.6085209003215435, 'consumed_train_tokens': 5653361}

652

{'loss': 0.07489185333251953, 'learning_rate': 1.35966149506347e-06, 'train_speed(iter/s)': 0.439217, 'epoch': 2.612540192926045, 'consumed_train_tokens': 5661755}

653

{'loss': 0.04294430017471314, 'learning_rate': 1.3455571227080397e-06, 'train_speed(iter/s)': 0.439253, 'epoch': 2.6165594855305465, 'consumed_train_tokens': 5669596}

654

{'loss': 0.0630152702331543, 'learning_rate': 1.3314527503526094e-06, 'train_speed(iter/s)': 0.439262, 'epoch': 2.620578778135048, 'consumed_train_tokens': 5678547}

655

{'loss': 0.07993980646133422, 'learning_rate': 1.3173483779971794e-06, 'train_speed(iter/s)': 0.439284, 'epoch': 2.62459807073955, 'consumed_train_tokens': 5687710}

656

{'loss': 0.056735682487487796, 'learning_rate': 1.303244005641749e-06, 'train_speed(iter/s)': 0.439305, 'epoch': 2.6286173633440515, 'consumed_train_tokens': 5696141}

657

{'loss': 0.05432428121566772, 'learning_rate': 1.2891396332863187e-06, 'train_speed(iter/s)': 0.439299, 'epoch': 2.632636655948553, 'consumed_train_tokens': 5704297}

658

{'loss': 0.10662949085235596, 'learning_rate': 1.2750352609308887e-06, 'train_speed(iter/s)': 0.439344, 'epoch': 2.6366559485530545, 'consumed_train_tokens': 5712551}

659

{'loss': 0.0694614291191101, 'learning_rate': 1.2609308885754584e-06, 'train_speed(iter/s)': 0.439351, 'epoch': 2.640675241157556, 'consumed_train_tokens': 5720782}

660

{'loss': 0.07518720626831055, 'learning_rate': 1.2468265162200284e-06, 'train_speed(iter/s)': 0.439388, 'epoch': 2.644694533762058, 'consumed_train_tokens': 5730049}

661

{'loss': 0.07686498761177063, 'learning_rate': 1.2327221438645981e-06, 'train_speed(iter/s)': 0.439396, 'epoch': 2.6487138263665595, 'consumed_train_tokens': 5738834}

662

{'loss': 0.059003806114196776, 'learning_rate': 1.2186177715091679e-06, 'train_speed(iter/s)': 0.439418, 'epoch': 2.652733118971061, 'consumed_train_tokens': 5747354}

663

{'loss': 0.06810548305511474, 'learning_rate': 1.2045133991537378e-06, 'train_speed(iter/s)': 0.439436, 'epoch': 2.656752411575563, 'consumed_train_tokens': 5756278}

664

{'loss': 0.06344023942947388, 'learning_rate': 1.1904090267983076e-06, 'train_speed(iter/s)': 0.439458, 'epoch': 2.660771704180064, 'consumed_train_tokens': 5765618}

665

{'loss': 0.07014458179473877, 'learning_rate': 1.1763046544428775e-06, 'train_speed(iter/s)': 0.439475, 'epoch': 2.664790996784566, 'consumed_train_tokens': 5774987}

666

{'loss': 0.06347382068634033, 'learning_rate': 1.162200282087447e-06, 'train_speed(iter/s)': 0.439481, 'epoch': 2.6688102893890675, 'consumed_train_tokens': 5783519}

667

{'loss': 0.07068905830383301, 'learning_rate': 1.148095909732017e-06, 'train_speed(iter/s)': 0.439521, 'epoch': 2.672829581993569, 'consumed_train_tokens': 5791977}

668

{'loss': 0.0780958116054535, 'learning_rate': 1.1339915373765868e-06, 'train_speed(iter/s)': 0.439524, 'epoch': 2.676848874598071, 'consumed_train_tokens': 5801295}

669

{'loss': 0.08398705720901489, 'learning_rate': 1.1198871650211568e-06, 'train_speed(iter/s)': 0.439545, 'epoch': 2.6808681672025725, 'consumed_train_tokens': 5810000}

670

{'loss': 0.044574037194252014, 'learning_rate': 1.1057827926657265e-06, 'train_speed(iter/s)': 0.439554, 'epoch': 2.684887459807074, 'consumed_train_tokens': 5818664}

671

{'loss': 0.055643731355667116, 'learning_rate': 1.0916784203102963e-06, 'train_speed(iter/s)': 0.439563, 'epoch': 2.6889067524115755, 'consumed_train_tokens': 5826864}

672

{'loss': 0.10051629543304444, 'learning_rate': 1.077574047954866e-06, 'train_speed(iter/s)': 0.43959, 'epoch': 2.692926045016077, 'consumed_train_tokens': 5835849}

673

{'loss': 0.057640540599823, 'learning_rate': 1.063469675599436e-06, 'train_speed(iter/s)': 0.439601, 'epoch': 2.696945337620579, 'consumed_train_tokens': 5844753}

674

{'loss': 0.04457935690879822, 'learning_rate': 1.0493653032440057e-06, 'train_speed(iter/s)': 0.439632, 'epoch': 2.7009646302250805, 'consumed_train_tokens': 5853540}

675

{'loss': 0.07604471445083619, 'learning_rate': 1.0352609308885755e-06, 'train_speed(iter/s)': 0.439627, 'epoch': 2.704983922829582, 'consumed_train_tokens': 5861472}

676

{'loss': 0.05228697657585144, 'learning_rate': 1.0211565585331454e-06, 'train_speed(iter/s)': 0.439644, 'epoch': 2.7090032154340835, 'consumed_train_tokens': 5869790}

677

{'loss': 0.10240916013717652, 'learning_rate': 1.0070521861777152e-06, 'train_speed(iter/s)': 0.439669, 'epoch': 2.713022508038585, 'consumed_train_tokens': 5879315}

678

{'loss': 0.061826282739639284, 'learning_rate': 9.929478138222851e-07, 'train_speed(iter/s)': 0.439699, 'epoch': 2.717041800643087, 'consumed_train_tokens': 5888374}

679

{'loss': 0.051966017484664916, 'learning_rate': 9.788434414668547e-07, 'train_speed(iter/s)': 0.439704, 'epoch': 2.7210610932475885, 'consumed_train_tokens': 5896627}

680

{'loss': 0.03900730609893799, 'learning_rate': 9.506346967559944e-07, 'train_speed(iter/s)': 0.439741, 'epoch': 2.7290996784565915, 'consumed_train_tokens': 5914179}

681

{'loss': 0.053106778860092164, 'learning_rate': 9.365303244005643e-07, 'train_speed(iter/s)': 0.439745, 'epoch': 2.733118971061093, 'consumed_train_tokens': 5923157}

682

{'loss': 0.04411388635635376, 'learning_rate': 9.224259520451341e-07, 'train_speed(iter/s)': 0.439784, 'epoch': 2.737138263665595, 'consumed_train_tokens': 5932121}

683

{'loss': 0.044888424873352054, 'learning_rate': 9.08321579689704e-07, 'train_speed(iter/s)': 0.439801, 'epoch': 2.7411575562700965, 'consumed_train_tokens': 5940867}

684

{'loss': 0.04689985513687134, 'learning_rate': 8.942172073342736e-07, 'train_speed(iter/s)': 0.439827, 'epoch': 2.745176848874598, 'consumed_train_tokens': 5949415}

685

{'loss': 0.07756737470626832, 'learning_rate': 8.801128349788435e-07, 'train_speed(iter/s)': 0.439842, 'epoch': 2.7491961414790995, 'consumed_train_tokens': 5958458}

686

{'loss': 0.0667335033416748, 'learning_rate': 8.660084626234133e-07, 'train_speed(iter/s)': 0.439854, 'epoch': 2.753215434083601, 'consumed_train_tokens': 5967255}

687

{'loss': 0.059124648571014404, 'learning_rate': 8.519040902679832e-07, 'train_speed(iter/s)': 0.439867, 'epoch': 2.757234726688103, 'consumed_train_tokens': 5975839}

688

{'loss': 0.048493200540542604, 'learning_rate': 8.37799717912553e-07, 'train_speed(iter/s)': 0.439878, 'epoch': 2.7612540192926045, 'consumed_train_tokens': 5984803}

689

{'loss': 0.044862383604049684, 'learning_rate': 8.236953455571228e-07, 'train_speed(iter/s)': 0.439915, 'epoch': 2.765273311897106, 'consumed_train_tokens': 5993339}

690

{'loss': 0.07854683995246887, 'learning_rate': 8.095909732016925e-07, 'train_speed(iter/s)': 0.43991, 'epoch': 2.769292604501608, 'consumed_train_tokens': 6002807}

691

{'loss': 0.0714066505432129, 'learning_rate': 7.954866008462624e-07, 'train_speed(iter/s)': 0.439908, 'epoch': 2.7733118971061095, 'consumed_train_tokens': 6011374}

692

{'loss': 0.05061043500900268, 'learning_rate': 7.813822284908321e-07, 'train_speed(iter/s)': 0.439927, 'epoch': 2.777331189710611, 'consumed_train_tokens': 6020365}

693

{'loss': 0.042950639128685, 'learning_rate': 7.67277856135402e-07, 'train_speed(iter/s)': 0.43995, 'epoch': 2.7813504823151125, 'consumed_train_tokens': 6028713}

694

{'loss': 0.05055454969406128, 'learning_rate': 7.531734837799719e-07, 'train_speed(iter/s)': 0.439952, 'epoch': 2.785369774919614, 'consumed_train_tokens': 6037598}

695

{'loss': 0.08369446992874145, 'learning_rate': 7.390691114245417e-07, 'train_speed(iter/s)': 0.439949, 'epoch': 2.789389067524116, 'consumed_train_tokens': 6046102}

696

{'loss': 0.07128422856330871, 'learning_rate': 7.249647390691116e-07, 'train_speed(iter/s)': 0.43998, 'epoch': 2.7934083601286175, 'consumed_train_tokens': 6054578}

697

{'loss': 0.06230741739273071, 'learning_rate': 7.108603667136812e-07, 'train_speed(iter/s)': 0.439987, 'epoch': 2.797427652733119, 'consumed_train_tokens': 6063469}

698

{'loss': 0.06868499517440796, 'learning_rate': 6.967559943582511e-07, 'train_speed(iter/s)': 0.44001, 'epoch': 2.8014469453376205, 'consumed_train_tokens': 6071939}

699

{'loss': 0.07712815999984741, 'learning_rate': 6.826516220028209e-07, 'train_speed(iter/s)': 0.440026, 'epoch': 2.805466237942122, 'consumed_train_tokens': 6079846}

700

{'loss': 0.0948693573474884, 'learning_rate': 6.685472496473908e-07, 'train_speed(iter/s)': 0.440046, 'epoch': 2.809485530546624, 'consumed_train_tokens': 6088107}

701

{'loss': 0.06791812181472778, 'learning_rate': 6.544428772919606e-07, 'train_speed(iter/s)': 0.44006, 'epoch': 2.8135048231511255, 'consumed_train_tokens': 6096995}

702

{'loss': 0.07176650762557983, 'learning_rate': 6.403385049365304e-07, 'train_speed(iter/s)': 0.440066, 'epoch': 2.817524115755627, 'consumed_train_tokens': 6104910}

703

{'loss': 0.06150472164154053, 'learning_rate': 6.262341325811001e-07, 'train_speed(iter/s)': 0.440093, 'epoch': 2.8215434083601285, 'consumed_train_tokens': 6114015}

704

{'loss': 0.03985375165939331, 'learning_rate': 6.1212976022567e-07, 'train_speed(iter/s)': 0.440094, 'epoch': 2.82556270096463, 'consumed_train_tokens': 6122848}

705

{'loss': 0.05276808738708496, 'learning_rate': 5.980253878702399e-07, 'train_speed(iter/s)': 0.440117, 'epoch': 2.829581993569132, 'consumed_train_tokens': 6131964}

706

{'loss': 0.08538354635238647, 'learning_rate': 5.839210155148096e-07, 'train_speed(iter/s)': 0.440128, 'epoch': 2.8336012861736335, 'consumed_train_tokens': 6140189}

707

{'loss': 0.1083641767501831, 'learning_rate': 5.698166431593795e-07, 'train_speed(iter/s)': 0.440139, 'epoch': 2.837620578778135, 'consumed_train_tokens': 6148682}

708

{'loss': 0.07285487651824951, 'learning_rate': 5.557122708039492e-07, 'train_speed(iter/s)': 0.440139, 'epoch': 2.8416398713826365, 'consumed_train_tokens': 6156739}

709

{'loss': 0.05680620074272156, 'learning_rate': 5.416078984485191e-07, 'train_speed(iter/s)': 0.440157, 'epoch': 2.845659163987138, 'consumed_train_tokens': 6164943}

710

{'loss': 0.04773781001567841, 'learning_rate': 5.275035260930889e-07, 'train_speed(iter/s)': 0.440173, 'epoch': 2.84967845659164, 'consumed_train_tokens': 6173541}

711

{'loss': 0.08317464590072632, 'learning_rate': 5.133991537376587e-07, 'train_speed(iter/s)': 0.440181, 'epoch': 2.8536977491961415, 'consumed_train_tokens': 6182452}

712

{'loss': 0.08879366517066956, 'learning_rate': 4.992947813822285e-07, 'train_speed(iter/s)': 0.440188, 'epoch': 2.857717041800643, 'consumed_train_tokens': 6191047}

713

{'loss': 0.0630942702293396, 'learning_rate': 4.851904090267984e-07, 'train_speed(iter/s)': 0.440204, 'epoch': 2.861736334405145, 'consumed_train_tokens': 6199757}

714

{'loss': 0.0929962158203125, 'learning_rate': 4.7108603667136815e-07, 'train_speed(iter/s)': 0.440223, 'epoch': 2.865755627009646, 'consumed_train_tokens': 6208828}

715

{'loss': 0.04382079839706421, 'learning_rate': 4.5698166431593795e-07, 'train_speed(iter/s)': 0.440236, 'epoch': 2.869774919614148, 'consumed_train_tokens': 6216971}

716

{'loss': 0.08122391700744629, 'learning_rate': 4.428772919605078e-07, 'train_speed(iter/s)': 0.440248, 'epoch': 2.8737942122186495, 'consumed_train_tokens': 6226045}

717

{'loss': 0.047824299335479735, 'learning_rate': 4.2877291960507756e-07, 'train_speed(iter/s)': 0.440261, 'epoch': 2.877813504823151, 'consumed_train_tokens': 6233854}

718

{'loss': 0.0409339964389801, 'learning_rate': 4.146685472496474e-07, 'train_speed(iter/s)': 0.440266, 'epoch': 2.881832797427653, 'consumed_train_tokens': 6241842}

719

{'loss': 0.06320428848266602, 'learning_rate': 4.0056417489421727e-07, 'train_speed(iter/s)': 0.440296, 'epoch': 2.8858520900321545, 'consumed_train_tokens': 6250407}

720

{'loss': 0.040923279523849485, 'learning_rate': 3.86459802538787e-07, 'train_speed(iter/s)': 0.440305, 'epoch': 2.889871382636656, 'consumed_train_tokens': 6258216}

721

{'loss': 0.10007698535919189, 'learning_rate': 3.723554301833569e-07, 'train_speed(iter/s)': 0.440324, 'epoch': 2.8938906752411575, 'consumed_train_tokens': 6266986}

722

{'loss': 0.06672428250312805, 'learning_rate': 3.582510578279267e-07, 'train_speed(iter/s)': 0.440345, 'epoch': 2.897909967845659, 'consumed_train_tokens': 6275702}

723

{'loss': 0.05475064516067505, 'learning_rate': 3.4414668547249654e-07, 'train_speed(iter/s)': 0.440363, 'epoch': 2.901929260450161, 'consumed_train_tokens': 6284238}

724

{'loss': 0.04948564171791077, 'learning_rate': 3.300423131170663e-07, 'train_speed(iter/s)': 0.440374, 'epoch': 2.9059485530546625, 'consumed_train_tokens': 6292823}

725

{'loss': 0.06773123145103455, 'learning_rate': 3.1593794076163614e-07, 'train_speed(iter/s)': 0.440377, 'epoch': 2.909967845659164, 'consumed_train_tokens': 6301517}

726

{'loss': 0.0640515923500061, 'learning_rate': 3.0183356840620595e-07, 'train_speed(iter/s)': 0.44041, 'epoch': 2.9139871382636655, 'consumed_train_tokens': 6309930}

727

{'loss': 0.06664483547210694, 'learning_rate': 2.8772919605077575e-07, 'train_speed(iter/s)': 0.440416, 'epoch': 2.918006430868167, 'consumed_train_tokens': 6318149}

728

{'loss': 0.06432173252105713, 'learning_rate': 2.736248236953456e-07, 'train_speed(iter/s)': 0.440435, 'epoch': 2.922025723472669, 'consumed_train_tokens': 6327450}

729

{'loss': 0.05932891368865967, 'learning_rate': 2.595204513399154e-07, 'train_speed(iter/s)': 0.44046, 'epoch': 2.9260450160771705, 'consumed_train_tokens': 6336241}

730

{'loss': 0.05092281103134155, 'learning_rate': 2.454160789844852e-07, 'train_speed(iter/s)': 0.440473, 'epoch': 2.930064308681672, 'consumed_train_tokens': 6344448}

731

{'loss': 0.07350825071334839, 'learning_rate': 2.3131170662905502e-07, 'train_speed(iter/s)': 0.440498, 'epoch': 2.9340836012861735, 'consumed_train_tokens': 6353707}

732

{'loss': 0.052234286069869997, 'learning_rate': 2.1720733427362482e-07, 'train_speed(iter/s)': 0.440526, 'epoch': 2.938102893890675, 'consumed_train_tokens': 6362658}

733

{'loss': 0.06689174175262451, 'learning_rate': 2.0310296191819468e-07, 'train_speed(iter/s)': 0.440545, 'epoch': 2.942122186495177, 'consumed_train_tokens': 6371594}

734

{'loss': 0.04810342788696289, 'learning_rate': 1.8899858956276448e-07, 'train_speed(iter/s)': 0.440552, 'epoch': 2.9461414790996785, 'consumed_train_tokens': 6380298}

735

{'loss': 0.06401904821395873, 'learning_rate': 1.7489421720733426e-07, 'train_speed(iter/s)': 0.440569, 'epoch': 2.95016077170418, 'consumed_train_tokens': 6389120}

736

{'loss': 0.07073561549186706, 'learning_rate': 1.6078984485190412e-07, 'train_speed(iter/s)': 0.440591, 'epoch': 2.9541800643086815, 'consumed_train_tokens': 6397498}

737

{'loss': 0.06943507194519043, 'learning_rate': 1.4668547249647392e-07, 'train_speed(iter/s)': 0.440601, 'epoch': 2.958199356913183, 'consumed_train_tokens': 6405485}

738

{'loss': 0.06941337585449218, 'learning_rate': 1.3258110014104372e-07, 'train_speed(iter/s)': 0.440611, 'epoch': 2.962218649517685, 'consumed_train_tokens': 6413829}

739

{'loss': 0.04490012526512146, 'learning_rate': 1.1847672778561355e-07, 'train_speed(iter/s)': 0.440617, 'epoch': 2.9662379421221865, 'consumed_train_tokens': 6422537}

740

{'loss': 0.0651373028755188, 'learning_rate': 1.0437235543018337e-07, 'train_speed(iter/s)': 0.440638, 'epoch': 2.970257234726688, 'consumed_train_tokens': 6431841}

741

{'loss': 0.04604911208152771, 'learning_rate': 9.026798307475317e-08, 'train_speed(iter/s)': 0.440628, 'epoch': 2.97427652733119, 'consumed_train_tokens': 6440244}

742

{'loss': 0.04377776384353638, 'learning_rate': 7.616361071932299e-08, 'train_speed(iter/s)': 0.440655, 'epoch': 2.9782958199356915, 'consumed_train_tokens': 6449168}

743

{'loss': 0.07179566621780395, 'learning_rate': 6.205923836389282e-08, 'train_speed(iter/s)': 0.44066, 'epoch': 2.982315112540193, 'consumed_train_tokens': 6458008}

744

{'loss': 0.04854081571102142, 'learning_rate': 4.7954866008462625e-08, 'train_speed(iter/s)': 0.440678, 'epoch': 2.9863344051446945, 'consumed_train_tokens': 6466713}

745

{'loss': 0.031360960006713866, 'learning_rate': 3.385049365303244e-08, 'train_speed(iter/s)': 0.440676, 'epoch': 2.990353697749196, 'consumed_train_tokens': 6474813}

746

{'loss': 0.0711132287979126, 'learning_rate': 1.974612129760226e-08, 'train_speed(iter/s)': 0.440685, 'epoch': 2.994372990353698, 'consumed_train_tokens': 6483640}

747

{'loss': 0.07324442267417908, 'learning_rate': 5.6417489421720734e-09, 'train_speed(iter/s)': 0.440708, 'epoch': 2.9983922829581995, 'consumed_train_tokens': 6492131}

748

{'eval_loss': 1.110217571258545, 'eval_runtime': 35.5817, 'eval_samples_per_second': 28.104, 'eval_steps_per_second': 3.513, 'epoch': 3.0}

749

{'eval_loss': 1.110217571258545, 'eval_runtime': 35.8177, 'eval_samples_per_second': 27.919, 'eval_steps_per_second': 3.49, 'epoch': 3.0}

750

2025-10-03 18:44:14,096 - INFO - fine-tuned output got, start to transfer it for inference

751

use checkpoint-3732 as final checkpoint

752

2025-10-03 19:33:02,421 - INFO - transfer for inference succeeded, start to deliver it for inference

753

2025-10-03 19:43:38,365 - INFO - start to save checkpoint

754

2025-10-03 20:21:33,259 - INFO - finetune-job succeeded

755

2025-10-03 20:21:33,817 - INFO - ##FT_COMPLETE##

756

2025-10-03 20:21:33,804 - INFO - training usage 6492131
