# Supplementary material for: Textual overlap rather than domain alignment: A comparative study of fine-tuning strategies for specialised machine translation with large language models
Source: PLoS One. 2026 Jul 20;21(7):e0352256. doi: 10.1371/journal.pone.0352256 (PMC13384323; doi:10.1371/journal.pone.0352256)
Supplement: S3 File — (ZIP) [file pone.0352256.s003.zip › peft_log.docx]

1

2025-10-05 20:10:03,617 - INFO - data process succeeded, start to fine-tune

2

2025-10-05 20:12:09,627 - INFO - data process succeeded, start to fine-tune

3

Fine-tune started

4

dataset info: {'train_dataset': {'total': 2165483, 'mean': 108.7854415754044, 'std': 48.783743636290694, 'min': 37, 'max': 454, 'size': 19906}, 'val_dataset': {'total': 107175, 'mean': 107.175, 'std': 45.28253940538229, 'min': 40, 'max': 341, 'size': 1000}, 'final_dataset': {'total': 2165483, 'mean': 108.7854415754044, 'std': 48.783743636290694, 'min': 37, 'max': 454, 'size': 19906}, 'total_token': 2165483}

5

{'loss': 3.46223783493042, 'learning_rate': 1.6042780748663098e-06, 'train_speed(iter/s)': 0.197146, 'epoch': 0.0008038585209003215, 'consumed_train_tokens': 1461}

6

{'loss': 2.583024501800537, 'learning_rate': 8.02139037433155e-06, 'train_speed(iter/s)': 0.398164, 'epoch': 0.0040192926045016075, 'consumed_train_tokens': 8508}

7

{'loss': 2.8800228118896483, 'learning_rate': 1.60427807486631e-05, 'train_speed(iter/s)': 0.447376, 'epoch': 0.008038585209003215, 'consumed_train_tokens': 16399}

8

{'loss': 2.61236457824707, 'learning_rate': 2.4064171122994652e-05, 'train_speed(iter/s)': 0.480803, 'epoch': 0.012057877813504822, 'consumed_train_tokens': 24359}

9

{'loss': 2.342015838623047, 'learning_rate': 3.20855614973262e-05, 'train_speed(iter/s)': 0.493812, 'epoch': 0.01607717041800643, 'consumed_train_tokens': 33628}

10

{'loss': 1.7556827545166016, 'learning_rate': 4.0106951871657754e-05, 'train_speed(iter/s)': 0.50627, 'epoch': 0.02009646302250804, 'consumed_train_tokens': 41828}

11

{'loss': 1.4946035385131835, 'learning_rate': 4.8128342245989304e-05, 'train_speed(iter/s)': 0.514663, 'epoch': 0.024115755627009645, 'consumed_train_tokens': 50133}

12

{'loss': 1.2020875930786132, 'learning_rate': 5.614973262032085e-05, 'train_speed(iter/s)': 0.523782, 'epoch': 0.028135048231511254, 'consumed_train_tokens': 58386}

13

{'loss': 0.9851716995239258, 'learning_rate': 6.41711229946524e-05, 'train_speed(iter/s)': 0.530238, 'epoch': 0.03215434083601286, 'consumed_train_tokens': 67211}

14

{'loss': 1.0191627502441407, 'learning_rate': 7.219251336898395e-05, 'train_speed(iter/s)': 0.532992, 'epoch': 0.03617363344051447, 'consumed_train_tokens': 75888}

15

{'loss': 1.0328993797302246, 'learning_rate': 8.021390374331551e-05, 'train_speed(iter/s)': 0.53353, 'epoch': 0.04019292604501608, 'consumed_train_tokens': 84086}

16

{'loss': 0.9527557373046875, 'learning_rate': 8.823529411764705e-05, 'train_speed(iter/s)': 0.533328, 'epoch': 0.04421221864951769, 'consumed_train_tokens': 92572}

17

{'loss': 0.8752375602722168, 'learning_rate': 9.625668449197861e-05, 'train_speed(iter/s)': 0.534084, 'epoch': 0.04823151125401929, 'consumed_train_tokens': 100965}

18

{'loss': 0.9681278228759765, 'learning_rate': 0.00010427807486631015, 'train_speed(iter/s)': 0.533832, 'epoch': 0.0522508038585209, 'consumed_train_tokens': 109756}

19

{'loss': 0.9142363548278809, 'learning_rate': 0.0001122994652406417, 'train_speed(iter/s)': 0.535049, 'epoch': 0.05627009646302251, 'consumed_train_tokens': 118242}

20

{'loss': 0.9582575798034668, 'learning_rate': 0.00012032085561497325, 'train_speed(iter/s)': 0.533606, 'epoch': 0.06028938906752412, 'consumed_train_tokens': 126815}

21

{'loss': 0.9647796630859375, 'learning_rate': 0.0001283422459893048, 'train_speed(iter/s)': 0.53434, 'epoch': 0.06430868167202572, 'consumed_train_tokens': 136295}

22

{'loss': 0.9586438179016114, 'learning_rate': 0.00013636363636363634, 'train_speed(iter/s)': 0.534401, 'epoch': 0.06832797427652733, 'consumed_train_tokens': 145060}

23

{'loss': 0.8901839256286621, 'learning_rate': 0.0001443850267379679, 'train_speed(iter/s)': 0.534938, 'epoch': 0.07234726688102894, 'consumed_train_tokens': 153298}

24

{'loss': 0.8605156898498535, 'learning_rate': 0.00015240641711229946, 'train_speed(iter/s)': 0.534339, 'epoch': 0.07636655948553055, 'consumed_train_tokens': 162019}

25

{'loss': 0.978880500793457, 'learning_rate': 0.00016042780748663101, 'train_speed(iter/s)': 0.534718, 'epoch': 0.08038585209003216, 'consumed_train_tokens': 170726}

26

{'loss': 0.9455799102783203, 'learning_rate': 0.00016844919786096257, 'train_speed(iter/s)': 0.534443, 'epoch': 0.08440514469453377, 'consumed_train_tokens': 178606}

27

{'loss': 0.9219223976135253, 'learning_rate': 0.0001764705882352941, 'train_speed(iter/s)': 0.535616, 'epoch': 0.08842443729903537, 'consumed_train_tokens': 186664}

28

{'loss': 0.8492271423339843, 'learning_rate': 0.00018449197860962566, 'train_speed(iter/s)': 0.535913, 'epoch': 0.09244372990353698, 'consumed_train_tokens': 195727}

29

{'loss': 0.9291240692138671, 'learning_rate': 0.00019251336898395722, 'train_speed(iter/s)': 0.537396, 'epoch': 0.09646302250803858, 'consumed_train_tokens': 203948}

30

{'loss': 0.8515830993652344, 'learning_rate': 0.00020053475935828875, 'train_speed(iter/s)': 0.537826, 'epoch': 0.10048231511254019, 'consumed_train_tokens': 212064}

31

{'loss': 0.9072285652160644, 'learning_rate': 0.0002085561497326203, 'train_speed(iter/s)': 0.538501, 'epoch': 0.1045016077170418, 'consumed_train_tokens': 220825}

32

{'loss': 0.9875930786132813, 'learning_rate': 0.00021657754010695186, 'train_speed(iter/s)': 0.538414, 'epoch': 0.1085209003215434, 'consumed_train_tokens': 229913}

33

{'loss': 0.840213394165039, 'learning_rate': 0.0002245989304812834, 'train_speed(iter/s)': 0.539535, 'epoch': 0.11254019292604502, 'consumed_train_tokens': 239018}

34

{'loss': 0.8775981903076172, 'learning_rate': 0.00023262032085561495, 'train_speed(iter/s)': 0.539816, 'epoch': 0.11655948553054662, 'consumed_train_tokens': 247234}

35

{'loss': 0.8874841690063476, 'learning_rate': 0.0002406417112299465, 'train_speed(iter/s)': 0.541241, 'epoch': 0.12057877813504823, 'consumed_train_tokens': 255950}

36

{'loss': 0.9144456863403321, 'learning_rate': 0.00024866310160427804, 'train_speed(iter/s)': 0.54143, 'epoch': 0.12459807073954984, 'consumed_train_tokens': 264342}

37

{'loss': 0.8050756454467773, 'learning_rate': 0.0002566844919786096, 'train_speed(iter/s)': 0.542422, 'epoch': 0.12861736334405144, 'consumed_train_tokens': 272778}

38

{'loss': 0.878603744506836, 'learning_rate': 0.00026470588235294115, 'train_speed(iter/s)': 0.541565, 'epoch': 0.13263665594855306, 'consumed_train_tokens': 282094}

39

{'loss': 0.8619194984436035, 'learning_rate': 0.0002727272727272727, 'train_speed(iter/s)': 0.541947, 'epoch': 0.13665594855305466, 'consumed_train_tokens': 291443}

40

{'loss': 0.7770240783691407, 'learning_rate': 0.00028074866310160427, 'train_speed(iter/s)': 0.541793, 'epoch': 0.14067524115755628, 'consumed_train_tokens': 300137}

41

{'loss': 0.8842171669006348, 'learning_rate': 0.0002887700534759358, 'train_speed(iter/s)': 0.542499, 'epoch': 0.14469453376205788, 'consumed_train_tokens': 308386}

42

{'loss': 0.8611980438232422, 'learning_rate': 0.00029679144385026733, 'train_speed(iter/s)': 0.54214, 'epoch': 0.1487138263665595, 'consumed_train_tokens': 316658}

43

{'loss': 0.8434852600097656, 'learning_rate': 0.0002997461212976022, 'train_speed(iter/s)': 0.542735, 'epoch': 0.1527331189710611, 'consumed_train_tokens': 324607}

44

{'loss': 0.8310667037963867, 'learning_rate': 0.00029932299012693935, 'train_speed(iter/s)': 0.542531, 'epoch': 0.1567524115755627, 'consumed_train_tokens': 332994}

45

{'loss': 0.820497703552246, 'learning_rate': 0.0002988998589562764, 'train_speed(iter/s)': 0.542294, 'epoch': 0.1607717041800643, 'consumed_train_tokens': 341638}

46

{'loss': 0.8230880737304688, 'learning_rate': 0.0002984767277856135, 'train_speed(iter/s)': 0.54221, 'epoch': 0.1647909967845659, 'consumed_train_tokens': 350053}

47

{'loss': 0.8190693855285645, 'learning_rate': 0.00029805359661495064, 'train_speed(iter/s)': 0.542054, 'epoch': 0.16881028938906753, 'consumed_train_tokens': 358955}

48

{'loss': 0.8815147399902343, 'learning_rate': 0.0002976304654442877, 'train_speed(iter/s)': 0.542105, 'epoch': 0.17282958199356913, 'consumed_train_tokens': 367497}

49

{'loss': 0.7968790054321289, 'learning_rate': 0.0002972073342736248, 'train_speed(iter/s)': 0.542199, 'epoch': 0.17684887459807075, 'consumed_train_tokens': 376391}

50

{'loss': 0.8184366226196289, 'learning_rate': 0.0002967842031029619, 'train_speed(iter/s)': 0.541934, 'epoch': 0.18086816720257234, 'consumed_train_tokens': 384351}

51

{'loss': 0.7547572612762451, 'learning_rate': 0.000296361071932299, 'train_speed(iter/s)': 0.541065, 'epoch': 0.18488745980707397, 'consumed_train_tokens': 392931}

52

{'loss': 0.8404088973999023, 'learning_rate': 0.0002959379407616361, 'train_speed(iter/s)': 0.540796, 'epoch': 0.18890675241157556, 'consumed_train_tokens': 401319}

53

{'loss': 0.8415028572082519, 'learning_rate': 0.0002955148095909732, 'train_speed(iter/s)': 0.54049, 'epoch': 0.19292604501607716, 'consumed_train_tokens': 409803}

54

{'loss': 0.8554150581359863, 'learning_rate': 0.0002950916784203103, 'train_speed(iter/s)': 0.540253, 'epoch': 0.19694533762057878, 'consumed_train_tokens': 419139}

55

{'loss': 0.8583257675170899, 'learning_rate': 0.0002946685472496474, 'train_speed(iter/s)': 0.540235, 'epoch': 0.20096463022508038, 'consumed_train_tokens': 427139}

56

{'loss': 0.7961574077606202, 'learning_rate': 0.00029424541607898447, 'train_speed(iter/s)': 0.54055, 'epoch': 0.204983922829582, 'consumed_train_tokens': 435064}

57

{'loss': 0.866610050201416, 'learning_rate': 0.00029382228490832155, 'train_speed(iter/s)': 0.539877, 'epoch': 0.2090032154340836, 'consumed_train_tokens': 443758}

58

{'loss': 0.8769966125488281, 'learning_rate': 0.00029339915373765863, 'train_speed(iter/s)': 0.540055, 'epoch': 0.21302250803858522, 'consumed_train_tokens': 452682}

59

{'loss': 0.8419232368469238, 'learning_rate': 0.00029255289139633285, 'train_speed(iter/s)': 0.539079, 'epoch': 0.22106109324758844, 'consumed_train_tokens': 470393}

60

{'loss': 0.7519349098205567, 'learning_rate': 0.0002921297602256699, 'train_speed(iter/s)': 0.537791, 'epoch': 0.22508038585209003, 'consumed_train_tokens': 478871}

61

{'loss': 0.8475372314453125, 'learning_rate': 0.000291706629055007, 'train_speed(iter/s)': 0.537861, 'epoch': 0.22909967845659163, 'consumed_train_tokens': 487602}

62

{'loss': 0.6906076431274414, 'learning_rate': 0.0002912834978843441, 'train_speed(iter/s)': 0.537195, 'epoch': 0.23311897106109325, 'consumed_train_tokens': 496585}

63

{'loss': 0.8662967681884766, 'learning_rate': 0.0002908603667136812, 'train_speed(iter/s)': 0.537251, 'epoch': 0.23713826366559485, 'consumed_train_tokens': 505487}

64

{'loss': 0.8760518074035645, 'learning_rate': 0.0002904372355430183, 'train_speed(iter/s)': 0.536931, 'epoch': 0.24115755627009647, 'consumed_train_tokens': 515297}

65

{'loss': 0.7845987796783447, 'learning_rate': 0.0002900141043723554, 'train_speed(iter/s)': 0.536882, 'epoch': 0.24517684887459806, 'consumed_train_tokens': 523379}

66

{'loss': 0.7953667163848877, 'learning_rate': 0.00028959097320169246, 'train_speed(iter/s)': 0.536637, 'epoch': 0.2491961414790997, 'consumed_train_tokens': 532799}

67

{'loss': 0.7942952156066895, 'learning_rate': 0.0002891678420310296, 'train_speed(iter/s)': 0.536215, 'epoch': 0.2532154340836013, 'consumed_train_tokens': 540227}

68

{'loss': 0.7654914855957031, 'learning_rate': 0.0002887447108603667, 'train_speed(iter/s)': 0.535676, 'epoch': 0.2572347266881029, 'consumed_train_tokens': 548563}

69

{'loss': 0.8441455841064454, 'learning_rate': 0.00028832157968970376, 'train_speed(iter/s)': 0.535405, 'epoch': 0.2612540192926045, 'consumed_train_tokens': 557034}

70

{'loss': 0.7345724105834961, 'learning_rate': 0.0002878984485190409, 'train_speed(iter/s)': 0.53508, 'epoch': 0.2652733118971061, 'consumed_train_tokens': 565611}

71

{'loss': 0.9201484680175781, 'learning_rate': 0.00028747531734837797, 'train_speed(iter/s)': 0.534703, 'epoch': 0.2692926045016077, 'consumed_train_tokens': 574055}

72

{'loss': 0.8589251518249512, 'learning_rate': 0.00028705218617771505, 'train_speed(iter/s)': 0.53418, 'epoch': 0.2733118971061093, 'consumed_train_tokens': 582824}

73

{'loss': 0.8710977554321289, 'learning_rate': 0.00028662905500705213, 'train_speed(iter/s)': 0.534113, 'epoch': 0.27733118971061094, 'consumed_train_tokens': 591134}

74

{'loss': 0.8975378036499023, 'learning_rate': 0.00028620592383638927, 'train_speed(iter/s)': 0.534058, 'epoch': 0.28135048231511256, 'consumed_train_tokens': 599763}

75

{'loss': 0.8846827507019043, 'learning_rate': 0.00028578279266572635, 'train_speed(iter/s)': 0.533544, 'epoch': 0.2853697749196141, 'consumed_train_tokens': 608961}

76

{'loss': 0.7930708408355713, 'learning_rate': 0.0002853596614950634, 'train_speed(iter/s)': 0.533528, 'epoch': 0.28938906752411575, 'consumed_train_tokens': 617305}

77

{'loss': 0.721195650100708, 'learning_rate': 0.00028493653032440056, 'train_speed(iter/s)': 0.533183, 'epoch': 0.2934083601286174, 'consumed_train_tokens': 626518}

78

{'loss': 0.8075569152832032, 'learning_rate': 0.00028451339915373764, 'train_speed(iter/s)': 0.532877, 'epoch': 0.297427652733119, 'consumed_train_tokens': 636178}

79

{'loss': 0.8502930641174317, 'learning_rate': 0.0002840902679830747, 'train_speed(iter/s)': 0.532646, 'epoch': 0.30144694533762056, 'consumed_train_tokens': 644613}

80

{'loss': 0.8558016777038574, 'learning_rate': 0.0002836671368124118, 'train_speed(iter/s)': 0.532488, 'epoch': 0.3054662379421222, 'consumed_train_tokens': 653374}

81

{'loss': 0.8601596832275391, 'learning_rate': 0.00028324400564174894, 'train_speed(iter/s)': 0.532273, 'epoch': 0.3094855305466238, 'consumed_train_tokens': 662234}

82

{'loss': 0.8032548904418946, 'learning_rate': 0.000282820874471086, 'train_speed(iter/s)': 0.5323, 'epoch': 0.3135048231511254, 'consumed_train_tokens': 671302}

83

{'loss': 0.8246597290039063, 'learning_rate': 0.0002823977433004231, 'train_speed(iter/s)': 0.532051, 'epoch': 0.317524115755627, 'consumed_train_tokens': 680232}

84

{'loss': 0.6792694091796875, 'learning_rate': 0.0002819746121297602, 'train_speed(iter/s)': 0.531989, 'epoch': 0.3215434083601286, 'consumed_train_tokens': 688970}

85

{'loss': 0.7928378582000732, 'learning_rate': 0.0002815514809590973, 'train_speed(iter/s)': 0.531772, 'epoch': 0.32556270096463025, 'consumed_train_tokens': 698305}

86

{'loss': 0.9227901458740234, 'learning_rate': 0.0002811283497884344, 'train_speed(iter/s)': 0.531981, 'epoch': 0.3295819935691318, 'consumed_train_tokens': 707290}

87

{'loss': 0.7304674625396729, 'learning_rate': 0.00028070521861777147, 'train_speed(iter/s)': 0.532034, 'epoch': 0.33360128617363344, 'consumed_train_tokens': 715601}

88

{'loss': 0.7545377254486084, 'learning_rate': 0.0002802820874471086, 'train_speed(iter/s)': 0.532403, 'epoch': 0.33762057877813506, 'consumed_train_tokens': 724422}

89

{'loss': 0.7585969924926758, 'learning_rate': 0.0002798589562764457, 'train_speed(iter/s)': 0.532458, 'epoch': 0.34163987138263663, 'consumed_train_tokens': 733324}

90

{'loss': 0.8385601043701172, 'learning_rate': 0.00027943582510578277, 'train_speed(iter/s)': 0.532552, 'epoch': 0.34565916398713825, 'consumed_train_tokens': 742767}

91

{'loss': 0.7999014377593994, 'learning_rate': 0.00027901269393511985, 'train_speed(iter/s)': 0.532447, 'epoch': 0.3496784565916399, 'consumed_train_tokens': 751171}

92

{'loss': 0.7950222969055176, 'learning_rate': 0.000278589562764457, 'train_speed(iter/s)': 0.532454, 'epoch': 0.3536977491961415, 'consumed_train_tokens': 759618}

93

{'loss': 0.8129806518554688, 'learning_rate': 0.00027816643159379406, 'train_speed(iter/s)': 0.532446, 'epoch': 0.35771704180064307, 'consumed_train_tokens': 768070}

94

{'loss': 0.8546628952026367, 'learning_rate': 0.00027774330042313114, 'train_speed(iter/s)': 0.532455, 'epoch': 0.3617363344051447, 'consumed_train_tokens': 776901}

95

{'loss': 0.7964793682098389, 'learning_rate': 0.0002773201692524682, 'train_speed(iter/s)': 0.532338, 'epoch': 0.3657556270096463, 'consumed_train_tokens': 785665}

96

{'loss': 0.9306144714355469, 'learning_rate': 0.00027689703808180536, 'train_speed(iter/s)': 0.532335, 'epoch': 0.36977491961414793, 'consumed_train_tokens': 794626}

97

{'loss': 0.7662803649902343, 'learning_rate': 0.00027647390691114244, 'train_speed(iter/s)': 0.532145, 'epoch': 0.3737942122186495, 'consumed_train_tokens': 803017}

98

{'loss': 0.6558635711669922, 'learning_rate': 0.0002760507757404795, 'train_speed(iter/s)': 0.532247, 'epoch': 0.3778135048231511, 'consumed_train_tokens': 811853}

99

{'loss': 0.8598474502563477, 'learning_rate': 0.00027562764456981665, 'train_speed(iter/s)': 0.531646, 'epoch': 0.38183279742765275, 'consumed_train_tokens': 820635}

100

{'loss': 0.8442249298095703, 'learning_rate': 0.00027520451339915373, 'train_speed(iter/s)': 0.531615, 'epoch': 0.3858520900321543, 'consumed_train_tokens': 828983}

101

{'loss': 0.7738531112670899, 'learning_rate': 0.0002747813822284908, 'train_speed(iter/s)': 0.531534, 'epoch': 0.38987138263665594, 'consumed_train_tokens': 837523}

102

{'loss': 0.8740942001342773, 'learning_rate': 0.0002743582510578279, 'train_speed(iter/s)': 0.531444, 'epoch': 0.39389067524115756, 'consumed_train_tokens': 846017}

103

{'loss': 0.7188767433166504, 'learning_rate': 0.00027393511988716503, 'train_speed(iter/s)': 0.531211, 'epoch': 0.3979099678456592, 'consumed_train_tokens': 853490}

104

{'loss': 0.8046602249145508, 'learning_rate': 0.0002735119887165021, 'train_speed(iter/s)': 0.531225, 'epoch': 0.40192926045016075, 'consumed_train_tokens': 862437}

105

{'loss': 0.9220663070678711, 'learning_rate': 0.0002730888575458392, 'train_speed(iter/s)': 0.531251, 'epoch': 0.4059485530546624, 'consumed_train_tokens': 871895}

106

{'loss': 0.7777037620544434, 'learning_rate': 0.0002726657263751763, 'train_speed(iter/s)': 0.531056, 'epoch': 0.409967845659164, 'consumed_train_tokens': 879816}

107

{'loss': 0.7948966979980469, 'learning_rate': 0.0002722425952045134, 'train_speed(iter/s)': 0.531061, 'epoch': 0.4139871382636656, 'consumed_train_tokens': 889188}

108

{'loss': 0.8626812934875489, 'learning_rate': 0.00027181946403385043, 'train_speed(iter/s)': 0.531084, 'epoch': 0.4180064308681672, 'consumed_train_tokens': 897566}

109

{'loss': 0.7233026504516602, 'learning_rate': 0.00027139633286318756, 'train_speed(iter/s)': 0.531179, 'epoch': 0.4220257234726688, 'consumed_train_tokens': 906818}

110

{'loss': 0.8222451210021973, 'learning_rate': 0.00027097320169252464, 'train_speed(iter/s)': 0.531256, 'epoch': 0.42604501607717044, 'consumed_train_tokens': 915909}

111

{'loss': 0.7423653602600098, 'learning_rate': 0.0002705500705218617, 'train_speed(iter/s)': 0.531265, 'epoch': 0.430064308681672, 'consumed_train_tokens': 924660}

112

{'loss': 0.7988332748413086, 'learning_rate': 0.00027012693935119886, 'train_speed(iter/s)': 0.531324, 'epoch': 0.4340836012861736, 'consumed_train_tokens': 933423}

113

{'loss': 0.8511985778808594, 'learning_rate': 0.00026970380818053594, 'train_speed(iter/s)': 0.531366, 'epoch': 0.43810289389067525, 'consumed_train_tokens': 942498}

114

{'loss': 0.7560265064239502, 'learning_rate': 0.000269280677009873, 'train_speed(iter/s)': 0.531471, 'epoch': 0.44212218649517687, 'consumed_train_tokens': 951531}

115

{'loss': 0.8438371658325196, 'learning_rate': 0.0002688575458392101, 'train_speed(iter/s)': 0.531643, 'epoch': 0.44614147909967844, 'consumed_train_tokens': 960199}

116

{'loss': 0.802060317993164, 'learning_rate': 0.00026843441466854723, 'train_speed(iter/s)': 0.531759, 'epoch': 0.45016077170418006, 'consumed_train_tokens': 969075}

117

{'loss': 0.7969474792480469, 'learning_rate': 0.0002680112834978843, 'train_speed(iter/s)': 0.531935, 'epoch': 0.4541800643086817, 'consumed_train_tokens': 978401}

118

{'loss': 0.8446496009826661, 'learning_rate': 0.0002675881523272214, 'train_speed(iter/s)': 0.532008, 'epoch': 0.45819935691318325, 'consumed_train_tokens': 986524}

119

{'loss': 0.7670262336730957, 'learning_rate': 0.00026716502115655853, 'train_speed(iter/s)': 0.532035, 'epoch': 0.4622186495176849, 'consumed_train_tokens': 995449}

120

{'loss': 0.8028373718261719, 'learning_rate': 0.0002667418899858956, 'train_speed(iter/s)': 0.531939, 'epoch': 0.4662379421221865, 'consumed_train_tokens': 1004341}

121

{'loss': 0.7902628898620605, 'learning_rate': 0.0002663187588152327, 'train_speed(iter/s)': 0.532127, 'epoch': 0.4702572347266881, 'consumed_train_tokens': 1013077}

122

{'loss': 0.840037727355957, 'learning_rate': 0.00026589562764456977, 'train_speed(iter/s)': 0.532035, 'epoch': 0.4742765273311897, 'consumed_train_tokens': 1022348}

123

{'loss': 0.87603759765625, 'learning_rate': 0.0002654724964739069, 'train_speed(iter/s)': 0.532207, 'epoch': 0.4782958199356913, 'consumed_train_tokens': 1032404}

124

{'loss': 0.7641261100769043, 'learning_rate': 0.000265049365303244, 'train_speed(iter/s)': 0.532195, 'epoch': 0.48231511254019294, 'consumed_train_tokens': 1040785}

125

{'loss': 0.8184738159179688, 'learning_rate': 0.00026462623413258106, 'train_speed(iter/s)': 0.532205, 'epoch': 0.48633440514469456, 'consumed_train_tokens': 1049595}

126

{'loss': 0.8273519515991211, 'learning_rate': 0.00026420310296191814, 'train_speed(iter/s)': 0.532122, 'epoch': 0.4903536977491961, 'consumed_train_tokens': 1058868}

127

{'loss': 0.7222124099731445, 'learning_rate': 0.0002637799717912553, 'train_speed(iter/s)': 0.532185, 'epoch': 0.49437299035369775, 'consumed_train_tokens': 1068539}

128

{'loss': 0.8103645324707032, 'learning_rate': 0.00026335684062059236, 'train_speed(iter/s)': 0.532143, 'epoch': 0.4983922829581994, 'consumed_train_tokens': 1076420}

129

{'loss': 0.7813583850860596, 'learning_rate': 0.00026293370944992944, 'train_speed(iter/s)': 0.532155, 'epoch': 0.502411575562701, 'consumed_train_tokens': 1084974}

130

{'loss': 0.7388108253479004, 'learning_rate': 0.0002625105782792666, 'train_speed(iter/s)': 0.532018, 'epoch': 0.5064308681672026, 'consumed_train_tokens': 1093400}

131

{'loss': 0.8103572845458984, 'learning_rate': 0.00026208744710860365, 'train_speed(iter/s)': 0.532054, 'epoch': 0.5104501607717041, 'consumed_train_tokens': 1101924}

132

{'loss': 0.7125824928283692, 'learning_rate': 0.00026166431593794073, 'train_speed(iter/s)': 0.531808, 'epoch': 0.5144694533762058, 'consumed_train_tokens': 1110986}

133

{'loss': 0.8992618560791016, 'learning_rate': 0.0002612411847672778, 'train_speed(iter/s)': 0.531777, 'epoch': 0.5184887459807074, 'consumed_train_tokens': 1119505}

134

{'loss': 0.8297893524169921, 'learning_rate': 0.00026081805359661495, 'train_speed(iter/s)': 0.531675, 'epoch': 0.522508038585209, 'consumed_train_tokens': 1127995}

135

{'loss': 0.8079532623291016, 'learning_rate': 0.00026039492242595203, 'train_speed(iter/s)': 0.531711, 'epoch': 0.5265273311897106, 'consumed_train_tokens': 1136945}

136

{'loss': 0.8293464660644532, 'learning_rate': 0.0002599717912552891, 'train_speed(iter/s)': 0.531615, 'epoch': 0.5305466237942122, 'consumed_train_tokens': 1145770}

137

{'loss': 0.7730323791503906, 'learning_rate': 0.0002595486600846262, 'train_speed(iter/s)': 0.53178, 'epoch': 0.5345659163987139, 'consumed_train_tokens': 1153951}

138

{'loss': 0.8591691970825195, 'learning_rate': 0.0002591255289139633, 'train_speed(iter/s)': 0.531675, 'epoch': 0.5385852090032154, 'consumed_train_tokens': 1162410}

139

{'loss': 0.7916035175323486, 'learning_rate': 0.0002587023977433004, 'train_speed(iter/s)': 0.531748, 'epoch': 0.542604501607717, 'consumed_train_tokens': 1171332}

140

{'loss': 0.7837770462036133, 'learning_rate': 0.0002582792665726375, 'train_speed(iter/s)': 0.531649, 'epoch': 0.5466237942122186, 'consumed_train_tokens': 1180079}

141

{'loss': 0.7658630847930908, 'learning_rate': 0.0002578561354019746, 'train_speed(iter/s)': 0.531667, 'epoch': 0.5506430868167203, 'consumed_train_tokens': 1188260}

142

{'loss': 0.8287040710449218, 'learning_rate': 0.0002574330042313117, 'train_speed(iter/s)': 0.531664, 'epoch': 0.5546623794212219, 'consumed_train_tokens': 1197146}

143

{'loss': 0.8641443252563477, 'learning_rate': 0.0002570098730606488, 'train_speed(iter/s)': 0.531773, 'epoch': 0.5586816720257235, 'consumed_train_tokens': 1205868}

144

{'loss': 0.7267802238464356, 'learning_rate': 0.00025658674188998586, 'train_speed(iter/s)': 0.531782, 'epoch': 0.5627009646302251, 'consumed_train_tokens': 1215192}

145

{'loss': 0.780949878692627, 'learning_rate': 0.000256163610719323, 'train_speed(iter/s)': 0.531936, 'epoch': 0.5667202572347267, 'consumed_train_tokens': 1223874}

146

{'loss': 0.7724663734436035, 'learning_rate': 0.0002557404795486601, 'train_speed(iter/s)': 0.531991, 'epoch': 0.5707395498392283, 'consumed_train_tokens': 1232510}

147

{'loss': 0.7763169288635254, 'learning_rate': 0.00025531734837799715, 'train_speed(iter/s)': 0.53209, 'epoch': 0.5747588424437299, 'consumed_train_tokens': 1240473}

148

{'loss': 0.7899177551269532, 'learning_rate': 0.0002548942172073343, 'train_speed(iter/s)': 0.532197, 'epoch': 0.5787781350482315, 'consumed_train_tokens': 1249168}

149

{'loss': 0.7572986125946045, 'learning_rate': 0.00025447108603667137, 'train_speed(iter/s)': 0.532337, 'epoch': 0.5827974276527331, 'consumed_train_tokens': 1258241}

150

{'loss': 0.8219436645507813, 'learning_rate': 0.00025404795486600845, 'train_speed(iter/s)': 0.532461, 'epoch': 0.5868167202572347, 'consumed_train_tokens': 1266188}

151

{'loss': 0.775553560256958, 'learning_rate': 0.00025362482369534553, 'train_speed(iter/s)': 0.532605, 'epoch': 0.5908360128617364, 'consumed_train_tokens': 1275196}

152

{'loss': 0.7995999813079834, 'learning_rate': 0.00025320169252468266, 'train_speed(iter/s)': 0.532695, 'epoch': 0.594855305466238, 'consumed_train_tokens': 1284121}

153

{'loss': 0.8210943222045899, 'learning_rate': 0.00025277856135401974, 'train_speed(iter/s)': 0.532877, 'epoch': 0.5988745980707395, 'consumed_train_tokens': 1292357}

154

{'loss': 0.8863027572631836, 'learning_rate': 0.0002523554301833568, 'train_speed(iter/s)': 0.533102, 'epoch': 0.6028938906752411, 'consumed_train_tokens': 1301609}

155

{'loss': 0.8249765396118164, 'learning_rate': 0.0002519322990126939, 'train_speed(iter/s)': 0.533092, 'epoch': 0.6069131832797428, 'consumed_train_tokens': 1310693}

156

{'loss': 0.7976859092712403, 'learning_rate': 0.00025150916784203104, 'train_speed(iter/s)': 0.533216, 'epoch': 0.6109324758842444, 'consumed_train_tokens': 1319791}

157

{'loss': 0.8995546340942383, 'learning_rate': 0.0002510860366713681, 'train_speed(iter/s)': 0.533114, 'epoch': 0.614951768488746, 'consumed_train_tokens': 1328783}

158

{'loss': 0.7851147651672363, 'learning_rate': 0.0002506629055007052, 'train_speed(iter/s)': 0.533181, 'epoch': 0.6189710610932476, 'consumed_train_tokens': 1337379}

159

{'loss': 0.8191974639892579, 'learning_rate': 0.0002502397743300423, 'train_speed(iter/s)': 0.533189, 'epoch': 0.6229903536977492, 'consumed_train_tokens': 1346508}

160

{'loss': 0.798715877532959, 'learning_rate': 0.00024981664315937936, 'train_speed(iter/s)': 0.5333, 'epoch': 0.6270096463022508, 'consumed_train_tokens': 1355368}

161

{'loss': 0.7575325012207031, 'learning_rate': 0.00024939351198871644, 'train_speed(iter/s)': 0.533401, 'epoch': 0.6310289389067524, 'consumed_train_tokens': 1364458}

162

{'loss': 0.7803393363952636, 'learning_rate': 0.0002489703808180536, 'train_speed(iter/s)': 0.533492, 'epoch': 0.635048231511254, 'consumed_train_tokens': 1372771}

163

{'loss': 0.8365579605102539, 'learning_rate': 0.00024854724964739065, 'train_speed(iter/s)': 0.533594, 'epoch': 0.6390675241157556, 'consumed_train_tokens': 1381573}

164

{'loss': 0.7778121948242187, 'learning_rate': 0.00024812411847672773, 'train_speed(iter/s)': 0.533734, 'epoch': 0.6430868167202572, 'consumed_train_tokens': 1390232}

165

{'loss': 0.7785665988922119, 'learning_rate': 0.00024770098730606487, 'train_speed(iter/s)': 0.533756, 'epoch': 0.6471061093247589, 'consumed_train_tokens': 1398599}

166

{'loss': 0.717808723449707, 'learning_rate': 0.00024727785613540195, 'train_speed(iter/s)': 0.533907, 'epoch': 0.6511254019292605, 'consumed_train_tokens': 1406772}

167

{'loss': 0.8161065101623535, 'learning_rate': 0.00024685472496473903, 'train_speed(iter/s)': 0.533856, 'epoch': 0.655144694533762, 'consumed_train_tokens': 1415434}

168

{'loss': 0.8455538749694824, 'learning_rate': 0.0002464315937940761, 'train_speed(iter/s)': 0.533982, 'epoch': 0.6591639871382636, 'consumed_train_tokens': 1424346}

169

{'loss': 0.8229450225830078, 'learning_rate': 0.00024600846262341324, 'train_speed(iter/s)': 0.534015, 'epoch': 0.6631832797427653, 'consumed_train_tokens': 1433362}

170

{'loss': 0.7775612831115722, 'learning_rate': 0.0002455853314527503, 'train_speed(iter/s)': 0.534195, 'epoch': 0.6672025723472669, 'consumed_train_tokens': 1441421}

171

{'loss': 0.7585845470428467, 'learning_rate': 0.0002451622002820874, 'train_speed(iter/s)': 0.534282, 'epoch': 0.6712218649517685, 'consumed_train_tokens': 1449606}

172

{'loss': 0.8019298553466797, 'learning_rate': 0.00024473906911142454, 'train_speed(iter/s)': 0.534401, 'epoch': 0.6752411575562701, 'consumed_train_tokens': 1458507}

173

{'loss': 0.7020326137542725, 'learning_rate': 0.0002443159379407616, 'train_speed(iter/s)': 0.534379, 'epoch': 0.6792604501607717, 'consumed_train_tokens': 1466668}

174

{'loss': 0.7443767070770264, 'learning_rate': 0.0002438928067700987, 'train_speed(iter/s)': 0.534495, 'epoch': 0.6832797427652733, 'consumed_train_tokens': 1475633}

175

{'loss': 0.7043767929077148, 'learning_rate': 0.0002434696755994358, 'train_speed(iter/s)': 0.534408, 'epoch': 0.6872990353697749, 'consumed_train_tokens': 1484521}

176

{'loss': 0.8345541000366211, 'learning_rate': 0.0002430465444287729, 'train_speed(iter/s)': 0.534449, 'epoch': 0.6913183279742765, 'consumed_train_tokens': 1492982}

177

{'loss': 0.7470024585723877, 'learning_rate': 0.00024262341325811, 'train_speed(iter/s)': 0.534498, 'epoch': 0.6953376205787781, 'consumed_train_tokens': 1501611}

178

{'loss': 0.7556286811828613, 'learning_rate': 0.00024220028208744707, 'train_speed(iter/s)': 0.534515, 'epoch': 0.6993569131832797, 'consumed_train_tokens': 1510189}

179

{'loss': 0.8173888206481934, 'learning_rate': 0.00024177715091678418, 'train_speed(iter/s)': 0.534547, 'epoch': 0.7033762057877814, 'consumed_train_tokens': 1519117}

180

{'loss': 0.8657959938049317, 'learning_rate': 0.00024135401974612126, 'train_speed(iter/s)': 0.534555, 'epoch': 0.707395498392283, 'consumed_train_tokens': 1528555}

181

{'loss': 0.8292352676391601, 'learning_rate': 0.00024093088857545837, 'train_speed(iter/s)': 0.534609, 'epoch': 0.7114147909967846, 'consumed_train_tokens': 1537437}

182

{'loss': 0.7509412288665771, 'learning_rate': 0.00024050775740479548, 'train_speed(iter/s)': 0.534615, 'epoch': 0.7154340836012861, 'consumed_train_tokens': 1545448}

183

{'loss': 0.7363619804382324, 'learning_rate': 0.00024008462623413256, 'train_speed(iter/s)': 0.534638, 'epoch': 0.7194533762057878, 'consumed_train_tokens': 1554172}

184

{'loss': 0.822944450378418, 'learning_rate': 0.00023966149506346966, 'train_speed(iter/s)': 0.534721, 'epoch': 0.7234726688102894, 'consumed_train_tokens': 1563627}

185

{'loss': 0.7454671859741211, 'learning_rate': 0.00023923836389280675, 'train_speed(iter/s)': 0.534721, 'epoch': 0.727491961414791, 'consumed_train_tokens': 1571929}

186

{'loss': 0.7768205642700196, 'learning_rate': 0.00023881523272214385, 'train_speed(iter/s)': 0.534814, 'epoch': 0.7315112540192926, 'consumed_train_tokens': 1581217}

187

{'loss': 0.793757438659668, 'learning_rate': 0.00023839210155148093, 'train_speed(iter/s)': 0.534845, 'epoch': 0.7355305466237942, 'consumed_train_tokens': 1590272}

188

{'loss': 0.8101380348205567, 'learning_rate': 0.00023796897038081804, 'train_speed(iter/s)': 0.534961, 'epoch': 0.7395498392282959, 'consumed_train_tokens': 1598771}

189

{'loss': 0.697064733505249, 'learning_rate': 0.00023754583921015512, 'train_speed(iter/s)': 0.534968, 'epoch': 0.7435691318327974, 'consumed_train_tokens': 1607701}

190

{'loss': 0.8050601959228516, 'learning_rate': 0.00023712270803949223, 'train_speed(iter/s)': 0.535013, 'epoch': 0.747588424437299, 'consumed_train_tokens': 1617088}

191

{'loss': 0.8072690010070801, 'learning_rate': 0.00023669957686882933, 'train_speed(iter/s)': 0.53509, 'epoch': 0.7516077170418006, 'consumed_train_tokens': 1625529}

192

{'loss': 0.7463855743408203, 'learning_rate': 0.00023627644569816642, 'train_speed(iter/s)': 0.535154, 'epoch': 0.7556270096463023, 'consumed_train_tokens': 1634656}

193

{'loss': 0.781770896911621, 'learning_rate': 0.00023585331452750352, 'train_speed(iter/s)': 0.535125, 'epoch': 0.7596463022508039, 'consumed_train_tokens': 1643269}

194

{'loss': 0.7528175354003906, 'learning_rate': 0.0002354301833568406, 'train_speed(iter/s)': 0.535065, 'epoch': 0.7636655948553055, 'consumed_train_tokens': 1651871}

195

{'loss': 0.8199456214904786, 'learning_rate': 0.0002350070521861777, 'train_speed(iter/s)': 0.5351, 'epoch': 0.7676848874598071, 'consumed_train_tokens': 1660337}

196

{'loss': 0.8293247222900391, 'learning_rate': 0.0002345839210155148, 'train_speed(iter/s)': 0.535151, 'epoch': 0.7717041800643086, 'consumed_train_tokens': 1669086}

197

{'loss': 0.836673355102539, 'learning_rate': 0.0002341607898448519, 'train_speed(iter/s)': 0.535106, 'epoch': 0.7757234726688103, 'consumed_train_tokens': 1678244}

198

{'loss': 0.7678126335144043, 'learning_rate': 0.00023373765867418898, 'train_speed(iter/s)': 0.535141, 'epoch': 0.7797427652733119, 'consumed_train_tokens': 1687209}

199

{'loss': 0.7658403396606446, 'learning_rate': 0.00023331452750352609, 'train_speed(iter/s)': 0.535184, 'epoch': 0.7837620578778135, 'consumed_train_tokens': 1696222}

200

{'loss': 0.8917409896850585, 'learning_rate': 0.00023289139633286317, 'train_speed(iter/s)': 0.535166, 'epoch': 0.7877813504823151, 'consumed_train_tokens': 1704725}

201

{'loss': 0.7546669960021972, 'learning_rate': 0.00023246826516220027, 'train_speed(iter/s)': 0.535274, 'epoch': 0.7918006430868167, 'consumed_train_tokens': 1714083}

202

{'loss': 0.7179839134216308, 'learning_rate': 0.00023204513399153738, 'train_speed(iter/s)': 0.535307, 'epoch': 0.7958199356913184, 'consumed_train_tokens': 1722556}

203

{'loss': 0.7524504661560059, 'learning_rate': 0.00023162200282087446, 'train_speed(iter/s)': 0.535365, 'epoch': 0.7998392282958199, 'consumed_train_tokens': 1730991}

204

{'loss': 0.8016445159912109, 'learning_rate': 0.00023119887165021157, 'train_speed(iter/s)': 0.53535, 'epoch': 0.8038585209003215, 'consumed_train_tokens': 1738978}

205

{'loss': 0.8262340545654296, 'learning_rate': 0.00023077574047954865, 'train_speed(iter/s)': 0.535429, 'epoch': 0.8078778135048231, 'consumed_train_tokens': 1747233}

206

{'loss': 0.6130701065063476, 'learning_rate': 0.00023035260930888576, 'train_speed(iter/s)': 0.535406, 'epoch': 0.8118971061093248, 'consumed_train_tokens': 1755649}

207

{'loss': 0.6921074390411377, 'learning_rate': 0.00022992947813822284, 'train_speed(iter/s)': 0.535532, 'epoch': 0.8159163987138264, 'consumed_train_tokens': 1764760}

208

{'loss': 0.7507166862487793, 'learning_rate': 0.00022950634696755994, 'train_speed(iter/s)': 0.535565, 'epoch': 0.819935691318328, 'consumed_train_tokens': 1773805}

209

{'loss': 0.746689748764038, 'learning_rate': 0.000229083215796897, 'train_speed(iter/s)': 0.535532, 'epoch': 0.8239549839228296, 'consumed_train_tokens': 1782930}

210

{'loss': 0.7338207721710205, 'learning_rate': 0.0002286600846262341, 'train_speed(iter/s)': 0.535511, 'epoch': 0.8279742765273312, 'consumed_train_tokens': 1791461}

211

{'loss': 0.6678847789764404, 'learning_rate': 0.00022823695345557118, 'train_speed(iter/s)': 0.535565, 'epoch': 0.8319935691318328, 'consumed_train_tokens': 1800514}

212

{'loss': 0.7685139656066895, 'learning_rate': 0.0002278138222849083, 'train_speed(iter/s)': 0.535595, 'epoch': 0.8360128617363344, 'consumed_train_tokens': 1808594}

213

{'loss': 0.5967988967895508, 'learning_rate': 0.00022739069111424537, 'train_speed(iter/s)': 0.535752, 'epoch': 0.840032154340836, 'consumed_train_tokens': 1817582}

214

{'loss': 0.7895120143890381, 'learning_rate': 0.00022696755994358248, 'train_speed(iter/s)': 0.535815, 'epoch': 0.8440514469453376, 'consumed_train_tokens': 1825667}

215

{'loss': 0.7897820472717285, 'learning_rate': 0.00022654442877291959, 'train_speed(iter/s)': 0.535939, 'epoch': 0.8480707395498392, 'consumed_train_tokens': 1834173}

216

{'loss': 0.7932607173919678, 'learning_rate': 0.00022612129760225667, 'train_speed(iter/s)': 0.535971, 'epoch': 0.8520900321543409, 'consumed_train_tokens': 1843275}

217

{'loss': 0.8124246597290039, 'learning_rate': 0.00022569816643159377, 'train_speed(iter/s)': 0.535958, 'epoch': 0.8561093247588425, 'consumed_train_tokens': 1852629}

218

{'loss': 0.8202606201171875, 'learning_rate': 0.00022527503526093085, 'train_speed(iter/s)': 0.536038, 'epoch': 0.860128617363344, 'consumed_train_tokens': 1861509}

219

{'loss': 0.7376375198364258, 'learning_rate': 0.00022485190409026796, 'train_speed(iter/s)': 0.536124, 'epoch': 0.8641479099678456, 'consumed_train_tokens': 1869882}

220

{'loss': 0.7183916568756104, 'learning_rate': 0.00022442877291960504, 'train_speed(iter/s)': 0.536202, 'epoch': 0.8681672025723473, 'consumed_train_tokens': 1878542}

221

{'loss': 0.7255347728729248, 'learning_rate': 0.00022400564174894215, 'train_speed(iter/s)': 0.53629, 'epoch': 0.8721864951768489, 'consumed_train_tokens': 1886908}

222

{'loss': 0.7251108646392822, 'learning_rate': 0.00022358251057827923, 'train_speed(iter/s)': 0.536347, 'epoch': 0.8762057877813505, 'consumed_train_tokens': 1896164}

223

{'loss': 0.7680606842041016, 'learning_rate': 0.00022315937940761634, 'train_speed(iter/s)': 0.536357, 'epoch': 0.8802250803858521, 'consumed_train_tokens': 1904345}

224

{'loss': 0.8178493499755859, 'learning_rate': 0.00022273624823695342, 'train_speed(iter/s)': 0.536451, 'epoch': 0.8842443729903537, 'consumed_train_tokens': 1913037}

225

{'loss': 0.8236395835876464, 'learning_rate': 0.00022231311706629052, 'train_speed(iter/s)': 0.536545, 'epoch': 0.8882636655948553, 'consumed_train_tokens': 1921134}

226

{'loss': 0.7734375953674316, 'learning_rate': 0.00022188998589562763, 'train_speed(iter/s)': 0.5365, 'epoch': 0.8922829581993569, 'consumed_train_tokens': 1929974}

227

{'loss': 0.8022323608398437, 'learning_rate': 0.0002214668547249647, 'train_speed(iter/s)': 0.536534, 'epoch': 0.8963022508038585, 'consumed_train_tokens': 1938493}

228

{'loss': 0.7937729835510254, 'learning_rate': 0.00022104372355430182, 'train_speed(iter/s)': 0.536587, 'epoch': 0.9003215434083601, 'consumed_train_tokens': 1946672}

229

{'loss': 0.8018917083740235, 'learning_rate': 0.0002206205923836389, 'train_speed(iter/s)': 0.536577, 'epoch': 0.9043408360128617, 'consumed_train_tokens': 1954942}

230

{'loss': 0.7517464637756348, 'learning_rate': 0.000220197461212976, 'train_speed(iter/s)': 0.536627, 'epoch': 0.9083601286173634, 'consumed_train_tokens': 1964075}

231

{'loss': 0.8100460052490235, 'learning_rate': 0.0002197743300423131, 'train_speed(iter/s)': 0.536719, 'epoch': 0.912379421221865, 'consumed_train_tokens': 1973040}

232

{'loss': 0.775754976272583, 'learning_rate': 0.0002193511988716502, 'train_speed(iter/s)': 0.536676, 'epoch': 0.9163987138263665, 'consumed_train_tokens': 1981580}

233

{'loss': 0.7712797164916992, 'learning_rate': 0.00021892806770098727, 'train_speed(iter/s)': 0.536673, 'epoch': 0.9204180064308681, 'consumed_train_tokens': 1990266}

234

{'loss': 0.7337568283081055, 'learning_rate': 0.00021850493653032438, 'train_speed(iter/s)': 0.536763, 'epoch': 0.9244372990353698, 'consumed_train_tokens': 1998195}

235

{'loss': 0.8030716896057128, 'learning_rate': 0.0002180818053596615, 'train_speed(iter/s)': 0.536679, 'epoch': 0.9284565916398714, 'consumed_train_tokens': 2006955}

236

{'loss': 0.7321510791778565, 'learning_rate': 0.00021765867418899857, 'train_speed(iter/s)': 0.53681, 'epoch': 0.932475884244373, 'consumed_train_tokens': 2014754}

237

{'loss': 0.8128586769104004, 'learning_rate': 0.00021723554301833568, 'train_speed(iter/s)': 0.536795, 'epoch': 0.9364951768488746, 'consumed_train_tokens': 2024164}

238

{'loss': 0.908284854888916, 'learning_rate': 0.00021681241184767276, 'train_speed(iter/s)': 0.536843, 'epoch': 0.9405144694533762, 'consumed_train_tokens': 2032832}

239

{'loss': 0.8197904586791992, 'learning_rate': 0.00021638928067700986, 'train_speed(iter/s)': 0.536849, 'epoch': 0.9445337620578779, 'consumed_train_tokens': 2041977}

240

{'loss': 0.7299350261688232, 'learning_rate': 0.00021596614950634694, 'train_speed(iter/s)': 0.536941, 'epoch': 0.9485530546623794, 'consumed_train_tokens': 2050770}

241

{'loss': 0.7733146667480468, 'learning_rate': 0.00021554301833568405, 'train_speed(iter/s)': 0.537008, 'epoch': 0.952572347266881, 'consumed_train_tokens': 2059606}

242

{'loss': 0.8048017501831055, 'learning_rate': 0.00021511988716502113, 'train_speed(iter/s)': 0.536961, 'epoch': 0.9565916398713826, 'consumed_train_tokens': 2068232}

243

{'loss': 0.7493824005126953, 'learning_rate': 0.00021469675599435824, 'train_speed(iter/s)': 0.536974, 'epoch': 0.9606109324758842, 'consumed_train_tokens': 2077212}

244

{'loss': 0.7577905654907227, 'learning_rate': 0.00021427362482369535, 'train_speed(iter/s)': 0.536984, 'epoch': 0.9646302250803859, 'consumed_train_tokens': 2086453}

245

{'loss': 0.8565336227416992, 'learning_rate': 0.00021385049365303243, 'train_speed(iter/s)': 0.537003, 'epoch': 0.9686495176848875, 'consumed_train_tokens': 2095873}

246

{'loss': 0.7684191226959228, 'learning_rate': 0.00021342736248236953, 'train_speed(iter/s)': 0.537, 'epoch': 0.9726688102893891, 'consumed_train_tokens': 2105011}

247

{'loss': 0.852409553527832, 'learning_rate': 0.00021300423131170661, 'train_speed(iter/s)': 0.536997, 'epoch': 0.9766881028938906, 'consumed_train_tokens': 2113735}

248

{'loss': 0.7947371006011963, 'learning_rate': 0.00021258110014104372, 'train_speed(iter/s)': 0.536974, 'epoch': 0.9807073954983923, 'consumed_train_tokens': 2122314}

249

{'loss': 0.7411166191101074, 'learning_rate': 0.0002121579689703808, 'train_speed(iter/s)': 0.53698, 'epoch': 0.9847266881028939, 'consumed_train_tokens': 2131493}

250

{'loss': 0.8682092666625977, 'learning_rate': 0.0002117348377997179, 'train_speed(iter/s)': 0.53693, 'epoch': 0.9887459807073955, 'consumed_train_tokens': 2140190}

251

{'loss': 0.788865041732788, 'learning_rate': 0.000211311706629055, 'train_speed(iter/s)': 0.536938, 'epoch': 0.9927652733118971, 'consumed_train_tokens': 2149357}

252

{'loss': 0.8286192893981934, 'learning_rate': 0.0002108885754583921, 'train_speed(iter/s)': 0.536974, 'epoch': 0.9967845659163987, 'consumed_train_tokens': 2158265}

253

{'eval_loss': 0.7436836957931519, 'eval_runtime': 18.8386, 'eval_samples_per_second': 53.083, 'eval_steps_per_second': 13.271, 'epoch': 1.0}

254

{'loss': 0.7373550415039063, 'learning_rate': 0.00021046544428772918, 'train_speed(iter/s)': 0.532408, 'epoch': 1.0008038585209003, 'consumed_train_tokens': 2166941}

255

{'loss': 0.6263211250305176, 'learning_rate': 0.00021004231311706628, 'train_speed(iter/s)': 0.532508, 'epoch': 1.004823151125402, 'consumed_train_tokens': 2175328}

256

{'loss': 0.6996028900146485, 'learning_rate': 0.0002096191819464034, 'train_speed(iter/s)': 0.532507, 'epoch': 1.0088424437299035, 'consumed_train_tokens': 2183475}

257

{'loss': 0.5378609657287597, 'learning_rate': 0.00020919605077574047, 'train_speed(iter/s)': 0.532645, 'epoch': 1.0128617363344052, 'consumed_train_tokens': 2192056}

258

{'loss': 0.5291036128997803, 'learning_rate': 0.00020877291960507758, 'train_speed(iter/s)': 0.532708, 'epoch': 1.0168810289389068, 'consumed_train_tokens': 2200362}

259

{'loss': 0.738727331161499, 'learning_rate': 0.00020834978843441466, 'train_speed(iter/s)': 0.532856, 'epoch': 1.0209003215434083, 'consumed_train_tokens': 2208994}

260

{'loss': 0.6211291313171386, 'learning_rate': 0.00020792665726375177, 'train_speed(iter/s)': 0.533031, 'epoch': 1.02491961414791, 'consumed_train_tokens': 2217653}

261

{'loss': 0.745482063293457, 'learning_rate': 0.00020750352609308882, 'train_speed(iter/s)': 0.533134, 'epoch': 1.0289389067524115, 'consumed_train_tokens': 2226579}

262

{'loss': 0.6066505908966064, 'learning_rate': 0.00020708039492242593, 'train_speed(iter/s)': 0.533196, 'epoch': 1.0329581993569132, 'consumed_train_tokens': 2235735}

263

{'loss': 0.5816302299499512, 'learning_rate': 0.000206657263751763, 'train_speed(iter/s)': 0.533351, 'epoch': 1.0369774919614148, 'consumed_train_tokens': 2244168}

264

{'loss': 0.6235658645629882, 'learning_rate': 0.00020623413258110011, 'train_speed(iter/s)': 0.53348, 'epoch': 1.0409967845659165, 'consumed_train_tokens': 2252974}

265

{'loss': 0.6894133567810059, 'learning_rate': 0.0002058110014104372, 'train_speed(iter/s)': 0.533651, 'epoch': 1.045016077170418, 'consumed_train_tokens': 2262156}

266

{'loss': 0.5515554904937744, 'learning_rate': 0.0002053878702397743, 'train_speed(iter/s)': 0.533754, 'epoch': 1.0490353697749195, 'consumed_train_tokens': 2271117}

267

{'loss': 0.5610471248626709, 'learning_rate': 0.00020496473906911138, 'train_speed(iter/s)': 0.533862, 'epoch': 1.0530546623794212, 'consumed_train_tokens': 2280155}

268

{'loss': 0.5338945388793945, 'learning_rate': 0.0002045416078984485, 'train_speed(iter/s)': 0.533953, 'epoch': 1.0570739549839228, 'consumed_train_tokens': 2288368}

269

{'loss': 0.6103802680969238, 'learning_rate': 0.0002041184767277856, 'train_speed(iter/s)': 0.534125, 'epoch': 1.0610932475884245, 'consumed_train_tokens': 2296931}

270

{'loss': 0.5945381164550781, 'learning_rate': 0.00020369534555712268, 'train_speed(iter/s)': 0.534186, 'epoch': 1.065112540192926, 'consumed_train_tokens': 2305753}

271

{'loss': 0.6380928039550782, 'learning_rate': 0.00020327221438645979, 'train_speed(iter/s)': 0.534288, 'epoch': 1.0691318327974277, 'consumed_train_tokens': 2314903}

272

{'loss': 0.5950185775756835, 'learning_rate': 0.00020284908321579687, 'train_speed(iter/s)': 0.534394, 'epoch': 1.0731511254019293, 'consumed_train_tokens': 2324088}

273

{'loss': 0.5513082504272461, 'learning_rate': 0.00020242595204513397, 'train_speed(iter/s)': 0.534472, 'epoch': 1.077170418006431, 'consumed_train_tokens': 2333039}

274

{'loss': 0.6278369903564454, 'learning_rate': 0.00020200282087447105, 'train_speed(iter/s)': 0.534527, 'epoch': 1.0811897106109325, 'consumed_train_tokens': 2342103}

275

{'loss': 0.5843902587890625, 'learning_rate': 0.00020157968970380816, 'train_speed(iter/s)': 0.534618, 'epoch': 1.085209003215434, 'consumed_train_tokens': 2350476}

276

{'loss': 0.6136153221130372, 'learning_rate': 0.00020115655853314524, 'train_speed(iter/s)': 0.534705, 'epoch': 1.0892282958199357, 'consumed_train_tokens': 2359446}

277

{'loss': 0.7039615154266358, 'learning_rate': 0.00020073342736248235, 'train_speed(iter/s)': 0.534872, 'epoch': 1.0932475884244373, 'consumed_train_tokens': 2368598}

278

{'loss': 0.6610403060913086, 'learning_rate': 0.00020031029619181943, 'train_speed(iter/s)': 0.535013, 'epoch': 1.097266881028939, 'consumed_train_tokens': 2377231}

279

{'loss': 0.6269991874694825, 'learning_rate': 0.00019988716502115654, 'train_speed(iter/s)': 0.535102, 'epoch': 1.1012861736334405, 'consumed_train_tokens': 2385588}

280

{'loss': 0.5356134414672852, 'learning_rate': 0.00019946403385049364, 'train_speed(iter/s)': 0.535286, 'epoch': 1.105305466237942, 'consumed_train_tokens': 2394251}

281

{'loss': 0.5797544002532959, 'learning_rate': 0.00019904090267983072, 'train_speed(iter/s)': 0.535356, 'epoch': 1.1093247588424437, 'consumed_train_tokens': 2403380}

282

{'loss': 0.6319257736206054, 'learning_rate': 0.00019861777150916783, 'train_speed(iter/s)': 0.535521, 'epoch': 1.1133440514469453, 'consumed_train_tokens': 2412402}

283

{'loss': 0.5593666076660156, 'learning_rate': 0.0001981946403385049, 'train_speed(iter/s)': 0.535684, 'epoch': 1.117363344051447, 'consumed_train_tokens': 2420978}

284

{'loss': 0.7086613178253174, 'learning_rate': 0.00019777150916784202, 'train_speed(iter/s)': 0.535871, 'epoch': 1.1213826366559485, 'consumed_train_tokens': 2429833}

285

{'loss': 0.5639712810516357, 'learning_rate': 0.0001973483779971791, 'train_speed(iter/s)': 0.536022, 'epoch': 1.1254019292604502, 'consumed_train_tokens': 2438245}

286

{'loss': 0.6861386299133301, 'learning_rate': 0.0001969252468265162, 'train_speed(iter/s)': 0.536225, 'epoch': 1.1294212218649518, 'consumed_train_tokens': 2446615}

287

{'loss': 0.6197976112365723, 'learning_rate': 0.00019650211565585329, 'train_speed(iter/s)': 0.536204, 'epoch': 1.1334405144694535, 'consumed_train_tokens': 2455484}

288

{'loss': 0.5317028045654297, 'learning_rate': 0.0001960789844851904, 'train_speed(iter/s)': 0.536346, 'epoch': 1.137459807073955, 'consumed_train_tokens': 2464550}

289

{'loss': 0.5947109222412109, 'learning_rate': 0.0001956558533145275, 'train_speed(iter/s)': 0.536393, 'epoch': 1.1414790996784565, 'consumed_train_tokens': 2473843}

290

{'loss': 0.5535234451293946, 'learning_rate': 0.00019523272214386458, 'train_speed(iter/s)': 0.53648, 'epoch': 1.1454983922829582, 'consumed_train_tokens': 2482125}

291

{'loss': 0.6227780818939209, 'learning_rate': 0.0001948095909732017, 'train_speed(iter/s)': 0.536488, 'epoch': 1.1495176848874598, 'consumed_train_tokens': 2491731}

292

{'loss': 0.6334080696105957, 'learning_rate': 0.00019438645980253877, 'train_speed(iter/s)': 0.536585, 'epoch': 1.1535369774919615, 'consumed_train_tokens': 2500441}

293

{'loss': 0.5098772048950195, 'learning_rate': 0.00019396332863187588, 'train_speed(iter/s)': 0.536627, 'epoch': 1.157556270096463, 'consumed_train_tokens': 2509570}

294

{'loss': 0.5299534797668457, 'learning_rate': 0.00019354019746121296, 'train_speed(iter/s)': 0.536631, 'epoch': 1.1615755627009645, 'consumed_train_tokens': 2518490}

295

{'loss': 0.5792361259460449, 'learning_rate': 0.00019311706629055006, 'train_speed(iter/s)': 0.536637, 'epoch': 1.1655948553054662, 'consumed_train_tokens': 2527084}

296

{'loss': 0.5964544296264649, 'learning_rate': 0.00019269393511988714, 'train_speed(iter/s)': 0.536774, 'epoch': 1.1696141479099678, 'consumed_train_tokens': 2535627}

297

{'loss': 0.614836311340332, 'learning_rate': 0.00019227080394922425, 'train_speed(iter/s)': 0.536832, 'epoch': 1.1736334405144695, 'consumed_train_tokens': 2544030}

298

{'loss': 0.5679223537445068, 'learning_rate': 0.00019184767277856136, 'train_speed(iter/s)': 0.536917, 'epoch': 1.177652733118971, 'consumed_train_tokens': 2552138}

299

{'loss': 0.6012802124023438, 'learning_rate': 0.00019142454160789844, 'train_speed(iter/s)': 0.536899, 'epoch': 1.1816720257234727, 'consumed_train_tokens': 2561029}

300

{'loss': 0.5477873802185058, 'learning_rate': 0.00019100141043723555, 'train_speed(iter/s)': 0.537032, 'epoch': 1.1856913183279743, 'consumed_train_tokens': 2569455}

301

{'loss': 0.5726665496826172, 'learning_rate': 0.00019057827926657263, 'train_speed(iter/s)': 0.537117, 'epoch': 1.189710610932476, 'consumed_train_tokens': 2577531}

302

{'loss': 0.6471715927124023, 'learning_rate': 0.0001897320169252468, 'train_speed(iter/s)': 0.537307, 'epoch': 1.197749196141479, 'consumed_train_tokens': 2595266}

303

{'loss': 0.55189208984375, 'learning_rate': 0.00018930888575458392, 'train_speed(iter/s)': 0.537411, 'epoch': 1.2017684887459807, 'consumed_train_tokens': 2603394}

304

{'loss': 0.5768201351165771, 'learning_rate': 0.000188885754583921, 'train_speed(iter/s)': 0.537429, 'epoch': 1.2057877813504823, 'consumed_train_tokens': 2612045}

305

{'loss': 0.5727392196655273, 'learning_rate': 0.0001884626234132581, 'train_speed(iter/s)': 0.537496, 'epoch': 1.209807073954984, 'consumed_train_tokens': 2620504}

306

{'loss': 0.5644481182098389, 'learning_rate': 0.0001880394922425952, 'train_speed(iter/s)': 0.537554, 'epoch': 1.2138263665594855, 'consumed_train_tokens': 2629859}

307

{'loss': 0.6561320781707763, 'learning_rate': 0.0001876163610719323, 'train_speed(iter/s)': 0.537604, 'epoch': 1.217845659163987, 'consumed_train_tokens': 2638596}

308

{'loss': 0.5618750572204589, 'learning_rate': 0.0001871932299012694, 'train_speed(iter/s)': 0.537625, 'epoch': 1.2218649517684887, 'consumed_train_tokens': 2647615}

309

{'loss': 0.582244873046875, 'learning_rate': 0.00018677009873060648, 'train_speed(iter/s)': 0.537688, 'epoch': 1.2258842443729903, 'consumed_train_tokens': 2657080}

310

{'loss': 0.5468413829803467, 'learning_rate': 0.00018634696755994354, 'train_speed(iter/s)': 0.537834, 'epoch': 1.229903536977492, 'consumed_train_tokens': 2665248}

311

{'loss': 0.5316450595855713, 'learning_rate': 0.00018592383638928064, 'train_speed(iter/s)': 0.537854, 'epoch': 1.2339228295819935, 'consumed_train_tokens': 2674166}

312

{'loss': 0.5625564575195312, 'learning_rate': 0.00018550070521861775, 'train_speed(iter/s)': 0.537969, 'epoch': 1.2379421221864952, 'consumed_train_tokens': 2683667}

313

{'loss': 0.5034240245819092, 'learning_rate': 0.00018507757404795483, 'train_speed(iter/s)': 0.537963, 'epoch': 1.2419614147909968, 'consumed_train_tokens': 2693009}

314

{'loss': 0.5757344722747803, 'learning_rate': 0.00018465444287729194, 'train_speed(iter/s)': 0.537997, 'epoch': 1.2459807073954985, 'consumed_train_tokens': 2701508}

315

{'loss': 0.5540644645690918, 'learning_rate': 0.00018423131170662902, 'train_speed(iter/s)': 0.53804, 'epoch': 1.25, 'consumed_train_tokens': 2709998}

316

{'loss': 0.6633570194244385, 'learning_rate': 0.00018380818053596613, 'train_speed(iter/s)': 0.538168, 'epoch': 1.2540192926045015, 'consumed_train_tokens': 2718325}

317

{'loss': 0.594077730178833, 'learning_rate': 0.0001833850493653032, 'train_speed(iter/s)': 0.538157, 'epoch': 1.2580385852090032, 'consumed_train_tokens': 2726589}

318

{'loss': 0.5476304531097412, 'learning_rate': 0.00018296191819464031, 'train_speed(iter/s)': 0.538251, 'epoch': 1.2620578778135048, 'consumed_train_tokens': 2734561}

319

{'loss': 0.5845719337463379, 'learning_rate': 0.0001825387870239774, 'train_speed(iter/s)': 0.538285, 'epoch': 1.2660771704180065, 'consumed_train_tokens': 2743292}

320

{'loss': 0.5611763000488281, 'learning_rate': 0.0001821156558533145, 'train_speed(iter/s)': 0.538421, 'epoch': 1.270096463022508, 'consumed_train_tokens': 2752457}

321

{'loss': 0.6438338279724121, 'learning_rate': 0.0001816925246826516, 'train_speed(iter/s)': 0.538442, 'epoch': 1.2741157556270095, 'consumed_train_tokens': 2761460}

322

{'loss': 0.6198166847229004, 'learning_rate': 0.0001812693935119887, 'train_speed(iter/s)': 0.538535, 'epoch': 1.2781350482315113, 'consumed_train_tokens': 2770190}

323

{'loss': 0.5600907802581787, 'learning_rate': 0.0001808462623413258, 'train_speed(iter/s)': 0.538494, 'epoch': 1.282154340836013, 'consumed_train_tokens': 2778144}

324

{'loss': 0.7058168411254883, 'learning_rate': 0.00018042313117066288, 'train_speed(iter/s)': 0.538591, 'epoch': 1.2861736334405145, 'consumed_train_tokens': 2786427}

325

{'loss': 0.6008402347564697, 'learning_rate': 0.00017999999999999998, 'train_speed(iter/s)': 0.538574, 'epoch': 1.290192926045016, 'consumed_train_tokens': 2794967}

326

{'loss': 0.6269058227539063, 'learning_rate': 0.00017957686882933706, 'train_speed(iter/s)': 0.53867, 'epoch': 1.2942122186495177, 'consumed_train_tokens': 2803028}

327

{'loss': 0.5590603351593018, 'learning_rate': 0.00017915373765867417, 'train_speed(iter/s)': 0.538644, 'epoch': 1.2982315112540193, 'consumed_train_tokens': 2812228}

328

{'loss': 0.5086845874786377, 'learning_rate': 0.00017873060648801125, 'train_speed(iter/s)': 0.538794, 'epoch': 1.302250803858521, 'consumed_train_tokens': 2820120}

329

{'loss': 0.5546183586120605, 'learning_rate': 0.00017830747531734836, 'train_speed(iter/s)': 0.538742, 'epoch': 1.3062700964630225, 'consumed_train_tokens': 2828412}

330

{'loss': 0.5509248733520508, 'learning_rate': 0.00017788434414668547, 'train_speed(iter/s)': 0.538807, 'epoch': 1.310289389067524, 'consumed_train_tokens': 2836917}

331

{'loss': 0.5922249317169189, 'learning_rate': 0.00017746121297602255, 'train_speed(iter/s)': 0.538732, 'epoch': 1.3143086816720257, 'consumed_train_tokens': 2845673}

332

{'loss': 0.5820208549499511, 'learning_rate': 0.00017703808180535965, 'train_speed(iter/s)': 0.538859, 'epoch': 1.3183279742765273, 'consumed_train_tokens': 2854645}

333

{'loss': 0.5792086601257325, 'learning_rate': 0.00017661495063469673, 'train_speed(iter/s)': 0.538893, 'epoch': 1.322347266881029, 'consumed_train_tokens': 2863683}

334

{'loss': 0.5834141731262207, 'learning_rate': 0.00017619181946403384, 'train_speed(iter/s)': 0.538925, 'epoch': 1.3263665594855305, 'consumed_train_tokens': 2872507}

335

{'loss': 0.5747252464294433, 'learning_rate': 0.00017576868829337092, 'train_speed(iter/s)': 0.538953, 'epoch': 1.330385852090032, 'consumed_train_tokens': 2881496}

336

{'loss': 0.5143255233764649, 'learning_rate': 0.00017534555712270803, 'train_speed(iter/s)': 0.539019, 'epoch': 1.3344051446945338, 'consumed_train_tokens': 2890205}

337

{'loss': 0.6161516666412353, 'learning_rate': 0.0001749224259520451, 'train_speed(iter/s)': 0.539027, 'epoch': 1.3384244372990355, 'consumed_train_tokens': 2898855}

338

{'loss': 0.5393246650695801, 'learning_rate': 0.00017449929478138222, 'train_speed(iter/s)': 0.539041, 'epoch': 1.342443729903537, 'consumed_train_tokens': 2907955}

339

{'loss': 0.6493902206420898, 'learning_rate': 0.0001740761636107193, 'train_speed(iter/s)': 0.538983, 'epoch': 1.3464630225080385, 'consumed_train_tokens': 2917457}

340

{'loss': 0.5976818084716797, 'learning_rate': 0.0001736530324400564, 'train_speed(iter/s)': 0.539009, 'epoch': 1.3504823151125402, 'consumed_train_tokens': 2926298}

341

{'loss': 0.6006343364715576, 'learning_rate': 0.0001732299012693935, 'train_speed(iter/s)': 0.539009, 'epoch': 1.3545016077170418, 'consumed_train_tokens': 2935154}

342

{'loss': 0.5145189762115479, 'learning_rate': 0.0001728067700987306, 'train_speed(iter/s)': 0.539015, 'epoch': 1.3585209003215435, 'consumed_train_tokens': 2944465}

343

{'loss': 0.5552654266357422, 'learning_rate': 0.0001723836389280677, 'train_speed(iter/s)': 0.53906, 'epoch': 1.362540192926045, 'consumed_train_tokens': 2953681}

344

{'loss': 0.538515567779541, 'learning_rate': 0.00017196050775740478, 'train_speed(iter/s)': 0.539039, 'epoch': 1.3665594855305465, 'consumed_train_tokens': 2961470}

345

{'loss': 0.648732852935791, 'learning_rate': 0.0001715373765867419, 'train_speed(iter/s)': 0.539065, 'epoch': 1.3705787781350482, 'consumed_train_tokens': 2970057}

346

{'loss': 0.6550267219543457, 'learning_rate': 0.00017111424541607897, 'train_speed(iter/s)': 0.538966, 'epoch': 1.3745980707395498, 'consumed_train_tokens': 2978725}

347

{'loss': 0.5165424823760987, 'learning_rate': 0.00017069111424541607, 'train_speed(iter/s)': 0.538941, 'epoch': 1.3786173633440515, 'consumed_train_tokens': 2987542}

348

{'loss': 0.5464788913726807, 'learning_rate': 0.00017026798307475315, 'train_speed(iter/s)': 0.538939, 'epoch': 1.382636655948553, 'consumed_train_tokens': 2996328}

349

{'loss': 0.6899614810943604, 'learning_rate': 0.00016984485190409026, 'train_speed(iter/s)': 0.538991, 'epoch': 1.3866559485530547, 'consumed_train_tokens': 3005438}

350

{'loss': 0.5340453147888183, 'learning_rate': 0.00016942172073342737, 'train_speed(iter/s)': 0.53895, 'epoch': 1.3906752411575563, 'consumed_train_tokens': 3013908}

351

{'loss': 0.5758454322814941, 'learning_rate': 0.00016899858956276445, 'train_speed(iter/s)': 0.538981, 'epoch': 1.394694533762058, 'consumed_train_tokens': 3022271}

352

{'loss': 0.567799186706543, 'learning_rate': 0.00016857545839210156, 'train_speed(iter/s)': 0.538938, 'epoch': 1.3987138263665595, 'consumed_train_tokens': 3030597}

353

{'loss': 0.6574516296386719, 'learning_rate': 0.00016815232722143864, 'train_speed(iter/s)': 0.53895, 'epoch': 1.402733118971061, 'consumed_train_tokens': 3039124}

354

{'loss': 0.5245522499084473, 'learning_rate': 0.00016772919605077574, 'train_speed(iter/s)': 0.538887, 'epoch': 1.4067524115755627, 'consumed_train_tokens': 3047676}

355

{'loss': 0.5871187210083008, 'learning_rate': 0.00016730606488011283, 'train_speed(iter/s)': 0.538879, 'epoch': 1.4107717041800643, 'consumed_train_tokens': 3056446}

356

{'loss': 0.5716616630554199, 'learning_rate': 0.00016688293370944993, 'train_speed(iter/s)': 0.538783, 'epoch': 1.414790996784566, 'consumed_train_tokens': 3065526}

357

{'loss': 0.5541763305664062, 'learning_rate': 0.000166459802538787, 'train_speed(iter/s)': 0.538802, 'epoch': 1.4188102893890675, 'consumed_train_tokens': 3074442}

358

{'loss': 0.6200732231140137, 'learning_rate': 0.00016603667136812412, 'train_speed(iter/s)': 0.538735, 'epoch': 1.422829581993569, 'consumed_train_tokens': 3083469}

359

{'loss': 0.6439158916473389, 'learning_rate': 0.00016561354019746123, 'train_speed(iter/s)': 0.538719, 'epoch': 1.4268488745980707, 'consumed_train_tokens': 3092542}

360

{'loss': 0.643857479095459, 'learning_rate': 0.0001651904090267983, 'train_speed(iter/s)': 0.538688, 'epoch': 1.4308681672025725, 'consumed_train_tokens': 3100976}

361

{'loss': 0.540685510635376, 'learning_rate': 0.00016476727785613536, 'train_speed(iter/s)': 0.53867, 'epoch': 1.434887459807074, 'consumed_train_tokens': 3109087}

362

{'loss': 0.6279495239257813, 'learning_rate': 0.00016434414668547247, 'train_speed(iter/s)': 0.538649, 'epoch': 1.4389067524115755, 'consumed_train_tokens': 3117964}

363

{'loss': 0.6624514579772949, 'learning_rate': 0.00016392101551480955, 'train_speed(iter/s)': 0.538678, 'epoch': 1.4429260450160772, 'consumed_train_tokens': 3126516}

364

{'loss': 0.5907713890075683, 'learning_rate': 0.00016349788434414666, 'train_speed(iter/s)': 0.538653, 'epoch': 1.4469453376205788, 'consumed_train_tokens': 3134782}

365

{'loss': 0.5918978214263916, 'learning_rate': 0.00016307475317348376, 'train_speed(iter/s)': 0.538659, 'epoch': 1.4509646302250805, 'consumed_train_tokens': 3143406}

366

{'loss': 0.6073541641235352, 'learning_rate': 0.00016265162200282084, 'train_speed(iter/s)': 0.538598, 'epoch': 1.454983922829582, 'consumed_train_tokens': 3152466}

367

{'loss': 0.6223671913146973, 'learning_rate': 0.00016222849083215795, 'train_speed(iter/s)': 0.53859, 'epoch': 1.4590032154340835, 'consumed_train_tokens': 3160905}

368

{'loss': 0.5266454696655274, 'learning_rate': 0.00016180535966149503, 'train_speed(iter/s)': 0.538506, 'epoch': 1.4630225080385852, 'consumed_train_tokens': 3169566}

369

{'loss': 0.5984995841979981, 'learning_rate': 0.00016138222849083214, 'train_speed(iter/s)': 0.538519, 'epoch': 1.4670418006430868, 'consumed_train_tokens': 3178009}

370

{'loss': 0.5854095935821533, 'learning_rate': 0.00016095909732016922, 'train_speed(iter/s)': 0.538484, 'epoch': 1.4710610932475885, 'consumed_train_tokens': 3185833}

371

{'loss': 0.636417293548584, 'learning_rate': 0.00016053596614950633, 'train_speed(iter/s)': 0.538473, 'epoch': 1.47508038585209, 'consumed_train_tokens': 3194873}

372

{'loss': 0.5836258411407471, 'learning_rate': 0.0001601128349788434, 'train_speed(iter/s)': 0.538425, 'epoch': 1.4790996784565915, 'consumed_train_tokens': 3203812}

373

{'loss': 0.5974038600921631, 'learning_rate': 0.0001596897038081805, 'train_speed(iter/s)': 0.538424, 'epoch': 1.4831189710610932, 'consumed_train_tokens': 3213021}

374

{'loss': 0.7031189918518066, 'learning_rate': 0.00015926657263751762, 'train_speed(iter/s)': 0.538431, 'epoch': 1.487138263665595, 'consumed_train_tokens': 3221507}

375

{'loss': 0.5858002662658691, 'learning_rate': 0.0001588434414668547, 'train_speed(iter/s)': 0.538422, 'epoch': 1.4911575562700965, 'consumed_train_tokens': 3230380}

376

{'loss': 0.6891232967376709, 'learning_rate': 0.0001579971791255289, 'train_speed(iter/s)': 0.538389, 'epoch': 1.4991961414790997, 'consumed_train_tokens': 3247157}

377

{'loss': 0.5878059387207031, 'learning_rate': 0.000157574047954866, 'train_speed(iter/s)': 0.538363, 'epoch': 1.5032154340836013, 'consumed_train_tokens': 3255130}

378

{'loss': 0.6012544631958008, 'learning_rate': 0.00015715091678420308, 'train_speed(iter/s)': 0.538341, 'epoch': 1.507234726688103, 'consumed_train_tokens': 3264027}

379

{'loss': 0.6523089408874512, 'learning_rate': 0.00015672778561354018, 'train_speed(iter/s)': 0.538321, 'epoch': 1.5112540192926045, 'consumed_train_tokens': 3272495}

380

{'loss': 0.6190018653869629, 'learning_rate': 0.00015630465444287726, 'train_speed(iter/s)': 0.538315, 'epoch': 1.515273311897106, 'consumed_train_tokens': 3281231}

381

{'loss': 0.6242691993713378, 'learning_rate': 0.00015588152327221437, 'train_speed(iter/s)': 0.538319, 'epoch': 1.5192926045016077, 'consumed_train_tokens': 3289741}

382

{'loss': 0.589319896697998, 'learning_rate': 0.00015545839210155148, 'train_speed(iter/s)': 0.538326, 'epoch': 1.5233118971061095, 'consumed_train_tokens': 3298232}

383

{'loss': 0.6069149494171142, 'learning_rate': 0.00015503526093088856, 'train_speed(iter/s)': 0.538306, 'epoch': 1.527331189710611, 'consumed_train_tokens': 3307081}

384

{'loss': 0.6065144538879395, 'learning_rate': 0.00015461212976022567, 'train_speed(iter/s)': 0.538251, 'epoch': 1.5313504823151125, 'consumed_train_tokens': 3316395}

385

{'loss': 0.6436751365661622, 'learning_rate': 0.00015418899858956275, 'train_speed(iter/s)': 0.53827, 'epoch': 1.535369774919614, 'consumed_train_tokens': 3325359}

386

{'loss': 0.6000955104827881, 'learning_rate': 0.00015376586741889985, 'train_speed(iter/s)': 0.538213, 'epoch': 1.5393890675241158, 'consumed_train_tokens': 3333749}

387

{'loss': 0.6540597915649414, 'learning_rate': 0.00015334273624823693, 'train_speed(iter/s)': 0.538193, 'epoch': 1.5434083601286175, 'consumed_train_tokens': 3342974}

388

{'loss': 0.4832716941833496, 'learning_rate': 0.00015291960507757404, 'train_speed(iter/s)': 0.538151, 'epoch': 1.547427652733119, 'consumed_train_tokens': 3351442}

389

{'loss': 0.49907970428466797, 'learning_rate': 0.00015249647390691112, 'train_speed(iter/s)': 0.538129, 'epoch': 1.5514469453376205, 'consumed_train_tokens': 3359716}

390

{'loss': 0.6296504020690918, 'learning_rate': 0.00015207334273624823, 'train_speed(iter/s)': 0.538065, 'epoch': 1.555466237942122, 'consumed_train_tokens': 3368315}

391

{'loss': 0.6034518718719483, 'learning_rate': 0.0001516502115655853, 'train_speed(iter/s)': 0.538042, 'epoch': 1.5594855305466238, 'consumed_train_tokens': 3376470}

392

{'loss': 0.5931142330169678, 'learning_rate': 0.00015122708039492242, 'train_speed(iter/s)': 0.538029, 'epoch': 1.5635048231511255, 'consumed_train_tokens': 3385602}

393

{'loss': 0.5845873832702637, 'learning_rate': 0.00015080394922425952, 'train_speed(iter/s)': 0.538009, 'epoch': 1.567524115755627, 'consumed_train_tokens': 3394404}

394

{'loss': 0.5828464031219482, 'learning_rate': 0.0001503808180535966, 'train_speed(iter/s)': 0.538, 'epoch': 1.5715434083601285, 'consumed_train_tokens': 3403226}

395

{'loss': 0.5175209045410156, 'learning_rate': 0.0001499576868829337, 'train_speed(iter/s)': 0.537964, 'epoch': 1.5755627009646302, 'consumed_train_tokens': 3411563}

396

{'loss': 0.5812587738037109, 'learning_rate': 0.0001495345557122708, 'train_speed(iter/s)': 0.537975, 'epoch': 1.579581993569132, 'consumed_train_tokens': 3420215}

397

{'loss': 0.6046332359313965, 'learning_rate': 0.0001491114245416079, 'train_speed(iter/s)': 0.537944, 'epoch': 1.5836012861736335, 'consumed_train_tokens': 3430632}

398

{'loss': 0.5521742343902588, 'learning_rate': 0.00014868829337094498, 'train_speed(iter/s)': 0.53789, 'epoch': 1.587620578778135, 'consumed_train_tokens': 3439681}

399

{'loss': 0.5691207885742188, 'learning_rate': 0.00014826516220028206, 'train_speed(iter/s)': 0.537897, 'epoch': 1.5916398713826365, 'consumed_train_tokens': 3448092}

400

{'loss': 0.5753486633300782, 'learning_rate': 0.00014784203102961917, 'train_speed(iter/s)': 0.537808, 'epoch': 1.5956591639871383, 'consumed_train_tokens': 3456958}

401

{'loss': 0.6049731254577637, 'learning_rate': 0.00014741889985895625, 'train_speed(iter/s)': 0.537828, 'epoch': 1.59967845659164, 'consumed_train_tokens': 3466078}

402

{'loss': 0.5679505348205567, 'learning_rate': 0.00014699576868829335, 'train_speed(iter/s)': 0.537768, 'epoch': 1.6036977491961415, 'consumed_train_tokens': 3474536}

403

{'loss': 0.4728222846984863, 'learning_rate': 0.00014657263751763046, 'train_speed(iter/s)': 0.537742, 'epoch': 1.607717041800643, 'consumed_train_tokens': 3482809}

404

{'loss': 0.5487497329711915, 'learning_rate': 0.00014614950634696754, 'train_speed(iter/s)': 0.537693, 'epoch': 1.6117363344051447, 'consumed_train_tokens': 3491644}

405

{'loss': 0.49289932250976565, 'learning_rate': 0.00014572637517630465, 'train_speed(iter/s)': 0.537714, 'epoch': 1.6157556270096463, 'consumed_train_tokens': 3500752}

406

{'loss': 0.5877913951873779, 'learning_rate': 0.00014530324400564173, 'train_speed(iter/s)': 0.537679, 'epoch': 1.619774919614148, 'consumed_train_tokens': 3509060}

407

{'loss': 0.6191031455993652, 'learning_rate': 0.00014488011283497884, 'train_speed(iter/s)': 0.537698, 'epoch': 1.6237942122186495, 'consumed_train_tokens': 3516968}

408

{'loss': 0.575957202911377, 'learning_rate': 0.00014445698166431592, 'train_speed(iter/s)': 0.53768, 'epoch': 1.627813504823151, 'consumed_train_tokens': 3525560}

409

{'loss': 0.5792960166931153, 'learning_rate': 0.00014403385049365302, 'train_speed(iter/s)': 0.537699, 'epoch': 1.6318327974276527, 'consumed_train_tokens': 3534295}

410

{'loss': 0.5752998352050781, 'learning_rate': 0.0001436107193229901, 'train_speed(iter/s)': 0.537678, 'epoch': 1.6358520900321545, 'consumed_train_tokens': 3543020}

411

{'loss': 0.6120525360107422, 'learning_rate': 0.0001431875881523272, 'train_speed(iter/s)': 0.53768, 'epoch': 1.639871382636656, 'consumed_train_tokens': 3552074}

412

{'loss': 0.6148652553558349, 'learning_rate': 0.0001427644569816643, 'train_speed(iter/s)': 0.537653, 'epoch': 1.6438906752411575, 'consumed_train_tokens': 3560742}

413

{'loss': 0.5838727951049805, 'learning_rate': 0.0001423413258110014, 'train_speed(iter/s)': 0.53761, 'epoch': 1.647909967845659, 'consumed_train_tokens': 3569257}

414

{'loss': 0.6248235702514648, 'learning_rate': 0.0001419181946403385, 'train_speed(iter/s)': 0.537609, 'epoch': 1.6519292604501608, 'consumed_train_tokens': 3577005}

415

{'loss': 0.5919002056121826, 'learning_rate': 0.0001414950634696756, 'train_speed(iter/s)': 0.537631, 'epoch': 1.6559485530546625, 'consumed_train_tokens': 3585463}

416

{'loss': 0.5870795249938965, 'learning_rate': 0.0001410719322990127, 'train_speed(iter/s)': 0.537619, 'epoch': 1.659967845659164, 'consumed_train_tokens': 3594961}

417

{'loss': 0.6068478584289551, 'learning_rate': 0.00014064880112834977, 'train_speed(iter/s)': 0.537652, 'epoch': 1.6639871382636655, 'consumed_train_tokens': 3603360}

418

{'loss': 0.5496441841125488, 'learning_rate': 0.00014022566995768688, 'train_speed(iter/s)': 0.537616, 'epoch': 1.6680064308681672, 'consumed_train_tokens': 3611642}

419

{'loss': 0.5715486526489257, 'learning_rate': 0.00013980253878702396, 'train_speed(iter/s)': 0.537611, 'epoch': 1.6720257234726688, 'consumed_train_tokens': 3619930}

420

{'loss': 0.6042613506317138, 'learning_rate': 0.00013937940761636107, 'train_speed(iter/s)': 0.537598, 'epoch': 1.6760450160771705, 'consumed_train_tokens': 3628899}

421

{'loss': 0.6228303909301758, 'learning_rate': 0.00013895627644569815, 'train_speed(iter/s)': 0.537555, 'epoch': 1.680064308681672, 'consumed_train_tokens': 3637636}

422

{'loss': 0.56146240234375, 'learning_rate': 0.00013853314527503526, 'train_speed(iter/s)': 0.537543, 'epoch': 1.6840836012861735, 'consumed_train_tokens': 3646032}

423

{'loss': 0.5911332130432129, 'learning_rate': 0.00013811001410437234, 'train_speed(iter/s)': 0.537505, 'epoch': 1.6881028938906752, 'consumed_train_tokens': 3655197}

424

{'loss': 0.5481966972351074, 'learning_rate': 0.00013768688293370942, 'train_speed(iter/s)': 0.537497, 'epoch': 1.692122186495177, 'consumed_train_tokens': 3663622}

425

{'loss': 0.5936191558837891, 'learning_rate': 0.00013726375176304652, 'train_speed(iter/s)': 0.537454, 'epoch': 1.6961414790996785, 'consumed_train_tokens': 3672052}

426

{'loss': 0.5462120056152344, 'learning_rate': 0.00013684062059238363, 'train_speed(iter/s)': 0.537439, 'epoch': 1.70016077170418, 'consumed_train_tokens': 3681199}

427

{'loss': 0.6101339340209961, 'learning_rate': 0.0001364174894217207, 'train_speed(iter/s)': 0.537383, 'epoch': 1.7041800643086815, 'consumed_train_tokens': 3691023}

428

{'loss': 0.5948652267456055, 'learning_rate': 0.00013599435825105782, 'train_speed(iter/s)': 0.537335, 'epoch': 1.7081993569131833, 'consumed_train_tokens': 3699765}

429

{'loss': 0.6346423149108886, 'learning_rate': 0.0001355712270803949, 'train_speed(iter/s)': 0.537323, 'epoch': 1.712218649517685, 'consumed_train_tokens': 3708362}

430

{'loss': 0.570752763748169, 'learning_rate': 0.000135148095909732, 'train_speed(iter/s)': 0.537315, 'epoch': 1.7162379421221865, 'consumed_train_tokens': 3717298}

431

{'loss': 0.5941257953643799, 'learning_rate': 0.0001347249647390691, 'train_speed(iter/s)': 0.537269, 'epoch': 1.720257234726688, 'consumed_train_tokens': 3725802}

432

{'loss': 0.5378665447235107, 'learning_rate': 0.0001343018335684062, 'train_speed(iter/s)': 0.537262, 'epoch': 1.7242765273311897, 'consumed_train_tokens': 3733770}

433

{'loss': 0.46761503219604494, 'learning_rate': 0.00013387870239774328, 'train_speed(iter/s)': 0.537187, 'epoch': 1.7282958199356915, 'consumed_train_tokens': 3741905}

434

{'loss': 0.5954689025878906, 'learning_rate': 0.00013345557122708038, 'train_speed(iter/s)': 0.537182, 'epoch': 1.732315112540193, 'consumed_train_tokens': 3750108}

435

{'loss': 0.6398868083953857, 'learning_rate': 0.0001330324400564175, 'train_speed(iter/s)': 0.53716, 'epoch': 1.7363344051446945, 'consumed_train_tokens': 3758380}

436

{'loss': 0.660976791381836, 'learning_rate': 0.00013260930888575457, 'train_speed(iter/s)': 0.537176, 'epoch': 1.740353697749196, 'consumed_train_tokens': 3767047}

437

{'loss': 0.6059123039245605, 'learning_rate': 0.00013218617771509168, 'train_speed(iter/s)': 0.537126, 'epoch': 1.7443729903536977, 'consumed_train_tokens': 3775430}

438

{'loss': 0.5819013595581055, 'learning_rate': 0.00013176304654442876, 'train_speed(iter/s)': 0.537146, 'epoch': 1.7483922829581995, 'consumed_train_tokens': 3783806}

439

{'loss': 0.6179468154907226, 'learning_rate': 0.00013133991537376587, 'train_speed(iter/s)': 0.537096, 'epoch': 1.752411575562701, 'consumed_train_tokens': 3792830}

440

{'loss': 0.4682276725769043, 'learning_rate': 0.00013091678420310295, 'train_speed(iter/s)': 0.537089, 'epoch': 1.7564308681672025, 'consumed_train_tokens': 3800606}

441

{'loss': 0.5942191123962403, 'learning_rate': 0.00013049365303244005, 'train_speed(iter/s)': 0.537058, 'epoch': 1.760450160771704, 'consumed_train_tokens': 3808803}

442

{'loss': 0.6023986339569092, 'learning_rate': 0.00013007052186177713, 'train_speed(iter/s)': 0.53708, 'epoch': 1.7644694533762058, 'consumed_train_tokens': 3817548}

443

{'loss': 0.4891871452331543, 'learning_rate': 0.00012964739069111424, 'train_speed(iter/s)': 0.537019, 'epoch': 1.7684887459807075, 'consumed_train_tokens': 3827055}

444

{'loss': 0.5555449485778808, 'learning_rate': 0.00012922425952045135, 'train_speed(iter/s)': 0.537021, 'epoch': 1.772508038585209, 'consumed_train_tokens': 3835155}

445

{'loss': 0.6163487434387207, 'learning_rate': 0.00012880112834978843, 'train_speed(iter/s)': 0.53698, 'epoch': 1.7765273311897105, 'consumed_train_tokens': 3844185}

446

{'loss': 0.6464570999145508, 'learning_rate': 0.00012837799717912554, 'train_speed(iter/s)': 0.536991, 'epoch': 1.7805466237942122, 'consumed_train_tokens': 3853138}

447

{'loss': 0.6586458206176757, 'learning_rate': 0.00012795486600846262, 'train_speed(iter/s)': 0.536914, 'epoch': 1.784565916398714, 'consumed_train_tokens': 3861948}

448

{'loss': 0.6387148857116699, 'learning_rate': 0.00012753173483779972, 'train_speed(iter/s)': 0.536897, 'epoch': 1.7885852090032155, 'consumed_train_tokens': 3870507}

449

{'loss': 0.6105431079864502, 'learning_rate': 0.0001271086036671368, 'train_speed(iter/s)': 0.536853, 'epoch': 1.792604501607717, 'consumed_train_tokens': 3878998}

450

{'loss': 0.5872406959533691, 'learning_rate': 0.00012668547249647388, 'train_speed(iter/s)': 0.536862, 'epoch': 1.7966237942122185, 'consumed_train_tokens': 3887277}

451

{'loss': 0.6152979373931885, 'learning_rate': 0.000126262341325811, 'train_speed(iter/s)': 0.536796, 'epoch': 1.8006430868167203, 'consumed_train_tokens': 3895740}

452

{'loss': 0.5455400466918945, 'learning_rate': 0.00012583921015514807, 'train_speed(iter/s)': 0.536804, 'epoch': 1.804662379421222, 'consumed_train_tokens': 3904133}

453

{'loss': 0.5454126834869385, 'learning_rate': 0.00012541607898448518, 'train_speed(iter/s)': 0.536757, 'epoch': 1.8086816720257235, 'consumed_train_tokens': 3912508}

454

{'loss': 0.5937404632568359, 'learning_rate': 0.00012499294781382226, 'train_speed(iter/s)': 0.536761, 'epoch': 1.812700964630225, 'consumed_train_tokens': 3920807}

455

{'loss': 0.5161521911621094, 'learning_rate': 0.00012456981664315937, 'train_speed(iter/s)': 0.536749, 'epoch': 1.8167202572347267, 'consumed_train_tokens': 3928889}

456

{'loss': 0.6508796215057373, 'learning_rate': 0.00012414668547249647, 'train_speed(iter/s)': 0.536736, 'epoch': 1.8207395498392283, 'consumed_train_tokens': 3937836}

457

{'loss': 0.6002727031707764, 'learning_rate': 0.00012372355430183355, 'train_speed(iter/s)': 0.53672, 'epoch': 1.82475884244373, 'consumed_train_tokens': 3946598}

458

{'loss': 0.574857759475708, 'learning_rate': 0.00012330042313117066, 'train_speed(iter/s)': 0.536722, 'epoch': 1.8287781350482315, 'consumed_train_tokens': 3955753}

459

{'loss': 0.5542935371398926, 'learning_rate': 0.00012287729196050774, 'train_speed(iter/s)': 0.536683, 'epoch': 1.832797427652733, 'consumed_train_tokens': 3964014}

460

{'loss': 0.609438133239746, 'learning_rate': 0.00012245416078984485, 'train_speed(iter/s)': 0.536689, 'epoch': 1.8368167202572347, 'consumed_train_tokens': 3972589}

461

{'loss': 0.6374025344848633, 'learning_rate': 0.00012203102961918194, 'train_speed(iter/s)': 0.536687, 'epoch': 1.8408360128617365, 'consumed_train_tokens': 3982166}

462

{'loss': 0.5799615859985352, 'learning_rate': 0.00012160789844851904, 'train_speed(iter/s)': 0.536671, 'epoch': 1.844855305466238, 'consumed_train_tokens': 3990875}

463

{'loss': 0.5616467475891114, 'learning_rate': 0.00012118476727785613, 'train_speed(iter/s)': 0.536616, 'epoch': 1.8488745980707395, 'consumed_train_tokens': 3999822}

464

{'loss': 0.5260744094848633, 'learning_rate': 0.00012076163610719322, 'train_speed(iter/s)': 0.53664, 'epoch': 1.852893890675241, 'consumed_train_tokens': 4007956}

465

{'loss': 0.573643684387207, 'learning_rate': 0.00012033850493653032, 'train_speed(iter/s)': 0.536662, 'epoch': 1.8569131832797428, 'consumed_train_tokens': 4016982}

466

{'loss': 0.658806324005127, 'learning_rate': 0.00011991537376586741, 'train_speed(iter/s)': 0.536691, 'epoch': 1.8609324758842445, 'consumed_train_tokens': 4025771}

467

{'loss': 0.5335788249969482, 'learning_rate': 0.0001194922425952045, 'train_speed(iter/s)': 0.536692, 'epoch': 1.864951768488746, 'consumed_train_tokens': 4034334}

468

{'loss': 0.5546660423278809, 'learning_rate': 0.0001190691114245416, 'train_speed(iter/s)': 0.536708, 'epoch': 1.8689710610932475, 'consumed_train_tokens': 4043008}

469

{'loss': 0.5269043922424317, 'learning_rate': 0.00011864598025387869, 'train_speed(iter/s)': 0.536677, 'epoch': 1.8729903536977492, 'consumed_train_tokens': 4052503}

470

{'loss': 0.5869142532348632, 'learning_rate': 0.00011822284908321579, 'train_speed(iter/s)': 0.536679, 'epoch': 1.8770096463022508, 'consumed_train_tokens': 4061685}

471

{'loss': 0.6239783287048339, 'learning_rate': 0.0001177997179125529, 'train_speed(iter/s)': 0.536703, 'epoch': 1.8810289389067525, 'consumed_train_tokens': 4071071}

472

{'loss': 0.5772471904754639, 'learning_rate': 0.00011737658674188999, 'train_speed(iter/s)': 0.536678, 'epoch': 1.885048231511254, 'consumed_train_tokens': 4079890}

473

{'loss': 0.5676973819732666, 'learning_rate': 0.00011695345557122708, 'train_speed(iter/s)': 0.536688, 'epoch': 1.8890675241157555, 'consumed_train_tokens': 4088906}

474

{'loss': 0.586322021484375, 'learning_rate': 0.00011653032440056416, 'train_speed(iter/s)': 0.536668, 'epoch': 1.8930868167202572, 'consumed_train_tokens': 4097543}

475

{'loss': 0.5244094371795655, 'learning_rate': 0.00011610719322990126, 'train_speed(iter/s)': 0.536636, 'epoch': 1.897106109324759, 'consumed_train_tokens': 4107059}

476

{'loss': 0.6107178688049316, 'learning_rate': 0.00011568406205923835, 'train_speed(iter/s)': 0.536587, 'epoch': 1.9011254019292605, 'consumed_train_tokens': 4115876}

477

{'loss': 0.5332762718200683, 'learning_rate': 0.00011526093088857544, 'train_speed(iter/s)': 0.536608, 'epoch': 1.905144694533762, 'consumed_train_tokens': 4123680}

478

{'loss': 0.5929516792297364, 'learning_rate': 0.00011483779971791254, 'train_speed(iter/s)': 0.536568, 'epoch': 1.9091639871382635, 'consumed_train_tokens': 4132490}

479

{'loss': 0.6496650218963623, 'learning_rate': 0.00011441466854724963, 'train_speed(iter/s)': 0.536581, 'epoch': 1.9131832797427653, 'consumed_train_tokens': 4141706}

480

{'loss': 0.5728755474090577, 'learning_rate': 0.00011399153737658672, 'train_speed(iter/s)': 0.536525, 'epoch': 1.917202572347267, 'consumed_train_tokens': 4150754}

481

{'loss': 0.5742472648620606, 'learning_rate': 0.00011356840620592382, 'train_speed(iter/s)': 0.536536, 'epoch': 1.9212218649517685, 'consumed_train_tokens': 4159676}

482

{'loss': 0.5088113784790039, 'learning_rate': 0.00011314527503526091, 'train_speed(iter/s)': 0.536516, 'epoch': 1.92524115755627, 'consumed_train_tokens': 4168788}

483

{'loss': 0.5732627868652344, 'learning_rate': 0.00011272214386459802, 'train_speed(iter/s)': 0.536529, 'epoch': 1.9292604501607717, 'consumed_train_tokens': 4177568}

484

{'loss': 0.5228656768798828, 'learning_rate': 0.0001118758815232722, 'train_speed(iter/s)': 0.536473, 'epoch': 1.937299035369775, 'consumed_train_tokens': 4194923}

485

{'loss': 0.6763626098632812, 'learning_rate': 0.0001114527503526093, 'train_speed(iter/s)': 0.536472, 'epoch': 1.9413183279742765, 'consumed_train_tokens': 4203738}

486

{'loss': 0.5663994312286377, 'learning_rate': 0.0001110296191819464, 'train_speed(iter/s)': 0.536465, 'epoch': 1.945337620578778, 'consumed_train_tokens': 4213144}

487

{'loss': 0.5777236938476562, 'learning_rate': 0.00011060648801128349, 'train_speed(iter/s)': 0.536415, 'epoch': 1.9493569131832797, 'consumed_train_tokens': 4221994}

488

{'loss': 0.5987288475036621, 'learning_rate': 0.00011018335684062058, 'train_speed(iter/s)': 0.536391, 'epoch': 1.9533762057877815, 'consumed_train_tokens': 4230418}

489

{'loss': 0.5102124214172363, 'learning_rate': 0.00010976022566995768, 'train_speed(iter/s)': 0.536385, 'epoch': 1.957395498392283, 'consumed_train_tokens': 4238901}

490

{'loss': 0.5473896026611328, 'learning_rate': 0.00010933709449929477, 'train_speed(iter/s)': 0.536386, 'epoch': 1.9614147909967845, 'consumed_train_tokens': 4247583}

491

{'loss': 0.5376116752624511, 'learning_rate': 0.00010891396332863188, 'train_speed(iter/s)': 0.536374, 'epoch': 1.965434083601286, 'consumed_train_tokens': 4255821}

492

{'loss': 0.5437031269073487, 'learning_rate': 0.00010849083215796897, 'train_speed(iter/s)': 0.53637, 'epoch': 1.9694533762057878, 'consumed_train_tokens': 4263990}

493

{'loss': 0.501892375946045, 'learning_rate': 0.00010806770098730606, 'train_speed(iter/s)': 0.536382, 'epoch': 1.9734726688102895, 'consumed_train_tokens': 4272037}

494

{'loss': 0.6061077117919922, 'learning_rate': 0.00010764456981664316, 'train_speed(iter/s)': 0.536355, 'epoch': 1.977491961414791, 'consumed_train_tokens': 4280462}

495

{'loss': 0.6234444618225098, 'learning_rate': 0.00010722143864598025, 'train_speed(iter/s)': 0.536365, 'epoch': 1.9815112540192925, 'consumed_train_tokens': 4289215}

496

{'loss': 0.6615960121154785, 'learning_rate': 0.00010679830747531735, 'train_speed(iter/s)': 0.53633, 'epoch': 1.9855305466237942, 'consumed_train_tokens': 4299084}

497

{'loss': 0.6010730743408204, 'learning_rate': 0.00010637517630465444, 'train_speed(iter/s)': 0.536317, 'epoch': 1.989549839228296, 'consumed_train_tokens': 4308041}

498

{'loss': 0.5695746421813965, 'learning_rate': 0.00010595204513399152, 'train_speed(iter/s)': 0.536317, 'epoch': 1.9935691318327975, 'consumed_train_tokens': 4316447}

499

{'loss': 0.5629600524902344, 'learning_rate': 0.00010552891396332861, 'train_speed(iter/s)': 0.536355, 'epoch': 1.997588424437299, 'consumed_train_tokens': 4325690}

500

{'eval_loss': 0.7414682507514954, 'eval_runtime': 18.4881, 'eval_samples_per_second': 54.089, 'eval_steps_per_second': 13.522, 'epoch': 2.0}

501

{'loss': 0.49610462188720705, 'learning_rate': 0.00010510578279266571, 'train_speed(iter/s)': 0.534121, 'epoch': 2.0016077170418005, 'consumed_train_tokens': 4334457}

502

{'loss': 0.39971959590911865, 'learning_rate': 0.0001046826516220028, 'train_speed(iter/s)': 0.53412, 'epoch': 2.0056270096463025, 'consumed_train_tokens': 4342987}

503

{'loss': 0.4134061813354492, 'learning_rate': 0.0001042595204513399, 'train_speed(iter/s)': 0.534184, 'epoch': 2.009646302250804, 'consumed_train_tokens': 4352195}

504

{'loss': 0.43750638961791993, 'learning_rate': 0.000103836389280677, 'train_speed(iter/s)': 0.534238, 'epoch': 2.0136655948553055, 'consumed_train_tokens': 4360851}

505

{'loss': 0.3893228769302368, 'learning_rate': 0.0001034132581100141, 'train_speed(iter/s)': 0.534317, 'epoch': 2.017684887459807, 'consumed_train_tokens': 4369327}

506

{'loss': 0.40632266998291017, 'learning_rate': 0.00010299012693935119, 'train_speed(iter/s)': 0.534352, 'epoch': 2.0217041800643085, 'consumed_train_tokens': 4377786}

507

{'loss': 0.43276515007019045, 'learning_rate': 0.00010256699576868828, 'train_speed(iter/s)': 0.534423, 'epoch': 2.0257234726688105, 'consumed_train_tokens': 4386262}

508

{'loss': 0.3243396520614624, 'learning_rate': 0.00010214386459802538, 'train_speed(iter/s)': 0.534389, 'epoch': 2.029742765273312, 'consumed_train_tokens': 4394191}

509

{'loss': 0.36840996742248533, 'learning_rate': 0.00010172073342736247, 'train_speed(iter/s)': 0.534446, 'epoch': 2.0337620578778135, 'consumed_train_tokens': 4404021}

510

{'loss': 0.3503280162811279, 'learning_rate': 0.00010129760225669956, 'train_speed(iter/s)': 0.534514, 'epoch': 2.037781350482315, 'consumed_train_tokens': 4412865}

511

{'loss': 0.4161827087402344, 'learning_rate': 0.00010087447108603666, 'train_speed(iter/s)': 0.53457, 'epoch': 2.0418006430868165, 'consumed_train_tokens': 4421446}

512

{'loss': 0.3733147144317627, 'learning_rate': 0.00010045133991537375, 'train_speed(iter/s)': 0.534651, 'epoch': 2.0458199356913185, 'consumed_train_tokens': 4429960}

513

{'loss': 0.40694689750671387, 'learning_rate': 0.00010002820874471085, 'train_speed(iter/s)': 0.534647, 'epoch': 2.04983922829582, 'consumed_train_tokens': 4438577}

514

{'loss': 0.3814934253692627, 'learning_rate': 9.960507757404795e-05, 'train_speed(iter/s)': 0.534725, 'epoch': 2.0538585209003215, 'consumed_train_tokens': 4447289}

515

{'loss': 0.4075164318084717, 'learning_rate': 9.918194640338505e-05, 'train_speed(iter/s)': 0.534759, 'epoch': 2.057877813504823, 'consumed_train_tokens': 4455957}

516

{'loss': 0.425286865234375, 'learning_rate': 9.875881523272214e-05, 'train_speed(iter/s)': 0.534826, 'epoch': 2.061897106109325, 'consumed_train_tokens': 4465337}

517

{'loss': 0.2405923128128052, 'learning_rate': 9.833568406205923e-05, 'train_speed(iter/s)': 0.534824, 'epoch': 2.0659163987138265, 'consumed_train_tokens': 4474749}

518

{'loss': 0.3977768898010254, 'learning_rate': 9.791255289139633e-05, 'train_speed(iter/s)': 0.534888, 'epoch': 2.069935691318328, 'consumed_train_tokens': 4483356}

519

{'loss': 0.4353185653686523, 'learning_rate': 9.748942172073342e-05, 'train_speed(iter/s)': 0.534898, 'epoch': 2.0739549839228295, 'consumed_train_tokens': 4492301}

520

{'loss': 0.3955967903137207, 'learning_rate': 9.706629055007052e-05, 'train_speed(iter/s)': 0.534969, 'epoch': 2.077974276527331, 'consumed_train_tokens': 4500833}

521

{'loss': 0.39546339511871337, 'learning_rate': 9.664315937940761e-05, 'train_speed(iter/s)': 0.534987, 'epoch': 2.081993569131833, 'consumed_train_tokens': 4509015}

522

{'loss': 0.3016445398330688, 'learning_rate': 9.62200282087447e-05, 'train_speed(iter/s)': 0.535046, 'epoch': 2.0860128617363345, 'consumed_train_tokens': 4517259}

523

{'loss': 0.32941784858703616, 'learning_rate': 9.579689703808181e-05, 'train_speed(iter/s)': 0.535004, 'epoch': 2.090032154340836, 'consumed_train_tokens': 4525969}

524

{'loss': 0.37445430755615233, 'learning_rate': 9.53737658674189e-05, 'train_speed(iter/s)': 0.535056, 'epoch': 2.0940514469453375, 'consumed_train_tokens': 4534377}

525

{'loss': 0.37208008766174316, 'learning_rate': 9.495063469675597e-05, 'train_speed(iter/s)': 0.535025, 'epoch': 2.098070739549839, 'consumed_train_tokens': 4543418}

526

{'loss': 0.44919352531433104, 'learning_rate': 9.452750352609308e-05, 'train_speed(iter/s)': 0.535056, 'epoch': 2.102090032154341, 'consumed_train_tokens': 4552126}

527

{'loss': 0.38172314167022703, 'learning_rate': 9.410437235543017e-05, 'train_speed(iter/s)': 0.535076, 'epoch': 2.1061093247588425, 'consumed_train_tokens': 4561234}

528

{'loss': 0.41310997009277345, 'learning_rate': 9.368124118476727e-05, 'train_speed(iter/s)': 0.535139, 'epoch': 2.110128617363344, 'consumed_train_tokens': 4569302}

529

{'loss': 0.3186774730682373, 'learning_rate': 9.325811001410436e-05, 'train_speed(iter/s)': 0.535133, 'epoch': 2.1141479099678455, 'consumed_train_tokens': 4577922}

530

{'loss': 0.2764899730682373, 'learning_rate': 9.283497884344145e-05, 'train_speed(iter/s)': 0.535193, 'epoch': 2.1181672025723475, 'consumed_train_tokens': 4586340}

531

{'loss': 0.39884099960327146, 'learning_rate': 9.241184767277855e-05, 'train_speed(iter/s)': 0.535203, 'epoch': 2.122186495176849, 'consumed_train_tokens': 4596000}

532

{'loss': 0.3272223472595215, 'learning_rate': 9.198871650211564e-05, 'train_speed(iter/s)': 0.53526, 'epoch': 2.1262057877813505, 'consumed_train_tokens': 4605251}

533

{'loss': 0.4226553916931152, 'learning_rate': 9.156558533145274e-05, 'train_speed(iter/s)': 0.535273, 'epoch': 2.130225080385852, 'consumed_train_tokens': 4614457}

534

{'loss': 0.3847321033477783, 'learning_rate': 9.114245416078983e-05, 'train_speed(iter/s)': 0.535305, 'epoch': 2.1342443729903535, 'consumed_train_tokens': 4624365}

535

{'loss': 0.36827912330627444, 'learning_rate': 9.071932299012694e-05, 'train_speed(iter/s)': 0.535281, 'epoch': 2.1382636655948555, 'consumed_train_tokens': 4632640}

536

{'loss': 0.38245985507965086, 'learning_rate': 9.029619181946403e-05, 'train_speed(iter/s)': 0.535327, 'epoch': 2.142282958199357, 'consumed_train_tokens': 4642192}

537

{'loss': 0.45864300727844237, 'learning_rate': 8.987306064880112e-05, 'train_speed(iter/s)': 0.535362, 'epoch': 2.1463022508038585, 'consumed_train_tokens': 4650539}

538

{'loss': 0.3399721384048462, 'learning_rate': 8.944992947813822e-05, 'train_speed(iter/s)': 0.535387, 'epoch': 2.15032154340836, 'consumed_train_tokens': 4659078}

539

{'loss': 0.371239972114563, 'learning_rate': 8.902679830747531e-05, 'train_speed(iter/s)': 0.535395, 'epoch': 2.154340836012862, 'consumed_train_tokens': 4668316}

540

{'loss': 0.38885719776153566, 'learning_rate': 8.86036671368124e-05, 'train_speed(iter/s)': 0.535428, 'epoch': 2.1583601286173635, 'consumed_train_tokens': 4676363}

541

{'loss': 0.38009042739868165, 'learning_rate': 8.81805359661495e-05, 'train_speed(iter/s)': 0.535418, 'epoch': 2.162379421221865, 'consumed_train_tokens': 4684623}

542

{'loss': 0.35316078662872313, 'learning_rate': 8.77574047954866e-05, 'train_speed(iter/s)': 0.535442, 'epoch': 2.1663987138263665, 'consumed_train_tokens': 4693413}

543

{'loss': 0.2994711875915527, 'learning_rate': 8.733427362482369e-05, 'train_speed(iter/s)': 0.535406, 'epoch': 2.170418006430868, 'consumed_train_tokens': 4702027}

544

{'loss': 0.41526260375976565, 'learning_rate': 8.691114245416078e-05, 'train_speed(iter/s)': 0.535423, 'epoch': 2.17443729903537, 'consumed_train_tokens': 4711250}

545

{'loss': 0.4002061367034912, 'learning_rate': 8.648801128349789e-05, 'train_speed(iter/s)': 0.535407, 'epoch': 2.1784565916398715, 'consumed_train_tokens': 4720071}

546

{'loss': 0.2932243824005127, 'learning_rate': 8.606488011283498e-05, 'train_speed(iter/s)': 0.535379, 'epoch': 2.182475884244373, 'consumed_train_tokens': 4728629}

547

{'loss': 0.38091468811035156, 'learning_rate': 8.564174894217208e-05, 'train_speed(iter/s)': 0.5354, 'epoch': 2.1864951768488745, 'consumed_train_tokens': 4738045}

548

{'loss': 0.389581036567688, 'learning_rate': 8.521861777150917e-05, 'train_speed(iter/s)': 0.535387, 'epoch': 2.190514469453376, 'consumed_train_tokens': 4747461}

549

{'loss': 0.35653223991394045, 'learning_rate': 8.479548660084626e-05, 'train_speed(iter/s)': 0.535428, 'epoch': 2.194533762057878, 'consumed_train_tokens': 4756892}

550

{'loss': 0.3967769622802734, 'learning_rate': 8.437235543018334e-05, 'train_speed(iter/s)': 0.535409, 'epoch': 2.1985530546623795, 'consumed_train_tokens': 4765504}

551

{'loss': 0.39456839561462403, 'learning_rate': 8.394922425952044e-05, 'train_speed(iter/s)': 0.535462, 'epoch': 2.202572347266881, 'consumed_train_tokens': 4774333}

552

{'loss': 0.3913128852844238, 'learning_rate': 8.352609308885753e-05, 'train_speed(iter/s)': 0.535405, 'epoch': 2.2065916398713825, 'consumed_train_tokens': 4782659}

553

{'loss': 0.3647205352783203, 'learning_rate': 8.310296191819462e-05, 'train_speed(iter/s)': 0.535464, 'epoch': 2.210610932475884, 'consumed_train_tokens': 4791351}

554

{'loss': 0.33092608451843264, 'learning_rate': 8.267983074753172e-05, 'train_speed(iter/s)': 0.535467, 'epoch': 2.214630225080386, 'consumed_train_tokens': 4799647}

555

{'loss': 0.3769690990447998, 'learning_rate': 8.225669957686881e-05, 'train_speed(iter/s)': 0.53552, 'epoch': 2.2186495176848875, 'consumed_train_tokens': 4807598}

556

{'loss': 0.35993616580963134, 'learning_rate': 8.18335684062059e-05, 'train_speed(iter/s)': 0.535479, 'epoch': 2.222668810289389, 'consumed_train_tokens': 4815871}

557

{'loss': 0.36876673698425294, 'learning_rate': 8.141043723554301e-05, 'train_speed(iter/s)': 0.535502, 'epoch': 2.2266881028938905, 'consumed_train_tokens': 4824149}

558

{'loss': 0.32119455337524416, 'learning_rate': 8.098730606488011e-05, 'train_speed(iter/s)': 0.535489, 'epoch': 2.2307073954983925, 'consumed_train_tokens': 4832604}

559

{'loss': 0.3759403467178345, 'learning_rate': 8.05641748942172e-05, 'train_speed(iter/s)': 0.535525, 'epoch': 2.234726688102894, 'consumed_train_tokens': 4840924}

560

{'loss': 0.37905476093292234, 'learning_rate': 8.01410437235543e-05, 'train_speed(iter/s)': 0.535514, 'epoch': 2.2387459807073955, 'consumed_train_tokens': 4849447}

561

{'loss': 0.35011246204376223, 'learning_rate': 7.971791255289139e-05, 'train_speed(iter/s)': 0.535518, 'epoch': 2.242765273311897, 'consumed_train_tokens': 4857625}

562

{'loss': 0.3482373714447021, 'learning_rate': 7.929478138222848e-05, 'train_speed(iter/s)': 0.535508, 'epoch': 2.2467845659163985, 'consumed_train_tokens': 4866687}

563

{'loss': 0.3753232002258301, 'learning_rate': 7.887165021156558e-05, 'train_speed(iter/s)': 0.535529, 'epoch': 2.2508038585209005, 'consumed_train_tokens': 4874988}

564

{'loss': 0.3738467454910278, 'learning_rate': 7.844851904090267e-05, 'train_speed(iter/s)': 0.535534, 'epoch': 2.254823151125402, 'consumed_train_tokens': 4884967}

565

{'loss': 0.3719653844833374, 'learning_rate': 7.802538787023976e-05, 'train_speed(iter/s)': 0.535537, 'epoch': 2.2588424437299035, 'consumed_train_tokens': 4893149}

566

{'loss': 0.41342916488647463, 'learning_rate': 7.760225669957687e-05, 'train_speed(iter/s)': 0.535543, 'epoch': 2.262861736334405, 'consumed_train_tokens': 4901543}

567

{'loss': 0.384342622756958, 'learning_rate': 7.717912552891397e-05, 'train_speed(iter/s)': 0.53558, 'epoch': 2.266881028938907, 'consumed_train_tokens': 4910275}

568

{'loss': 0.378667426109314, 'learning_rate': 7.675599435825106e-05, 'train_speed(iter/s)': 0.535554, 'epoch': 2.2709003215434085, 'consumed_train_tokens': 4918641}

569

{'loss': 0.3832320928573608, 'learning_rate': 7.633286318758815e-05, 'train_speed(iter/s)': 0.53555, 'epoch': 2.27491961414791, 'consumed_train_tokens': 4927403}

570

{'loss': 0.40775189399719236, 'learning_rate': 7.590973201692525e-05, 'train_speed(iter/s)': 0.535526, 'epoch': 2.2789389067524115, 'consumed_train_tokens': 4935764}

571

{'loss': 0.3699026584625244, 'learning_rate': 7.548660084626234e-05, 'train_speed(iter/s)': 0.535527, 'epoch': 2.282958199356913, 'consumed_train_tokens': 4944537}

572

{'loss': 0.3144049167633057, 'learning_rate': 7.506346967559943e-05, 'train_speed(iter/s)': 0.535475, 'epoch': 2.286977491961415, 'consumed_train_tokens': 4953286}

573

{'loss': 0.3679019927978516, 'learning_rate': 7.464033850493653e-05, 'train_speed(iter/s)': 0.53547, 'epoch': 2.2909967845659165, 'consumed_train_tokens': 4961966}

574

{'loss': 0.4802206039428711, 'learning_rate': 7.421720733427362e-05, 'train_speed(iter/s)': 0.535436, 'epoch': 2.295016077170418, 'consumed_train_tokens': 4970808}

575

{'loss': 0.4515659809112549, 'learning_rate': 7.379407616361072e-05, 'train_speed(iter/s)': 0.535423, 'epoch': 2.2990353697749195, 'consumed_train_tokens': 4978996}

576

{'loss': 0.4176229476928711, 'learning_rate': 7.337094499294781e-05, 'train_speed(iter/s)': 0.535406, 'epoch': 2.303054662379421, 'consumed_train_tokens': 4987714}

577

{'loss': 0.4446138858795166, 'learning_rate': 7.29478138222849e-05, 'train_speed(iter/s)': 0.535385, 'epoch': 2.307073954983923, 'consumed_train_tokens': 4996128}

578

{'loss': 0.37184805870056153, 'learning_rate': 7.2524682651622e-05, 'train_speed(iter/s)': 0.535371, 'epoch': 2.3110932475884245, 'consumed_train_tokens': 5004615}

579

{'loss': 0.4182904243469238, 'learning_rate': 7.210155148095909e-05, 'train_speed(iter/s)': 0.53535, 'epoch': 2.315112540192926, 'consumed_train_tokens': 5013427}

580

{'loss': 0.3526875972747803, 'learning_rate': 7.167842031029618e-05, 'train_speed(iter/s)': 0.535335, 'epoch': 2.3191318327974275, 'consumed_train_tokens': 5021421}

581

{'loss': 0.3898531436920166, 'learning_rate': 7.125528913963328e-05, 'train_speed(iter/s)': 0.535308, 'epoch': 2.323151125401929, 'consumed_train_tokens': 5030138}

582

{'loss': 0.3529295206069946, 'learning_rate': 7.083215796897037e-05, 'train_speed(iter/s)': 0.535334, 'epoch': 2.327170418006431, 'consumed_train_tokens': 5038724}

583

{'loss': 0.3684830665588379, 'learning_rate': 7.040902679830747e-05, 'train_speed(iter/s)': 0.535294, 'epoch': 2.3311897106109325, 'consumed_train_tokens': 5048899}

584

{'loss': 0.45230989456176757, 'learning_rate': 6.998589562764456e-05, 'train_speed(iter/s)': 0.535311, 'epoch': 2.335209003215434, 'consumed_train_tokens': 5057359}

585

{'loss': 0.44119720458984374, 'learning_rate': 6.956276445698165e-05, 'train_speed(iter/s)': 0.535285, 'epoch': 2.3392282958199355, 'consumed_train_tokens': 5066406}

586

{'loss': 0.4363577842712402, 'learning_rate': 6.913963328631875e-05, 'train_speed(iter/s)': 0.535342, 'epoch': 2.3432475884244375, 'consumed_train_tokens': 5075343}

587

{'loss': 0.43594980239868164, 'learning_rate': 6.871650211565584e-05, 'train_speed(iter/s)': 0.535333, 'epoch': 2.347266881028939, 'consumed_train_tokens': 5083983}

588

{'loss': 0.36310520172119143, 'learning_rate': 6.829337094499295e-05, 'train_speed(iter/s)': 0.535354, 'epoch': 2.3512861736334405, 'consumed_train_tokens': 5092999}

589

{'loss': 0.3761561632156372, 'learning_rate': 6.787023977433004e-05, 'train_speed(iter/s)': 0.535371, 'epoch': 2.355305466237942, 'consumed_train_tokens': 5101309}

590

{'loss': 0.3831522464752197, 'learning_rate': 6.744710860366714e-05, 'train_speed(iter/s)': 0.535386, 'epoch': 2.359324758842444, 'consumed_train_tokens': 5110258}

591

{'loss': 0.3633324146270752, 'learning_rate': 6.702397743300423e-05, 'train_speed(iter/s)': 0.535371, 'epoch': 2.3633440514469455, 'consumed_train_tokens': 5118972}

592

{'loss': 0.3064321517944336, 'learning_rate': 6.660084626234132e-05, 'train_speed(iter/s)': 0.535376, 'epoch': 2.367363344051447, 'consumed_train_tokens': 5128017}

593

{'loss': 0.31992995738983154, 'learning_rate': 6.617771509167842e-05, 'train_speed(iter/s)': 0.535358, 'epoch': 2.3713826366559485, 'consumed_train_tokens': 5136957}

594

{'loss': 0.38301725387573243, 'learning_rate': 6.575458392101551e-05, 'train_speed(iter/s)': 0.53538, 'epoch': 2.37540192926045, 'consumed_train_tokens': 5145899}

595

{'loss': 0.3890284538269043, 'learning_rate': 6.53314527503526e-05, 'train_speed(iter/s)': 0.53538, 'epoch': 2.379421221864952, 'consumed_train_tokens': 5154538}

596

{'loss': 0.4174403190612793, 'learning_rate': 6.49083215796897e-05, 'train_speed(iter/s)': 0.535406, 'epoch': 2.3834405144694535, 'consumed_train_tokens': 5163506}

597

{'loss': 0.423185396194458, 'learning_rate': 6.448519040902679e-05, 'train_speed(iter/s)': 0.535384, 'epoch': 2.387459807073955, 'consumed_train_tokens': 5172110}

598

{'loss': 0.2618732929229736, 'learning_rate': 6.406205923836389e-05, 'train_speed(iter/s)': 0.5354, 'epoch': 2.3914790996784565, 'consumed_train_tokens': 5180892}

599

{'loss': 0.3531649112701416, 'learning_rate': 6.363892806770098e-05, 'train_speed(iter/s)': 0.535376, 'epoch': 2.395498392282958, 'consumed_train_tokens': 5189531}

600

{'loss': 0.37523794174194336, 'learning_rate': 6.321579689703807e-05, 'train_speed(iter/s)': 0.535377, 'epoch': 2.39951768488746, 'consumed_train_tokens': 5198445}

601

{'loss': 0.36220314502716067, 'learning_rate': 6.279266572637517e-05, 'train_speed(iter/s)': 0.535362, 'epoch': 2.4035369774919615, 'consumed_train_tokens': 5206588}

602

{'loss': 0.36202068328857423, 'learning_rate': 6.236953455571226e-05, 'train_speed(iter/s)': 0.535396, 'epoch': 2.407556270096463, 'consumed_train_tokens': 5214818}

603

{'loss': 0.3893800973892212, 'learning_rate': 6.194640338504936e-05, 'train_speed(iter/s)': 0.535383, 'epoch': 2.4115755627009645, 'consumed_train_tokens': 5222950}

604

{'loss': 0.3430525302886963, 'learning_rate': 6.152327221438646e-05, 'train_speed(iter/s)': 0.535377, 'epoch': 2.415594855305466, 'consumed_train_tokens': 5231846}

605

{'loss': 0.4007270812988281, 'learning_rate': 6.110014104372356e-05, 'train_speed(iter/s)': 0.535407, 'epoch': 2.419614147909968, 'consumed_train_tokens': 5239431}

606

{'loss': 0.38307971954345704, 'learning_rate': 6.067700987306065e-05, 'train_speed(iter/s)': 0.535442, 'epoch': 2.4236334405144695, 'consumed_train_tokens': 5247994}

607

{'loss': 0.36373214721679686, 'learning_rate': 6.025387870239774e-05, 'train_speed(iter/s)': 0.535462, 'epoch': 2.427652733118971, 'consumed_train_tokens': 5256917}

608

{'loss': 0.4563951015472412, 'learning_rate': 5.983074753173483e-05, 'train_speed(iter/s)': 0.535444, 'epoch': 2.4316720257234725, 'consumed_train_tokens': 5266015}

609

{'loss': 0.3759207248687744, 'learning_rate': 5.9407616361071925e-05, 'train_speed(iter/s)': 0.535436, 'epoch': 2.435691318327974, 'consumed_train_tokens': 5274070}

610

{'loss': 0.3058905124664307, 'learning_rate': 5.898448519040902e-05, 'train_speed(iter/s)': 0.535415, 'epoch': 2.439710610932476, 'consumed_train_tokens': 5282220}

611

{'loss': 0.27860350608825685, 'learning_rate': 5.856135401974611e-05, 'train_speed(iter/s)': 0.535441, 'epoch': 2.4437299035369775, 'consumed_train_tokens': 5290157}

612

{'loss': 0.386484169960022, 'learning_rate': 5.813822284908321e-05, 'train_speed(iter/s)': 0.535424, 'epoch': 2.447749196141479, 'consumed_train_tokens': 5298858}

613

{'loss': 0.40139050483703614, 'learning_rate': 5.771509167842031e-05, 'train_speed(iter/s)': 0.535441, 'epoch': 2.4517684887459805, 'consumed_train_tokens': 5307663}

614

{'loss': 0.4421054840087891, 'learning_rate': 5.72919605077574e-05, 'train_speed(iter/s)': 0.535409, 'epoch': 2.4557877813504825, 'consumed_train_tokens': 5316309}

615

{'loss': 0.35824995040893554, 'learning_rate': 5.6868829337094494e-05, 'train_speed(iter/s)': 0.53543, 'epoch': 2.459807073954984, 'consumed_train_tokens': 5325024}

616

{'loss': 0.38307528495788573, 'learning_rate': 5.644569816643159e-05, 'train_speed(iter/s)': 0.535438, 'epoch': 2.4638263665594855, 'consumed_train_tokens': 5333526}

617

{'loss': 0.4240251541137695, 'learning_rate': 5.602256699576869e-05, 'train_speed(iter/s)': 0.535453, 'epoch': 2.467845659163987, 'consumed_train_tokens': 5342360}

618

{'loss': 0.33363654613494875, 'learning_rate': 5.559943582510578e-05, 'train_speed(iter/s)': 0.535435, 'epoch': 2.471864951768489, 'consumed_train_tokens': 5351077}

619

{'loss': 0.3445601463317871, 'learning_rate': 5.517630465444287e-05, 'train_speed(iter/s)': 0.535431, 'epoch': 2.4758842443729905, 'consumed_train_tokens': 5360572}

620

{'loss': 0.4097161293029785, 'learning_rate': 5.475317348377996e-05, 'train_speed(iter/s)': 0.535435, 'epoch': 2.479903536977492, 'consumed_train_tokens': 5368708}

621

{'loss': 0.33888514041900636, 'learning_rate': 5.433004231311706e-05, 'train_speed(iter/s)': 0.535456, 'epoch': 2.4839228295819935, 'consumed_train_tokens': 5377676}

622

{'loss': 0.4064308166503906, 'learning_rate': 5.390691114245415e-05, 'train_speed(iter/s)': 0.535474, 'epoch': 2.487942122186495, 'consumed_train_tokens': 5386726}

623

{'loss': 0.36492218971252444, 'learning_rate': 5.348377997179125e-05, 'train_speed(iter/s)': 0.535488, 'epoch': 2.491961414790997, 'consumed_train_tokens': 5395099}

624

{'loss': 0.30016183853149414, 'learning_rate': 5.3060648801128345e-05, 'train_speed(iter/s)': 0.535513, 'epoch': 2.4959807073954985, 'consumed_train_tokens': 5403408}

625

{'loss': 0.3799457311630249, 'learning_rate': 5.263751763046544e-05, 'train_speed(iter/s)': 0.535524, 'epoch': 2.5, 'consumed_train_tokens': 5411738}

626

{'loss': 0.3790318489074707, 'learning_rate': 5.221438645980253e-05, 'train_speed(iter/s)': 0.535493, 'epoch': 2.5040192926045015, 'consumed_train_tokens': 5420129}

627

{'loss': 0.37256290912628176, 'learning_rate': 5.1791255289139626e-05, 'train_speed(iter/s)': 0.535493, 'epoch': 2.508038585209003, 'consumed_train_tokens': 5429787}

628

{'loss': 0.3767810106277466, 'learning_rate': 5.136812411847673e-05, 'train_speed(iter/s)': 0.535465, 'epoch': 2.512057877813505, 'consumed_train_tokens': 5438680}

629

{'loss': 0.3556367874145508, 'learning_rate': 5.094499294781382e-05, 'train_speed(iter/s)': 0.535465, 'epoch': 2.5160771704180065, 'consumed_train_tokens': 5447737}

630

{'loss': 0.35794506072998045, 'learning_rate': 5.0521861777150915e-05, 'train_speed(iter/s)': 0.535438, 'epoch': 2.520096463022508, 'consumed_train_tokens': 5457136}

631

{'loss': 0.40340938568115237, 'learning_rate': 5.009873060648801e-05, 'train_speed(iter/s)': 0.535449, 'epoch': 2.5241157556270095, 'consumed_train_tokens': 5465769}

632

{'loss': 0.3194626808166504, 'learning_rate': 4.9675599435825095e-05, 'train_speed(iter/s)': 0.53543, 'epoch': 2.528135048231511, 'consumed_train_tokens': 5474077}

633

{'loss': 0.39080562591552737, 'learning_rate': 4.9252468265162196e-05, 'train_speed(iter/s)': 0.535436, 'epoch': 2.532154340836013, 'consumed_train_tokens': 5482585}

634

{'loss': 0.3540614604949951, 'learning_rate': 4.882933709449929e-05, 'train_speed(iter/s)': 0.535409, 'epoch': 2.5361736334405145, 'consumed_train_tokens': 5490985}

635

{'loss': 0.39537692070007324, 'learning_rate': 4.8406205923836384e-05, 'train_speed(iter/s)': 0.535446, 'epoch': 2.540192926045016, 'consumed_train_tokens': 5499877}

636

{'loss': 0.4151315689086914, 'learning_rate': 4.798307475317348e-05, 'train_speed(iter/s)': 0.535438, 'epoch': 2.5442122186495175, 'consumed_train_tokens': 5509322}

637

{'loss': 0.3548682451248169, 'learning_rate': 4.755994358251057e-05, 'train_speed(iter/s)': 0.535482, 'epoch': 2.548231511254019, 'consumed_train_tokens': 5518295}

638

{'loss': 0.4232761383056641, 'learning_rate': 4.713681241184767e-05, 'train_speed(iter/s)': 0.535486, 'epoch': 2.552250803858521, 'consumed_train_tokens': 5526835}

639

{'loss': 0.43976173400878904, 'learning_rate': 4.6713681241184766e-05, 'train_speed(iter/s)': 0.535473, 'epoch': 2.5562700964630225, 'consumed_train_tokens': 5535110}

640

{'loss': 0.39546942710876465, 'learning_rate': 4.629055007052186e-05, 'train_speed(iter/s)': 0.535469, 'epoch': 2.560289389067524, 'consumed_train_tokens': 5544125}

641

{'loss': 0.3911599636077881, 'learning_rate': 4.586741889985895e-05, 'train_speed(iter/s)': 0.535447, 'epoch': 2.564308681672026, 'consumed_train_tokens': 5552973}

642

{'loss': 0.3463834524154663, 'learning_rate': 4.544428772919605e-05, 'train_speed(iter/s)': 0.535474, 'epoch': 2.5683279742765275, 'consumed_train_tokens': 5561770}

643

{'loss': 0.403352689743042, 'learning_rate': 4.502115655853315e-05, 'train_speed(iter/s)': 0.535453, 'epoch': 2.572347266881029, 'consumed_train_tokens': 5570862}

644

{'loss': 0.33768839836120607, 'learning_rate': 4.459802538787024e-05, 'train_speed(iter/s)': 0.535472, 'epoch': 2.5763665594855305, 'consumed_train_tokens': 5579584}

645

{'loss': 0.3421605348587036, 'learning_rate': 4.417489421720733e-05, 'train_speed(iter/s)': 0.535483, 'epoch': 2.580385852090032, 'consumed_train_tokens': 5588330}

646

{'loss': 0.42171521186828614, 'learning_rate': 4.375176304654442e-05, 'train_speed(iter/s)': 0.535479, 'epoch': 2.584405144694534, 'consumed_train_tokens': 5597077}

647

{'loss': 0.3757301092147827, 'learning_rate': 4.3328631875881516e-05, 'train_speed(iter/s)': 0.53547, 'epoch': 2.5884244372990355, 'consumed_train_tokens': 5606446}

648

{'loss': 0.3499581813812256, 'learning_rate': 4.290550070521861e-05, 'train_speed(iter/s)': 0.535471, 'epoch': 2.592443729903537, 'consumed_train_tokens': 5615021}

649

{'loss': 0.39512226581573484, 'learning_rate': 4.248236953455571e-05, 'train_speed(iter/s)': 0.53544, 'epoch': 2.5964630225080385, 'consumed_train_tokens': 5623854}

650

{'loss': 0.3753098726272583, 'learning_rate': 4.2059238363892804e-05, 'train_speed(iter/s)': 0.53543, 'epoch': 2.60048231511254, 'consumed_train_tokens': 5632509}

651

{'loss': 0.4669802665710449, 'learning_rate': 4.16361071932299e-05, 'train_speed(iter/s)': 0.535403, 'epoch': 2.604501607717042, 'consumed_train_tokens': 5641658}

652

{'loss': 0.3838948249816895, 'learning_rate': 4.121297602256699e-05, 'train_speed(iter/s)': 0.535384, 'epoch': 2.6085209003215435, 'consumed_train_tokens': 5650321}

653

{'loss': 0.33837261199951174, 'learning_rate': 4.0789844851904085e-05, 'train_speed(iter/s)': 0.535373, 'epoch': 2.612540192926045, 'consumed_train_tokens': 5659020}

654

{'loss': 0.3602497100830078, 'learning_rate': 4.0366713681241186e-05, 'train_speed(iter/s)': 0.535366, 'epoch': 2.6165594855305465, 'consumed_train_tokens': 5667135}

655

{'loss': 0.35701441764831543, 'learning_rate': 3.994358251057828e-05, 'train_speed(iter/s)': 0.535346, 'epoch': 2.620578778135048, 'consumed_train_tokens': 5676051}

656

{'loss': 0.3885300636291504, 'learning_rate': 3.9520451339915374e-05, 'train_speed(iter/s)': 0.535323, 'epoch': 2.62459807073955, 'consumed_train_tokens': 5685032}

657

{'loss': 0.43814849853515625, 'learning_rate': 3.909732016925246e-05, 'train_speed(iter/s)': 0.535297, 'epoch': 2.6286173633440515, 'consumed_train_tokens': 5693636}

658

{'loss': 0.3256420612335205, 'learning_rate': 3.8674188998589554e-05, 'train_speed(iter/s)': 0.535311, 'epoch': 2.632636655948553, 'consumed_train_tokens': 5702137}

659

{'loss': 0.4024925231933594, 'learning_rate': 3.825105782792665e-05, 'train_speed(iter/s)': 0.535306, 'epoch': 2.6366559485530545, 'consumed_train_tokens': 5709960}

660

{'loss': 0.34529805183410645, 'learning_rate': 3.782792665726375e-05, 'train_speed(iter/s)': 0.535318, 'epoch': 2.640675241157556, 'consumed_train_tokens': 5719314}

661

{'loss': 0.3774927377700806, 'learning_rate': 3.740479548660084e-05, 'train_speed(iter/s)': 0.535296, 'epoch': 2.644694533762058, 'consumed_train_tokens': 5727521}

662

{'loss': 0.38132879734039304, 'learning_rate': 3.6981664315937936e-05, 'train_speed(iter/s)': 0.535304, 'epoch': 2.6487138263665595, 'consumed_train_tokens': 5736044}

663

{'loss': 0.37965855598449705, 'learning_rate': 3.655853314527503e-05, 'train_speed(iter/s)': 0.535294, 'epoch': 2.652733118971061, 'consumed_train_tokens': 5745715}

664

{'loss': 0.3262141227722168, 'learning_rate': 3.6135401974612124e-05, 'train_speed(iter/s)': 0.535322, 'epoch': 2.656752411575563, 'consumed_train_tokens': 5754684}

665

{'loss': 0.3840010166168213, 'learning_rate': 3.5712270803949224e-05, 'train_speed(iter/s)': 0.535309, 'epoch': 2.660771704180064, 'consumed_train_tokens': 5763693}

666

{'loss': 0.3546534776687622, 'learning_rate': 3.528913963328632e-05, 'train_speed(iter/s)': 0.535288, 'epoch': 2.664790996784566, 'consumed_train_tokens': 5772619}

667

{'loss': 0.42096357345581054, 'learning_rate': 3.4866008462623405e-05, 'train_speed(iter/s)': 0.535269, 'epoch': 2.6688102893890675, 'consumed_train_tokens': 5780818}

668

{'loss': 0.31003198623657224, 'learning_rate': 3.4442877291960506e-05, 'train_speed(iter/s)': 0.535239, 'epoch': 2.672829581993569, 'consumed_train_tokens': 5789725}

669

{'loss': 0.419179630279541, 'learning_rate': 3.40197461212976e-05, 'train_speed(iter/s)': 0.535229, 'epoch': 2.676848874598071, 'consumed_train_tokens': 5798549}

670

{'loss': 0.3675557851791382, 'learning_rate': 3.359661495063469e-05, 'train_speed(iter/s)': 0.535205, 'epoch': 2.6808681672025725, 'consumed_train_tokens': 5807725}

671

{'loss': 0.31893391609191896, 'learning_rate': 3.317348377997179e-05, 'train_speed(iter/s)': 0.535216, 'epoch': 2.684887459807074, 'consumed_train_tokens': 5817103}

672

{'loss': 0.3616243124008179, 'learning_rate': 3.275035260930889e-05, 'train_speed(iter/s)': 0.535228, 'epoch': 2.6889067524115755, 'consumed_train_tokens': 5826514}

673

{'loss': 0.384961199760437, 'learning_rate': 3.2327221438645975e-05, 'train_speed(iter/s)': 0.535257, 'epoch': 2.692926045016077, 'consumed_train_tokens': 5834608}

674

{'loss': 0.4349827289581299, 'learning_rate': 3.190409026798307e-05, 'train_speed(iter/s)': 0.535272, 'epoch': 2.696945337620579, 'consumed_train_tokens': 5843749}

675

{'loss': 0.3355870246887207, 'learning_rate': 3.148095909732017e-05, 'train_speed(iter/s)': 0.53528, 'epoch': 2.7009646302250805, 'consumed_train_tokens': 5852515}

676

{'loss': 0.3251935005187988, 'learning_rate': 3.105782792665726e-05, 'train_speed(iter/s)': 0.535225, 'epoch': 2.704983922829582, 'consumed_train_tokens': 5861042}

677

{'loss': 0.40128116607666015, 'learning_rate': 3.063469675599436e-05, 'train_speed(iter/s)': 0.535203, 'epoch': 2.7090032154340835, 'consumed_train_tokens': 5869926}

678

{'loss': 0.3213503837585449, 'learning_rate': 3.021156558533145e-05, 'train_speed(iter/s)': 0.535196, 'epoch': 2.713022508038585, 'consumed_train_tokens': 5879288}

679

{'loss': 0.36540637016296384, 'learning_rate': 2.9788434414668548e-05, 'train_speed(iter/s)': 0.535192, 'epoch': 2.717041800643087, 'consumed_train_tokens': 5888114}

680

{'loss': 0.2923316717147827, 'learning_rate': 2.9365303244005638e-05, 'train_speed(iter/s)': 0.535126, 'epoch': 2.7210610932475885, 'consumed_train_tokens': 5896799}

681

{'loss': 0.40366668701171876, 'learning_rate': 2.851904090267983e-05, 'train_speed(iter/s)': 0.535071, 'epoch': 2.7290996784565915, 'consumed_train_tokens': 5914186}

682

{'loss': 0.34505395889282225, 'learning_rate': 2.8095909732016923e-05, 'train_speed(iter/s)': 0.53506, 'epoch': 2.733118971061093, 'consumed_train_tokens': 5922931}

683

{'loss': 0.3878589868545532, 'learning_rate': 2.767277856135402e-05, 'train_speed(iter/s)': 0.535049, 'epoch': 2.737138263665595, 'consumed_train_tokens': 5931460}

684

{'loss': 0.36542658805847167, 'learning_rate': 2.7249647390691114e-05, 'train_speed(iter/s)': 0.535054, 'epoch': 2.7411575562700965, 'consumed_train_tokens': 5939575}

685

{'loss': 0.35235013961791994, 'learning_rate': 2.6826516220028204e-05, 'train_speed(iter/s)': 0.535035, 'epoch': 2.745176848874598, 'consumed_train_tokens': 5948350}

686

{'loss': 0.3488276481628418, 'learning_rate': 2.64033850493653e-05, 'train_speed(iter/s)': 0.535012, 'epoch': 2.7491961414790995, 'consumed_train_tokens': 5956445}

687

{'loss': 0.3241608142852783, 'learning_rate': 2.5980253878702395e-05, 'train_speed(iter/s)': 0.535011, 'epoch': 2.753215434083601, 'consumed_train_tokens': 5965717}

688

{'loss': 0.36849403381347656, 'learning_rate': 2.555712270803949e-05, 'train_speed(iter/s)': 0.535007, 'epoch': 2.757234726688103, 'consumed_train_tokens': 5974395}

689

{'loss': 0.3205351114273071, 'learning_rate': 2.5133991537376586e-05, 'train_speed(iter/s)': 0.535004, 'epoch': 2.7612540192926045, 'consumed_train_tokens': 5982879}

690

{'loss': 0.3759245634078979, 'learning_rate': 2.471086036671368e-05, 'train_speed(iter/s)': 0.534991, 'epoch': 2.765273311897106, 'consumed_train_tokens': 5990879}

691

{'loss': 0.38630716800689696, 'learning_rate': 2.428772919605077e-05, 'train_speed(iter/s)': 0.534991, 'epoch': 2.769292604501608, 'consumed_train_tokens': 5999412}

692

{'loss': 0.4111736297607422, 'learning_rate': 2.3864598025387868e-05, 'train_speed(iter/s)': 0.534987, 'epoch': 2.7733118971061095, 'consumed_train_tokens': 6008121}

693

{'loss': 0.369177508354187, 'learning_rate': 2.344146685472496e-05, 'train_speed(iter/s)': 0.534975, 'epoch': 2.777331189710611, 'consumed_train_tokens': 6016498}

694

{'loss': 0.4054384231567383, 'learning_rate': 2.301833568406206e-05, 'train_speed(iter/s)': 0.534963, 'epoch': 2.7813504823151125, 'consumed_train_tokens': 6025696}

695

{'loss': 0.35865471363067625, 'learning_rate': 2.2595204513399152e-05, 'train_speed(iter/s)': 0.534966, 'epoch': 2.785369774919614, 'consumed_train_tokens': 6034553}

696

{'loss': 0.3765880107879639, 'learning_rate': 2.2172073342736246e-05, 'train_speed(iter/s)': 0.534964, 'epoch': 2.789389067524116, 'consumed_train_tokens': 6042999}

697

{'loss': 0.2763675689697266, 'learning_rate': 2.1748942172073343e-05, 'train_speed(iter/s)': 0.534981, 'epoch': 2.7934083601286175, 'consumed_train_tokens': 6051572}

698

{'loss': 0.3638096809387207, 'learning_rate': 2.1325811001410434e-05, 'train_speed(iter/s)': 0.534951, 'epoch': 2.797427652733119, 'consumed_train_tokens': 6059373}

699

{'loss': 0.2911883592605591, 'learning_rate': 2.0902679830747527e-05, 'train_speed(iter/s)': 0.534957, 'epoch': 2.8014469453376205, 'consumed_train_tokens': 6068825}

700

{'loss': 0.38112525939941405, 'learning_rate': 2.0479548660084625e-05, 'train_speed(iter/s)': 0.534962, 'epoch': 2.805466237942122, 'consumed_train_tokens': 6077423}

701

{'loss': 0.35434751510620116, 'learning_rate': 2.005641748942172e-05, 'train_speed(iter/s)': 0.534985, 'epoch': 2.809485530546624, 'consumed_train_tokens': 6085626}

702

{'loss': 0.40532426834106444, 'learning_rate': 1.9633286318758816e-05, 'train_speed(iter/s)': 0.534971, 'epoch': 2.8135048231511255, 'consumed_train_tokens': 6094568}

703

{'loss': 0.40397186279296876, 'learning_rate': 1.921015514809591e-05, 'train_speed(iter/s)': 0.534979, 'epoch': 2.817524115755627, 'consumed_train_tokens': 6102833}

704

{'loss': 0.3605435848236084, 'learning_rate': 1.8787023977433e-05, 'train_speed(iter/s)': 0.534966, 'epoch': 2.8215434083601285, 'consumed_train_tokens': 6111577}

705

{'loss': 0.3643901586532593, 'learning_rate': 1.8363892806770097e-05, 'train_speed(iter/s)': 0.535, 'epoch': 2.82556270096463, 'consumed_train_tokens': 6120425}

706

{'loss': 0.39566917419433595, 'learning_rate': 1.794076163610719e-05, 'train_speed(iter/s)': 0.534979, 'epoch': 2.829581993569132, 'consumed_train_tokens': 6128830}

707

{'loss': 0.44511804580688474, 'learning_rate': 1.7517630465444288e-05, 'train_speed(iter/s)': 0.534981, 'epoch': 2.8336012861736335, 'consumed_train_tokens': 6138077}

708

{'loss': 0.3398555278778076, 'learning_rate': 1.7094499294781382e-05, 'train_speed(iter/s)': 0.534971, 'epoch': 2.837620578778135, 'consumed_train_tokens': 6146953}

709

{'loss': 0.404541540145874, 'learning_rate': 1.6671368124118476e-05, 'train_speed(iter/s)': 0.534989, 'epoch': 2.8416398713826365, 'consumed_train_tokens': 6154830}

710

{'loss': 0.40536274909973147, 'learning_rate': 1.624823695345557e-05, 'train_speed(iter/s)': 0.534989, 'epoch': 2.845659163987138, 'consumed_train_tokens': 6163589}

711

{'loss': 0.4472348213195801, 'learning_rate': 1.5825105782792666e-05, 'train_speed(iter/s)': 0.534998, 'epoch': 2.84967845659164, 'consumed_train_tokens': 6171855}

712

{'loss': 0.35651535987854005, 'learning_rate': 1.5401974612129757e-05, 'train_speed(iter/s)': 0.53502, 'epoch': 2.8536977491961415, 'consumed_train_tokens': 6180623}

713

{'loss': 0.3322479009628296, 'learning_rate': 1.4555712270803948e-05, 'train_speed(iter/s)': 0.535067, 'epoch': 2.861736334405145, 'consumed_train_tokens': 6198596}

714

{'loss': 0.35284345149993895, 'learning_rate': 1.4132581100141042e-05, 'train_speed(iter/s)': 0.53505, 'epoch': 2.865755627009646, 'consumed_train_tokens': 6207437}

715

{'loss': 0.38554906845092773, 'learning_rate': 1.3709449929478137e-05, 'train_speed(iter/s)': 0.535055, 'epoch': 2.869774919614148, 'consumed_train_tokens': 6215961}

716

{'loss': 0.3224207878112793, 'learning_rate': 1.3286318758815233e-05, 'train_speed(iter/s)': 0.535074, 'epoch': 2.8737942122186495, 'consumed_train_tokens': 6224233}

717

{'loss': 0.48133411407470705, 'learning_rate': 1.2863187588152325e-05, 'train_speed(iter/s)': 0.535074, 'epoch': 2.877813504823151, 'consumed_train_tokens': 6233387}

718

{'loss': 0.3484313726425171, 'learning_rate': 1.244005641748942e-05, 'train_speed(iter/s)': 0.535097, 'epoch': 2.881832797427653, 'consumed_train_tokens': 6241837}

719

{'loss': 0.40407662391662597, 'learning_rate': 1.2016925246826516e-05, 'train_speed(iter/s)': 0.535129, 'epoch': 2.8858520900321545, 'consumed_train_tokens': 6250678}

720

{'loss': 0.30222220420837403, 'learning_rate': 1.159379407616361e-05, 'train_speed(iter/s)': 0.535137, 'epoch': 2.889871382636656, 'consumed_train_tokens': 6259426}

721

{'loss': 0.36746878623962403, 'learning_rate': 1.1170662905500703e-05, 'train_speed(iter/s)': 0.535174, 'epoch': 2.8938906752411575, 'consumed_train_tokens': 6267889}

722

{'loss': 0.41803340911865233, 'learning_rate': 1.0747531734837799e-05, 'train_speed(iter/s)': 0.535216, 'epoch': 2.897909967845659, 'consumed_train_tokens': 6276946}

723

{'loss': 0.34108092784881594, 'learning_rate': 1.0324400564174894e-05, 'train_speed(iter/s)': 0.535252, 'epoch': 2.901929260450161, 'consumed_train_tokens': 6285182}

724

{'loss': 0.4171647548675537, 'learning_rate': 9.901269393511988e-06, 'train_speed(iter/s)': 0.535291, 'epoch': 2.9059485530546625, 'consumed_train_tokens': 6293531}

725

{'loss': 0.37908923625946045, 'learning_rate': 9.478138222849082e-06, 'train_speed(iter/s)': 0.53531, 'epoch': 2.909967845659164, 'consumed_train_tokens': 6301907}

726

{'loss': 0.33609273433685305, 'learning_rate': 9.055007052186177e-06, 'train_speed(iter/s)': 0.535333, 'epoch': 2.9139871382636655, 'consumed_train_tokens': 6311887}

727

{'loss': 0.43314423561096194, 'learning_rate': 8.631875881523271e-06, 'train_speed(iter/s)': 0.535363, 'epoch': 2.918006430868167, 'consumed_train_tokens': 6320577}

728

{'loss': 0.40961418151855467, 'learning_rate': 8.208744710860367e-06, 'train_speed(iter/s)': 0.535387, 'epoch': 2.922025723472669, 'consumed_train_tokens': 6328910}

729

{'loss': 0.3296086072921753, 'learning_rate': 7.78561354019746e-06, 'train_speed(iter/s)': 0.535412, 'epoch': 2.9260450160771705, 'consumed_train_tokens': 6337171}

730

{'loss': 0.31569409370422363, 'learning_rate': 7.362482369534556e-06, 'train_speed(iter/s)': 0.535431, 'epoch': 2.930064308681672, 'consumed_train_tokens': 6345733}

731

{'loss': 0.3399463653564453, 'learning_rate': 6.93935119887165e-06, 'train_speed(iter/s)': 0.535429, 'epoch': 2.9340836012861735, 'consumed_train_tokens': 6354759}

732

{'loss': 0.3870511770248413, 'learning_rate': 6.516220028208743e-06, 'train_speed(iter/s)': 0.535424, 'epoch': 2.938102893890675, 'consumed_train_tokens': 6363302}

733

{'loss': 0.3977831840515137, 'learning_rate': 6.093088857545839e-06, 'train_speed(iter/s)': 0.53544, 'epoch': 2.942122186495177, 'consumed_train_tokens': 6371891}

734

{'loss': 0.3795290470123291, 'learning_rate': 5.6699576868829335e-06, 'train_speed(iter/s)': 0.535454, 'epoch': 2.9461414790996785, 'consumed_train_tokens': 6380128}

735

{'loss': 0.3658801555633545, 'learning_rate': 5.246826516220027e-06, 'train_speed(iter/s)': 0.535441, 'epoch': 2.95016077170418, 'consumed_train_tokens': 6388111}

736

{'loss': 0.3640072107315063, 'learning_rate': 4.823695345557123e-06, 'train_speed(iter/s)': 0.535438, 'epoch': 2.9541800643086815, 'consumed_train_tokens': 6396873}

737

{'loss': 0.33306357860565183, 'learning_rate': 4.400564174894217e-06, 'train_speed(iter/s)': 0.535444, 'epoch': 2.958199356913183, 'consumed_train_tokens': 6405147}

738

{'loss': 0.39460196495056155, 'learning_rate': 3.977433004231311e-06, 'train_speed(iter/s)': 0.535439, 'epoch': 2.962218649517685, 'consumed_train_tokens': 6413960}

739

{'loss': 0.36114501953125, 'learning_rate': 3.554301833568406e-06, 'train_speed(iter/s)': 0.535467, 'epoch': 2.9662379421221865, 'consumed_train_tokens': 6422768}

740

{'loss': 0.3815159797668457, 'learning_rate': 3.1311706629055005e-06, 'train_speed(iter/s)': 0.535449, 'epoch': 2.970257234726688, 'consumed_train_tokens': 6432270}

741

{'loss': 0.33249726295471194, 'learning_rate': 2.7080394922425947e-06, 'train_speed(iter/s)': 0.535454, 'epoch': 2.97427652733119, 'consumed_train_tokens': 6440482}

742

{'loss': 0.42241883277893066, 'learning_rate': 2.2849083215796894e-06, 'train_speed(iter/s)': 0.535437, 'epoch': 2.9782958199356915, 'consumed_train_tokens': 6448528}

743

{'loss': 0.41171798706054685, 'learning_rate': 1.8617771509167842e-06, 'train_speed(iter/s)': 0.535427, 'epoch': 2.982315112540193, 'consumed_train_tokens': 6456874}

744

{'loss': 0.3142673492431641, 'learning_rate': 1.4386459802538787e-06, 'train_speed(iter/s)': 0.53543, 'epoch': 2.9863344051446945, 'consumed_train_tokens': 6465409}

745

{'loss': 0.34425232410430906, 'learning_rate': 1.015514809590973e-06, 'train_speed(iter/s)': 0.535425, 'epoch': 2.990353697749196, 'consumed_train_tokens': 6474402}

746

{'loss': 0.3002054214477539, 'learning_rate': 5.923836389280676e-07, 'train_speed(iter/s)': 0.535432, 'epoch': 2.994372990353698, 'consumed_train_tokens': 6483223}

747

{'loss': 0.3696011543273926, 'learning_rate': 1.6925246826516217e-07, 'train_speed(iter/s)': 0.53545, 'epoch': 2.9983922829581995, 'consumed_train_tokens': 6492171}

748

{'eval_loss': 0.851229727268219, 'eval_runtime': 18.3471, 'eval_samples_per_second': 54.505, 'eval_steps_per_second': 13.626, 'epoch': 3.0}

749

{'eval_loss': 0.851229727268219, 'eval_runtime': 18.4698, 'eval_samples_per_second': 54.142, 'eval_steps_per_second': 13.536, 'epoch': 3.0}

750

{'train_runtime': 7006.8851, 'train_samples_per_second': 8.523, 'train_steps_per_second': 0.533, 'train_loss': 0.6038002791991188, 'epoch': 3.0, 'consumed_train_tokens': 6495807}

751

Actual number of consumed tokens is 6495807!

752

Fine-tune succeeded!

753

2025-10-05 22:13:10,041 - INFO - fine-tuned output got, start to transfer it for inference

754

2025-10-05 22:14:15,520 - INFO - transfer for inference succeeded, start to deliver it for inference

755

2025-10-05 22:16:27,512 - INFO - start to save checkpoint

756

2025-10-05 22:19:49,050 - INFO - finetune-job succeeded

757

2025-10-05 22:19:49,559 - INFO - training usage 6495807

758

2025-10-05 22:19:49,574 - INFO - ##FT_COMPLETE##
