# Supplementary figures and images for: Textual overlap rather than domain alignment: A comparative study of fine-tuning strategies for specialised machine translation with large language models
Source: PLoS One. 2026 Jul 20;21(7):e0352256. doi: 10.1371/journal.pone.0352256 (PMC13384323; doi:10.1371/journal.pone.0352256)

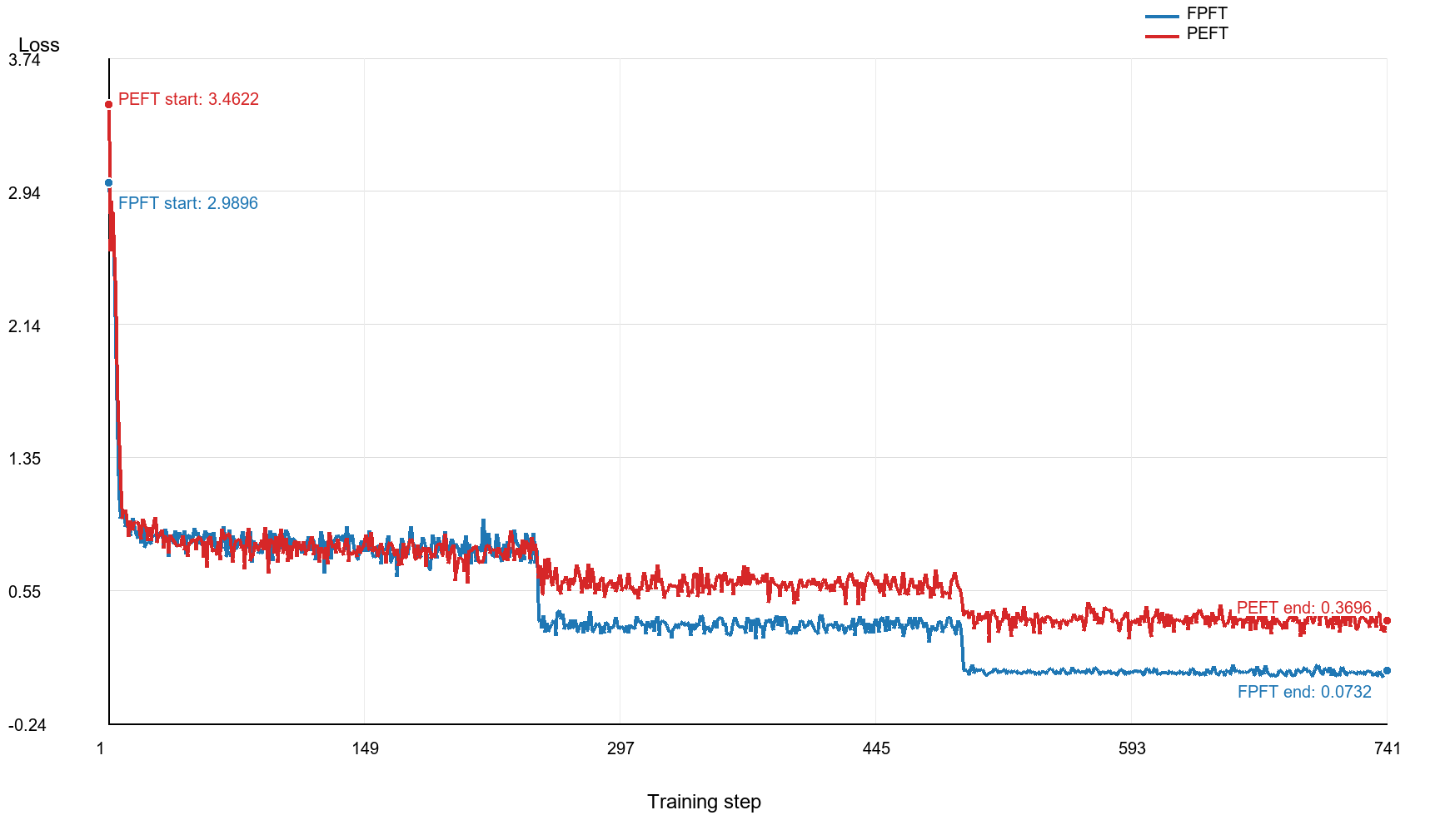

Supplement: S3 File — (ZIP) [file pone.0352256.s003.zip › Fig1.tif]

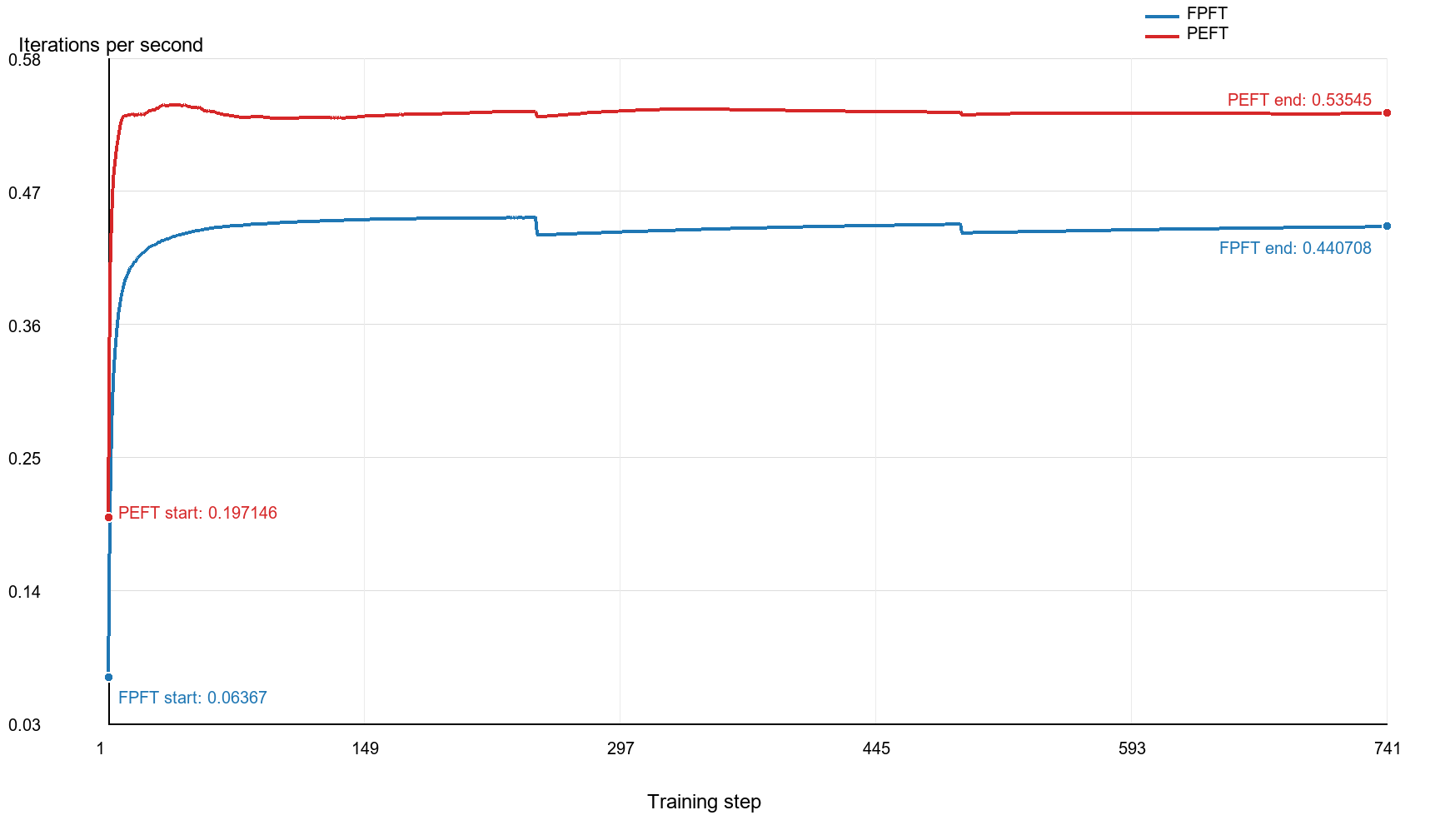

Supplement: S3 File — (ZIP) [file pone.0352256.s003.zip › Fig2.tif]

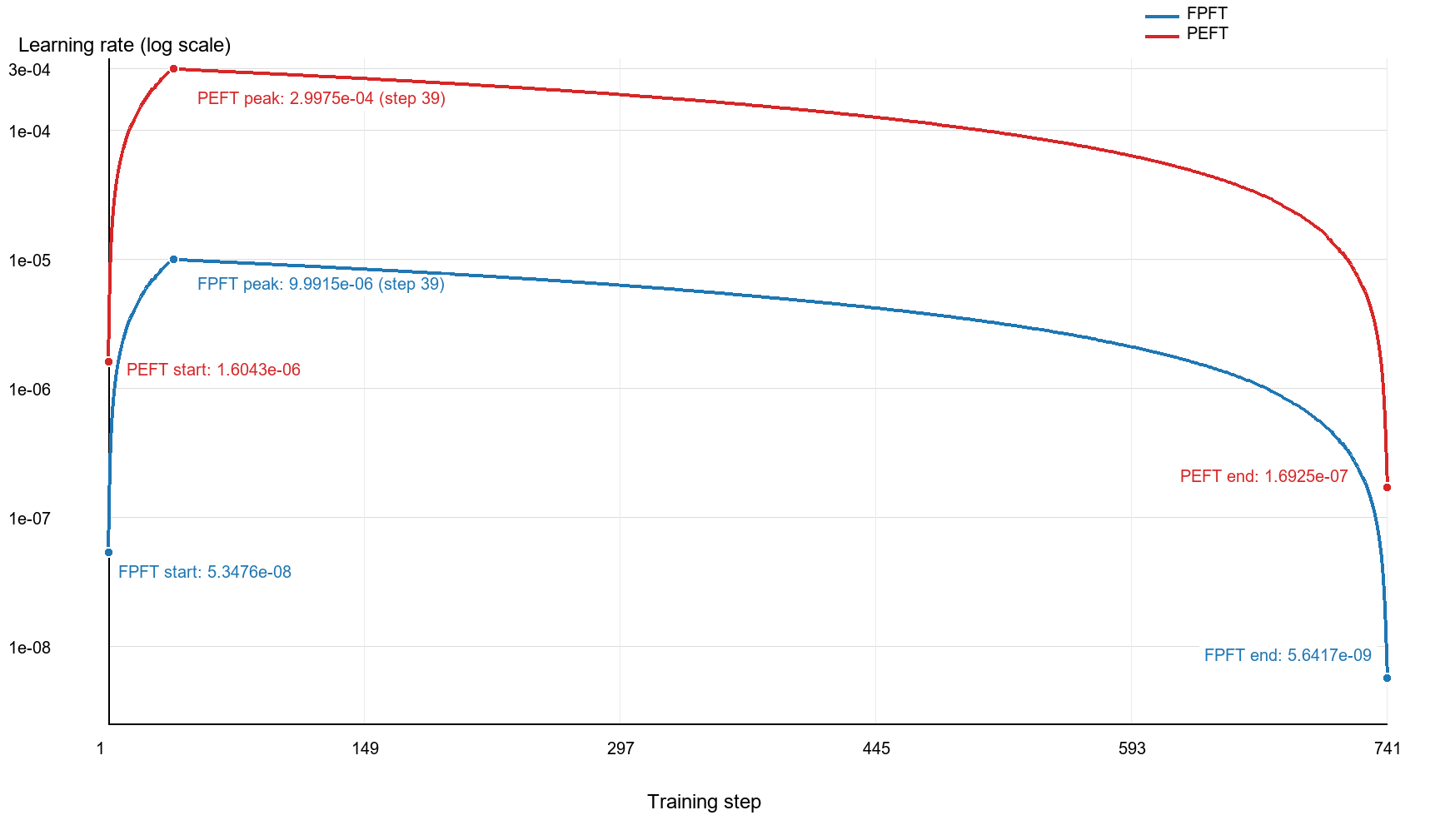

Supplement: S3 File — (ZIP) [file pone.0352256.s003.zip › Fig3.tif]

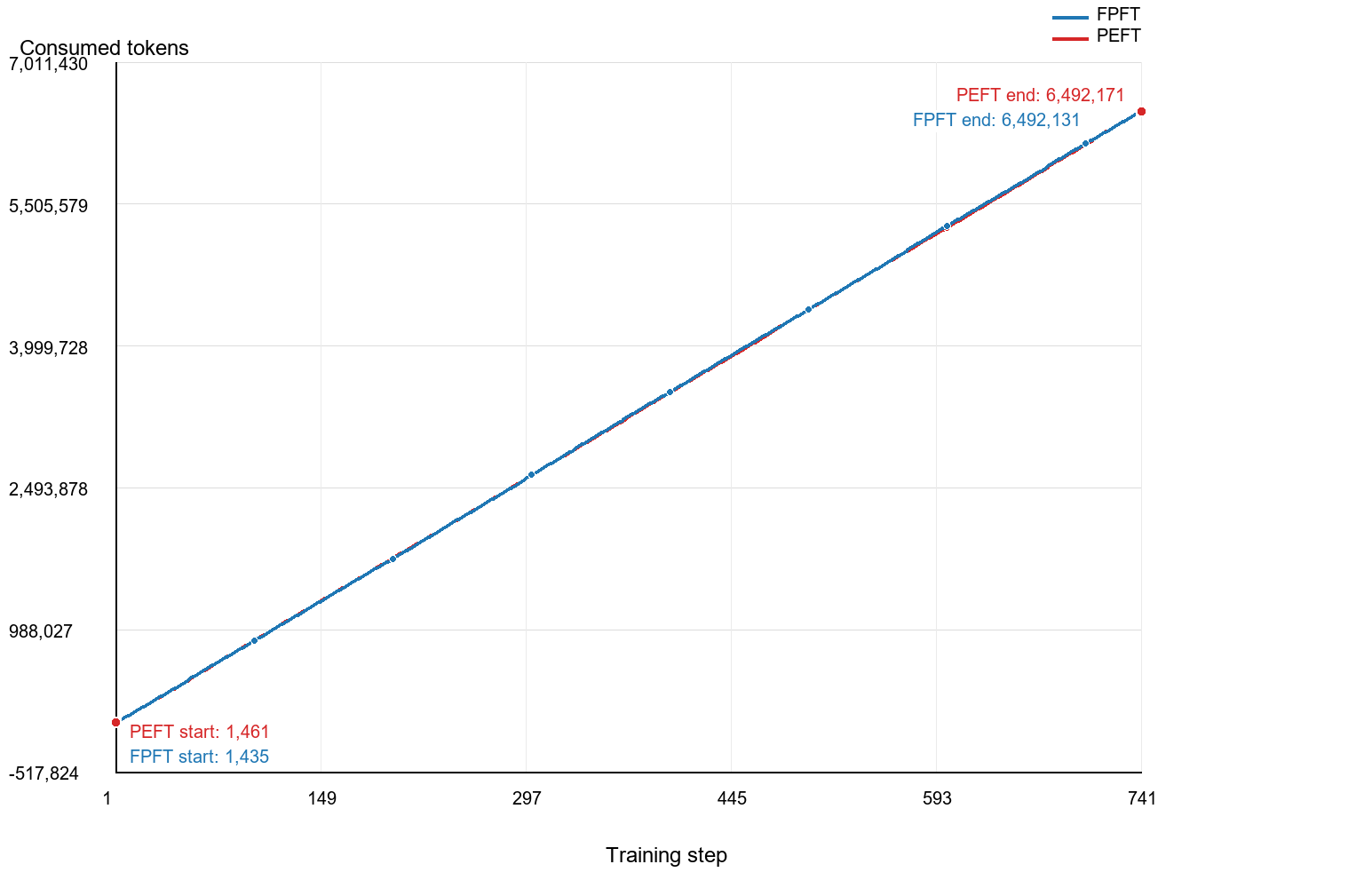

Supplement: S3 File — (ZIP) [file pone.0352256.s003.zip › Fig4.tif]

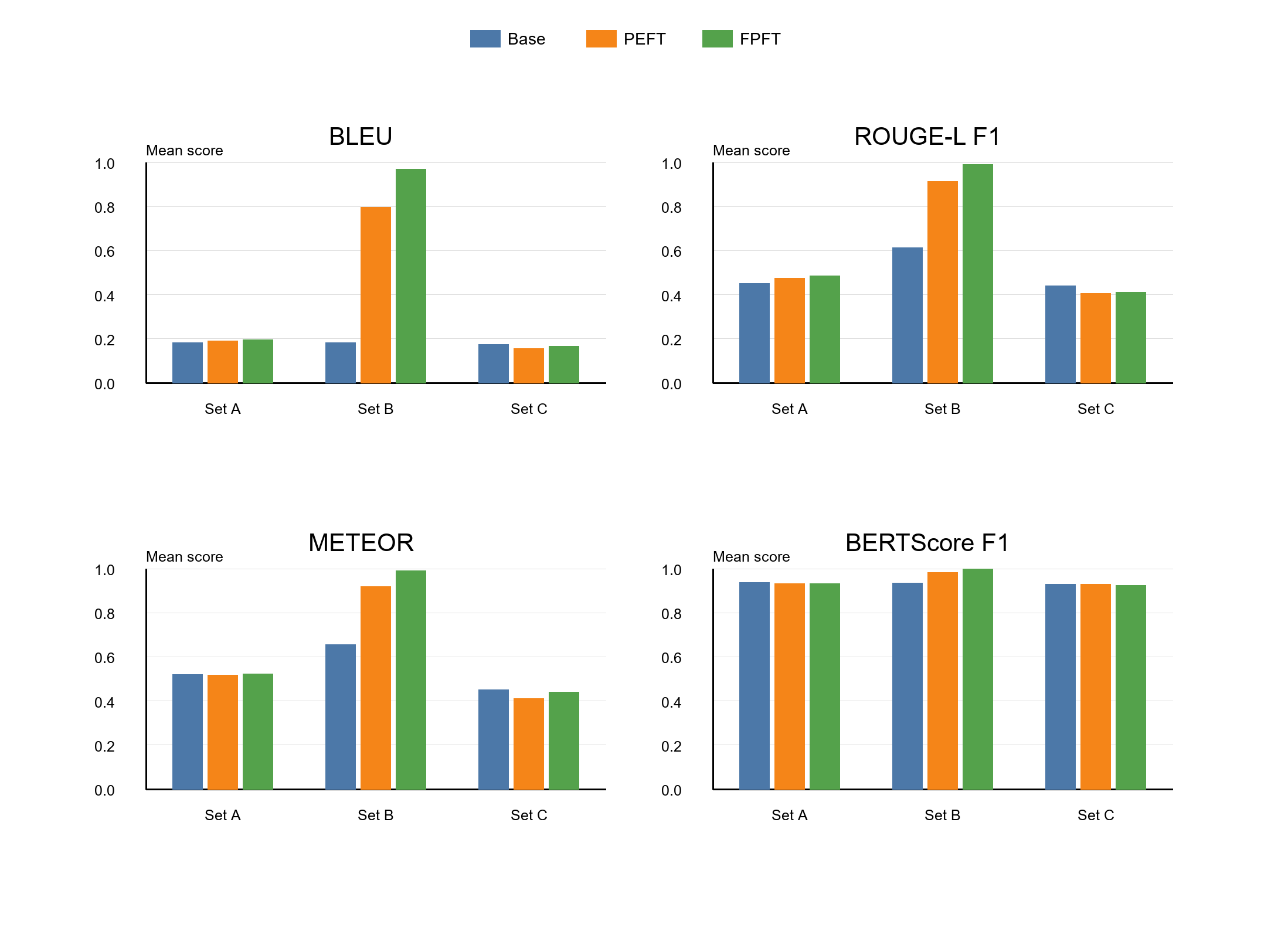

Supplement: S3 File — (ZIP) [file pone.0352256.s003.zip › Fig5.tif]
