# Supplementary material for: Textual overlap rather than domain alignment: A comparative study of fine-tuning strategies for specialised machine translation with large language models
Source: PLoS One. 2026 Jul 20;21(7):e0352256. doi: 10.1371/journal.pone.0352256 (PMC13384323; doi:10.1371/journal.pone.0352256)
Supplement: Attachment — Submitted filename: Review.pdf [file pone.0352256.s004.pdf]

Recommendation: Major Revision

Overall Assessment

The manuscript presents a comparative study on the use of Full-Parameter Fine-Tuning (FPFT) and Parameter-Efficient Fine-Tuning (PEFT) in large language models for specialized machine translation. Based on an experiment conducted with Chinese–English political discourse data using the Qwen-3-14B model, it is indicated that the choice of technique, as well as the selection of the model, depends on the characteristics of the dataset and the constraints imposed by the available computational resources.

Strengths

- The manuscript addresses an important problem in the field of Natural Language Processing, namely the comparative evaluation of Full-Parameter Fine-Tuning (FPFT) and Parameter-Efficient Fine-Tuning (PEFT) strategies for specialized machine translation in large language models.
- The experiments are conducted using the Qwen-3-14B model, which represents a modern architecture and increases the relevance of the findings for current research on large language models.
- The study is supported by experimental evaluation using Chinese–English political discourse data, providing practical evidence for the discussion of fine-tuning strategies.
- The manuscript appropriately highlights the role of computational resources in selecting between FPFT and PEFT approaches, which is highly relevant for real-world deployments.
- The use of unseen domain data (Set A) and familiar domain data (Set B) in Section 4.5 demonstrates an effort to assess model generalization and domain adaptation, which is a strong methodological choice.
- The results contribute to understanding how different fine-tuning approaches perform under specific domain and resource conditions, offering insights useful for practitioners.

Weaknesses

- The role of the Bailian platform in the dataset construction process is not sufficiently explained, which reduces transparency and reproducibility.
- The motivation for normalizing epochs instead of presenting training steps directly on the X-axis is not clearly justified. The methodological details of this normalization process are also not adequately described.
- The comparison in Figure 4 might be more effectively illustrated by using training steps on the X-axis rather than normalized epochs.
- The description of Dataset B suggests that it may contain data drawn from the same corpus used during training, which would undermine the intended

comparison with unseen data and weaken the validity of the evaluation design.

Major Comments

1. On page 8, it is suggested that the use of the Bailian platform be better contextualized with respect to its role in the construction of the dataset.
2. On page 10, it is recommended that the first paragraph of Section 4.1 be revised so that the initial loss value reported in the text is consistent with the value presented in Figure 1. In addition, the visibility of the final loss indication in Figure 1 should be improved. In the same paragraph, the motivation for normalizing the epochs is not clearly explained. It would be useful to clarify the disadvantages of presenting the training steps directly on the X-axis of the loss graph instead of the normalized epoch format currently shown in the figures. It may therefore be beneficial to include an explanatory paragraph in the Research Methods section describing both the motivation for the normalization procedure and the methodological details of how it was performed.
3. Section 4.5 adopts a comparative approach based on the use of unseen domain data (Set A) and familiar domain data (Set B), which represents a well-conceived evaluation strategy. However, the description of the data extraction procedure, particularly the statement “This set was drawn from the same corpus used for training,” suggests that the data may already be known to the model, thereby weakening the validity of the comparison. It is therefore recommended that Dataset B be constructed using texts that were not seen by the model during training but that still belong to the same domain as the training data. This adjustment would ensure better alignment with the characterization of the dataset as “Familiar In-Domain Data.”

Minor Comments

1. On page 12, the paragraph titled “Magnitude and Practical Significance” appears to contain redundant information. A revision aimed at eliminating repetition and improving conciseness is recommended.
2. Presenting the training steps on the X-axis in Figure 4 may allow the performance comparison between the two approaches to be demonstrated more effectively.

Recommendation

Major Revision

The manuscript addresses a relevant topic and presents a useful empirical comparison of fine-tuning strategies for large language models. However, several

issues related to methodological clarity, dataset construction, and figure presentation should be addressed before publication.
