# Supplementary material for: Textual overlap rather than domain alignment: A comparative study of fine-tuning strategies for specialised machine translation with large language models
Source: PLoS One. 2026 Jul 20;21(7):e0352256. doi: 10.1371/journal.pone.0352256 (PMC13384323; doi:10.1371/journal.pone.0352256)
Supplement: Attachment — Submitted filename: Review 2 - Reviewr 1.pdf [file pone.0352256.s006.pdf]

Recommendation: Minor Revision

Overall Assessment

The manuscript presents a comparative study on the use of Full-Parameter Fine-Tuning (FPFT) and Parameter-Efficient Fine-Tuning (PEFT) in large language models for specialized machine translation. Based on an experiment conducted with Chinese–English political discourse data using the Qwen-3-14B model, it is indicated that the choice of technique, as well as the selection of the model, depends on the characteristics of the dataset and the constraints imposed by the available computational resources.

The manuscript addresses a relevant topic within Natural Language Processing and provides a well-structured and methodologically sound contribution. Only minor enhancements are required to further strengthen the robustness of the experimental evaluation.

Strengths

- The manuscript addresses an important problem in the field of Natural Language Processing, namely the comparative evaluation of Full-Parameter Fine-Tuning (FPFT) and Parameter-Efficient Fine-Tuning (PEFT) strategies for specialized machine translation in large language models.
- The experiments are conducted using the Qwen-3-14B model, which represents a modern architecture and increases the relevance of the findings for current research on large language models.
- The study is supported by experimental evaluation using Chinese–English political discourse data, providing practical evidence for the discussion of fine-tuning strategies.
- The manuscript appropriately highlights the role of computational resources in selecting between FPFT and PEFT approaches, which is highly relevant for real-world deployments.
- The use of unseen domain data (Set A) and familiar domain data (Set B) in Section 4.5 demonstrates an effort to assess model generalization and domain adaptation, which is a strong methodological choice.
- The results contribute to understanding how different fine-tuning approaches perform under specific domain and resource conditions, offering insights useful for practitioners.
- Statistical comparisons of the results are presented, contributing to the reliability of the findings.

Weaknesses

- The evaluation is limited by the use of a single primary metric, which restricts the comprehensiveness and robustness of the performance analysis.

Minor Comments

1. The inclusion of a third dataset composed of cases that are semantically similar to Set B is recommended. This addition would strengthen the discussion on domain specialization by enabling a more nuanced evaluation of generalization within closely related semantic contexts.
2. The evaluation framework should be expanded to include additional metrics, such as ROUGE, METEOR, and BERTScore. The use of multiple complementary metrics would enhance the scientific robustness of the results. This recommendation becomes particularly important if the suggested third dataset is incorporated, as it would allow for a more comprehensive and reliable comparison across different evaluation scenarios.

Recommendation

Minor Revision

The manuscript addresses a relevant topic and presents a useful empirical comparison of fine-tuning strategies for large language models. The study is now well structured and methodologically sound, with only minor enhancements suggested to further strengthen the experimental evaluation. Two additional points are proposed which would further improve the robustness of the research.
