# Supplementary material for: Textual overlap rather than domain alignment: A comparative study of fine-tuning strategies for specialised machine translation with large language models
Source: PLoS One. 2026 Jul 20;21(7):e0352256. doi: 10.1371/journal.pone.0352256 (PMC13384323; doi:10.1371/journal.pone.0352256)
Supplement: Attachment — Submitted filename: Response to Reviewers.docx [file pone.0352256.s007.docx]

**Response to Academic Editor and Reviewers**

**1.Response to the Academic Editor**

**1.1 Financial disclosure**

In response to the editorial request regarding financial disclosure, we revised the cover letter to include the updated funding statement: this work was supported by the Tianjin Higher Education Postgraduate Education Reform Research Project, “Development of an Intelligent Teaching Platform for Master of Translation and Interpreting Based on Large Language Models and Practice of the EPIP Teaching Model” (Grant No. TJYG25116). We also clarified that the funder had no role in study design, data collection and analysis, decision to publish, or preparation of the manuscript.

**1.2 Laboratory protocol / reproducibility**

In response to the editor’s recommendation regarding protocol sharing, we prepared and deposited a detailed step-by-step protocol in protocols.io for the revised submission. The protocol documents the full workflow of the study, including corpus preparation, ChatML formatting, train/validation splitting, FPFT and LoRA-based PEFT fine-tuning on Alibaba Cloud Bailian, training-log extraction, generation of PLOS-ready TIFF figures, test-set construction, BLEU-4 evaluation, pass-rate log-likelihood testing, and paired Cohen’s $\text{d}_{\text{z}}$ calculation for item-level BLEU-score differences. The protocol DOI has been added to the Methods section of the revised manuscript: https://dx.doi.org/10.17504/protocols.io.kqdg3mj5zl25/v1. For the convenience of the editor and reviewers during peer review, we also provide the following private reviewer link: https://www.protocols.io/private/FE0C0C8647AE11F18CC70A58A9FEAC02. This private link will be removed before publication.

**1.3 ORCID ID verification**

In response to the editorial note regarding ORCID IDs, we confirm that the corresponding author’s ORCID ID has already been verified in the submission system. In addition, the two coauthors have been informed to register for an ORCID ID and to enter and verify their ORCID IDs in the submission system. We have also reminded them that only individually verified ORCID IDs can appear in the published article, and we will ensure that the verification step is completed before final acceptance.

**1.4 PLOS ONE style requirements and file naming**

We carefully checked the revised submission against the PLOS ONE formatting guidance and updated the manuscript accordingly. Specifically, we standardized section headings, added separate figure captions, added Supporting Information captions, and revised the file naming and supporting-file descriptions to better align with the journal’s requirements. In addition, following the format used in published PLOS ONE articles, we reformatted the in-text references from author–year citations to bracketed numerical citations (e.g., [1]) and reordered the reference list according to the order of first appearance in the manuscript. We also checked the technical presentation of Figures 1-4 and regenerated the figure files as 300 dpi TIFF files with figure titles omitted from the image files, so that the figure files better conform to PLOS figure-preparation guidance.

**1.5 Code sharing**

In response to the journal’s code-sharing requirement, we added a Code Availability Statement to the revised manuscript. We also clarified that the Python scripts used to extract the Alibaba Cloud Bailian training logs and generate the revised figures will be provided as Supporting Information for peer review and made openly available upon publication in accordance with PLOS ONE policy.

**1.6 Separate figure captions**

We added a separate caption for each figure in the revised manuscript. In particular, Figures 1-4 now each have their own standalone caption immediately following the corresponding figure.

**1.7 Data availability and sharing plan**

We revised the Data Availability Statement to clarify the scope of data sharing more explicitly. The revised statement explains that the underlying materials include bilingual political texts derived in part from officially published sources, and that the full bilingual corpus cannot be redistributed in its entirety because some source texts and published translations may be subject to copyright or reuse restrictions. To support transparency and reproducibility, we provide corpus-source metadata, selection criteria, test-set definitions, field explanations, and processing notes in S1 File. Evaluation files for Test Sets A and B and aggregated BLEU-based comparison tables are provided in S2 Dataset.

**1.8 English-language version of the supporting dataset materials**

In response to the editorial request, we prepared an English-language supporting file and an English-language summary workbook for the revised submission. Specifically, S1 provides an English-language description of the shared data materials and metadata, including corpus sources, test-set definitions, field explanations, and evaluation use. S2 provides an English-language workbook summarizing Test Sets A and B together with aggregated BLEU-4 results, pass-rate summaries, log-likelihood comparisons based on pass-count distributions, and paired Cohen’s $\text{d}_{\text{z}}$ effect-size calculations corresponding to the revised manuscript Table 3.

**1.9 Accessibility of the supporting Python script file**

We checked the supporting file containing the figure-generation Python scripts, revised the file organization, and prepared it for re-upload as an accessible ZIP package (S3_Code_Figure_Generation_Scripts.zip) containing the simplified figure-generation script, the relevant FPFT/PEFT training-log files, the generated figure files, and a README. We also updated the script so that it regenerates Figures 1-4 as PLOS-ready TIFF files (300 dpi, RGB) with figure titles omitted from the image files. In addition, Figure 3 was regenerated with a logarithmic y-axis to make both learning-rate schedules visible, and Figure 4 was redrawn with adjusted line ordering, markers, and endpoint labels to clarify the near-overlap in token consumption. The README was revised accordingly to document the output format and usage.

**1.10 Supporting Information captions and in-text citations**

We added captions for the Supporting Information files at the end of the revised manuscript and updated the corresponding in-text references accordingly, in line with the journal’s Supporting Information guidelines. The revised manuscript now refers explicitly to S1 File, which provides the English-language data description; S2 Dataset, which provides the evaluation workbook and aggregated BLEU-4/pass-rate/effect-size summaries; and S3 File, which provides the figure-generation Python scripts, FPFT/PEFT training logs, and PLOS-ready TIFF figure files.

**1.11 Clarification of statistical testing and effect-size calculation**

During the final consistency check of the revised manuscript and supporting files, we refined the description and reporting of the statistical analysis to avoid ambiguity. Log-likelihood ratio tests are now explicitly reported as comparisons of pass-rate distributions, with corpus size set to 50 for each model and frequency defined as the number of translations reaching the operational threshold of BLEU ≥ 0.4. Effect sizes for item-level BLEU-score differences are now reported as paired Cohen’s $\text{d}_{\text{z}}$, calculated from paired BLEU-score differences across the same 50 test items. The revised Table 3 therefore integrates descriptive BLEU-4 results, pass-rate summaries, log-likelihood comparisons, and paired Cohen’s $\text{d}_{\text{z}}$ values in a single table. This clarification improves statistical transparency and ensures that the Methods, Results, Supporting Information, and protocol are fully consistent.

**2.Response to Reviewer #1**

**2.1 Comment on the role of the Bailian platform in corpus construction**

We thank the reviewer for this important comment. In the revised manuscript, we substantially clarified the role of the Alibaba Cloud Bailian platform in Section 3.1. We now distinguish more explicitly between corpus construction and model fine-tuning: the corpus itself was assembled from officially published bilingual political materials, whereas Bailian was used only for supervised fine-tuning, token accounting, and model deployment configuration. We also expanded the description of the corpus sources, document types, time range, and quality-control procedures to improve methodological transparency and reproducibility.

**2.2 Comment on Figure 1, initial loss values, and the x-axis design**

We appreciate this careful observation. In response, we revisited the original training logs and corrected the reported initial loss values in Section 4.1 so that they are fully consistent with the empirical log data. We also improved the readability of Figure 1 by explicitly labeling the starting and final loss values. More importantly, instead of retaining normalized epochs as the principal x-axis, we revised the training-diagnostic figures to use raw training steps. This directly addresses the reviewer’s concern about interpretability and makes the comparison between FPFT and PEFT more transparent. The corresponding methodological description in the Research Methods section was also revised accordingly.

**2.3 Comment on Set B and the validity of the evaluation design**

We are grateful for this thoughtful comment. We agree that, if Set B is drawn directly from the fine-tuning corpus, it should not be interpreted as a conventional unseen in-domain test set. Rather than preserving the earlier wording, we revised the manuscript to redefine Set B explicitly as a maximum-overlap benchmark. In Sections 3.3, 4.5, 5.1, and 5.4, we now clarify that Set B is used to estimate performance under the highest possible degree of training-test overlap, whereas Set A remains the benchmark for unseen in-domain evaluation. This revision avoids overstating generalization and makes the interpretation of the results more rigorous and transparent.

**2.4 Comment on redundancy in the “Magnitude and Practical Significance” paragraph**

In response to the reviewer’s comment, we revised the paragraph on throughput magnitude and practical significance to eliminate redundancy and improve conciseness. We also recalculated the interpretation using the final and late-stage logged throughput values. The revised manuscript now reports that PEFT maintained an approximately 21%-22% throughput advantage over FPFT, with final logged speeds of 0.53545 iter/s for PEFT and 0.440708 iter/s for FPFT. The practical interpretation was also compressed into a direct statement that PEFT can complete the same training schedule in less time or execute more optimization steps within the same wall-clock budget.

**2.5 Comment on using training steps in Figure 4**

As suggested, we revised Figure 4 so that the x-axis is expressed in training steps rather than normalized epochs. We also rechecked the consumed-token logs and updated the quantitative description accordingly. The revised manuscript now reports final token totals of 6,492,131 for FPFT and 6,492,171 for PEFT, a difference of only 40 tokens, indicating essentially identical token exposure under the matched three-epoch schedule. Figure 4 was also redrawn with clearer markers and endpoint labels to make the near-overlapping trajectories easier to interpret.

**3.Response to Reviewer #2**

**3.1 Comment on corpus construction details and reproducibility**

We thank the reviewer for this constructive suggestion. In the revised manuscript, we substantially expanded Section 3.1 to provide a clearer description of the corpus sources, document types, time range, and selection criteria. We now specify that the corpus was compiled from officially published Chinese-English political materials issued between 2014 and 2024, including sources such as *Xi Jinping: The Governance of China*, *100 Years of the Communist Party of China*, and annual institutional reports of the NPC and CPPCC. We also added explicit inclusion and exclusion rules, as well as the major quality-control principles applied during corpus preparation, to improve reproducibility.

**3.2 Comment on ChatML conversion, bidirectional training, and corpus processing**

We agree that these procedural details should be made more explicit. Accordingly, we expanded the Methods section to describe the ChatML structure used for supervised fine-tuning, including the roles of the system prompt, user instruction, and assistant reference translation. We also clarified how bidirectional training data were constructed by reversing the translation direction in the system prompt and swapping source and target texts. In addition, we now explain the corpus-cleaning process more clearly, including the removal of duplicate or near-duplicate entries, incomplete segments, evidently mistranslated pairs, and severely noisy or non-parallel records.

**3.3 Comment on the analysis of loss convergence**

We appreciate this insightful comment and have deepened the analysis in both the Results and Discussion sections. The revised manuscript now explains more clearly that FPFT updates the full parameter space and therefore reduces loss rapidly but with greater instability and sensitivity to local noise, whereas PEFT, implemented via LoRA, constrains adaptation to a low-rank parameter subspace, which yields a smoother and more regularized optimization trajectory. We also retained the key step-based observations from the training-loss curve, including the faster early descent of FPFT, its broader middle-phase oscillations, and the more controlled decline of PEFT under the same training schedule. We further clarified that the smoother PEFT trajectory did not, under the present training configuration, translate into the lowest final training loss; FPFT ultimately achieved the lower final logged loss, while PEFT showed greater optimization stability. This revision strengthens the logical connection between the observed convergence patterns and the underlying fine-tuning mechanisms.

**3.4 Comment on the slightly lower PEFT performance on unseen data**

We agree that the slightly lower PEFT result on unseen Test Set A required further explanation. In the revised manuscript, we therefore added a more explicit and cautious discussion of this pattern. Specifically, we note that PEFT obtained a mean BLEU score of 0.0816 on Test Set A, compared with 0.1214 for the base model and 0.1164 for FPFT. Log-likelihood ratio tests based on pass-rate distributions showed no statistically significant differences between model pairs on Test Set A (all p > 0.05), and the paired Cohen’s $\text{d}_{\text{z}}$ values for item-level BLEU-score differences remained below the 0.2 threshold for a small effect. We further explain that LoRA-based PEFT concentrates adaptation in a low-rank parameter subspace, which can efficiently encode recurring patterns from the fine-tuning corpus, but may offer limited transfer benefit when evaluation items share little direct overlap with those patterns. We therefore interpret the lower PEFT mean as a plausible consequence of concentrated task-specific adaptation under low-overlap conditions, rather than as definitive evidence that LoRA inherently harms generalisation.

**3.5 Comment on the limitations section**

We appreciate this helpful suggestion and substantially expanded Section 5.4 to provide a fuller account of the study’s limitations. In the revised manuscript, we now explicitly acknowledge that the modelling evidence is based on a single base model (Qwen-3-14B), that the PEFT condition includes only LoRA rather than multiple parameter-efficient methods, and that the current data design includes only one training scale, one unseen in-domain evaluation set, and one maximum-overlap benchmark. We also clarify two further boundaries of interpretation: first, that evaluation relied primarily on BLEU-4 and therefore does not fully capture semantic and stylistic aspects of political translation; and second, that Test Set B represents a maximum-overlap benchmark sampled directly from the fine-tuning corpus rather than a held-out in-domain condition, which limits the extent to which those gains can be interpreted as transferable beyond matched materials. We believe this revision provides a more transparent and balanced statement of the study’s scope.

**3.6 Comment on future research directions**

We also revised the future-research discussion to make it more specific and actionable. Specifically, the revised manuscript now proposes four concrete directions: (i) testing whether the present findings generalise across additional base models and model families, (ii) comparing LoRA with other PEFT methods such as Adapters and Prefix-Tuning in the same political-translation setting, (iii) systematically varying training-data scale and overlap ratio to examine their relationship with fine-tuning benefit, and (iv) extending the evaluation framework by combining BLEU with human assessment and semantic-aware metrics such as COMET and chrF. These additions were made to ensure that the future-work section responds directly to the reviewer’s request for implementable research directions.

**3.7 Comment on terminology, repetition, and academic style**

We carefully revised the manuscript to reduce repetition, standardize terminology, and remove colloquial phrasing. In particular, repetitive statements of the main findings were reduced across the Abstract, Results, Discussion, and Conclusion sections, and several informal or contrastive expressions were replaced with more neutral academic wording. We also carried out an additional line-by-line language check to improve grammatical consistency, punctuation, and the uniform use of stylistic conventions throughout the manuscript. We also revised the Discussion and Conclusion so that the interpretation is presented more clearly as part of the manuscript’s substantive argument. In particular, the revised text now consistently distinguishes unseen in-domain evaluation on Test Set A from maximum-overlap benchmarking on Test Set B, and avoids treating the Set B gains as evidence of broad unseen in-domain generalisation. We also standardised the statistical terminology by distinguishing pass-rate log-likelihood testing from paired Cohen’s $\text{d}_{\text{z}}$ effect-size interpretation.

**3.8 Comment on adding an abbreviation table**

Following the reviewer’s recommendation, we added an Abbreviations table before the main text in the revised manuscript. The table includes the principal abbreviations used throughout the paper, including LLM, FPFT, PEFT, LoRA, SFT, ChatML, BLEU-4, COMET, chrF, and LR, to improve readability and reduce ambiguity for readers from different disciplinary backgrounds.
