# Supplementary material for: Textual overlap rather than domain alignment: A comparative study of fine-tuning strategies for specialised machine translation with large language models
Source: PLoS One. 2026 Jul 20;21(7):e0352256. doi: 10.1371/journal.pone.0352256 (PMC13384323; doi:10.1371/journal.pone.0352256)
Supplement: Attachment — Submitted filename: Response_to_Reviewers_auresp_2.docx [file pone.0352256.s008.docx]

Response to Academic Editor and Reviewers

Manuscript ID: PONE-D-26-09149R1

Title: Textual Overlap Rather Than Domain Alignment: A Comparative Study of Fine-Tuning Strategies for Specialised Machine Translation with Large Language Models

**Response to the Academic Editor**

We appreciate the opportunity to revise the manuscript. In response to the Academic Editor’s and reviewer’s comments, we have submitted a revised manuscript, an updated protocol(DOI: https://dx.doi.org/10.17504/protocols.io.kqdg3re7pg25/v1 [Private link for reviewers: https://www.protocols.io/private/83938F755CCD11F18D230A58A9FEAC02 to be removed before publication.]), revised supporting files, and this response letter. The main revision expands the evaluation design to include Test Sets A, B, and C and four complementary automatic translation-quality metrics: BLEU, ROUGE-L F1, METEOR, and BERTScore F1. The revised S2 Dataset contains test-set metadata, item-level metric scores, aggregated metric summaries, BLEU pass-rate analyses, paired t-tests with paired effect sizes, and Fig 5 source data; the revised S3 File contains the scripts and source files needed to reproduce the metric scores, figure files, and table-data outputs.

**Response to Reviewer #1**

**Comment 1**: The inclusion of a third dataset composed of cases that are semantically similar to Set B is recommended. This addition would strengthen the discussion on domain specialization by enabling a more nuanced evaluation of generalization within closely related semantic contexts.

**Response**: Thank you for this helpful suggestion. We constructed Test Set C as a 50-item political-discourse test set that is semantically related to Set B but was not included in the fine-tuning corpus. Test Set C belongs to the same broad Chinese-English political-discourse domain and is close to Set B in content type, but its source texts and reference translations were not used for fine-tuning. This allowed us to test whether the strong performance observed on the maximum-overlap Set B transfers to closely related but non-overlapping material. Across the four automatic metrics, Set C did not show the maximum-overlap gains observed on Set B; BLEU pass rates were 12% for the base model, 8% for PEFT, and 8% for FPFT, with no significant pass-rate differences between model pairs. This strengthens the central conclusion that textual overlap, rather than broad domain similarity alone, conditions the observed benefit of fine-tuning. We revised the Methods, Results, Discussion, Conclusion, protocol, S1 File, and S2 Dataset accordingly.

**Comment 2:** The evaluation framework should be expanded to include additional metrics, such as ROUGE, METEOR, and BERTScore. The use of multiple complementary metrics would enhance the scientific robustness of the results.

**Response**: We agree and have expanded the evaluation framework. In addition to BLEU, we computed ROUGE-L F1, METEOR, and BERTScore F1 for all translations produced by the base model, PEFT model, and FPFT model on Test Sets A, B, and C. BERTScore F1 was computed using the official bert-score 0.3.12 package with a local roberta-large model and the recommended English layer 17. The exact metric configuration is now documented in S1 File and the S3 File README: sacrebleu 2.6.0 sentence BLEU on a 0-1 scale, word-level ROUGE-L F1, NLTK 3.9.1 meteor_score, and official bert-score 0.3.12 with local roberta-large. The revised Table 3 reports the mean values of all four metrics, and Fig 5 visualizes the metric patterns across models and test sets. The updated S2 Dataset includes item-level metric scores, aggregated metric summaries, BLEU pass-rate likelihood-ratio G^2^ tests, paired t-tests for the four continuous metrics, and paired Cohen’s $d_{z}$ effect-size values. In the revised Results and Discussion, we now report the corresponding t statistics, p values, and $d_{z}$ values for the key comparisons, rather than referring to paired t-tests without numerical evidence. To strengthen reproducibility, we also added calculate_translation_metrics.py to S3 File so that readers can generate item_level_metrics.csv directly from the nine source translation workbooks and then reproduce Table 3, Table 4, Table 5, and Fig 5 from the same item-level metric file. The multi-metric results support the original conclusion while making it more robust: fine-tuned models perform strongly under maximum textual overlap (Set B), but the advantage is not reproduced on the unseen Set A or the semantically related but non-overlapping Set C.
