# Supplementary material for: Textual overlap rather than domain alignment: A comparative study of fine-tuning strategies for specialised machine translation with large language models
Source: PLoS One. 2026 Jul 20;21(7):e0352256. doi: 10.1371/journal.pone.0352256 (PMC13384323; doi:10.1371/journal.pone.0352256)
Supplement: Peer review history

# Peer review: Decision Letter 0

**DOI:** 10.1371/journal.pone.0352256.r001

**Authors:** Zeheng Wang

25 Mar 2026

PONE-D-26-09149

Data Overlap over Domain Alignment: A Comparative Study of Fine-Tuning Strategies for Specialized Machine Translation with Large Language Models

PLOS One

Dear Dr. Yang,

Thank you for submitting your manuscript to PLOS ONE. After careful consideration, we feel that it has merit but does not fully meet PLOS ONE’s publication criteria as it currently stands. Therefore, we invite you to submit a revised version of the manuscript that addresses the points raised during the review process.

Please submit your revised manuscript by May 09 2026 11:59PM. If you will need more time than this to complete your revisions, please reply to this message or contact the journal office at plosone@plos.org. When you're ready to submit your revision, log on to https://www.editorialmanager.com/pone/ and select the 'Submissions Needing Revision' folder to locate your manuscript file.

Please include the following items when submitting your revised manuscript:

- A letter that responds to each point raised by the academic editor and reviewer(s). You should upload this letter as a separate file labeled 'Response to Reviewers'.
- A marked-up copy of your manuscript that highlights changes made to the original version. You should upload this as a separate file labeled 'Revised Manuscript with Track Changes'.
- An unmarked version of your revised paper without tracked changes. You should upload this as a separate file labeled 'Manuscript'.

If you would like to make changes to your financial disclosure, please include your updated statement in your cover letter. Guidelines for resubmitting your figure files are available below the reviewer comments at the end of this letter.

If applicable, we recommend that you deposit your laboratory protocols in protocols.io to enhance the reproducibility of your results. Protocols.io assigns your protocol its own identifier (DOI) so that it can be cited independently in the future. For instructions see: https://journals.plos.org/plosone/s/submission-guidelines#loc-laboratory-protocols. Additionally, PLOS ONE offers an option for publishing peer-reviewed Lab Protocol articles, which describe protocols hosted on protocols.io. Read more information on sharing protocols at https://plos.org/protocols?utm_medium=editorial-email&utm_source=authorletters&utm_campaign=protocols.

As the corresponding author, your ORCID iD is verified in the submission system and will appear in the published article. PLOS supports the use of ORCID, and we encourage all coauthors to register for an ORCID iD and use it as well. Please encourage your coauthors to verify their ORCID iD within the submission system before final acceptance, as unverified ORCID iDs will not appear in the published article. *Only*  the individual author can complete the verification step; PLOS staff *cannot*  verify ORCID iDs on behalf of authors.

We look forward to receiving your revised manuscript.

Kind regards,

Zeheng Wang

Academic Editor

PLOS One

Journal requirements:

When submitting your revision, we need you to address these additional requirements.

1. Please ensure that your manuscript meets PLOS ONE's style requirements, including those for file naming. The PLOS ONE style templates can be found at

https://journals.plos.org/plosone/s/file?id=wjVg/PLOSOne_formatting_sample_main_body.pdf and

https://journals.plos.org/plosone/s/file?id=ba62/PLOSOne_formatting_sample_title_authors_affiliations.pdf

2. Please note that PLOS One has specific guidelines on code sharing for submissions in which author-generated code underpins the findings in the manuscript. In these cases, we expect all author-generated code to be made available without restrictions upon publication of the work. Please review our guidelines at https://journals.plos.org/plosone/s/materials-and-software-sharing#loc-sharing-code and ensure that your code is shared in a way that follows best practice and facilitates reproducibility and reuse.

3. Please include a separate caption for each figure in your manuscript.

4. When completing the data availability statement of the submission form, you indicated that you will make your data available on acceptance. We strongly recommend all authors decide on a data sharing plan before acceptance, as the process can be lengthy and hold up publication timelines. Please note that, though access restrictions are acceptable now, your entire data will need to be made freely accessible if your manuscript is accepted for publication. This policy applies to all data except where public deposition would breach compliance with the protocol approved by your research ethics board. If you are unable to adhere to our open data policy, please kindly revise your statement to explain your reasoning and we will seek the editor's input on an exemption. Please be assured that, once you have provided your new statement, the assessment of your exemption will not hold up the peer review process.

5. Thank you for providing your underlying data as Supporting Information.

We note that the data set contains text or data that is not in English. Please note that PLOS is an English-language publisher, so we require data sets to be provided in English as well. Please upload an English-language version of your data set.

This will also allow us to determine if your data follows PLOS standards per our Data Availability policy here: https://journals.plos.org/plosone/s/data-availability

6. We are unable to open your Supporting Information file [figures' python script]. Please kindly revise as necessary and re-upload.

7. Please include captions for your Supporting Information files at the end of your manuscript, and update any in-text citations to match accordingly. Please see our Supporting Information guidelines for more information: http://journals.plos.org/plosone/s/supporting-information.

If the reviewer comments include a recommendation to cite specific previously published works, please review and evaluate these publications to determine whether they are relevant and should be cited. There is no requirement to cite these works unless the editor has indicated otherwise.

[Note: HTML markup is below. Please do not edit.]

Reviewers' comments:

Reviewer's Responses to Questions

**Comments to the Author**

1. Is the manuscript technically sound, and do the data support the conclusions?

The manuscript must describe a technically sound piece of scientific research with data that supports the conclusions. Experiments must have been conducted rigorously, with appropriate controls, replication, and sample sizes. The conclusions must be drawn appropriately based on the data presented.

Reviewer #1: Yes

Reviewer #2: Yes

**********

2. Has the statistical analysis been performed appropriately and rigorously?

Reviewer #1: Yes

Reviewer #2: Yes

**********

3. Have the authors made all data underlying the findings in their manuscript fully available?

The PLOS Data policy requires authors to make all data underlying the findings described in their manuscript fully available without restriction, with rare exception (please refer to the Data Availability Statement in the manuscript PDF file). The data should be provided as part of the manuscript or its supporting information, or deposited to a public repository. For example, in addition to summary statistics, the data points behind means, medians and variance measures should be available. If there are restrictions on publicly sharing data—e.g. participant privacy or use of data from a third party—those must be specified.

Reviewer #1: Yes

Reviewer #2: Yes

**********

4. Is the manuscript presented in an intelligible fashion and written in standard English?

PLOS ONE does not copyedit accepted manuscripts, so the language in submitted articles must be clear, correct, and unambiguous. Any typographical or grammatical errors should be corrected at revision, so please note any specific errors here.

Reviewer #1: Yes

Reviewer #2: Yes

**********

5. Review Comments to the Author

Please use the space provided to explain your answers to the questions above. You may also include additional comments for the author, including concerns about dual publication, research ethics, or publication ethics. (Please upload your review as an attachment if it exceeds 20,000 characters)

Reviewer #1: Overall Assessment:

The manuscript presents a comparative study on the use of Full-Parameter Fine-Tuning (FPFT) and Parameter-Efficient Fine-Tuning (PEFT) in large language models for specialized machine translation. Based on an experiment conducted with Chinese–English political discourse data using the Qwen-3-14B model, it is indicated that the choice of technique, as well as the selection of the model, depends on the characteristics of the dataset and the constraints imposed by the available computational resources.

Strengths:

- The manuscript addresses an important problem in the field of Natural Language Processing, namely the comparative evaluation of Full-Parameter Fine-Tuning (FPFT) and Parameter-Efficient Fine-Tuning (PEFT) strategies for specialized machine translation in large language models.

- The experiments are conducted using the Qwen-3-14B model, which represents a modern architecture and increases the relevance of the findings for current research on large language models.

- The study is supported by experimental evaluation using Chinese–English political discourse data, providing practical evidence for the discussion of fine-tuning strategies.

- The manuscript appropriately highlights the role of computational resources in selecting between FPFT and PEFT approaches, which is highly relevant for real-world deployments.

- The use of unseen domain data (Set A) and familiar domain data (Set B) in Section 4.5 demonstrates an effort to assess model generalization and domain adaptation, which is a strong methodological choice.

- The results contribute to understanding how different fine-tuning approaches perform under specific domain and resource conditions, offering insights useful for practitioners.

Weaknesses:

- The role of the Bailian platform in the dataset construction process is not sufficiently explained, which reduces transparency and reproducibility.

- The motivation for normalizing epochs instead of presenting training steps directly on the X-axis is not clearly justified. The methodological details of this normalization process are also not adequately described.

- The comparison in Figure 4 might be more effectively illustrated by using training steps on the X-axis rather than normalized epochs.

- The description of Dataset B suggests that it may contain data drawn from the same corpus used during training, which would undermine the intended comparison with unseen data and weaken the validity of the evaluation design.

Major Comments:

1. On page 8, it is suggested that the use of the Bailian platform be better contextualized with respect to its role in the construction of the dataset.

2. On page 10, it is recommended that the first paragraph of Section 4.1 be revised so that the initial loss value reported in the text is consistent with the value presented in Figure 1. In addition, the visibility of the final loss indication in Figure 1 should be improved. In the same paragraph, the motivation for normalizing the epochs is not clearly explained. It would be useful to clarify the disadvantages of presenting the training steps directly on the X-axis of the loss graph instead of the normalized epoch format currently shown in the figures. It may therefore be beneficial to include an explanatory paragraph in the Research Methods section describing both the motivation for the normalization procedure and the methodological details of how it was performed.

3. Section 4.5 adopts a comparative approach based on the use of unseen domain data (Set A) and familiar domain data (Set B), which represents a well-conceived evaluation strategy. However, the description of the data extraction procedure, particularly the statement “This set was drawn from the same corpus used for training,” suggests that the data may already be known to the model, thereby weakening the validity of the comparison. It is therefore recommended that Dataset B be constructed using texts that were not seen by the model during training but that still belong to the same domain as the training data. This adjustment would ensure better alignment with the characterization of the dataset as “Familiar In-Domain Data.”

Minor Comments:

1. On page 12, the paragraph titled “Magnitude and Practical Significance” appears to contain redundant information. A revision aimed at eliminating repetition and improving conciseness is recommended.

2. Presenting the training steps on the X-axis in Figure 4 may allow the performance comparison between the two approaches to be demonstrated more effectively.

Recommendation: Major Revision

The manuscript addresses a relevant topic and presents a useful empirical comparison of fine-tuning strategies for large language models. However, several issues related to methodological clarity, dataset construction, and figure presentation should be addressed before publication.

Reviewer #2: Thanks for the opportunity to review this manuscript. This paper focuses on the comparison of fine-tuning strategies for large language models in specialized machine translation, taking Chinese-English translation of political discourse as the research object, exploring the performance differences between Full Parameter Fine-Tuning (FPFT) and Parameter-Efficient Fine-Tuning (PEFT), and proposing a strategy selection framework based on data overlap and resource constraints. The research topic is closely aligned with the research hotspots in the field of machine translation, the research design is relatively standardized, the experimental data is detailed, and the conclusions have certain practical guiding significance. However, the manuscript still has several issues that need to be revised and improved, and the overall suggestion is Revise Major. Please find major issues below:

1.Corpus construction details need to be supplemented to improve research reproducibility

(1)Vague description of corpus sources: The manuscript mentions that the corpus is from an "authentic state translation program", but does not specify the specific source channel, text type (e.g., government work reports, diplomatic statements, policy documents, etc.), time range, nor the screening criteria of the corpus (e.g., how to ensure the professionalism of political discourse and the accuracy of translation).

(2)Missing corpus processing details: In the process of converting bilingual texts into ChatML format and constructing a bidirectional training corpus, the specific design of system prompts and the specific rules for source-target pair exchange are not explained, nor the corpus cleaning steps (e.g., how to handle duplicate sentences, incomplete sentences, and mistranslated sentences) are mentioned.

2.The analysis of some experimental results needs to be deepened to enhance logical relevance

(1)Insufficient analysis of the causes of loss convergence: The manuscript points out that FPFT loss oscillates and PEFT loss is stable, but fails to deeply analyze the underlying reasons—for example, FPFT is prone to overfitting to noise in the training data because it updates all 14 billion parameters, while PEFT only updates a small number of low-rank matrices and has a natural regularization effect. This logical connection needs to be further clarified.

(2)Failure to explain the reason for the slightly lower performance of PEFT on unseen data: In Test Set A, the BLEU value of PEFT (0.0816) is lower than that of the base model (0.1214) and FPFT (0.1164). The manuscript only mentions that the difference is not statistically significant, but does not analyze why PEFT has a slight performance decline (e.g., whether the low-rank adaptation of LoRA has a slight negative impact on the generalization ability of the model).

3.The limitations section needs further expansion and future research directions need to be more specific

(1)Superficial description of existing limitations: The manuscript only mentions three limitations: the corpus is limited to Sino-Western political discourse, the evaluation only uses the BLEU index, and no hybrid strategies are explored. It fails to fully analyze other objective limitations of the research, such as model singularity (only Qwen-3-14B is used, and the universality of the conclusions in other large language models is not verified), PEFT method singularity (only LoRA is adopted, and no comparison with other PEFT methods such as Adapter and Prefix-Tuning is made), and data scale limitation (the impact of corpus scale on the data overlap effect is not explored).

(2)Lack of implementability in future research directions: It is suggested to refine the future research directions into executable research content, for example: ① Verify the applicability of the conclusions in different large models such as GPT, Llama, and Baichuan; ② Compare the performance differences of multiple PEFT methods in political translation; ③ Explore the quantitative relationship between data overlap and fine-tuning performance under different data scales; ④ Improve the translation quality evaluation system by combining human evaluation with semantic-aware indicators such as COMET and CHRF.

4.Some terms and expressions need to be standardized to avoid repetition and colloquialism

(1)Repetitive and colloquial expressions: Some conclusions in the manuscript (e.g., "data overlap is the core influencing factor") are repeatedly mentioned in the abstract, results, discussion, and conclusion sections, and some expressions are too colloquial (e.g., "fast but volatile vs. Stable-Controlled Optimization", "dispelling the Data-Efficiency Myth"), which is not in line with the expression norms of academic papers.

(2)Grammatical and punctuation errors: Some sentences have grammatical errors and punctuation errors (e.g., non-unified use of parentheses and dashes), which need to be proofread word by word.

(3)Add an abbreviation table: A large number of abbreviations (e.g., FPFT, PEFT, BLEU, LoRA, ChatML, etc.) appear in the manuscript. It is recommended to add an abbreviation table before the main text for the convenience of readers.

**********

6. PLOS authors have the option to publish the peer review history of their article (what does this mean?). If published, this will include your full peer review and any attached files.

If you choose “no”, your identity will remain anonymous but your review may still be made public.

**Do you want your identity to be public for this peer review?**  For information about this choice, including consent withdrawal, please see our Privacy Policy.

Reviewer #1: **Yes:**  Raphael Souza de Oliveira

Reviewer #2: No

**********

[NOTE: If reviewer comments were submitted as an attachment file, they will be attached to this email and accessible via the submission site. Please log into your account, locate the manuscript record, and check for the action link "View Attachments". If this link does not appear, there are no attachment files.]

To ensure your figures meet our technical requirements, please review our figure guidelines: https://journals.plos.org/plosone/s/figures

You may also use PLOS’s free figure tool, NAAS, to help you prepare publication quality figures: https://journals.plos.org/plosone/s/figures#loc-tools-for-figure-preparation.

NAAS will assess whether your figures meet our technical requirements by comparing each figure against our figure specifications.

Attachment Submitted filename: Review.pdf

# Author comment: Author response to Decision Letter 1

**DOI:** 10.1371/journal.pone.0352256.r002

5 May 2026

Response to Academic Editor and Reviewers

1.Response to the Academic Editor

1.1 Financial disclosure

In response to the editorial request regarding financial disclosure, we revised the cover letter to include the updated funding statement: this work was supported by the Tianjin Higher Education Postgraduate Education Reform Research Project, “Development of an Intelligent Teaching Platform for Master of Translation and Interpreting Based on Large Language Models and Practice of the EPIP Teaching Model” (Grant No. TJYG25116). We also clarified that the funder had no role in study design, data collection and analysis, decision to publish, or preparation of the manuscript.

1.2 Laboratory protocol / reproducibility

In response to the editor’s recommendation regarding protocol sharing, we prepared and deposited a detailed step-by-step protocol in protocols.io for the revised submission. The protocol documents the full workflow of the study, including corpus preparation, ChatML formatting, train/validation splitting, FPFT and LoRA-based PEFT fine-tuning on Alibaba Cloud Bailian, training-log extraction, generation of PLOS-ready TIFF figures, test-set construction, BLEU-4 evaluation, pass-rate log-likelihood testing, and paired Cohen’s dz calculation for item-level BLEU-score differences. The protocol DOI has been added to the Methods section of the revised manuscript: https://dx.doi.org/10.17504/protocols.io.kqdg3mj5zl25/v1. For the convenience of the editor and reviewers during peer review, we also provide the following private reviewer link: https://www.protocols.io/private/FE0C0C8647AE11F18CC70A58A9FEAC02. This private link will be removed before publication.

1.3 ORCID ID verification

In response to the editorial note regarding ORCID IDs, we confirm that the corresponding author’s ORCID ID has already been verified in the submission system. In addition, the two coauthors have been informed to register for an ORCID ID and to enter and verify their ORCID IDs in the submission system. We have also reminded them that only individually verified ORCID IDs can appear in the published article, and we will ensure that the verification step is completed before final acceptance.

1.4 PLOS ONE style requirements and file naming

We carefully checked the revised submission against the PLOS ONE formatting guidance and updated the manuscript accordingly. Specifically, we standardized section headings, added separate figure captions, added Supporting Information captions, and revised the file naming and supporting-file descriptions to better align with the journal’s requirements. In addition, following the format used in published PLOS ONE articles, we reformatted the in-text references from author–year citations to bracketed numerical citations (e.g., [1]) and reordered the reference list according to the order of first appearance in the manuscript. We also checked the technical presentation of Figures 1-4 and regenerated the figure files as 300 dpi TIFF files with figure titles omitted from the image files, so that the figure files better conform to PLOS figure-preparation guidance.

1.5 Code sharing

In response to the journal’s code-sharing requirement, we added a Code Availability Statement to the revised manuscript. We also clarified that the Python scripts used to extract the Alibaba Cloud Bailian training logs and generate the revised figures will be provided as Supporting Information for peer review and made openly available upon publication in accordance with PLOS ONE policy.

1.6 Separate figure captions

We added a separate caption for each figure in the revised manuscript. In particular, Figures 1-4 now each have their own standalone caption immediately following the corresponding figure.

1.7 Data availability and sharing plan

We revised the Data Availability Statement to clarify the scope of data sharing more explicitly. The revised statement explains that the underlying materials include bilingual political texts derived in part from officially published sources, and that the full bilingual corpus cannot be redistributed in its entirety because some source texts and published translations may be subject to copyright or reuse restrictions. To support transparency and reproducibility, we provide corpus-source metadata, selection criteria, test-set definitions, field explanations, and processing notes in S1 File. Evaluation files for Test Sets A and B and aggregated BLEU-based comparison tables are provided in S2 Dataset.

1.8 English-language version of the supporting dataset materials

In response to the editorial request, we prepared an English-language supporting file and an English-language summary workbook for the revised submission. Specifically, S1 provides an English-language description of the shared data materials and metadata, including corpus sources, test-set definitions, field explanations, and evaluation use. S2 provides an English-language workbook summarizing Test Sets A and B together with aggregated BLEU-4 results, pass-rate summaries, log-likelihood comparisons based on pass-count distributions, and paired Cohen’s dz effect-size calculations corresponding to the revised manuscript Table 3.

1.9 Accessibility of the supporting Python script file

We checked the supporting file containing the figure-generation Python scripts, revised the file organization, and prepared it for re-upload as an accessible ZIP package (S3_Code_Figure_Generation_Scripts.zip) containing the simplified figure-generation script, the relevant FPFT/PEFT training-log files, the generated figure files, and a README. We also updated the script so that it regenerates Figures 1-4 as PLOS-ready TIFF files (300 dpi, RGB) with figure titles omitted from the image files. In addition, Figure 3 was regenerated with a logarithmic y-axis to make both learning-rate schedules visible, and Figure 4 was redrawn with adjusted line ordering, markers, and endpoint labels to clarify the near-overlap in token consumption. The README was revised accordingly to document the output format and usage.

1.10 Supporting Information captions and in-text citations

We added captions for the Supporting Information files at the end of the revised manuscript and updated the corresponding in-text references accordingly, in line with the journal’s Supporting Information guidelines. The revised manuscript now refers explicitly to S1 File, which provides the English-language data description; S2 Dataset, which provides the evaluation workbook and aggregated BLEU-4/pass-rate/effect-size summaries; and S3 File, which provides the figure-generation Python scripts, FPFT/PEFT training logs, and PLOS-ready TIFF figure files.

1.11 Clarification of statistical testing and effect-size calculation

During the final consistency check of the revised manuscript and supporting files, we refined the description and reporting of the statistical analysis to avoid ambiguity. Log-likelihood ratio tests are now explicitly reported as comparisons of pass-rate distributions, with corpus size set to 50 for each model and frequency defined as the number of translations reaching the operational threshold of BLEU ≥ 0.4. Effect sizes for item-level BLEU-score differences are now reported as paired Cohen’s dz, calculated from paired BLEU-score differences across the same 50 test items. The revised Table 3 therefore integrates descriptive BLEU-4 results, pass-rate summaries, log-likelihood comparisons, and paired Cohen’s dz values in a single table. This clarification improves statistical transparency and ensures that the Methods, Results, Supporting Information, and protocol are fully consistent.

2.Response to Reviewer #1

2.1 Comment on the role of the Bailian platform in corpus construction

We thank the reviewer for this important comment. In the revised manuscript, we substantially clarified the role of the Alibaba Cloud Bailian platform in Section 3.1. We now distinguish more explicitly between corpus construction and model fine-tuning: the corpus itself was assembled from officially published bilingual political materials, whereas Bailian was used only for supervised fine-tuning, token accounting, and model deployment configuration. We also expanded the description of the corpus sources, document types, time range, and quality-control procedures to improve methodological transparency and reproducibility.

2.2 Comment on Figure 1, initial loss values, and the x-axis design

We appreciate this careful observation. In response, we revisited the original training logs and corrected the reported initial loss values in Section 4.1 so that they are fully consistent with the empirical log data. We also improved the readability of Figure 1 by explicitly labeling the starting and final loss values. More importantly, instead of retaining normalized epochs as the principal x-axis, we revised the training-diagnostic figures to use raw training steps. This directly addresses the reviewer’s concern about interpretability and makes the comparison between FPFT and PEFT more transparent. The corresponding methodological description in the Research Methods section was also revised accordingly.

2.3 Comment on Set B and the validity of the evaluation design

We are grateful for this thoughtful comment. We agree that, if Set B is drawn directly from the fine-tuning corpus, it should not be interpreted as a conventional unseen in-domain test set. Rather than preserving the earlier wording, we revised the manuscript to redefine Set B explicitly as a maximum-overlap benchmark. In Sections 3.3, 4.5, 5.1, and 5.4, we now clarify that Set B is used to estimate performance under the highest possible degree of training-test overlap, whereas Set A remains the benchmark for unseen in-domain evaluation. This revision avoids overstating generalization and makes the interpretation of the results more rigorous and transparent.

2.4 Comment on redundancy in the “Magnitude and Practical Significance” paragraph

In response to the reviewer’s comment, we revised the paragraph on throughput magnitude and practical significance to eliminate redundancy and improve conciseness. We also recalculated the interpretation using the final and late-stage logged throughput values. The revised manuscript now reports that PEFT maintained an approximately 21%-22% throughput advantage over FPFT, with final logged speeds of 0.53545 iter/s for PEFT and 0.440708 iter/s for FPFT. The practical interpretation was also compressed into a direct statement that PEFT can complete the same training schedule in less time or execute more optimization steps within the same wall-clock budget.

2.5 Comment on using training steps in Figure 4

As suggested, we revised Figure 4 so that the x-axis is expressed in training steps rather than normalized epochs. We also rechecked the consumed-token logs and updated the quantitative description accordingly. The revised manuscript now reports final token totals of 6,492,131 for FPFT and 6,492,171 for PEFT, a difference of only 40 tokens, indicating essentially identical token exposure under the matched three-epoch schedule. Figure 4 was also redrawn with clearer markers and endpoint labels to make the near-overlapping trajectories easier to interpret.

3.Response to Reviewer #2

3.1 Comment on corpus construction details and reproducibility

We thank the reviewer for this constructive suggestion. In the revised manuscript, we substantially expanded Section 3.1 to provide a clearer description of the corpus sources, document types, time range, and selection criteria. We now specify that the corpus was compiled from officially published Chinese-English political materials issued between 2014 and 2024, including sources such as Xi Jinping: The Governance of China, 100 Years of the Communist Party of China, and annual institutional reports of the NPC and CPPCC. We also added explicit inclusion and exclusion rules, as well as the major quality-control principles applied during corpus preparation, to improve reproducibility.

3.2 Comment on ChatML conversion, bidirectional training, and corpus processing

We agree that these procedural details should be made more explicit. Accordingly, we expanded the Methods section to describe the ChatML structure used for supervised fine-tuning, including the roles of the system prompt, user instruction, and assistant reference translation. We also clarified how bidirectional training data were constructed by reversing the translation direction in the system prompt and swapping source and target texts. In addition, we now explain the corpus-cleaning process more clearly, including the removal of duplicate or near-duplicate entries, incomplete segments, evidently mistranslated pairs, and severely noisy or non-parallel records.

3.3 Comment on the analysis of loss convergence

We appreciate this insightful comment and have deepened the analysis in both the Results and Discussion sections. The revised manuscript now explains more clearly that FPFT updates the full parameter space and therefore reduces loss rapidly but with greater instability and sensitivity to local noise, whereas PEFT, implemented via LoRA, constrains adaptation to a low-rank parameter subspace, which yields a smoother and more regularized optimization trajectory. We also retained the key step-based observations from the training-loss curve, including the faster early descent of FPFT, its broader middle-phase oscillations, and the more controlled decline of PEFT under the same training schedule. We further clarified that the smoother PEFT trajectory did not, under the present training configuration, translate into the lowest final training loss; FPFT ultimately achieved the lower final logged loss, while PEFT showed greater optimization stability. This revision strengthens the logical connection between the observed convergence patterns and the underlying fine-tuning mechanisms.

3.4 Comment on the slightly lower PEFT performance on unseen data

We agree that the slightly lower PEFT result on unseen Test Set A required further explanation. In the revised manuscript, we therefore added a more explicit and cautious discussion of this pattern. Specifically, we note that PEFT obtained a mean BLEU score of 0.0816 on Test Set A, compared with 0.1214 for the base model and 0.1164 for FPFT. Log-likelihood ratio tests based on pass-rate distributions showed no statistically significant differences between model pairs on Test Set A (all p > 0.05), and the paired Cohen’s dz values for item-level BLEU-score differences remained below the 0.2 threshold for a small effect. We further explain that LoRA-based PEFT concentrates adaptation in a low-rank parameter subspace, which can efficiently encode recurring patterns from the fine-tuning corpus, but may offer limited transfer benefit when evaluation items share little direct overlap with those patterns. We therefore interpret the lower PEFT mean as a plausible consequence of concentrated task-specific adaptation under low-overlap conditions, rather than as definitive evidence that LoRA inherently harms generalisation.

3.5 Comment on the limitations section

We appreciate this helpful suggestion and substantially expanded Section 5.4 to provide a fuller account of the study’s limitations. In the revised manuscript, we now explicitly acknowledge that the modelling evidence is based on a single base model (Qwen-3-14B), that the PEFT condition includes only LoRA rather than multiple parameter-efficient methods, and that the current data design includes only one training scale, one unseen in-domain evaluation set, and one maximum-overlap benchmark. We also clarify two further boundaries of interpretation: first, that evaluation relied primarily on BLEU-4 and therefore does not fully capture semantic and stylistic aspects of political translation; and second, that Test Set B represents a maximum-overlap benchmark sampled directly from the fine-tuning corpus rather than a held-out in-domain condition, which limits the extent to which those gains can be interpreted as transferable beyond matched materials. We believe this revision provides a more transparent and balanced statement of the study’s scope.

3.6 Comment on future research directions

We also revised the future-research discussion to make it more specific and actionab

Attachment Submitted filename: Response to Reviewers.docx

# Peer review: Decision Letter 1

**DOI:** 10.1371/journal.pone.0352256.r003

**Authors:** Zeheng Wang, Zeheng Wang

12 May 2026

PONE-D-26-09149R1

Textual Overlap Rather Than Domain Alignment: A Comparative Study of Fine-Tuning Strategies for Specialised Machine Translation with Large Language Models

PLOS One

Dear Dr. Yang,

Thank you for submitting your manuscript to PLOS ONE. After careful consideration, we feel that it has merit but does not fully meet PLOS ONE’s publication criteria as it currently stands. Therefore, we invite you to submit a revised version of the manuscript that addresses the points raised during the review process.

Please submit your revised manuscript by Jun 26 2026 11:59PM. If you will need more time than this to complete your revisions, please reply to this message or contact the journal office at plosone@plos.org. When you're ready to submit your revision, log on to https://www.editorialmanager.com/pone/ and select the 'Submissions Needing Revision' folder to locate your manuscript file.

Please include the following items when submitting your revised manuscript:

- A letter that responds to each point raised by the academic editor and reviewer(s). You should upload this letter as a separate file labeled 'Response to Reviewers'.
- A marked-up copy of your manuscript that highlights changes made to the original version. You should upload this as a separate file labeled 'Revised Manuscript with Track Changes'.
- An unmarked version of your revised paper without tracked changes. You should upload this as a separate file labeled 'Manuscript'.

If you would like to make changes to your financial disclosure, please include your updated statement in your cover letter. Guidelines for resubmitting your figure files are available below the reviewer comments at the end of this letter.

If applicable, we recommend that you deposit your laboratory protocols in protocols.io to enhance the reproducibility of your results. Protocols.io assigns your protocol its own identifier (DOI) so that it can be cited independently in the future. For instructions see: https://journals.plos.org/plosone/s/submission-guidelines#loc-laboratory-protocols. Additionally, PLOS ONE offers an option for publishing peer-reviewed Lab Protocol articles, which describe protocols hosted on protocols.io. Read more information on sharing protocols at https://plos.org/protocols?utm_medium=editorial-email&utm_source=authorletters&utm_campaign=protocols.

As the corresponding author, your ORCID iD is verified in the submission system and will appear in the published article. PLOS supports the use of ORCID, and we encourage all coauthors to register for an ORCID iD and use it as well. Please encourage your coauthors to verify their ORCID iD within the submission system before final acceptance, as unverified ORCID iDs will not appear in the published article. *Only*  the individual author can complete the verification step; PLOS staff *cannot*  verify ORCID iDs on behalf of authors.

We look forward to receiving your revised manuscript.

Kind regards,

Zeheng Wang

Academic Editor

PLOS One

Journal Requirements:

If the reviewer comments include a recommendation to cite specific previously published works, please review and evaluate these publications to determine whether they are relevant and should be cited. There is no requirement to cite these works unless the editor has indicated otherwise.

Please review your reference list to ensure that it is complete and correct. If you have cited papers that have been retracted, please include the rationale for doing so in the manuscript text, or remove these references and replace them with relevant current references. Any changes to the reference list should be mentioned in the rebuttal letter that accompanies your revised manuscript. If you need to cite a retracted article, indicate the article’s retracted status in the References list and also include a citation and full reference for the retraction notice.

[Note: HTML markup is below. Please do not edit.]

Reviewers' comments:

Reviewer's Responses to Questions

**Comments to the Author**

1. If the authors have adequately addressed your comments raised in a previous round of review and you feel that this manuscript is now acceptable for publication, you may indicate that here to bypass the “Comments to the Author” section, enter your conflict of interest statement in the “Confidential to Editor” section, and submit your "Accept" recommendation.

Reviewer #1: All comments have been addressed

Reviewer #2: All comments have been addressed

**********

2. Is the manuscript technically sound, and do the data support the conclusions?

The manuscript must describe a technically sound piece of scientific research with data that supports the conclusions. Experiments must have been conducted rigorously, with appropriate controls, replication, and sample sizes. The conclusions must be drawn appropriately based on the data presented.

Reviewer #1: Yes

Reviewer #2: Yes

**********

3. Has the statistical analysis been performed appropriately and rigorously?

Reviewer #1: Yes

Reviewer #2: Yes

**********

4. Have the authors made all data underlying the findings in their manuscript fully available?

The PLOS Data policy requires authors to make all data underlying the findings described in their manuscript fully available without restriction, with rare exception (please refer to the Data Availability Statement in the manuscript PDF file). The data should be provided as part of the manuscript or its supporting information, or deposited to a public repository. For example, in addition to summary statistics, the data points behind means, medians and variance measures should be available. If there are restrictions on publicly sharing data—e.g. participant privacy or use of data from a third party—those must be specified.

Reviewer #1: Yes

Reviewer #2: Yes

**********

5. Is the manuscript presented in an intelligible fashion and written in standard English?

PLOS ONE does not copyedit accepted manuscripts, so the language in submitted articles must be clear, correct, and unambiguous. Any typographical or grammatical errors should be corrected at revision, so please note any specific errors here.

Reviewer #1: Yes

Reviewer #2: Yes

**********

6. Review Comments to the Author

Please use the space provided to explain your answers to the questions above. You may also include additional comments for the author, including concerns about dual publication, research ethics, or publication ethics. (Please upload your review as an attachment if it exceeds 20,000 characters)

Reviewer #1: Overall Assessment

The manuscript presents a comparative study on the use of Full-Parameter Fine-Tuning (FPFT) and Parameter-Efficient Fine-Tuning (PEFT) in large language models for specialized machine translation. Based on an experiment conducted with Chinese–English political discourse data using the Qwen-3-14B model, it is indicated that the choice of technique, as well as the selection of the model, depends on the characteristics of the dataset and the constraints imposed by the available computational resources.

The manuscript addresses a relevant topic within Natural Language Processing and provides a well-structured and methodologically sound contribution. Only minor enhancements are required to further strengthen the robustness of the experimental evaluation.

Strengths

The manuscript addresses an important problem in the field of Natural Language Processing, namely the comparative evaluation of Full-Parameter Fine-Tuning (FPFT) and Parameter-Efficient Fine-Tuning (PEFT) strategies for specialized machine translation in large language models.

The experiments are conducted using the Qwen-3-14B model, which represents a modern architecture and increases the relevance of the findings for current research on large language models.

The study is supported by experimental evaluation using Chinese–English political discourse data, providing practical evidence for the discussion of fine-tuning strategies.

The manuscript appropriately highlights the role of computational resources in selecting between FPFT and PEFT approaches, which is highly relevant for real-world deployments.

The use of unseen domain data (Set A) and familiar domain data (Set B) in Section 4.5 demonstrates an effort to assess model generalization and domain adaptation, which is a strong methodological choice.

The results contribute to understanding how different fine-tuning approaches perform under specific domain and resource conditions, offering insights useful for practitioners.

Statistical comparisons of the results are presented, contributing to the reliability of the findings.

Weaknesses

The evaluation is limited by the use of a single primary metric, which restricts the comprehensiveness and robustness of the performance analysis.

Minor Comments

The inclusion of a third dataset composed of cases that are semantically similar to Set B is recommended. This addition would strengthen the discussion on domain specialization by enabling a more nuanced evaluation of generalization within closely related semantic contexts.

The evaluation framework should be expanded to include additional metrics, such as ROUGE, METEOR, and BERTScore. The use of multiple complementary metrics would enhance the scientific robustness of the results. This recommendation becomes particularly important if the suggested third dataset is incorporated, as it would allow for a more comprehensive and reliable comparison across different evaluation scenarios.

Recommendation

Minor Revision

The manuscript addresses a relevant topic and presents a useful empirical comparison of fine-tuning strategies for large language models. The study is now well structured and methodologically sound, with only minor enhancements suggested to further strengthen the experimental evaluation. Two additional points are proposed which would further improve the robustness of the research.

Reviewer #2: (No Response)

**********

7. PLOS authors have the option to publish the peer review history of their article (what does this mean?). If published, this will include your full peer review and any attached files.

If you choose “no”, your identity will remain anonymous but your review may still be made public.

**Do you want your identity to be public for this peer review?**  For information about this choice, including consent withdrawal, please see our Privacy Policy.

Reviewer #1: **Yes:**  Raphael Souza de Oliveira

Reviewer #2: No

**********

[NOTE: If reviewer comments were submitted as an attachment file, they will be attached to this email and accessible via the submission site. Please log into your account, locate the manuscript record, and check for the action link "View Attachments". If this link does not appear, there are no attachment files.]

To ensure your figures meet our technical requirements, please review our figure guidelines: https://journals.plos.org/plosone/s/figures

You may also use PLOS’s free figure tool, NAAS, to help you prepare publication quality figures: https://journals.plos.org/plosone/s/figures#loc-tools-for-figure-preparation.

NAAS will assess whether your figures meet our technical requirements by comparing each figure against our figure specifications.

Attachment Submitted filename: Review 2 - Reviewr 1.pdf

# Author comment: Author response to Decision Letter 2

**DOI:** 10.1371/journal.pone.0352256.r004

7 Jun 2026

Manuscript ID: PONE-D-26-09149R1

Title: Textual Overlap Rather Than Domain Alignment: A Comparative Study of Fine-Tuning Strategies for Specialised Machine Translation with Large Language Models

Response to the Academic Editor

We appreciate the opportunity to revise the manuscript. In response to the Academic Editor’s and reviewer’s comments, we have submitted a revised manuscript, an updated protocol(DOI: https://dx.doi.org/10.17504/protocols.io.kqdg3re7pg25/v1 [Private link for reviewers: https://www.protocols.io/private/83938F755CCD11F18D230A58A9FEAC02 to be removed before publication.]), revised supporting files, and this response letter. The main revision expands the evaluation design to include Test Sets A, B, and C and four complementary automatic translation-quality metrics: BLEU, ROUGE-L F1, METEOR, and BERTScore F1. The revised S2 Dataset contains test-set metadata, item-level metric scores, aggregated metric summaries, BLEU pass-rate analyses, paired t-tests with paired effect sizes, and Fig 5 source data; the revised S3 File contains the scripts and source files needed to reproduce the metric scores, figure files, and table-data outputs.

Response to Reviewer #1

Comment 1: The inclusion of a third dataset composed of cases that are semantically similar to Set B is recommended. This addition would strengthen the discussion on domain specialization by enabling a more nuanced evaluation of generalization within closely related semantic contexts.

Response: Thank you for this helpful suggestion. We constructed Test Set C as a 50-item political-discourse test set that is semantically related to Set B but was not included in the fine-tuning corpus. Test Set C belongs to the same broad Chinese-English political-discourse domain and is close to Set B in content type, but its source texts and reference translations were not used for fine-tuning. This allowed us to test whether the strong performance observed on the maximum-overlap Set B transfers to closely related but non-overlapping material. Across the four automatic metrics, Set C did not show the maximum-overlap gains observed on Set B; BLEU pass rates were 12% for the base model, 8% for PEFT, and 8% for FPFT, with no significant pass-rate differences between model pairs. This strengthens the central conclusion that textual overlap, rather than broad domain similarity alone, conditions the observed benefit of fine-tuning. We revised the Methods, Results, Discussion, Conclusion, protocol, S1 File, and S2 Dataset accordingly.

Comment 2: The evaluation framework should be expanded to include additional metrics, such as ROUGE, METEOR, and BERTScore. The use of multiple complementary metrics would enhance the scientific robustness of the results.

Response: We agree and have expanded the evaluation framework. In addition to BLEU, we computed ROUGE-L F1, METEOR, and BERTScore F1 for all translations produced by the base model, PEFT model, and FPFT model on Test Sets A, B, and C. BERTScore F1 was computed using the official bert-score 0.3.12 package with a local roberta-large model and the recommended English layer 17. The exact metric configuration is now documented in S1 File and the S3 File README: sacrebleu 2.6.0 sentence BLEU on a 0-1 scale, word-level ROUGE-L F1, NLTK 3.9.1 meteor_score, and official bert-score 0.3.12 with local roberta-large. The revised Table 3 reports the mean values of all four metrics, and Fig 5 visualizes the metric patterns across models and test sets. The updated S2 Dataset includes item-level metric scores, aggregated metric summaries, BLEU pass-rate likelihood-ratio G2 tests, paired t-tests for the four continuous metrics, and paired Cohen’s dz effect-size values. In the revised Results and Discussion, we now report the corresponding t statistics, p values, and dz values for the key comparisons, rather than referring to paired t-tests without numerical evidence. To strengthen reproducibility, we also added calculate_translation_metrics.py to S3 File so that readers can generate item_level_metrics.csv directly from the nine source translation workbooks and then reproduce Table 3, Table 4, Table 5, and Fig 5 from the same item-level metric file. The multi-metric results support the original conclusion while making it more robust: fine-tuned models perform strongly under maximum textual overlap (Set B), but the advantage is not reproduced on the unseen Set A or the semantically related but non-overlapping Set C.

Attachment Submitted filename: Response_to_Reviewers_auresp_2.docx

# Editor report: Decision Letter 2

**DOI:** 10.1371/journal.pone.0352256.r005

**Authors:** Zeheng Wang, Zeheng Wang, Zeheng Wang

8 Jun 2026

Textual Overlap Rather Than Domain Alignment: A Comparative Study of Fine-Tuning Strategies for Specialised Machine Translation with Large Language Models

PONE-D-26-09149R2

Dear Dr. Yang,

We’re pleased to inform you that your manuscript has been judged scientifically suitable for publication and will be formally accepted for publication once it meets all outstanding technical requirements.

Within one week, you’ll receive an e-mail detailing the required amendments. When these have been addressed, you’ll receive a formal acceptance letter and your manuscript will be scheduled for publication.

An invoice will be generated when your article is formally accepted. Please note, if your institution has a publishing partnership with PLOS and your article meets the relevant criteria, all or part of your publication costs will be covered. Please make sure your user information is up-to-date by logging into Editorial Manager at Editorial Manager® and clicking the ‘Update My Information' link at the top of the page. For questions related to billing, please contact billing support.

If your institution or institutions have a press office, please notify them about your upcoming paper to help maximize its impact. If they’ll be preparing press materials, please inform our press team as soon as possible -- no later than 48 hours after receiving the formal acceptance. Your manuscript will remain under strict press embargo until 2 pm Eastern Time on the date of publication. For more information, please contact onepress@plos.org.

Kind regards,

Zeheng Wang

Academic Editor

PLOS One

Additional Editor Comments (optional):

Reviewers' comments:

# Editor report: Acceptance letter

**DOI:** 10.1371/journal.pone.0352256.r006

**Authors:** Zeheng Wang, Zeheng Wang, Zeheng Wang

PONE-D-26-09149R2

PLOS One

Dear Dr. Yang,

I'm pleased to inform you that your manuscript has been deemed suitable for publication in PLOS One. Congratulations! Your manuscript is now being handed over to our production team.

At this stage, our production department will prepare your paper for publication. This includes ensuring the following:

* All references, tables, and figures are properly cited

* All relevant supporting information is included in the manuscript submission,

* There are no issues that prevent the paper from being properly typeset

You will receive further instructions from the production team, including instructions on how to review your proof when it is ready. Please keep in mind that we are working through a large volume of accepted articles, so please give us a few days to review your paper and let you know the next and final steps.

Lastly, if your institution or institutions have a press office, please let them know about your upcoming paper now to help maximize its impact. If they'll be preparing press materials, please inform our press team within the next 48 hours. Your manuscript will remain under strict press embargo until 2 pm Eastern Time on the date of publication. For more information, please contact onepress@plos.org.

You will receive an invoice from PLOS for your publication fee after your manuscript has reached the completed accept phase. If you receive an email requesting payment before acceptance or for any other service, this may be a phishing scheme. Learn how to identify phishing emails and protect your accounts at https://explore.plos.org/phishing.

If we can help with anything else, please email us at customercare@plos.org.

Thank you for submitting your work to PLOS ONE and supporting open access.

Kind regards,

PLOS ONE Editorial Office Staff

on behalf of

Dr. Zeheng Wang

Academic Editor

PLOS One
